# Identification of Complex Rumen Microbiome Interaction Within Diverse Functional Niches as Mechanisms Affecting the Variation of Methane Emissions in Bovine

**DOI:** 10.3389/fmicb.2020.00659

**Published:** 2020-04-17

**Authors:** Marina Martínez-Álvaro, Marc D. Auffret, Robert D. Stewart, Richard J. Dewhurst, Carol-Anne Duthie, John A. Rooke, R. John Wallace, Barbara Shih, Tom C. Freeman, Mick Watson, Rainer Roehe

**Affiliations:** ^1^Scotland’s Rural College, Edinburgh, United Kingdom; ^2^Institute for Animal Science and Technology, Polytechnic University of Valencia, Valencia, Spain; ^3^Edinburgh Genomics, The Roslin Institute and R(D)SVS, The University of Edinburgh, Edinburgh, United Kingdom; ^4^The Rowett Institute, University of Aberdeen, Aberdeen, United Kingdom; ^5^Division of Genetics and Genomics, The Roslin Institute and R(D)SVS, The University of Edinburgh, Edinburgh, United Kingdom

**Keywords:** rumen microbiome, network analysis, methane emissions, functional niches, metagenomics

## Abstract

A network analysis including relative abundances of all ruminal microbial genera (archaea, bacteria, fungi, and protists) and their genes was performed to improve our understanding of how the interactions within the ruminal microbiome affects methane emissions (CH_4_). Metagenomics and CH_4_ data were available from 63 bovines of a two-breed rotational cross, offered two basal diets. Co-abundance network analysis revealed 10 clusters of functional niches. The most abundant hydrogenotrophic *Methanobacteriales* with key microbial genes involved in methanogenesis occupied a different functional niche (i.e., “methanogenesis” cluster) than methylotrophic *Methanomassiliicoccales* (Candidatus *Methanomethylophylus*) and acetogens (*Blautia*). Fungi and protists clustered together and other plant fiber degraders like *Fibrobacter* occupied a seperate cluster. A Partial Least Squares analysis approach to predict CH_4_ variation in each cluster showed the methanogenesis cluster had the best prediction ability (57.3%). However, the most important explanatory variables in this cluster were genes involved in complex carbohydrate degradation, metabolism of sugars and amino acids and Candidatus *Azobacteroides* carrying nitrogen fixation genes, but not methanogenic archaea and their genes. The cluster containing *Fibrobacter*, isolated from other microorganisms, was positively associated with CH_4_ and explained 49.8% of its variability, showing fermentative advantages compared to other bacteria and fungi in providing substrates (e.g., formate) for methanogenesis. In other clusters, genes with enhancing effect on CH_4_ were related to lactate and butyrate (*Butyrivibrio* and *Pseudobutyrivibrio*) production and simple amino acids metabolism. In comparison, ruminal genes negatively related to CH_4_ were involved in carbohydrate degradation via lactate and succinate and synthesis of more complex amino acids by γ-Proteobacteria. When analyzing low- and high-methane emitters data in separate networks, competition between methanogens in the methanogenesis cluster was uncovered by a broader diversity of methanogens involved in the three methanogenesis pathways and larger interactions within and between communities in low compared to high emitters. Generally, our results suggest that differences in CH_4_ are mainly explained by other microbial communities and their activities rather than being only methanogens-driven. Our study provides insight into the interactions of the rumen microbial communities and their genes by uncovering functional niches affecting CH_4_, which will benefit the development of efficient CH_4_ mitigation strategies.

## Introduction

By 2050, the human population will grow to over 9 billion people, and in the same time frame, global meat consumption is projected to increase by 73% (FAO, 2011). Ruminant agriculture plays a key role in maintaining and enhancing provision of protein and essential micronutrients to humans. However, intensive food production affects the environment with the release of greenhouse gas (GHG) emissions (Johnson and Johnson, 1995). Ruminants are major emitters of methane (CH_4_), a GHG being 28-fold more potent than carbon dioxide (IPCC, 2014) and accounting for 37% of total GHG from agriculture in the United Kingdom (Cottle et al., 2011).

Future ruminant production systems will need to capitalize on their ability to utilize human inedible ligno-cellulose material for animal production, but will also need to select animals releasing less CH_4_ as an end product of anaerobic microbial fermentation in the rumen.

A limited number of archaeal taxa within Euryarchaeota are responsible for CH_4_ production in the rumen, using substrates released from organic matter fermentation. Methane can be synthesized following three different pathways (hydrogenotrophy, methylotrophy, and acetoclastic methanogenesis) and the genes involved in methanogenesis are well characterized (Thauer et al., 2008; Leahy et al., 2010; Borrel et al., 2013). However, new methanogens are still being discovered (Poulsen et al., 2013; Vanwonterghem et al., 2017; Stewart et al., 2018). In contrast with methanogenesis, microbial fermentation is conducted by complex and diverse microbial populations composed of bacteria, protozoa and fungi potentially sharing similar genes and functions, interacting together, adapting to different environments (e.g., diet change) and playing a central role in the ability of ruminants to utilize fibrous substrates. Bacterial populations interacting with methanogens that utilize H_2_ or involved in different metabolic pathways associated with amino acids, lactate or volatile fatty acids (VFA) are known to have different effects on CH_4_ emissions (Moss et al., 2000; Janssen, 2010; Wanapat et al., 2015; Kamke et al., 2016; Sa et al., 2016). In addition, several authors revealed the importance of interactions between bacteria, fungi, protists (protozoa and micro-algae) and archaea in their effects on CH_4_ emissions (Kumar et al., 2015; Wang et al., 2017; Huws et al., 2018).

Several authors have succeeded in using information about microbial communities or microbial genes to predict CH_4_ emissions (Roehe et al., 2016; Shabat et al., 2016; Auffret et al., 2018; Difford et al., 2018) but restricted to archaea and bacteria communities. However, in order to develop efficient CH_4_ mitigation strategies using microbiome information, we need improved knowledge about the rumen microbiome. In particular, we need to apply microbial ecology principles including niche occupancy potentially associated with a specific function, selective pressure, adaptation, and interactions (Weimer, 1998) that will help explain the relevance of each domain associated with differences in CH_4_ emissions.

Recently, the ruminal microbiome was explored using a combination of culturing and sequencing as in the Hungate 1000 collection (Seshadri et al., 2018). However, the limitations of culturing techniques need to be alleviated prior to fully represent the rumen microbiome (Seshadri et al., 2018). Alternatively, the development of metagenomic binning as a bioinformatics tool enabled near-complete microbial genomes to be assembled directly from metagenomic sequencing data. This methodology was successfully applied in different ecosystems (Parks et al., 2017) including the bovine (Stewart et al., 2018) and moose (Svartström et al., 2017) rumen and substantially improved the coverage of rumen microbial genomes (Stewart et al., 2018, 2019).

Co-abundance network analysis helps to represent the complexity behind intra- and inter-domain interactions within the rumen microbiome as a whole, largely overcoming the limitations of culture based or molecular genetic analysis to study these interactions, and identify microbial groups related to function (Janssen and Kirs, 2008; Gruninger et al., 2014; Henderson et al., 2015; Taxis et al., 2015). Co-abundance patterns between microbials have been previously used as a prediction of microbial interactions (Faust and Raes, 2012). Network-based analytical approaches have helped disentangle complex polymicrobial and microbe–host interactions in ruminants, humans and soil (Barberán et al., 2012; Roehe et al., 2016; Sung et al., 2017; Auffret et al., 2018) by identifying patterns of microbial interactions in ecosystems occupied by highly diverse microorganisms. Within a network, several clusters considered as a single biological unit may provide information about the local interaction patterns, the biological contribution of each cluster and therefore its function in the microbiome (reviewed in Faust and Raes, 2012).

In the present study we are combining co-abundances networks of the microbial communities, not only bacteria and archaea but also fungi and protists, and their genes. This study highlights the importance of microbial interactions of different domains within functional niches compared to variation in microbial composition or abundances. One highlight of this analysis is the identification of functional niches within the rumen microbiome differently explaining variations in methane emissions and that microbial domains and functions other than methanogenesis affect mainly the variation in methane emissions from bovine.

## Materials and Methods

### Animals, Experimental Design, and Diets

Our animal experiment was carried out in 2011 (Rooke et al., 2014; Wallace et al., 2015; Roehe et al., 2016) and used a 2 × 2 factorial design of breed types and diets, with 72 crossbred Aberdeen Angus (AA) and Limousin (LIM) steers. The animals were offered one of two complete diets *ad libitum* consisting (g/kg DM) of 480 forage to 520 concentrate or 80 straw to 920 concentrate – which are subsequently referred to as forage (FOR) and concentrate (CONC) diets, respectively. Breed type and diet were balanced within experiment. The detailed diet composition has been reported previously by Rooke et al. (2014). Animals were fed *ad libitum* and had free access to drinking water throughout the experiment. The animals had an average age of 521 ± 30 days and weight of 673 ± 35 kg before entering individually in the six available respiration chambers. Further descriptions of animal data and farm conditions [breed, diet, experimental design, feeding, husbandry over the entire trial are available in Rooke et al. (2014)]. Methane emissions were successfully measured from 63 animals individually for 48 h in respiration chambers (Rooke et al., 2014). The animals were fed *ad libitum* until they left the farm and were slaughtered within 3 h at a commercial abattoir where two samples of rumen digesta (approximately 50 mL) were taken immediately after the rumen was opened to be drained. The main advantage of collecting rumen contents after slaughter is to obtain samples that are representative of both solid and liquid phases.

### Genomic Analysis

DNA was extracted from the rumen digesta samples following the protocol from Yu and Morrison (2004) and was based on repeated bead beating with column filtration. The procedure is fully described in Rooke et al. (2014). Sixty-three rumen digesta samples including the eight animals used in Roehe et al. (2016) selected for extreme CH_4_ emissions were prepared for sequencing; the remaining nine animals of the experiment did not yield rumen samples of sufficient quality for metagenomics analysis or failed during methane measurements. Therefore, there were samples from 63 animals left where we had both methane measurements and high-quality rumen digesta samples.

Illumina TruSeq libraries were prepared from DNA from rumen samples and sequenced on an Illumina HiSeq 4000 instrument by Edinburgh Genomics (Edinburgh, United Kingdom). Paired-end reads (2 × 100 bp) were generated, resulting in between 8 and 15 GB per sample (between 40 and 73 million paired sequence reads) with on average 73% passing quality check and being subsequently annotated. Bioinformatics analysis followed the same procedure as previously described in Wallace et al. (2015) and Roehe et al. (2016). In order to measure the abundance of known microbial genes in the rumen samples, reads from whole metagenome sequencing were aligned to the Kyoto Encyclopedia of Genes and Genomes (KEGG^1^) database using Novoalign^2^. Parameters were adjusted such that all hits reported were equal in quality to the best hit for each read, and allowing up to a 10% mismatch across the fragment. The KEGG Ortholog groups (KO) of all hits that were equal to the best hit were examined. In the case we were unable to resolve the sequence read to a single KO, the read was ignored; otherwise, the read was assigned to the unique KO. Statistical analysis of the metagenomics samples was based on the complete sample profiles as expressed by the pattern of metagenomic sequence reads classified within KEGG ortholog groups with >90% similarity and belonging to a single KEGG ortholog (KO) groups. The alignment of the reads generated by whole metagenomic sequencing to the KEGG genes database resulted in identification of 4,427 microbial genes for each animal. Microbial genes were expressed in relative abundance (percentage) within animal and only those with a relative abundance greater than 0.001% (*n* = 1,936) were carried forward for downstream analysis.

For phylogenetic annotation, the sequence reads were aligned to a custom database using Kraken software combining several databases including genomes from the Hungate 1000 collection and metagenome-assembled genomes (MAGs) from beef rumen samples (Wood and Salzberg, 2014; Stewart et al., 2018). In total, 1,178 genera were identified and described as the genus having the highest similarity with the identified microbial genome or MAG, applying the same cutoff used in previous MAG studies (Parks et al., 2017; Svartström et al., 2017; Stewart et al., 2018) (estimated completeness ≥80% and estimated contamination ≤10%). As for microbial genes, microbial genera identified were normalized between animals expressing them as relative abundances.

Microbial KEGG genes and genera with zero counts in 3 or more of the 63 animals were removed from the analysis to avoid statistical limitations due to interferences in the study of co-abundances (Faust and Raes, 2012). Following this step, 1,557 genes and 1,160 genera were selected for the statistical analysis.

The raw sequencing data can be downloaded from the European Nucleotide Archive under accession PRJEB10338 and PRJEB31266.

### Co-abundance Network Analysis

**FIGURE 1:**
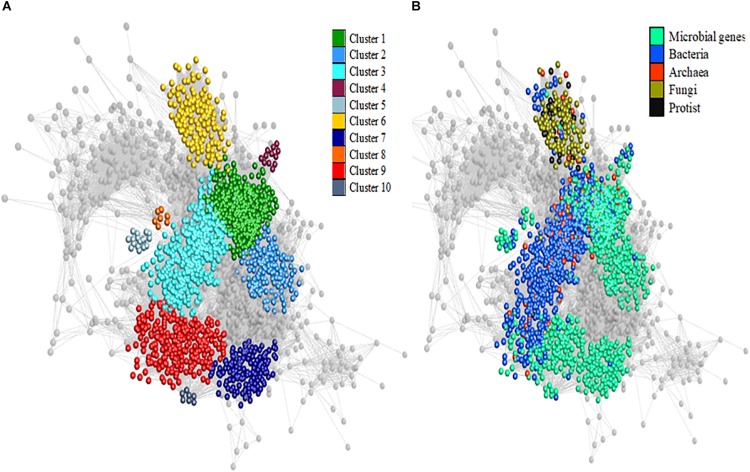
Functional clusters composed of microbial genera and genes generated using co-abundance network analysis in beef cattle. **(A)** Distribution of the clusters in the network. **(B)** Distribution of genes and microbial genera (bacteria, archaea, fungi, and protist) among the clusters. Nodes represent microbial genera and genes, and edges illustrate co-abundances between their relative abundances. Networks were clustered using the MCL algorithm, and clusters 1 to 10 are shown. Only variables with correlation values greater than 0.70 between nodes were kept during the analysis. Cluster 1 containing most abundant methanogenic archaea (*Methanobrevibacter*, *Methanosphaera*, and *Methanosarcina*), and microbial genes involved in methanogenesis pathway, and also bacteria (*Sarcina*), fungi (*Tremella*) and genes in degradation pathways for amino acids (nitrogen fixation capacity of Candidatus *Azobacteroides*) and carbohydrates, was referred to as methanogenesis cluster. Cluster 2 includes only genus *Fibrobacter* and microbial genes involved in the synthesis of central metabolic enzymes. Cluster 3 is mainly comprised of bacteria of the phyla Firmicutes, Proteobacteria, and Acidobacteria with low abundant archaea, some of them methanogen. Cluster 4 is a small cluster containing *Butyrivibrio*, *Pseudobutyrivibrio* and few microbial genes related to sugar metabolism. Cluster 5 is also a reduced cluster containing *Bacillus*, other bacteria and genes related to sugar degradation. Cluster 6 is dominated by genera of the fungal community, and three hydrogenotrophic and/or acetoclastic methanogens. Cluster 7 included *Bifidobacterium* and microbial genes relevant for carbohydrate degradation. Cluster 8 contained *Prevotella* with genes involved in nitrogen metabolism and pentose phosphate pathway. Cluster 9 contained the methylotrophic *Methanomassiliicoccales* Candidatus *Methanomethylophilus*, the acetogens *Eubacterium*, *Blautia*, and *Acetitomaculum* and a high diversity of Proteobacteria (mainly γ-Proteobacteria) and microbial genes involved in carbohydrates, lipids, and aminoacids metabolism. Cluster 10 includes *Selenomonas* and few microbial genes related to oligosaccharide transport.

The interactions among all microbial genes and genera (2,717 variables in total) were investigated from the rumen microbiome of the 63 animals in a co-abundance network analysis using Miru software [Kajeka Ltd., Edinburgh, Freeman et al. (2007)]. The applied procedure to generate the network is fully described in Freeman et al. (2007). Briefly, the network grouped variables based on Pearson correlation and a MCL algorithm is applied to cluster the network according to connectivity and local structure. The software receives back from MCL algorithm a list of nodes and their cluster assignments. These cluster assignments are added to the network as annotation data and provide a basis for statistical analysis of annotation terms across clusters. In our study, a positive correlation threshold of 0.70 filtered out 217 variables that were not correlated (*r* < 0.70) to any other microbial variables, leaving 2,500 variables that constituted the network with 43 clusters identified in total. The combination of previous knowledge on variables associated with CH_4_ emissions (Wallace et al., 2015; Roehe et al., 2016; Auffret et al., 2018) and the results from the network analysis allowed us to identify 10 different functional niches potentially involved in CH_4_ emissions, corresponding to different clusters (Supplementary Table S1A and Figure 1).

Due to the compositional nature of the metagenomics data, artefactual co-abundances between variables may appear. Converting data into log ratio coordinates is an adequate approach to attenuate this problem (Faust and Raes, 2012; Greenacre, 2018). Then, we compared the network analysis results obtained using relative abundance data with those using log ratio coordinates generated by SPARCC software (Friedman and Alm, 2012) in order to identify the impact of potential compositional bias on the results (Supplementary Table S1B and Supplementary Figure S2).

### Influence of Each Cluster in CH_4_ Emissions

The influence of each of the 10 clusters on CH_4_ emissions was studied by Partial Least Squares analysis (PLS), performing a different PLS model per cluster. Each model was built considering CH_4_ emissions as dependent variable, diet and breed as fixed effects and microbial genes and communities composing each cluster as explanatory variables [PLS, R 3.4.3 statistical software, mixOmics package (Le Cao et al., 2016)]. The most influential variables from each cluster that were important in explaining CH_4_ emissions were selected based on the variable importance for projection (VIP) criterion (Wold, 1995) built on one latent component whereby microbial parameters with a VIP <0.8 contribute little to the prediction, and on our previous biological knowledge (Wallace et al., 2015; Roehe et al., 2016; Auffret et al., 2018). Then, the importance of each cluster explaining the variability of CH_4_ emissions was tested by a final PLS with CH_4_ as dependent variable and the variables selected per cluster as explanatory variables, without fixed effects. We used the Search Tool for the Retrieval of Interacting Genes (STRING) database (Szklarczyk et al., 2017) to get insight about the role of genes (metabolic pathways) identified as important by the final PLS and previously detected in the genome of microbial species within databases.

Microbial variables (genes and genera) selected by PLS were analyzed with Linear Discriminant Analysis (LDA), performed in R version 3.6.0 (2019-04-26) package MASS_7.3-51.4, to analyze the accuracy of discrimination between high (HME) and low CH_4_ emitters (LME).

### Animal Grouping, Statistical Analysis, and Separate Co-abundance Networks

Based on methane measurements recorded in respiration chamber, 31 animals were considered as LME whilst the other 32 animals were classified as HME. Due to a final number of 63 animals studied, this classification based on CH_4_ emissions is only partly balanced by breed type and diet with HME composed of 10 AA and 7 LIM fed CONC and 7 AA and 8 LIM fed FOR whilst LME comprised 7 AA and 7 LIM fed CONC and 10 AA and 7 LIM fed FOR. The difference between HME and LME in CH_4_ emissions (g/kg DMI), was estimated with a model including group (HME and LME), breed (AA and LIM) and diet (FOR and CONC) as fixed effects [GLM analysis, ‘lsmeans’ R package, R version 3.6.0 (2019-04-26)]. Residuals were assumed to be normally distributed.

Additionally, data from HME or LME animals were analyzed in separate networks (correlation threshold of 0.70) to identify any differences in cluster composition and microbiome interactions (genera and genes) by enrichment analysis using the option in Miru. Enrichment analysis compared variables/nodes significantly different (*P* < 0.05) between LME and HME animals.

A Venn diagram was generated using Venny software (Oliveros, 2007-2015) to compare the cluster composition for the cluster containing most abundant methanogens and microbial genes involved in methanogenesis between HME or LME animals.

## Results

### Systemic Factors Influencing CH_4_ Emissions

The distribution of CH_4_ emissions from 63 beef cattle overall and for groups of high and low CH_4_ emitters (HME and LME), forage and concentrate diets (FOR and CONC) and crossbred Aberdeen Angus (AA) and Limousin (LIM) steers are illustrated in Supplementary Figures S1A,B, respectively. Average CH_4_ emissions were 17.56 g/kg dry matter intake (DMI), with a coefficient of variation of 12.5%. High methane-emitting animals had 5.73 g greater CH_4_/kg DMI than LME (*P* < 0.001), which is equivalent to 2.61 standard deviations of this trait. Methane emissions were also greater in animals fed with forage in comparison to concentrate, with a difference of 8.48 g CH_4_/kg DMI (*P* < 0.001). Breed type effect was not significant for CH_4_ emissions per kg DMI.

### Composition of the 10 Clusters in the Rumen Microbiome of 63 Beef Cattle

A co-abundance network analysis was applied on the relative abundances of 1,557 microbial genes and 1,160 genera identified by metagenomics sequencing. A positive correlation cutoff of 0.70 was applied (Figures 1A,B). Among the clusters generated by network analysis, 10 individual clusters (1,565 variables within these clusters) corresponding to different functional niches and considered as important to explain differences in CH_4_ emissions were selected for further analysis (Supplementary Table S1A). In parallel, a network analysis was repeated with relative abundance data transformed in log ratio coordinates using SPARCC. Such strategy can help to reduce potential compositionality bias yielding to artefactual correlations. Results obtained showed a similar correlation structure between variables and similar composition of larger clusters compared to the network obtained with relative abundance data (Supplementary Table S1B and Supplementary Figure S2). Therefore, the following description of the 10 clusters will be based on results obtained with relative abundance data.

The largest cluster identified was cluster 1 (Figure 1) and contained 329 microbial genes, mostly involved in the CH_4_ synthesis, degradation pathways for amino acids and carbohydrates as confirmed using STRING database, as well as 98 genera (69 bacteria, 15 archaea, 13 fungi, and 1 protist). In terms of abundance, archaea-related genera were the most abundant in this cluster (5.81% of the total abundance of microbes), represented the three methanogenic pathways and were dominated by *Methanobrevibacter* (5.69%). The next most abundant genera belonged to bacteria (3.56%), mostly composed of *Sarcina* (2.70%). Fungi and protist were less abundant (0.05 and 0.007%, respectively) in cluster 1. Cluster 1 is subsequently referred to as “methanogenesis cluster”.

Others clusters (2–6) were highly connected with cluster 1 in the network (Figure 1). These clusters were associated with particular bacteria and functions. For example, the small cluster 4 contained *Butyrivibrio* (2.51%) and *Pseudobutyrivibrio* (0.49%), two Firmicutes producing butyrate, and few genes related to sugar metabolism (glucose, K05350; rhamnose, K05989; galactosamine, K02474 and multiple sugar transport system, K10546) whilst small cluster 5 comprised *Bacillus* (0.18%), as well as other bacteria and genes related to sugar degradation (such as K00163 and K00627).

Cluster 2 (Figure 1) included only one genus *Fibrobacter* (1.74% of relative abundance), and 146 genes mainly involved in the synthesis of central metabolic enzymes (for instance, malate dehydrogenase K00029, alcohol dehydrogenase K00001, glutamate-5-semialdehyde dehydrogenase K00147 or aldehyde dehydrogenase K00128).

Furthermore, clusters 3 and 6 (composed of 409 and 143 nodes, respectively) mainly comprised microbial genera accounting for 8.19 and 6.27%, respectively, of the total microbial abundance in the rumen, and only few genes. In cluster 3, the main genera were from α, β, δ, and γ Proteobacteria (181), Actinobacteria (88) and Firmicutes (39) phyla, also interacting with 31 different genera of archaea, some of them identified as methanogens (such as *Methanosphaerula*, *Methanocella*, *Methanoculleus*, or *Methanolaicina*). In contrast, cluster 6 was dominated by genera of the fungal community (93) followed by 20 protist genera, eight Cyanobacteria, five Proteobacteria (γ and β) and three methanogen archaea (*Methanococcus*, *Methanocaldococcus*, and *Methanothermococcus*). Methanogen archaea in clusters 3 and 6 are capable of hydrogenotrophic and/or acetoclastic methanogenesis.

Whereas clusters 1–6 were closely connected, clusters 7–10 contained different microbial genera and were not directly connected with cluster 1 (Figure 1). Within this group of clusters, cluster 9 was the larger cluster combining 111 microbial genera (4.82% of the total microbial abundance in the rumen), with 140 genes. Most of the genera identified in these clusters belong to bacteria, within different phyla but dominated by γ-Proteobacteria (41/111) such as *Enterobacter* or *Methylomonas*, by other Proteobacteria (20/111), and by Firmicutes (25/111) such as *Lactobacillus* or *Eubacterium*. Genes in this cluster were involved in carbohydrate and amino acid degradation and in lipid metabolism. This cluster contained the genus Candidatus *Methanomethylophilus* following the methylotrophic methanogenic pathway and the acetogenes such as *Eubacterium*, *Blautia*, and *Acetitomaculum*. In cluster 10, *Selenomonas* genus (2.58%) was connected with 6 genes, some of which are involved in oligosaccharide transport (K10108 and K10110). Cluster 7 included *Bifidobacterium* (1.64% of relative abundance), a main lactate producer and oligosaccharide degrader, as well as 143 genes, some relevant for carbohydrate degradation (such as K00873 or K01193). The most abundant microbial genus in the rumen *Prevotella* (38.6%) was classified in a small cluster 8 (Figure 1) associated with 7 genes, some of which related to nitrogen metabolism (K02600 and K13043) and the pentose phosphate pathway (K01786).

### Identification of the Main Clusters and Variables Explaining Variability in CH_4_ Emissions

Partial Least Squares analysis models were performed per cluster to compare them together and determine their importance within the network at explaining variability in CH_4_ emissions monitored over the 63 animals. A maximum of 5 variables per cluster with the highest VIP values (>0.8) were selected. These variables explained most of the variability in CH_4_ emissions (Tables 1, 2).

**TABLE 1:** Microbial genera and genes that mainly explain the variability of methane (CH_4_) emissions within each cluster positively related to the trait.

**Description of genus or functional genes identified**		**PLS results**^1^	

Cluster 1: Variables explained 57.3% of the variation in CH_4_ emissions			
Phylum/Class//gene	Genus/KEGG gene id	VIP	Reg. Coef.
Bacteroidetes (Bacteria)	*Candidatus Azobacteroides*	1.03	0.177
Basidiomycota (Fungi)	*Tremella*	1.01	0.174
Nitrogen fixation protein NifB	K02585	0.99	0.171
Glycine *C*-acetyltransferase	K00639	0.99	0.17
Dihydroflavonol-4-reductase	K00091	0.98	0.169

Cluster 2: Variables explained 49.8% of the variation in CH_4_ emissions			

Phylum/gene	Genus/KEGG gene id	VIP	Reg. Coef.
Endo-1,4-beta-xylanase	K01181	1.02	0.154
Sulfonate/nitrate/taurine transport system ATP-binding protein	K02049	1.01	0.153
Hypothetical protein	K09702	1	0.151
Nitrogenase iron protein NifH	K02588	0.99	0.151
Fibrobacteres (Bacteria)	*Fibrobacter*	0.98	0.148

Cluster 3: Variables explained 36.0% of the variation in CH_4_ emissions			

Phylum/gene	Genus/KEGG gene id	VIP	Reg. Coef.
Bacteroidetes (Bacteria)	*Niastella*	1.05	0.138
β-Proteobacteria (Bacteria)	*Polaromonas*	1.04	0.136
Bacteroidetes (Bacteria)	*Salinibacter*	1.02	0.134
Acidobacteria (Bacteria)	*Acidobacterium*	0.99	0.129
Actinobacteria (Bacteria)	*Alloactinosynnema*	0.88	0.116

Cluster 4: Variables explained 26.9% of the variation in CH_4_ emissions			

Phylum/gene	Genus/KEGG gene id	VIP	Reg. Coef.
Firmicutes (Bacteria)	*Butyrivibrio*	1.25	0.152
Beta-glucosidase	K05350	1.02	0.123
Alpha-L-rhamnosidase	K05989	0.93	0.113
Firmicutes (Bacteria)	*Pseudobutyrivibrio*	0.89	0.108
Aquificae (Bacteria)	*Hydrogenobacter*	0.87	0.106

Cluster 5: Variables explained 13.2% of the variation in CH_4_ emissions			

Phylum/gene	Genus/KEGG gene id	VIP	Reg. Coef.
Spirochaetes (Bacteria)	*Sediminispirochaeta*	1.23	0.175
2-oxoglutarate dehydrogenase E1 component	K00164	1.23	0.175
Glycine dehydrogenase	K00281	0.85	0.12
Actinobacteria (Bacteria)	*Saccharomonospora*	0.81	0.115
Firmicutes (Bacteria)	*Bacillus*	0.79	0.112

Cluster 6: Variables explained 38.3% of the variation in CH_4_ emissions			

Phylum/gene	Genus/KEGG gene id	VIP	Reg. Coef.
Heterokonta (Protist)	*Aphanomyces*	1.06	0.159
Basidiomycota (Fungi)	*Tsuchiyaea*	1.03	0.153
Ascomycota (Fungi)	*Pochonia*	1.03	0.153
Euryarchaeota (Archaea)	*Methanocaldococcus*	0.96	0.143
Basidiomycota (Fungi)	*Fomitiporia*	0.92	0.138

*VIP, variable importance for projection; Reg. Coef., Regression Coefficient. ^1^In the PLS analysis CH_4_ emissions were fitted as dependent variable and microbial populations and genes as independent variables and separately analyzed for each cluster. Only the first factor was considered in the PLS analysis.*

**TABLE 2:** Microbial genera and genes that mainly explain the variability of methane (CH_4_) emissions within each cluster negatively related to the trait.

**Description of genus or functional genes identified**		**PLS results^1^**	

Cluster 7: Variables explained 27.1% of the variation in CH_4_ emissions			

Phylum/Class/gene	Genus/KEGG gene id	VIP	Reg. Coef.
Pyruvate kinase	K00873	1.16	−0.13
Homoserine *O*-succinyltransferase	K00651	1.01	−0.112
Branched-chain amino acid transport system permease protein	K01998	0.96	−0.107
Alanine-synthesizing transaminase	K14260	0.93	−0.104
Branched-chain amino acid transport system ATP-binding protein	K01995	0.92	−0.102

Cluster 8: Variables explained 14.9% of the variation in CH_4_ emissions			

Phylum/gene	Genus/KEGG gene id	VIP	Reg. Coef.
Uncharacterized protein	K06950	1.19	−0.174
Bacteroidetes (Bacteria)	*Prevotella*	0.97	−0.143
N utilization substance protein A	K02600	0.8	−0.118

Cluster 9: Variables explained 31.8% of the variation in CH_4_ emissions			

Phylum/gene	Genus/KEGG gene id	VIP	Reg. Coef.
γ-Proteobacteria (Bacteria)	*Leclercia*	1.05	−0.133
γ-Proteobacteria (Bacteria)	*Tolumonas*	1.02	−0.129
Euryarchaeota (Archaea)	Candidatus *Methanomethylophilus*	0.98	−0.125
γ-Proteobacteria (Bacteria)	*Moraxella*	0.98	−0.125
L-lactate dehydrogenase	K00016	0.97	−0.123

Cluster 10: Variables explained 24.6% of the variation in CH_4_ emissions			

Phylum/gene	Genus/KEGG gene id	VIP	Reg. Coef.
Maltose/maltodextrin transport system permease protein	K10110	1.06	−0.12
Maltose/maltodextrin transport system substrate-binding protein	K10108	1.04	−0.118
Firmicutes (Bacteria)	*Selenomonas*	1.04	−0.118
Peroxiredoxin Q/BCP	K03564	1	−0.114
Ethanolamine ammonia-lyase large subunit	K03735	0.84	−0.096

*VIP, variable importance for projection; Reg. Coef., Regression Coefficient. ^1^In the PLS analysis CH_4_ emissions were fitted as dependent variable and microbial populations and genes as independent variables and separately analyzed for each cluster. Only the first factor was considered in the PLS analysis.*

Variables selected from Clusters 1 to 6 showed positive regression coefficients with CH_4_ emissions (Table 1). Most of the variability observed in CH_4_ emissions was explained by the 5 variables in cluster 1 (57.3%) including genera and genes associated with nitrogen fixation capacity (Candidatus *Azobacteroides* and K02585), lignin degradation (*Tremella*), or genes involved in amino acid (glycine, K00639) or sugar (K00091) metabolism. Although the most abundant methanogens (e.g., *Methanobrevibacter* or *Methanosphaera*) or the genes involved in the CH_4_ synthesis pathway (e.g., K00399 for *mcrA*) composed cluster 1, these variables were not identified by PLS with the highest VIP values.

Within fiber degraders (clusters 2 and 6), *Fibrobacter* and a gene encoding for xylan degradation (K01181) explained more variability in CH_4_ emissions (49.8%) than fungi and protists (38.3%). In clusters 3, 4, and 5, the most important bacterial populations included the two butyrate producers *Butyrivibrio* and *Pseudobutyrivibrio* (cluster 4), salt resistant bacteria (cluster 3) and *Bacillus* (cluster 5). Genes associated with glycine metabolism (K00281) or sugar metabolism like glucose and rhamnose (K05350 and K05989) were also important. However, the ability of these clusters to predict CH_4_ emissions was equivalent to or below 36.0%.

Variables selected from clusters 7 to 10, not directly connected to cluster 1 in the network, showed negative regression coefficients (Table 2), indicating that their higher relative abundance will result in a reduction in CH_4_ emissions. The variability in CH_4_ emissions explained by these clusters ranges between 14.9 and 31.8%, with cluster 9 showing the largest effect. The results in cluster 9 are due to the presence of 3 relatively abundant γ-Proteobacteria (between 0.0036 and 0.0103%) including *Leclercia*, *Moraxella*, and *Tolumonas*, the methylotrophic *Methanomassiliicoccales* Candidatus *Methanomethylophilus* (average abundance of 0.0491%) and a gene associated with lactate metabolism (K00016).

In cluster 7, the variables with high VIP values were mostly genes encoding for amino acid metabolism activities (K00651, K01995, K01998, and K14260). In cluster 10, *Selenomonas* genus and genes involved in polysaccharide transport (K10108 and K10110) and ammonia production (K03735) were identified as the main variables resulting in reduced CH_4_ emissions. The most dominant bacteria in ruminant, *Prevotella* and two genes (K06950 and K02600) in cluster 8 explained only 14.9% of variability in CH_4_ emissions.

**FIGURE 2:**
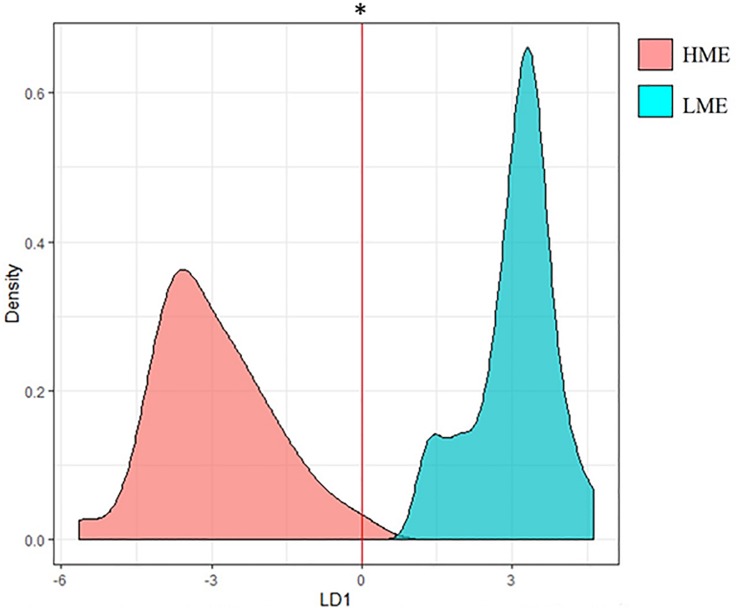
Linear Discriminant Analysis (LDA) density plot performed with microbial genera and functional genes previously selected by PLS showing their ability to discriminate between high (HME) and low (LME) methane emitters. HME, high methane emitters (light red color); LME, low methane emitters (light blue color). *LDA showed an accuracy value on prediction of 100%, all animals correctly assigned as HME or LME.

A Linear Discriminant Analysis using all microbial genes and genera selected by PLS within the 10 clusters confirmed the capacity to discriminate between HME and LME animals with a prediction accuracy of 100% (Figure 2).

### Changes in Methanogenesis Cluster Between HME and LME

**FIGURE 3:**
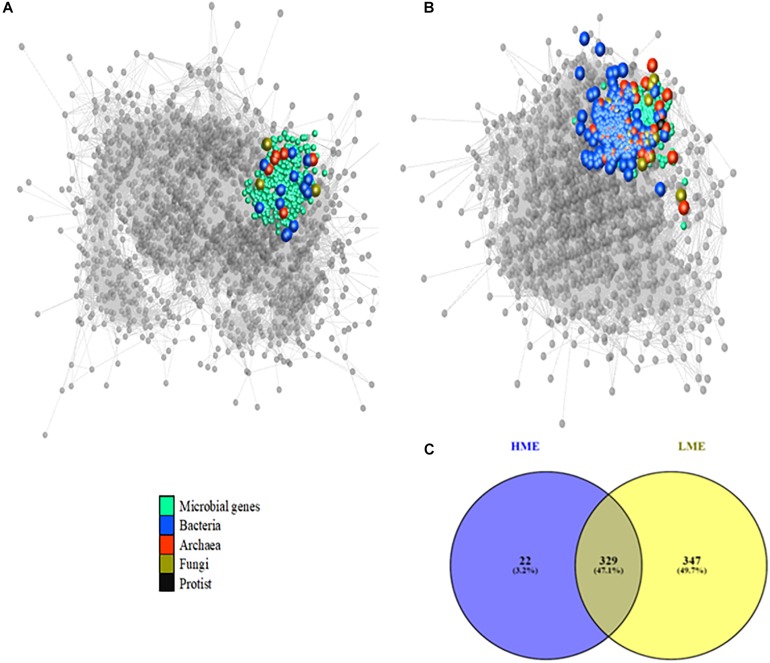
Focus on the “methanogenesis” cluster in co-abundance networks (correlation threshold of 0.70) in **(A)** high (HME) and **(B)** low (LME) methane emitters of beef cattle. This cluster contains the main methanogens and genes involved in methane synthesis. Larger nodes represent microbial genera whilst smaller ones represent microbial genes. Edges represent the correlation between their abundances. **(C)** Venn diagram showing 329 genera and genes present in both groups, whereas 22 and 347 are exclusively in HME or LME, respectively.

Two network analyses were performed in parallel with HME or LME data to compare the differences in the co-abundance structure and variables (Figures 3A,B). The composition of clusters between HME and LME networks was compared by enrichment analysis, in both directions, using Miru (Supplementary Tables S2A,B). The main differences (*P* < 0.01) were explained by the cluster containing the most abundant methanogens (*Methanobrevibacter*, *Methanosarcina*, and *Methanosphaera*) and their genes involved in CH_4_ synthesis (e.g., K00203, K00400, or K14128), corresponding to the methanogenesis cluster 1 in Figure 1.

The methanogenesis cluster in LME contained more nodes and edges than in HME (see Figure 3 and Supplementary Table S3). This can be explained by the addition of nodes within this cluster in LME related to other bacterial and archaeal genera identified within the methanogenesis cluster and other clusters in HME. Genes classified in the methanogenesis cluster and shared between HME and LME animals were involved in amino acid (e.g., K00186, K00187, and K00188) or carbohydrate degradation (e.g., K01959 and K01622), nitrogen fixation capacity (e.g., K02585), and biosynthesis of cofactors and vitamins (e.g., K03750, K03752, or K03753; Supplementary Table S3A).

As a main result, only fourteen bacterial genera (e.g., Candidatus *Azotobacteroides* and *Sarcina*) and three fungal genera (e.g., *Tremella*) clustered with the main methanogens in HME (Figure 3A and Supplementary Tables S3A,B) whilst a higher number of bacteria (*n* = 271), fungi (*n* = 19), and other archaea (*n* = 37) including other methanogens such as *Methanomicrobium* or *Methanosaeta* were identified in LME (Figure 3B and Supplementary Tables S3A,C). These additional populations in LME mostly belonged to the phyla Proteobacteria (e.g., *Gluconobacter*), Firmicutes (e.g., *Butyrivibrio*) and Actinobacteria (e.g., *Pseudopropionibacterium*), also carrying genes (*n* = 29) identified in their genome and highly correlated to methanogens in LME. For example, genes encoding for multiple carbohydrate degradation like starch and sucrose (K05350 and K05989) were identified in the genome of *Butyrivibrio* or *Gluconobacter* (K02474).

Other clusters showed limited changes between LME and HME networks based on enrichment analysis.

## Discussion

### Functional Niches in the Rumen Microbiome

The novelty of this study lies in the capacity to unprecedently identify 10 microbial functional niches associated with different functions in the rumen. In addition of quantifying their impact on CH_4_ emissions, our results highlight the importance of microbial interaction and their change explaining variability in CH_4_ emissions. Previous studies focused on change in microbial community structure, taxa or genes directly involved in methanogenesis and showing conflicting associations with CH_4_ emissions (Mosoni et al., 2011; Morgavi et al., 2012; Auffret et al., 2018). Moreover, most of these studies did not address inter-domain microbial interactions (Roehe et al., 2016). Identification of functional niches and complex microbial interactions in the rumen microbiome was possible using co-abundance network analysis. This approach was successfully applied for the study of the gut microbiome and other ecosystems (Coutinho et al., 2015; Xiao et al., 2017; Bauer et al., 2018).

### Importance of Methanogens Explaining Differences in CH_4_ Emissions Between HME and LME

When comparing the cluster for methanogenesis between HME and LME, several mechanisms could explain changes in CH_4_ emissions.

As previous studies only focusing on methanogenesis have shown, most of the genes involved in the three methanogenic pathways (hydrogenotrophic, methylotrophic, and acetoclastic) grouped together in the methanogenesis cluster with the most abundant methanogens including the hydrogenotrophic *Methanobrevibacter* (Roehe et al., 2016; Auffret et al., 2018). In this study, one main novelty is that a lower number of hydrogenotrophic methanogenic genera with limited interaction dominated in HME whilst LME animals had more diverse methanogens involved in the three methanogenic pathways and interacting more with other communities. Competition for, e.g., substrates (mainly H_2_) and space (Morgavi et al., 2010) combined with thermodynamics differences for the synthesis of methane (Hydrogenotrophy > Methylotrophy > Acetoclasty; Sprenger et al., 2007; Molenaar et al., 2017) seemed to reduce the importance of *Methanobrevibacter* explaining CH_4_ emissions in LME. For example, the hydrogenotrophic *Methanobacterium* could directly compete for substrates with *Methanobrevibacter*.

Methylotrophic *Methanomassiliicoccales* Candidatus *Methanomethylophilus* known to occupy a different functional niche (cluster 9) than other methanogens (Poulsen et al., 2013) was negatively correlated with CH_4_ emissions. In addition, its relative abundance seemed to be favored (e.g., substrate and thermodynamics) in LME animals (Auffret et al., 2018). Candidatus *Methanomethylophilus* was previously identified as metabolically active in ruminants (Wang et al., 2017; Mann et al., 2018). In the same cluster as Candidatus *Methanomethylophilus* are the acetogens *Eubacterium*, *Blautia*, and *Acetitomaculum*, which are highly active H_2_ sinks as recently shown in sheep (Greening et al., 2019).

Change in relative abundance of most of the methanogens or genes involved in CH_4_ production did not seem important to explain differences between HME and LME. Similar results were previously shown in cattle and sheep (Béra-Maillet et al., 2004; Pandit et al., 2018) and also showed a lack of co-abundances between methanogenic populations, genes and CH_4_ emissions (Zhou et al., 2011; Danielsson et al., 2012; Shi et al., 2014; Wallace et al., 2014; Tapio et al., 2017; Zheng et al., 2018). One explanation for this is the identification of different clades of *Methanobrevibacter* known to differ in their production of CH_4_ (Tapio et al., 2017).

Alternative new CH_4_ synthesis pathway has recently been described in bacteria carrying genes encoding for iron-only nitrogenase (Zheng et al., 2018). Interestingly, Candidatus *Azobacteroides* carrying in its genome the nitrifying gene K02588 had both a strong and positive relationship with CH_4_ emissions. Moreover, this genus was shown to be highly active by metatranscriptomics in the rumen of *Bos indicus* across different dietary treatments confirming its importance in the rumen microbiome (Pandit et al., 2018). Such new result confirmed the diversity of ruminal CH_4_ synthesis pathways and further work on nitrogen-fixing bacteria that also produce CH_4_ is needed to validate these results.

### Importance of the Network Structuring Organic Matter Metabolic Pathways

Contrasting with the general idea, the main variables explaining variability in CH_4_ emissions were bacterial genus (Candidatus *Azobacteroides*) and fungal genus (*Tremella*) or genes involved in specific and limited metabolic pathways (e.g., xylan degradation) but not directly associated with methanogenesis. A more complex microbial network composed of more diverse bacterial and fungal genera and genes were detected in the methanogenesis cluster in LME compared to HME. These results reinforce the hypothesis that CH_4_ production in the rumen is also driven by other microbial communities and their metabolism than methanogens (Vanwonterghem et al., 2017).

In our study, ruminal fiber degraders were identified in different functional niches. For example, *Fibrobacter* (cluster 2) one of the main plant fiber degraders in the rumen (Rychlik and May, 2000), carried genes encoding for xylan degradation (e.g., K01181), and explaining more variability in CH_4_ emissions (49.8%) than other fiber digesting microbes (e.g., fungi or protists). *Ruminococcus*, which is also a well-known bacterial plant fiber degrader in the rumen (Danielsson et al., 2017), was not detected in the network. Such result can be explained by *Fibrobacter* showing fermentation advantages compared to *Ruminococcus* (Danielsson et al., 2017) during diculture experiment with *Methanobrevibacter* and enhancing CH_4_ emissions (Rychlik and May, 2000).

Most of fungi and protist genera were highly correlated with each other (cluster 6), suggesting a close interdependence, in agreement with other studies (Tokura et al., 1997; Miltko et al., 2016). This interaction can be explained by the effect of protist impacting on pH in rumen, enhancing CH_4_ emissions (Eugène et al., 2004; Newbold et al., 2015) as well as their capacity to create anaerobic conditions that favor fungal zoospores development (Ellis et al., 1989). The fungi identified positively correlated with CH_4_ emissions (Aydin et al., 2017) were phylogenetically distant from previously identified fungi within Chytridiomycota (Gruninger et al., 2014). This result and the limited number of genes found associated with fungi or protists can be partly explained by the current limitation when sequencing genome with low GC content (<20%) like in some fungi and protists (Chen et al., 2013) supporting the need for methodological improvement in the study of eukaryotes in the rumen.

### Importance of Clusters Involved in Metabolite Pathways Impacting on CH_4_ Emissions

It is known that microbial metabolites released after the degradation of plant fiber can differently impact CH_4_ emissions (Janssen, 2010; Kamke et al., 2016). For example, *Butyrivibrio* and *Pseudobutyrivibrio* (cluster 4) previously identified as biomarkers of CH_4_ emissions (Auffret et al., 2018) played an important role in the release of substrates (lactate or butyrate) enhancing CH_4_ emissions (Kamke et al., 2016). In contrast, the lactic acid producer *Bifidobacterium* in cluster 7 was negatively correlated (PLS) with CH_4_ emissions (Kamke et al., 2016). Furthermore, *Butyrivibrio* and *Pseudobutyrivibrio* are also formate producers (Tokura et al., 1997; Tapio et al., 2017) as *Fibrobacter*. The importance of formate metabolism associated with changes in CH_4_ emissions need further work (Tapio et al., 2017), especially when the quantity of formate produced seems to be several times greater than H_2_ (Rychlik and May, 2000).

This study refined the importance of Proteobacteria (Tapio et al., 2017), by focusing on γ-Proteobacteria genera (mostly grouping in cluster 9) that showed negative impact on CH_4_ emissions (Wallace et al., 2015; Danielsson et al., 2017). Moreover, some mechanistic explanations for LME involved the presence of some γ-Proteobacteria producing succinate as intermediate of propionate or carrying gene for lactate degradation (K00016), releasing less H_2_ in comparison to other VFA, which may explain a lower synthesis of CH_4_ (Janssen, 2010). In addition, some other γ-Proteobacteria and genes involved in branched chain or aromatic amino acid biosynthesis (shikimate pathway) were previously considered as strong indicator of LME in sheep (Kamke et al., 2016).

Another explanation is the possible impact of H_2_-consuming bacteria like *Selenomonas* in cluster 10 or genes involved in ammonia metabolism; these are all negatively correlated with CH_4_ emissions (PLS) and known to reduce CH_4_ emissions (Olijhoek et al., 2015; Kamke et al., 2016; Sa et al., 2016).

Our study characterizes functional niches in the rumen microbiome by applying network analysis and identifying potential mechanisms having an impact on CH_4_ emissions. Methane emissions variability was mainly explained by variables involved in organic matter degradation pathways, like *Fibrobacter*, or alternative CH_4_ emission pathway. A more complex microbiome involving more interaction between communities or methanogens and involving more metabolic pathways reduced CH_4_ emissions. New CH_4_ mitigation strategies can be developed based on the microbial ecology information obtained in this study, like enhancing populations within γ-Proteobacteria through nutrition intervention, without impacting on animal feed conversion efficiency.

## Data Availability Statement

The datasets generated for this study can be found in the European Nucleotide Archive under accession PRJEB10338 and PRJEB31266.

## Ethics Statement

The animal study was reviewed and approved by the Animal Experiment Committee of SRUC and was conducted in accordance with the requirements of the UK Animals (Scientific Procedures) Act 1986.

## Author Contributions

MA, MM-Á, and RR conceptualized the study and wrote the original draft of the manuscript. MM-Á, MA, BS, and MW worked on the formal analysis. MA, MM-Á, RR, RD, C-AD, JR, RW, BS, TF, RS, and MW reviewed and edited the manuscript. All authors read and approved the final manuscript.

## Conflict of Interest

The authors declare that the research was conducted in the absence of any commercial or financial relationships that could be construed as a potential conflict of interest.

## Footnotes

1 http://www.kegg.jp

2 www.novocraft.com
