# Supplementary material for: Identification of Complex Rumen Microbiome Interaction Within Diverse Functional Niches as Mechanisms Affecting the Variation of Methane Emissions in Bovine
Source: Front Microbiol. 2020 Apr 17;11:659. doi: 10.3389/fmicb.2020.00659 (PMC7181398; doi:10.3389/fmicb.2020.00659)
Supplement: Supplementary file 1 [file Data_Sheet_1.docx]

**Identification of complex rumen microbiome interaction within diverse functional niches as mechanisms affecting the variation of methane emissions in bovine**

Marina Martínez-Álvaro†*^1,2^, Marc D. Auffret†^1€^, Robert D. Stewart^3^, Richard J. Dewhurst^1^, Carol-Anne Duthie^1^, John A. Rooke^1^, R. John Wallace^4^, Barbara Shih^5^, Tom C. Freeman^5^, Mick Watson^3,5^, Rainer Roehe*^1^.

† These authors contributed equally to this work

* Corresponding author

^1^SRUC, Edinburgh, United Kingdom, ^2^Institute for Animal Science and Technology, Universitat Politècnica de València, 46022 Valencia, Spain, ^3^Edinburgh Genomics, The Roslin Institute and R(D)SVS, University of Edinburgh, Edinburgh, United Kingdom, ^4^Rowett Institute, University of Aberdeen, Aberdeen, United Kingdom, ^5^Division of Genetics and Genomics, The Roslin Institute and R(D)SVS, University of Edinburgh, Edinburgh, United Kingdom.

^€^Current address: Danone Nutricia Research, Innovation, Science & Nutrition Department, Gut & Microbiology Laboratories, RD 128 Avenue de la Vauve, 91767 Palaiseau Cedex, France.

**Correspondence:**

[**Marina.Alvaro@sruc.ac.uk**](mailto:Marina.Alvaro@sruc.ac.uk) **; Rainer.Roehe@sruc.ac.uk**

# SUPPLEMENTARY MATERIAL

# Figure S1A. Individual methane emissions for the 63 beef cattle studied

#
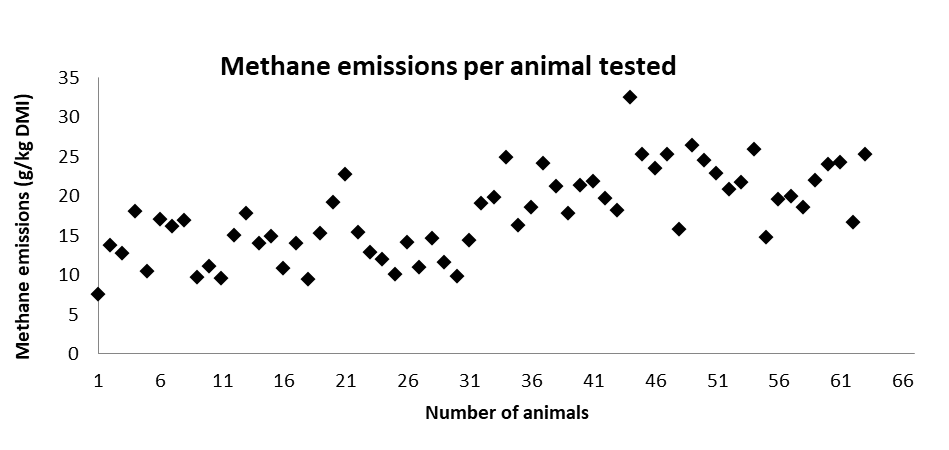


# Figure S1B. Distribution of methane emissions of beef cattle classified by methane emitter groups (HME and LME), diet (FOR and CONC) and breed type.


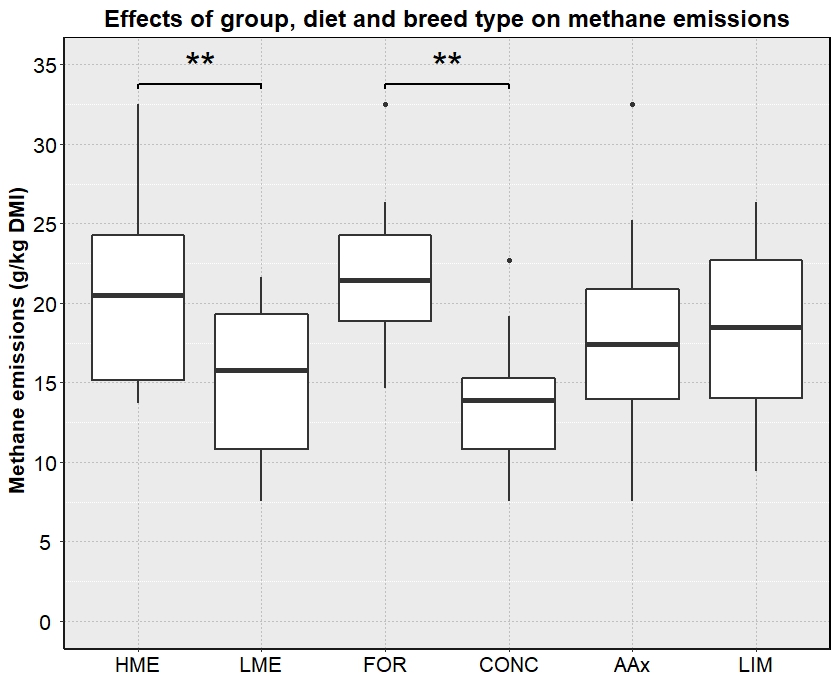


HME = high methane emitters; LME= low methane emitters; FOR = forage based diet; CONC = concentrate based diet; AAx = Aberdeen Angus sired; LIM = Limousin sired; ** = levels significantly different (P-value < 0.001) tested fitting a General Lineal Model including methane emissions as dependent variable and group, diet and breed as independent variables.


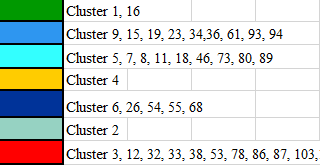
**Figure S2. Functional clusters composed of microbial genera and genes generated from data transformed in log ratio coordinates using SPARCC and then using network analysis in beef cattle.**


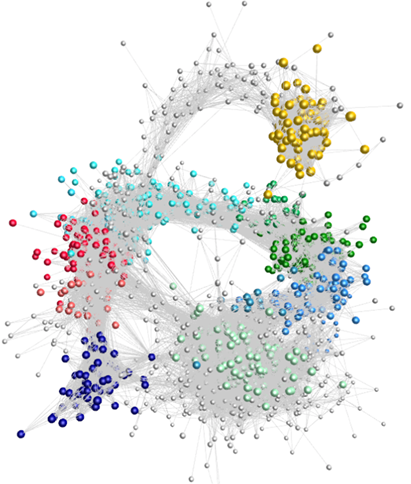


Nodes represent microbial genera and genes, and edges illustrate correlations between their relative abundances. The network was clustered using the MCL algorithm, and only variables with correlation values between nodes greater than 0.70 were kept during the analysis. The colours in the network and the legend indicates the equivalence between the clustering of genera and geness using SPARCC or just their relative abundances (Figure 1, and Table S1A) The clusters are not exactly identical between the two networks, but locations of variables and links between different clusters show that the two networks are similar. Clusters 1 and 16 (in green), closely connected, are composed by the most abundant methanogenic archaea (*Methanobrevibacter, Methanosphaera*), and microbial genes involved in methanogenesis pathway, and also bacteria (*Sarcina*), fungi (*Verrucomicrobium*) and genes in degradation pathways for amino acids and carbohydrates, equivalent to Cluster 1 in the network based on relative abundances, referred to as methanogenic cluster. Clusters 9, 15, 19, 23, 34, 36, 61, 93 and 94 (light blue), all grouping together, contain genus *Fibrobacter* and microbial genes involved in the synthesis of central metabolic enzymes, corresponding to Cluster 2 in the relative abundances network. Clusters 5, 7, 8, 11,18, 46, 73, 80 and 89 (deep turquoise, equivalent to Cluster 3 in the relative abundances network) are composed by microbial genera Proteobacteria, Actinobacteria and Firmicutes interacting with different archaea, some of them methanogen archaea. Cluster 4 (yellow) is dominated by genera of the fungal community, and three hydrogenotrophic and/or acetoclastic methanogens (Cluster 6 in the original network). Clusters 6, 26, 54, 55 and 68 (dark blue) contains the single genus *Bifidobacterium* and microbial genes relevant for carbohydrate degradation, matching with Cluster 7 in the relative abundances network. Cluster 2 (in light turquoise) includes *Prevotella* with genes involved in nitrogen metabolism and pentose phosphate pathway, which were classified as Cluster 8 in the relative abundances network. It also contains other microbial genes involved in carbon cycle, aminoacids metabolism and ribosomal biosynthesis that were out of the 10 niches identified in the analysis with relative abundances. Finally, Clusters 3, 12, 32, 33, 38, 53, 78, 86, 87, 103 and 104 (red), all closely correlated, correspond to relative abundances network Cluster 9 and contained the methylotropic *Methanomassiliicoccales* Candidatus *Methanomethylophilus*, the acetogens *Eubacterium*, *Blautia* and *Acetitomaculum* and a high diversity of Proteobacteria (mainly γ-Proteobacteria) and microbial genes involved in carbohydrates, lipids and aminoacids metabolism. Small clusters identified as 4, 5 and 10 in the relative abundance network were left out of the SPARCC network.

# Table S1A. Composition of the 10 different clusters identified as biological niches in a network analysis including microbial genera and their functional genes considering in the analysis a correlation threshold of 0.70.

| Microbial taxon/gene | Genus/KEGG gene id | Cluster Relative abundance | Cluster SPARCC* | Mean abundance (%) | Coefficient of Variation |
| --- | --- | --- | --- | --- | --- |
| Archaea Euryarchaeota | Candidatus *Halobonum* | 1 | 52 | 0.001 | 49.5 |
| Archaea Euryarchaeota | Candidatus *Methanoplasma* | 1 | No Class | 0.0019 | 78.6 |
| Archaea Euryarchaeota | *Halobellus* | 1 | out of the network | 0.0013 | 45 |
| Archaea Euryarchaeota | *Halogeometricum* | 1 | 16 | 0.0026 | 50 |
| Archaea Euryarchaeota | *Halovivax* | 1 | 16 | 0.0005 | 50.5 |
| Archaea Euryarchaeota | *Methanobacterium* | 1 | No Class | 0.0525 | 38.3 |
| Archaea Euryarchaeota | *Methanobrevibacter* | 1 | 1 | 5.6956 | 72.1 |
| Archaea Euryarchaeota | *Methanosalsum* | 1 | out of the network | 0.0007 | 72.1 |
| Archaea Euryarchaeota | *Methanosarcina* | 1 | 4 | 0.0173 | 30.4 |
| Archaea Euryarchaeota | *Methanosphaera* | 1 | 1 | 0.0298 | 61.4 |
| Archaea Euryarchaeota | *Methanospirillum* | 1 | out of the network | 0.0005 | 67.6 |
| Archaea Euryarchaeota | *Methanothermus* | 1 | 1 | 0.0021 | 65 |
| Archaea Euryarchaeota | *Methanotorris* | 1 | No Class | 0.002 | 63.8 |
| Archaea Euryarchaeota | *Salinarchaeum* | 1 | 16 | 0.0009 | 60.3 |
| Archaea Thaumarchaeota | *Nitrosopumilus* | 1 | out of the network | 0.002 | 41.5 |
| Bacteria Acidobacteria | Candidatus *Solibacter* | 1 | 63 | 0.0118 | 41.5 |
| Bacteria Actinobacteria | *Jonesia* | 1 | 16 | 0.0014 | 66.5 |
| Bacteria Actinobacteria | *Kineococcus* | 1 | 52 | 0.0063 | 44.5 |
| Bacteria Actinobacteria | *Saccharothrix* | 1 | out of the network | 0.0038 | 38.5 |
| Bacteria Actinobacteria | *Segniliparus* | 1 | 11 | 0.0036 | 41 |
| Bacteria Actinobacteria | *Tropheryma* | 1 | out of the network | 0.0003 | 60.8 |
| Bacteria Actinobacteria | *Verrucosispora* | 1 | 11 | 0.0047 | 46.8 |
| Bacteria Bacteroidetes | *Aequorivita* | 1 | out of the network | 0.0026 | 47.4 |
| Bacteria Bacteroidetes | Candidatus *Azobacteroides* | 1 | No Class | 0.0056 | 44.1 |
| Bacteria Bacteroidetes | *Croceibacter* | 1 | No Class | 0.002 | 61.1 |
| Bacteria Bacteroidetes | *Emticicia* | 1 | 16 | 0.0039 | 59.7 |
| Bacteria Bacteroidetes | *Fibrella* | 1 | No Class | 0.0157 | 35.4 |
| Bacteria Bacteroidetes | *Myroides* | 1 | 1 | 0.0139 | 64.4 |
| Bacteria Bacteroidetes | *Pseudopedobacter* | 1 | out of the network | 0.0025 | 59.8 |
| Bacteria Bacteroidetes | *Wenyingzhuangia* | 1 | out of the network | 0.0014 | 55.3 |
| Bacteria Bacteroidetes | *Zunongwangia* | 1 | 95 | 0.0021 | 62 |
| Bacteria Chlorobi | *Prosthecochloris* | 1 | No Class | 0.0061 | 37.8 |
| Bacteria Chloroflexi | *Anaerolinea* | 1 | 16 | 0.0045 | 54.1 |
| Bacteria Chloroflexi | *Roseiflexus* | 1 | No Class | 0.0111 | 40.2 |
| Bacteria Cyanobacteria | *Cyanothece* | 1 | No Class | 0.0126 | 49.1 |
| Bacteria Cyanobacteria | *Fischerella* | 1 | out of the network | 0.0034 | 59.2 |
| Bacteria Cyanobacteria | *Halothece* | 1 | out of the network | 0.0005 | 64.6 |
| Bacteria Cyanobacteria | *Microcystis* | 1 | No Class | 0.0076 | 77 |
| Bacteria Cyanobacteria | *Planktothrix* | 1 | out of the network | 0.0027 | 53.2 |
| Bacteria Deferribacteres | *Denitrovibrio* | 1 | No Class | 0.0018 | 55.4 |
| Bacteria Elusimicrobia | *Endomicrobium* | 1 | No Class | 0.0058 | 62.3 |
| Bacteria Firmicutes | *Acetohalobium* | 1 | 46 | 0.0024 | 43.1 |
| Bacteria Firmicutes | Candidatus *Desulforudis* | 1 | 11 | 0.0075 | 39.6 |
| Bacteria Firmicutes | *Coprothermobacter* | 1 | No Class | 0.0006 | 57.9 |
| Bacteria Firmicutes | *Dehalobacter* | 1 | 52 | 0.0133 | 45 |
| Bacteria Firmicutes | *Flavonifractor* | 1 | 46 | 0.3304 | 42.2 |
| Bacteria Firmicutes | *Intestinimonas* | 1 | 46 | 0.1614 | 42.6 |
| Bacteria Firmicutes | *Limnochorda* | 1 | 11 | 0.0188 | 40.5 |
| Bacteria Firmicutes | *Parageobacillus* | 1 | 52 | 0.0019 | 50.4 |
| Bacteria Firmicutes | *Sarcina* | 1 | 1 | 2.7031 | 87.8 |
| Bacteria Firmicutes | *Syntrophomonas* | 1 | 1 | 0.0046 | 52.8 |
| Bacteria Ignavibacteriae | *Ignavibacterium* | 1 | out of the network | 0.0042 | 75.7 |
| Bacteria Kiritimatiellaeota | *Kiritimatiella* | 1 | 63 | 0.0163 | 57.6 |
| Bacteria Planctomycetes | *Isosphaera* | 1 | 16 | 0.0035 | 55.8 |
| Bacteria Planctomycetes | *Paludisphaera* | 1 | 16 | 0.0106 | 51.2 |
| Bacteria Planctomycetes | *Pirellula* | 1 | No Class | 0.004 | 47.4 |
| Bacteria Planctomycetes | *Planctomyces* | 1 | 63 | 0.0232 | 45 |
| Bacteria Planctomycetes | *Planctopirus* | 1 | No Class | 0.0023 | 51 |
| Bacteria Planctomycetes | *Rhodopirellula* | 1 | 16 | 0.003 | 54.6 |
| Bacteria Planctomycetes | *Rubinisphaera* | 1 | No Class | 0.0052 | 54 |
| Bacteria Planctomycetes | *Singulisphaera* | 1 | 16 | 0.0059 | 56.6 |
| Bacteria α-Proteobacteria | *Asaia* | 1 | 11 | 0.0025 | 46.3 |
| Bacteria α-Proteobacteria | *Magnetospira* | 1 | No Class | 0.0043 | 43.5 |
| Bacteria α-Proteobacteria | *Marinovum* | 1 | 11 | 0.005 | 38.8 |
| Bacteria α-Proteobacteria | *Oligotropha* | 1 | 16 | 0.0029 | 52.7 |
| Bacteria α-Proteobacteria | *Parvularcula* | 1 | 16 | 0.0019 | 52.2 |
| Bacteria α-Proteobacteria | *Phenylobacterium* | 1 | 52 | 0.0076 | 44.4 |
| Bacteria α-Proteobacteria | *Planktomarina* | 1 | 16 | 0.0008 | 52.7 |
| Bacteria β-Proteobacteria | Candidatus *Symbiobacter* | 1 | 11 | 0.0031 | 42.7 |
| Bacteria β-Proteobacteria | *Methylobacillus* | 1 | 11 | 0.0026 | 41.8 |
| Bacteria δ-Proteobacteria | Candidatus *Desulfofervidus* | 1 | No Class | 0.0009 | 74.5 |
| Bacteria δ-Proteobacteria | *Chondromyces* | 1 | 16 | 0.0077 | 48 |
| Bacteria δ-Proteobacteria | *Desulfobacter* | 1 | out of the network | 0.0033 | 42 |
| Bacteria δ-Proteobacteria | *Desulfohalobium* | 1 | 11 | 0.0053 | 37.6 |
| Bacteria δ-Proteobacteria | *Desulfurivibrio* | 1 | 11 | 0.0072 | 42.3 |
| Bacteria γ-Proteobacteria | *Endozoicomonas* | 1 | 16 | 0.0025 | 45.1 |
| Bacteria γ-Proteobacteria | *Fluoribacter* | 1 | out of the network | 0.0006 | 53.5 |
| Bacteria γ-Proteobacteria | *Saccharophagus* | 1 | No Class | 0.0016 | 52 |
| Bacteria Spirochaetes | *Turneriella* | 1 | out of the network | 0.0038 | 56 |
| Bacteria Thermodesulfobacteria | *Thermodesulfatator* | 1 | 16 | 0.0015 | 65.1 |
| Bacteria Verrucomicrobia | Candidatus *Xiphinematobacter* | 1 | 16 | 0.0006 | 78 |
| Bacteria Verrucomicrobia | *Methylacidiphilum* | 1 | No Class | 0.0025 | 66.6 |
| Bacteria Verrucomicrobia | *Opitutus* | 1 | 16 | 0.0152 | 48.1 |
| Bacteria Verrucomicrobia | *Verrucomicrobium* | 1 | 16 | 0.0161 | 39.8 |
| Fungi Ascomycota | *Capronia* | 1 | No Class | 0.0068 | 46 |
| Fungi Ascomycota | *Lachancea* | 1 | 9 | 0.0005 | 66.1 |
| Fungi Ascomycota | *Verticillium* | 1 | out of the network | 0.0068 | 37 |
| Fungi Basidiomycota | *Anthracocystis* | 1 | 11 | 0.0042 | 38.3 |
| Fungi Basidiomycota | *Auricularia* | 1 | No Class | 0.0023 | 43.9 |
| Fungi Basidiomycota | *Coniophora* | 1 | No Class | 0.0012 | 47.5 |
| Fungi Basidiomycota | *Coprinopsis* | 1 | out of the network | 0.0016 | 40.5 |
| Fungi Basidiomycota | *Dichomitus* | 1 | 16 | 0.0014 | 53.1 |
| Fungi Basidiomycota | *Punctularia* | 1 | out of the network | 0.0017 | 33.2 |
| Fungi Basidiomycota | *Rhodotorula* | 1 | No Class | 0.0044 | 43.2 |
| Fungi Basidiomycota | *Trametes* | 1 | 16 | 0.0011 | 55.3 |
| Fungi Basidiomycota | *Tremella* | 1 | No Class | 0.0102 | 61.3 |
| Fungi Basidiomycota | *Ustilago* | 1 | out of the network | 0.0039 | 49.2 |
| Protist Stramenopiles | *Saprolegnia* | 1 | 4 | 0.0074 | 35 |
| Hydroxymethylglutaryl-CoA reductase (NADPH) [EC:1.1.1.34] | K00021 | 1 | 1 | 0.0026 | 93.8 |
| Gluconate 5-dehydrogenase [EC:1.1.1.69] | K00046 | 1 | No Class | 0.0901 | 35.2 |
| Ketol-acid reductoisomerase [EC:1.1.1.86] | K00053 | 1 | 2 | 0.2767 | 13 |
| Dihydroflavonol-4-reductase [EC:1.1.1.219] | K00091 | 1 | out of the network | 0.0066 | 60.7 |
| Formate dehydrogenase, alpha subunit [EC:1.2.1.2] | K00123 | 1 | 1 | 0.1734 | 55.6 |
| Formate dehydrogenase, beta subunit [EC:1.2.1.2] | K00125 | 1 | 1 | 0.0717 | 61.2 |
| Aspartate-semialdehyde dehydrogenase [EC:1.2.1.11] | K00133 | 1 | 2 | 0.1281 | 22.2 |
| Glyceraldehyde-3-phosphate dehydrogenase (NAD(P)) [EC:1.2.1.59] | K00150 | 1 | 1 | 0.0444 | 77.8 |
| Pyruvate ferredoxin oxidoreductase, alpha subunit [EC:1.2.7.1] | K00169 | 1 | 1 | 0.0477 | 68.1 |
| Pyruvate ferredoxin oxidoreductase, beta subunit [EC:1.2.7.1] | K00170 | 1 | 1 | 0.0343 | 69.3 |
| Pyruvate ferredoxin oxidoreductase, delta subunit [EC:1.2.7.1] | K00171 | 1 | 1 | 0.0074 | 78 |
| Pyruvate ferredoxin oxidoreductase, gamma subunit [EC:1.2.7.1] | K00172 | 1 | 1 | 0.0204 | 71.2 |
| 2-oxoisovalerate ferredoxin oxidoreductase, alpha subunit [EC:1.2.7.7] | K00186 | 1 | 1 | 0.0117 | 62.5 |
| 2-oxoisovalerate ferredoxin oxidoreductase, beta subunit [EC:1.2.7.7] | K00187 | 1 | 1 | 0.0169 | 72.9 |
| 2-oxoisovalerate ferredoxin oxidoreductase, delta subunit [EC:1.2.7.7] | K00188 | 1 | 1 | 0.0033 | 101.8 |
| Carbon-monoxide dehydrogenase iron sulfur subunit | K00196 | 1 | 1 | 0.0097 | 93.9 |
| Formylmethanofuran dehydrogenase subunit A [EC:1.2.99.5] | K00200 | 1 | 1 | 0.0923 | 69.8 |
| Formylmethanofuran dehydrogenase subunit B [EC:1.2.99.5] | K00201 | 1 | 1 | 0.1235 | 59.6 |
| Formylmethanofuran dehydrogenase subunit C [EC:1.2.99.5] | K00202 | 1 | 1 | 0.0559 | 59 |
| Formylmethanofuran dehydrogenase subunit D [EC:1.2.99.5] | K00203 | 1 | 1 | 0.0253 | 68.8 |
| Formylmethanofuran dehydrogenase subunit H [EC:1.2.99.5] | K00204 | 1 | 1 | 0.0098 | 84.9 |
| Formylmethanofuran dehydrogenase subunit F [EC:1.2.99.5] | K00205 | 1 | 1 | 0.0213 | 90.2 |
| Dihydrodipicolinate reductase [EC:1.3.1.26] | K00215 | 1 | 21 | 0.0662 | 27.6 |
| Fumarate reductase iron-sulfur protein [EC:1.3.99.1] | K00245 | 1 | 1 | 0.0061 | 91.6 |
| Methylenetetrahydromethanopterin dehydrogenase [EC:1.5.99.9] | K00319 | 1 | 1 | 0.0927 | 53.4 |
| Coenzyme F420-dependent N5,N10-methenyltetrahydromethanopterin reductase [EC:1.5.99.11] | K00320 | 1 | 1 | 0.1 | 51.1 |
| Methyl-coenzyme M reductase alpha subunit [EC:2.8.4.1] | K00399 | 1 | 1 | 0.1825 | 59.9 |
| Methyl coenzyme M reductase system, component A2 | K00400 | 1 | 1 | 0.0382 | 77.3 |
| Methyl-coenzyme M reductase beta subunit [EC:2.8.4.1] | K00401 | 1 | 1 | 0.1152 | 52.8 |
| Methyl-coenzyme M reductase gamma subunit [EC:2.8.4.1] | K00402 | 1 | 1 | 0.0664 | 53 |
| Coenzyme F420 hydrogenase alpha subunit [EC:1.12.98.1] | K00440 | 1 | 1 | 0.0525 | 64.3 |
| Coenzyme F420 hydrogenase beta subunit [EC:1.12.98.1] | K00441 | 1 | 1 | 0.0358 | 75.9 |
| Coenzyme F420 hydrogenase delta subunit | K00442 | 1 | 1 | 0.0036 | 96.7 |
| Coenzyme F420 hydrogenase gamma subunit [EC:1.12.98.1] | K00443 | 1 | 1 | 0.0327 | 71.8 |
| Ribonucleoside-triphosphate reductase [EC:1.17.4.2] | K00527 | 1 | 46 | 0.5996 | 24.8 |
| Tetrahydromethanopterin S-methyltransferase subunit A [EC:2.1.1.86] | K00577 | 1 | 1 | 0.0458 | 73.3 |
| Tetrahydromethanopterin S-methyltransferase subunit B [EC:2.1.1.86] | K00578 | 1 | 66 | 0.0055 | 75.9 |
| Tetrahydromethanopterin S-methyltransferase subunit C [EC:2.1.1.86] | K00579 | 1 | 1 | 0.0138 | 89.9 |
| Tetrahydromethanopterin S-methyltransferase subunit D [EC:2.1.1.86] | K00580 | 1 | 1 | 0.0343 | 70.6 |
| Tetrahydromethanopterin S-methyltransferase subunit E [EC:2.1.1.86] | K00581 | 1 | 1 | 0.0765 | 61.2 |
| Tetrahydromethanopterin S-methyltransferase subunit G [EC:2.1.1.86] | K00583 | 1 | 1 | 0.0065 | 64.4 |
| Tetrahydromethanopterin S-methyltransferase subunit H [EC:2.1.1.86] | K00584 | 1 | 1 | 0.08 | 70.2 |
| Acetyl-CoA C-acetyltransferase [EC:2.3.1.9] | K00626 | 1 | 40 | 0.0695 | 45.8 |
| Chloramphenicol O-acetyltransferase [EC:2.3.1.28] | K00638 | 1 | 1 | 0.1107 | 54.8 |
| Glycine C-acetyltransferase [EC:2.3.1.29] | K00639 | 1 | 1 | 0.0143 | 62 |
| Maltose O-acetyltransferase [EC:2.3.1.79] | K00661 | 1 | out of the network | 0.0075 | 82.3 |
| Formylmethanofuran--tetrahydromethanopterin N-formyltransferase [EC:2.3.1.101] | K00672 | 1 | 1 | 0.0442 | 79.3 |
| Orotate phosphoribosyltransferase [EC:2.4.2.10] | K00762 | 1 | No Class | 0.044 | 40.6 |
| Riboflavin synthase beta chain [EC:2.5.1.-] | K00794 | 1 | No Class | 0.0308 | 53.5 |
| Deoxyhypusine synthase [EC:2.5.1.46] | K00809 | 1 | out of the network | 0.0015 | 76.6 |
| Nucleoside-diphosphate kinase [EC:2.7.4.6] | K00940 | 1 | 1 | 0.0309 | 59.3 |
| UTP--glucose-1-phosphate uridylyltransferase [EC:2.7.7.9] | K00963 | 1 | No Class | 0.0143 | 56.8 |
| Glycerol-3-phosphate cytidylyltransferase [EC:2.7.7.39] | K00980 | 1 | 66 | 0.0086 | 67.4 |
| Pyruvate, water dikinase [EC:2.7.9.2] | K01007 | 1 | 1 | 0.0897 | 54.7 |
| Phosphoserine phosphatase [EC:3.1.3.3] | K01079 | 1 | 1 | 0.1076 | 51.7 |
| Adenosylhomocysteinase [EC:3.3.1.1] | K01251 | 1 | 1 | 0.0845 | 67.5 |
| Phosphoribosyl-AMP cyclohydrolase [EC:3.5.4.19] | K01496 | 1 | No Class | 0.0112 | 74.4 |
| Methenyltetrahydromethanopterin cyclohydrolase [EC:3.5.4.27] | K01499 | 1 | 1 | 0.0665 | 68.1 |
| Nucleoside-triphosphatase [EC:3.6.1.15] | K01516 | 1 | 1 | 0.0445 | 53.1 |
| Arsenite-transporting ATPase [EC:3.6.3.16] | K01551 | 1 | 1 | 0.0105 | 82 |
| Fructose 1,6-bisphosphate aldolase/phosphatase [EC:4.1.2.13 3.1.3.11] | K01622 | 1 | 1 | 0.0871 | 58.2 |
| Fructose-bisphosphate aldolase, class I [EC:4.1.2.13] | K01623 | 1 | 1 | 0.0116 | 59.4 |
| N-acetylneuraminate synthase [EC:2.5.1.56] | K01654 | 1 | 1 | 0.0035 | 86.7 |
| Carbonic anhydrase [EC:4.2.1.1] | K01673 | 1 | 1 | 0.0133 | 74.1 |
| Fumarate hydratase subunit alpha [EC:4.2.1.2] | K01677 | 1 | 40 | 0.0155 | 60.7 |
| Threonine synthase [EC:4.2.3.1] | K01733 | 1 | out of the network | 0.0978 | 32.7 |
| 6-pyruvoyl tetrahydrobiopterin synthase [EC:4.2.3.12] | K01737 | 1 | 1 | 0.0069 | 71.1 |
| Diaminopimelate epimerase [EC:5.1.1.7] | K01778 | 1 | out of the network | 0.025 | 44.9 |
| Myo-inositol-1-phosphate synthase [EC:5.5.1.4] | K01858 | 1 | out of the network | 0.055 | 35.4 |
| Aspartyl-tRNA synthetase [EC:6.1.1.12] | K01876 | 1 | 90 | 0.2609 | 17.6 |
| Succinyl-CoA synthetase alpha subunit [EC:6.2.1.5] | K01902 | 1 | 1 | 0.0077 | 60.6 |
| Succinyl-CoA synthetase beta subunit [EC:6.2.1.5] | K01903 | 1 | 1 | 0.0178 | 73.6 |
| NAD+ synthase [EC:6.3.1.5] | K01916 | 1 | 1 | 0.0059 | 79 |
| Phosphoribosylamine--glycine ligase [EC:6.3.4.13] | K01945 | 1 | No Class | 0.0943 | 35.6 |
| GMP synthase (glutamine-hydrolysing) [EC:6.3.5.2] | K01951 | 1 | 90 | 0.1479 | 16.7 |
| Pyruvate carboxylase subunit A [EC:6.4.1.1] | K01959 | 1 | 1 | 0.0437 | 64.6 |
| SRP RNA | K01983 | 1 | 1 | 0.0117 | 86.3 |
| Cobalt/nickel transport system permease protein | K02007 | 1 | 1 | 0.052 | 72.4 |
| Cobalt transport protein | K02009 | 1 | 1 | 0.007 | 75.9 |
| Molybdate transport system permease protein | K02018 | 1 | 1 | 0.018 | 80.4 |
| Molybdate transport system regulatory protein | K02019 | 1 | 1 | 0.0018 | 91.9 |
| Molybdate transport system substrate-binding protein | K02020 | 1 | 40 | 0.0187 | 52.3 |
| Peptide/nickel transport system ATP-binding protein | K02031 | 1 | 9 | 0.0378 | 60.3 |
| V-type H+-transporting ATPase subunit A [EC:3.6.3.14] | K02117 | 1 | 1 | 0.1784 | 55.4 |
| V-type H+-transporting ATPase subunit B [EC:3.6.3.14] | K02118 | 1 | 1 | 0.1587 | 54.8 |
| V-type H+-transporting ATPase subunit C [EC:3.6.3.14] | K02119 | 1 | 1 | 0.0286 | 68.3 |
| V-type H+-transporting ATPase subunit D [EC:3.6.3.14] | K02120 | 1 | 1 | 0.0424 | 65 |
| V-type H+-transporting ATPase subunit E [EC:3.6.3.14] | K02121 | 1 | 1 | 0.0271 | 58.8 |
| V-type H+-transporting ATPase subunit F [EC:3.6.3.14] | K02122 | 1 | 1 | 0.0118 | 56.9 |
| V-type H+-transporting ATPase subunit I [EC:3.6.3.14] | K02123 | 1 | 1 | 0.0538 | 63.6 |
| V-type H+-transporting ATPase subunit K [EC:3.6.3.14] | K02124 | 1 | 1 | 0.066 | 52.5 |
| Ferritin [EC:1.16.3.1] | K02217 | 1 | 1 | 0.0208 | 70.9 |
| Adenosylcobyric acid synthase [EC:6.3.5.10] | K02232 | 1 | No Class | 0.0274 | 60.8 |
| Uroporphyrin-III C-methyltransferase [EC:2.1.1.107] | K02303 | 1 | 1 | 0.0085 | 71 |
| DNA polymerase I [EC:2.7.7.7] | K02319 | 1 | 1 | 0.0108 | 84.2 |
| DNA polymerase II large subunit [EC:2.7.7.7] | K02322 | 1 | 1 | 0.028 | 70.9 |
| DNA polymerase II small subunit [EC:2.7.7.7] | K02323 | 1 | 1 | 0.0117 | 67.2 |
| FdhD protein | K02379 | 1 | 1 | 0.0024 | 92.9 |
| Ribosomal RNA large subunit methyltransferase E [EC:2.1.1.-] | K02427 | 1 | 66 | 0.0046 | 73.3 |
| UDP-N-acetyl-D-mannosaminuronic acid dehydrogenase [EC:1.1.1.-] | K02472 | 1 | 1 | 0.0049 | 86.4 |
| Glutamyl-tRNA reductase [EC:1.2.1.70] | K02492 | 1 | 1 | 0.0154 | 75.8 |
| Nitrogen fixation protein NifB | K02585 | 1 | 1 | 0.0179 | 72.6 |
| Transcriptional antiterminator NusG | K02601 | 1 | out of the network | 0.0459 | 36.2 |
| Uracil permease | K02824 | 1 | out of the network | 0.0465 | 38.7 |
| 3,4-dihydroxy 2-butanone 4-phosphate synthase [EC:4.1.99.12] | K02858 | 1 | 1 | 0.0047 | 100.4 |
| Large subunit ribosomal protein L10e | K02866 | 1 | 1 | 0.033 | 54.3 |
| Large subunit ribosomal protein L12 | K02869 | 1 | 1 | 0.0275 | 63.2 |
| Large subunit ribosomal protein L14e | K02875 | 1 | 1 | 0.0102 | 58.7 |
| Large subunit ribosomal protein L15e | K02877 | 1 | 1 | 0.0472 | 68.5 |
| Large subunit ribosomal protein L18e | K02883 | 1 | 1 | 0.0098 | 75.4 |
| Large subunit ribosomal protein L19e | K02885 | 1 | 1 | 0.032 | 64.1 |
| Large subunit ribosomal protein L21e | K02889 | 1 | 1 | 0.0131 | 77.2 |
| Large subunit ribosomal protein L24e | K02896 | 1 | 1 | 0.0087 | 59.1 |
| Large subunit ribosomal protein L29 | K02904 | 1 | No Class | 0.0386 | 34.4 |
| Large subunit ribosomal protein L30e | K02908 | 1 | 1 | 0.0206 | 61 |
| Large subunit ribosomal protein L31e | K02910 | 1 | 1 | 0.0136 | 58.3 |
| Large subunit ribosomal protein L32e | K02912 | 1 | 1 | 0.0205 | 76.9 |
| Large subunit ribosomal protein L34e | K02915 | 1 | 1 | 0.0174 | 61 |
| Large subunit ribosomal protein L37Ae | K02921 | 1 | 1 | 0.0171 | 65.8 |
| Large subunit ribosomal protein L37e | K02922 | 1 | 1 | 0.0104 | 52.8 |
| Large subunit ribosomal protein L39e | K02924 | 1 | 1 | 0.0159 | 64 |
| Large subunit ribosomal protein L40e | K02927 | 1 | 1 | 0.0126 | 59.1 |
| Large subunit ribosomal protein L44e | K02929 | 1 | 1 | 0.0303 | 63 |
| Large subunit ribosomal protein L4e | K02930 | 1 | 1 | 0.0385 | 71.4 |
| Large subunit ribosomal protein L7Ae | K02936 | 1 | 1 | 0.0337 | 53.9 |
| Large subunit ribosomal protein LX | K02944 | 1 | 1 | 0.0036 | 80.3 |
| Small subunit ribosomal protein S15 | K02956 | 1 | No Class | 0.0665 | 30.3 |
| Small subunit ribosomal protein S17e | K02962 | 1 | 1 | 0.0089 | 62.6 |
| Small subunit ribosomal protein S19e | K02966 | 1 | 1 | 0.0384 | 49.7 |
| Small subunit ribosomal protein S24e | K02974 | 1 | 1 | 0.0095 | 75.6 |
| Small subunit ribosomal protein S27Ae | K02977 | 1 | 66 | 0.0047 | 57.9 |
| Small subunit ribosomal protein S27e | K02978 | 1 | 1 | 0.0129 | 48.1 |
| Small subunit ribosomal protein S28e | K02979 | 1 | 1 | 0.0148 | 56.7 |
| Small subunit ribosomal protein S3Ae | K02984 | 1 | 1 | 0.0389 | 61.3 |
| Small subunit ribosomal protein S4e | K02987 | 1 | 1 | 0.0205 | 69.8 |
| Small subunit ribosomal protein S6e | K02991 | 1 | 1 | 0.0195 | 53.7 |
| Small subunit ribosomal protein S8e | K02995 | 1 | 1 | 0.0178 | 64.7 |
| Small subunit ribosomal protein S9 | K02996 | 1 | 76 | 0.1114 | 18.9 |
| DNA-directed RNA polymerase subunit Aprime [EC:2.7.7.6] | K03041 | 1 | 1 | 0.0649 | 70.4 |
| DNA-directed RNA polymerase subunit Aprime [EC:2.7.7.6] | K03042 | 1 | 1 | 0.0393 | 79.5 |
| DNA-directed RNA polymerase subunit Bprime [EC:2.7.7.6] | K03044 | 1 | 1 | 0.086 | 65 |
| DNA-directed RNA polymerase subunit Bprime [EC:2.7.7.6] | K03045 | 1 | 1 | 0.0543 | 69.5 |
| DNA-directed RNA polymerase subunit D [EC:2.7.7.6] | K03047 | 1 | 1 | 0.0196 | 63.9 |
| DNA-directed RNA polymerase subunit Eprime [EC:2.7.7.6] | K03049 | 1 | 1 | 0.0107 | 68 |
| DNA-directed RNA polymerase subunit K [EC:2.7.7.6] | K03055 | 1 | 1 | 0.0033 | 85.9 |
| DNA-directed RNA polymerase subunit L [EC:2.7.7.6] | K03056 | 1 | 66 | 0.004 | 70.6 |
| DNA-directed RNA polymerase subunit N [EC:2.7.7.6] | K03058 | 1 | 1 | 0.0104 | 60.2 |
| DNA-directed RNA polymerase subunit P [EC:2.7.7.6] | K03059 | 1 | 1 | 0.0031 | 90.4 |
| Preprotein translocase subunit SecD | K03072 | 1 | No Class | 0.0101 | 60.1 |
| Preprotein translocase subunit SecF | K03074 | 1 | No Class | 0.0078 | 64.7 |
| Translation initiation factor eIF-1 | K03113 | 1 | 1 | 0.0196 | 61.7 |
| Transcription initiation factor TFIID TATA-box-binding protein | K03120 | 1 | 1 | 0.0489 | 77 |
| Transcription initiation factor TFIIB | K03124 | 1 | 1 | 0.048 | 70.1 |
| Transcription initiation factor TFIIE alpha subunit | K03136 | 1 | 1 | 0.0048 | 85.4 |
| Thiamine biosynthesis protein ThiC | K03147 | 1 | out of the network | 0.143 | 36.8 |
| DNA topoisomerase VI subunit A [EC:5.99.1.3] | K03166 | 1 | 1 | 0.0442 | 63.2 |
| DNA topoisomerase VI subunit B [EC:5.99.1.3] | K03167 | 1 | 1 | 0.03 | 64 |
| tRNA pseudouridine synthase B [EC:5.4.99.12] | K03177 | 1 | No Class | 0.0203 | 52.4 |
| Elongation factor EF-1 alpha subunit [EC:3.6.5.3] | K03231 | 1 | 1 | 0.1208 | 52.4 |
| Elongation factor EF-1 beta subunit | K03232 | 1 | 1 | 0.0082 | 99.4 |
| Elongation factor EF-2 [EC:3.6.5.3] | K03234 | 1 | 1 | 0.0706 | 91.1 |
| Translation initiation factor eIF-1A | K03236 | 1 | 1 | 0.0199 | 67.4 |
| Translation initiation factor eIF-2 alpha subunit | K03237 | 1 | 1 | 0.0411 | 61.7 |
| Translation initiation factor eIF-2 beta subunit | K03238 | 1 | 1 | 0.025 | 60.9 |
| Translation initiation factor eIF-2 gamma subunit | K03242 | 1 | 1 | 0.0426 | 66.8 |
| Translation initiation factor eIF-5B | K03243 | 1 | 1 | 0.0154 | 79.9 |
| Translation initiation factor eIF-5A | K03263 | 1 | 1 | 0.0264 | 64.9 |
| Translation initiation factor eIF-6 | K03264 | 1 | 1 | 0.0057 | 107.5 |
| Peptide chain release factor eRF subunit 1 | K03265 | 1 | 1 | 0.0143 | 73.2 |
| Glutamyl-tRNA(Gln) amidotransferase subunit E [EC:6.3.5.7] | K03330 | 1 | No Class | 0.0201 | 53.6 |
| Heterodisulfide reductase subunit A [EC:1.8.98.1] | K03388 | 1 | 1 | 0.2083 | 55.9 |
| Heterodisulfide reductase subunit B [EC:1.8.98.1] | K03389 | 1 | 1 | 0.0624 | 51.8 |
| Heterodisulfide reductase subunit C [EC:1.8.98.1] | K03390 | 1 | 1 | 0.0336 | 58.7 |
| Proteasome regulatory subunit | K03420 | 1 | 1 | 0.0419 | 69.8 |
| Methyl-coenzyme M reductase subunit C | K03421 | 1 | 1 | 0.0188 | 66.9 |
| Methyl-coenzyme M reductase subunit D | K03422 | 1 | 1 | 0.0073 | 93.9 |
| Proteasome alpha subunit [EC:3.4.25.1] | K03432 | 1 | 1 | 0.0291 | 83.2 |
| Proteasome beta subunit [EC:3.4.25.1] | K03433 | 1 | 1 | 0.0333 | 71.7 |
| Ribonuclease P protein subunit POP4 [EC:3.1.26.5] | K03538 | 1 | 1 | 0.0036 | 82.3 |
| Ribonuclease P protein subunit RPR2 [EC:3.1.26.5] | K03540 | 1 | 1 | 0.0085 | 61.5 |
| Holliday junction resolvase, archaea type | K03552 | 1 | 1 | 0.0067 | 72.9 |
| Molybdopterin synthase catalytic subunit [EC:2.-.-.-] | K03635 | 1 | 1 | 0.0047 | 100.8 |
| Molybdenum cofactor biosynthesis protein | K03639 | 1 | 40 | 0.0154 | 86 |
| Exosome complex component RRP4 | K03679 | 1 | 1 | 0.0385 | 73 |
| DtxR family transcriptional regulator, Mn-dependent transcriptional regulator | K03709 | 1 | 1 | 0.0032 | 79.7 |
| Archaea-specific helicase [EC:3.6.1.-] | K03725 | 1 | 1 | 0.0038 | 81.5 |
| Helicase [EC:3.6.4.-] | K03726 | 1 | 1 | 0.0032 | 68.6 |
| Aldehyde:ferredoxin oxidoreductase [EC:1.2.7.5] | K03738 | 1 | 1 | 0.0142 | 55.2 |
| Molybdopterin biosynthesis protein MoeA | K03750 | 1 | 1 | 0.0084 | 84.3 |
| Molybdopterin-guanine dinucleotide biosynthesis protein A | K03752 | 1 | 1 | 0.0025 | 91.1 |
| Molybdopterin-guanine dinucleotide biosynthesis protein B | K03753 | 1 | 1 | 0.0054 | 98.5 |
| Peptidyl-prolyl cis-trans isomerase B (cyclophilin B) [EC:5.2.1.8] | K03768 | 1 | 1 | 0.0378 | 43.8 |
| FKBP-type peptidyl-prolyl cis-trans isomerase SlyD [EC:5.2.1.8] | K03775 | 1 | No Class | 0.0145 | 49.6 |
| Sirohydrochlorin cobaltochelatase [EC:4.99.1.3] | K03795 | 1 | 1 | 0.0026 | 74 |
| NADH dehydrogenase [EC:1.6.99.3] | K03885 | 1 | 1 | 0.0109 | 87 |
| Lon-like ATP-dependent protease [EC:3.4.21.-] | K04076 | 1 | 1 | 0.0543 | 63.6 |
| Chorismate mutase [EC:5.4.99.5] | K04093 | 1 | 1 | 0.005 | 104.1 |
| DNA repair protein RadA | K04483 | 1 | 1 | 0.0407 | 75.6 |
| Nitrogen fixation protein NifU and related proteins | K04488 | 1 | No Class | 0.0084 | 67.5 |
| Lysyl-tRNA synthetase, class I [EC:6.1.1.6] | K04566 | 1 | 1 | 0.0391 | 73.7 |
| Hydrogenase nickel incorporation protein HypB | K04652 | 1 | 1 | 0.0074 | 72.2 |
| Hydrogenase expression/formation protein HypC | K04653 | 1 | 1 | 0.0087 | 71.9 |
| Hydrogenase expression/formation protein HypD | K04654 | 1 | 1 | 0.0136 | 58.6 |
| Hydrogenase expression/formation protein HypE | K04655 | 1 | 1 | 0.0148 | 59.4 |
| Hydrogenase maturation protein HypF | K04656 | 1 | 1 | 0.0124 | 76.4 |
| Peptidyl-tRNA hydrolase, PTH2 family [EC:3.1.1.29] | K04794 | 1 | 1 | 0.0085 | 99.2 |
| Fibrillarin-like pre-rRNA processing protein | K04795 | 1 | 1 | 0.0031 | 79.4 |
| Small nuclear ribonucleoprotein | K04796 | 1 | 1 | 0.0174 | 53.3 |
| Prefoldin alpha subunit | K04797 | 1 | 1 | 0.01 | 74.4 |
| Prefoldin beta subunit | K04798 | 1 | 1 | 0.0065 | 85.8 |
| Replication factor C small subunit | K04801 | 1 | 1 | 0.0108 | 89.6 |
| Proliferating cell nuclear antigen | K04802 | 1 | 1 | 0.0327 | 67.6 |
| (R)-2-hydroxyacid dehydrogenase [EC:1.1.1.272] | K05884 | 1 | 1 | 0.0092 | 68.6 |
| Precorrin-6X reductase [EC:1.3.1.54] | K05895 | 1 | 1 | 0.0041 | 100.3 |
| Precorrin-3B C17-methyltransferase [EC:2.1.1.131] | K05934 | 1 | No Class | 0.0146 | 51.4 |
| Precorrin-4 C11-methyltransferase [EC:2.1.1.133] | K05936 | 1 | 40 | 0.0066 | 65.5 |
| Precorrin-8X methylmutase [EC:5.4.1.2] | K06042 | 1 | 1 | 0.0197 | 67.9 |
| ATP-binding cassette, sub-family E, member 1 | K06174 | 1 | 1 | 0.0336 | 77.1 |
| tRNA pseudouridine synthase D [EC:5.4.99.12] | K06176 | 1 | 1 | 0.0139 | 93.9 |
| 5-formaminoimidazole-4-carboxamide-1-(beta)-D-ribofuranosyl 5prime-monophosphate synthetase [EC:6.3.4.-] | K06863 | 1 | 1 | 0.0467 | 63.3 |
| ATPase | K06865 | 1 | 1 | 0.0049 | 89.4 |
| Programmed cell death protein 5 | K06875 | 1 | 1 | 0.0065 | 80.3 |
| Nicotinic acid adenine dinucleotide carboxylase/hydrolase | K06898 | 1 | out of the network | 0.0023 | 72.9 |
| Uncharacterized protein | K06915 | 1 | 41 | 0.0471 | 33.2 |
| Queuosine biosynthesis protein QueC | K06920 | 1 | No Class | 0.0133 | 52.8 |
| tRNA(Ile2)-agmatinylcytidine synthase [EC:6.3.4.22] | K06932 | 1 | 1 | 0.0208 | 73.8 |
| 7,8-dihydro-6-hydroxymethylpterin dimethyltransferase [EC:2.1.1.-] | K06937 | 1 | 1 | 0.0137 | 82.1 |
| Nucleolar GTP-binding protein | K06943 | 1 | 1 | 0.0056 | 82.1 |
| Uncharacterized protein | K06944 | 1 | 1 | 0.0201 | 81 |
| Ribosomal RNA assembly protein | K06961 | 1 | 1 | 0.0135 | 82.7 |
| Beta-ribofuranosylaminobenzene 5'-phosphate synthase [EC:2.4.2.54] | K06984 | 1 | 1 | 0.0076 | 99.7 |
| Uncharacterized protein | K06988 | 1 | 1 | 0.0212 | 89.3 |
| Uncharacterized protein | K07013 | 1 | 1 | 0.01 | 86.4 |
| No such data | K07021 | 1 | 1 | 0.0331 | 70.6 |
| Uncharacterized protein | K07041 | 1 | 1 | 0.0412 | 72.6 |
| Uncharacterized protein | K07045 | 1 | No Class | 0.0111 | 84.9 |
| Uncharacterized protein | K07068 | 1 | 1 | 0.0182 | 56.3 |
| (4-(4-[2-(gamma-L-glutamylamino)ethyl]phenoxymethyl)furan-2-yl)methanamine synthase [EC:2.5.1.131] | K07072 | 1 | 1 | 0.0022 | 96 |
| Putative glycerol-1-phosphate prenyltransferase [EC:2.5.1.-] | K07094 | 1 | 1 | 0.0119 | 82.9 |
| Uncharacterized protein | K07096 | 1 | 1 | 0.0072 | 91.3 |
| Uncharacterized protein | K07108 | 1 | 1 | 0.0079 | 89.5 |
| Uncharacterized protein | K07135 | 1 | 1 | 0.0045 | 98.8 |
| 5-(aminomethyl)-3-furanmethanol phosphate kinase [EC:2.7.4.31] | K07144 | 1 | 1 | 0.013 | 86.2 |
| Dolichyl-diphosphooligosaccharide--protein glycosyltransferase [EC:2.4.1.119] | K07151 | 1 | 1 | 0.0096 | 94.7 |
| Uncharacterized protein | K07158 | 1 | 1 | 0.0037 | 97 |
| Uncharacterized protein | K07159 | 1 | 1 | 0.0082 | 94.8 |
| Uncharacterized protein | K07161 | 1 | 1 | 0.017 | 81.4 |
| Hypothetical protein | K07254 | 1 | 1 | 0.0109 | 92.5 |
| Anaerobic dimethyl sulfoxide reductase subunit A [EC:1.8.99.-] | K07306 | 1 | out of the network | 0.003 | 58.2 |
| Hydrogenase expression/formation protein | K07388 | 1 | 1 | 0.026 | 68.5 |
| AAA family ATPase | K07392 | 1 | 1 | 0.0031 | 80.1 |
| Putative methyltransferase | K07446 | 1 | 1 | 0.004 | 121.1 |
| Archaea-specific RecJ-like exonuclease | K07463 | 1 | 1 | 0.0072 | 83 |
| Putative transposase | K07491 | 1 | No Class | 0.0124 | 48.1 |
| Diphthamide synthase subunit DPH2 | K07561 | 1 | 1 | 0.0033 | 106.2 |
| RNA-binding protein | K07569 | 1 | 1 | 0.002 | 88.6 |
| Putative nucleotide binding protein | K07572 | 1 | 1 | 0.0055 | 103.7 |
| Exosome complex component CSL4 | K07573 | 1 | 1 | 0.0088 | 86 |
| Putative RNA-binding protein containing KH domain | K07574 | 1 | 1 | 0.009 | 66 |
| Hypothetical protein | K07580 | 1 | 1 | 0.0117 | 53.2 |
| Hypothetical protein | K07582 | 1 | 1 | 0.0171 | 50.8 |
| Hypothetical protein | K07585 | 1 | 1 | 0.0111 | 74.7 |
| ArsR family transcriptional regulator | K07721 | 1 | 1 | 0.0116 | 84.5 |
| CopG family transcriptional regulator, nickel-responsive regulator | K07722 | 1 | 1 | 0.006 | 80.2 |
| Putative transcriptional regulator | K07728 | 1 | 1 | 0.0105 | 94.5 |
| Putative transcriptional regulator | K07730 | 1 | 1 | 0.0072 | 94.6 |
| Elongator complex protein 3 [EC:2.3.1.48] | K07739 | 1 | 1 | 0.0085 | 82 |
| Hypothetical protein | K07744 | 1 | 1 | 0.0027 | 96.5 |
| 6-phospho-3-hexuloisomerase [EC:5.3.1.27] | K08094 | 1 | 1 | 0.0072 | 87.9 |
| GTP cyclohydrolase IIa [EC:3.5.4.29] | K08096 | 1 | 1 | 0.0194 | 76.2 |
| Methylthioribose-1-phosphate isomerase [EC:5.3.1.23] | K08963 | 1 | 1 | 0.0097 | 68.5 |
| Hypothetical protein | K09003 | 1 | 1 | 0.0037 | 107.4 |
| Hypothetical protein | K09007 | 1 | 1 | 0.0176 | 63 |
| Hypothetical protein | K09116 | 1 | 1 | 0.0023 | 103.6 |
| Hypothetical protein | K09123 | 1 | 1 | 0.0158 | 73 |
| Hypothetical protein | K09128 | 1 | 1 | 0.0048 | 111.4 |
| Hypothetical protein | K09136 | 1 | 1 | 0.0037 | 103.7 |
| Hypothetical protein | K09140 | 1 | 1 | 0.0026 | 94.6 |
| Hypothetical protein | K09142 | 1 | 1 | 0.0372 | 70.1 |
| Hypothetical protein | K09154 | 1 | 1 | 0.0473 | 93.9 |
| Hypothetical protein | K09181 | 1 | No Class | 0.0677 | 63.6 |
| Glutamyl-tRNA(Gln) amidotransferase subunit D [EC:6.3.5.7] | K09482 | 1 | 1 | 0.0131 | 82.2 |
| Hypothetical protein | K09717 | 1 | 1 | 0.0078 | 94.9 |
| Hypothetical protein | K09720 | 1 | 1 | 0.004 | 94.9 |
| Hypothetical protein | K09726 | 1 | 1 | 0.0149 | 77 |
| Hypothetical protein | K09728 | 1 | 1 | 0.0063 | 96.5 |
| Hypothetical protein | K09733 | 1 | 1 | 0.0253 | 73.4 |
| Hypothetical protein | K09735 | 1 | 1 | 0.0082 | 76.8 |
| LL-diaminopimelate aminotransferase [EC:2.6.1.83] | K10206 | 1 | No Class | 0.0393 | 40.5 |
| Archaeal cell division control protein 6 | K10725 | 1 | 1 | 0.0088 | 81.1 |
| Replicative DNA helicase Mcm [EC:3.6.4.-] | K10726 | 1 | 1 | 0.0366 | 58.9 |
| Fanconi anemia group M protein [EC:3.6.4.13] | K10896 | 1 | 1 | 0.0026 | 90.9 |
| Trans-homoaconitate synthase [EC:4.1.3.-] | K10977 | 1 | 1 | 0.0071 | 88.9 |
| IMP cyclohydrolase [EC:3.5.4.10] | K11176 | 1 | 1 | 0.0138 | 67.2 |
| Formylmethanofuran dehydrogenase subunit G [EC:1.2.99.5] | K11260 | 1 | 1 | 0.0045 | 73.1 |
| Formylmethanofuran dehydrogenase subunit E [EC:1.2.99.5] | K11261 | 1 | 1 | 0.0083 | 100.9 |
| Exosome complex component RRP41 | K11600 | 1 | 1 | 0.0298 | 62.4 |
| Dehydroquinate synthase II [EC:1.4.1.-] | K11646 | 1 | 1 | 0.0067 | 83.3 |
| FO synthase subunit 1 [EC:2.5.1.-] | K11780 | 1 | 1 | 0.0125 | 94.5 |
| FO synthase subunit 2 [EC:2.5.1.-] | K11781 | 1 | 1 | 0.0045 | 86.7 |
| Exosome complex component RRP42 | K12589 | 1 | 1 | 0.0211 | 75.6 |
| Transitional endoplasmic reticulum ATPase | K13525 | 1 | 1 | 0.0588 | 67.4 |
| Geranylgeranyl diphosphate synthase, type I [EC:2.5.1.1 2.5.1.10 2.5.1.29] | K13787 | 1 | 1 | 0.0059 | 58.6 |
| Bifunctional enzyme Fae/Hps [EC:4.3.-.- 4.1.2.43] | K13812 | 1 | 1 | 0.0491 | 73.2 |
| 5,10-methenyltetrahydromethanopterin hydrogenase [EC:1.12.98.2] | K13942 | 1 | 1 | 0.0878 | 68.4 |
| Alcohol dehydrogenase, propanol-preferring [EC:1.1.1.1] | K13953 | 1 | 1 | 0.0169 | 113.7 |
| Energy-converting hydrogenase A subunit C | K14094 | 1 | 1 | 0.0027 | 88.2 |
| Energy-converting hydrogenase A subunit E | K14096 | 1 | 1 | 0.0024 | 83.5 |
| Energy-converting hydrogenase A subunit G | K14098 | 1 | 1 | 0.0025 | 93.3 |
| Energy-converting hydrogenase A subunit H | K14099 | 1 | 1 | 0.0041 | 95 |
| Energy-converting hydrogenase A subunit J | K14101 | 1 | 1 | 0.0117 | 82.1 |
| Energy-converting hydrogenase A subunit M | K14104 | 1 | 1 | 0.007 | 91.4 |
| Energy-converting hydrogenase A subunit N | K14105 | 1 | 1 | 0.0181 | 73.4 |
| Energy-converting hydrogenase A subunit O | K14106 | 1 | 1 | 0.0178 | 83.8 |
| Energy-converting hydrogenase A subunit P | K14107 | 1 | 1 | 0.0031 | 97.4 |
| Energy-converting hydrogenase A subunit Q | K14108 | 1 | 1 | 0.0072 | 88.5 |
| Energy-converting hydrogenase A subunit R | K14109 | 1 | 1 | 0.0041 | 88.2 |
| Energy-converting hydrogenase B subunit A | K14110 | 1 | 1 | 0.0056 | 98 |
| Energy-converting hydrogenase B subunit F | K14115 | 1 | 1 | 0.0112 | 85 |
| Energy-converting hydrogenase B subunit K | K14120 | 1 | 1 | 0.0088 | 94.9 |
| Energy-converting hydrogenase B subunit L | K14121 | 1 | 1 | 0.0046 | 108.2 |
| Energy-converting hydrogenase B subunit M | K14122 | 1 | 1 | 0.0125 | 77.6 |
| Energy-converting hydrogenase B subunit N | K14123 | 1 | 1 | 0.0161 | 83 |
| Energy-converting hydrogenase B subunit O | K14124 | 1 | out of the network | 0.0021 | 87.6 |
| F420-non-reducing hydrogenase subunit A [EC:1.12.99.-] | K14126 | 1 | 1 | 0.1033 | 54.8 |
| F420-non-reducing hydrogenase iron-sulfur subunit D [EC:1.12.99.-] | K14127 | 1 | 1 | 0.0589 | 54.8 |
| F420-non-reducing hydrogenase subunit G [EC:1.12.99.-] | K14128 | 1 | 1 | 0.0623 | 59.1 |
| tRNA His | K14226 | 1 | out of the network | 0.0029 | 49.2 |
| tRNA Met | K14230 | 1 | 2 | 0.035 | 21.2 |
| Bacteria Fibrobacteres | *Fibrobacter* | 2 | 9 | 1.7391 | 114.7 |
| Alcohol dehydrogenase [EC:1.1.1.1] | K00001 | 2 | 94 | 0.005 | 103.4 |
| UDPglucose 6-dehydrogenase [EC:1.1.1.22] | K00012 | 2 | out of the network | 0.0754 | 34.1 |
| Malate dehydrogenase (oxaloacetate-decarboxylating)(NADP+) [EC:1.1.1.40] | K00029 | 2 | 9 | 0.0324 | 106.4 |
| Isocitrate dehydrogenase [EC:1.1.1.42] | K00031 | 2 | 39 | 0.059 | 46.2 |
| 3-isopropylmalate dehydrogenase [EC:1.1.1.85] | K00052 | 2 | 22 | 0.0525 | 38.7 |
| dTDP-4-dehydrorhamnose reductase [EC:1.1.1.133] | K00067 | 2 | 93 | 0.0165 | 72.5 |
| 2-dehydropantoate 2-reductase [EC:1.1.1.169] | K00077 | 2 | 47 | 0.0109 | 83.3 |
| IMP dehydrogenase [EC:1.1.1.205] | K00088 | 2 | out of the network | 0.1601 | 18 |
| Aldehyde dehydrogenase (NAD+) [EC:1.2.1.3] | K00128 | 2 | 9 | 0.007 | 98.8 |
| Glutamate-5-semialdehyde dehydrogenase [EC:1.2.1.41] | K00147 | 2 | 15 | 0.0302 | 50.1 |
| Acyl-ACP dehydrogenase [EC:1.3.99.-] | K00257 | 2 | 9 | 0.0348 | 92.6 |
| NADH dehydrogenase I subunit B [EC:1.6.5.3] | K00331 | 2 | 9 | 0.0081 | 109.8 |
| NADH dehydrogenase I subunit D [EC:1.6.5.3] | K00333 | 2 | 9 | 0.0256 | 112.2 |
| NADH dehydrogenase I subunit F [EC:1.6.5.3] | K00335 | 2 | 9 | 0.0324 | 65.2 |
| NADH dehydrogenase I subunit G [EC:1.6.5.3] | K00336 | 2 | 23 | 0.0237 | 78 |
| NADH dehydrogenase I subunit N [EC:1.6.5.3] | K00343 | 2 | 36 | 0.0345 | 66.4 |
| Adenylylsulfate reductase, subunit A [EC:1.8.99.2] | K00394 | 2 | 19 | 0.0152 | 81 |
| 5-methyltetrahydrofolate--homocysteine methyltransferase [EC:2.1.1.13] | K00548 | 2 | No Class | 0.0622 | 69.9 |
| 5-methyltetrahydropteroyltriglutamate--homocysteine methyltransferase [EC:2.1.1.14] | K00549 | 2 | 34 | 0.0403 | 67.8 |
| tRNA-specific 2-thiouridylase [EC:2.8.1.-] | K00566 | 2 | No Class | 0.0235 | 69.2 |
| Ornithine carbamoyltransferase [EC:2.1.3.3] | K00611 | 2 | 39 | 0.0321 | 44.1 |
| Glutamate N-acetyltransferase / amino-acid N-acetyltransferase [EC:2.3.1.35 2.3.1.1] | K00620 | 2 | 34 | 0.0184 | 72.9 |
| Homoserine O-acetyltransferase [EC:2.3.1.31] | K00641 | 2 | 9 | 0.0136 | 104.3 |
| Dolichol-phosphate mannosyltransferase [EC:2.4.1.83] | K00721 | 2 | 36 | 0.02 | 71.7 |
| Nicotinate-nucleotide pyrophosphorylase (carboxylating) [EC:2.4.2.19] | K00767 | 2 | 17 | 0.0263 | 39 |
| Histidinol-phosphate aminotransferase [EC:2.6.1.9] | K00817 | 2 | 15 | 0.0455 | 46.8 |
| Adenosylmethionine-8-amino-7-oxononanoate aminotransferase [EC:2.6.1.62] | K00833 | 2 | 23 | 0.0113 | 85.4 |
| dTMP kinase [EC:2.7.4.9] | K00943 | 2 | No Class | 0.0054 | 80.3 |
| Cytidylate kinase [EC:2.7.4.14] | K00945 | 2 | No Class | 0.02 | 45 |
| Sulfate adenylyltransferase subunit 1 [EC:2.7.7.4] | K00956 | 2 | 23 | 0.0169 | 92.3 |
| Sulfate adenylyltransferase subunit 2 [EC:2.7.7.4] | K00957 | 2 | 23 | 0.0125 | 75.4 |
| UDP-N-acetylglucosamine pyrophosphorylase [EC:2.7.7.23] | K00972 | 2 | 19 | 0.0107 | 98.5 |
| Glucose-1-phosphate thymidylyltransferase [EC:2.7.7.24] | K00973 | 2 | out of the network | 0.0312 | 34.2 |
| Ribonuclease PH [EC:2.7.7.56] | K00989 | 2 | 61 | 0.0085 | 73.9 |
| CDP-diacylglycerol--glycerol-3-phosphate 3-phosphatidyltransferase [EC:2.7.8.5] | K00995 | 2 | 34 | 0.0119 | 69.6 |
| Biotin synthetase [EC:2.8.1.6] | K01012 | 2 | 19 | 0.0306 | 65.5 |
| dGTPase [EC:3.1.5.1] | K01129 | 2 | 15 | 0.0249 | 58.2 |
| Crossover junction endodeoxyribonuclease RuvC [EC:3.1.22.4] | K01159 | 2 | 42 | 0.0083 | 51.8 |
| Endoglucanase [EC:3.2.1.4] | K01179 | 2 | 9 | 0.0523 | 89.2 |
| Endo-1,4-beta-xylanase [EC:3.2.1.8] | K01181 | 2 | 9 | 0.0194 | 101.2 |
| Beta-galactosidase [EC:3.2.1.23] | K01190 | 2 | No Class | 0.1179 | 32.9 |
| Beta-N-acetylhexosaminidase [EC:3.2.1.52] | K01207 | 2 | 94 | 0.0118 | 75.2 |
| Arabinogalactan endo-1,4-beta-galactosidase [EC:3.2.1.89] | K01224 | 2 | No Class | 0.0127 | 82.7 |
| Leucyl aminopeptidase [EC:3.4.11.1] | K01255 | 2 | 9 | 0.0056 | 106.4 |
| dCMP deaminase [EC:3.5.4.12] | K01493 | 2 | No Class | 0.0115 | 41.2 |
| Phosphoribosyl-ATP pyrophosphohydrolase [EC:3.6.1.31] | K01523 | 2 | 34 | 0.0051 | 64.8 |
| Phosphoenolpyruvate carboxykinase (GTP) [EC:4.1.1.32] | K01596 | 2 | 9 | 0.0385 | 98.1 |
| Acetolactate synthase I/II/III large subunit [EC:2.2.1.6] | K01652 | 2 | 97 | 0.106 | 32.1 |
| Anthranilate synthase component I [EC:4.1.3.27] | K01657 | 2 | No Class | 0.0561 | 47.7 |
| 4-hydroxy 2-oxovalerate aldolase [EC:4.1.3.39] | K01666 | 2 | 23 | 0.0152 | 93.5 |
| Dihydroxy-acid dehydratase [EC:4.2.1.9] | K01687 | 2 | 28 | 0.132 | 21.7 |
| Enolase [EC:4.2.1.11] | K01689 | 2 | out of the network | 0.1027 | 40.9 |
| Tryptophan synthase beta chain [EC:4.2.1.20] | K01696 | 2 | out of the network | 0.0881 | 24.2 |
| Porphobilinogen synthase [EC:4.2.1.24] | K01698 | 2 | 9 | 0.0079 | 85.4 |
| 3-isopropylmalate/(R)-2-methylmalate dehydratase large subunit [EC:4.2.1.33 4.2.1.35] | K01703 | 2 | 34 | 0.0895 | 32.6 |
| 3-isopropylmalate/(R)-2-methylmalate dehydratase small subunit [EC:4.2.1.33 4.2.1.35] | K01704 | 2 | 34 | 0.0282 | 51.1 |
| O-acetylhomoserine (thiol)-lyase [EC:2.5.1.49] | K01740 | 2 | 100 | 0.215 | 22.7 |
| Histidine ammonia-lyase [EC:4.3.1.3] | K01745 | 2 | 19 | 0.0066 | 103.9 |
| Cystathionine beta-lyase [EC:4.4.1.8] | K01760 | 2 | 47 | 0.0089 | 84.6 |
| dTDP-4-dehydrorhamnose 3,5-epimerase [EC:5.1.3.13] | K01790 | 2 | out of the network | 0.0046 | 54.4 |
| Glutamate-1-semialdehyde 2,1-aminomutase [EC:5.4.3.8] | K01845 | 2 | 9 | 0.0131 | 86.8 |
| Carbamoyl-phosphate synthase large subunit [EC:6.3.5.5] | K01955 | 2 | 90 | 0.5213 | 22 |
| HlyD family secretion protein | K01993 | 2 | 36 | 0.0233 | 59.8 |
| Peptide/nickel transport system ATP-binding protein | K02032 | 2 | 85 | 0.028 | 46.9 |
| Peptide/nickel transport system substrate-binding protein | K02035 | 2 | 34 | 0.0854 | 46.3 |
| Sulfate transport system substrate-binding protein | K02048 | 2 | 23 | 0.0104 | 92.8 |
| Sulfonate/nitrate/taurine transport system ATP-binding protein | K02049 | 2 | 9 | 0.0216 | 88 |
| Sulfonate/nitrate/taurine transport system permease protein | K02050 | 2 | 34 | 0.0226 | 64 |
| Putative ABC transport system ATP-binding protein | K02065 | 2 | 93 | 0.0163 | 70.2 |
| Putative ABC transport system permease protein | K02066 | 2 | No Class | 0.0235 | 95.8 |
| Phosphoserine / homoserine phosphotransferase [EC:3.1.3.3 2.7.1.39] | K02203 | 2 | 23 | 0.0098 | 86.7 |
| Competence protein ComEC | K02238 | 2 | 94 | 0.0155 | 91.8 |
| Precorrin-2 dehydrogenase / sirohydrochlorin ferrochelatase [EC:1.3.1.76 4.99.1.4] | K02304 | 2 | 23 | 0.0051 | 88.5 |
| 3R-hydroxymyristoyl ACP dehydrase [EC:4.2.1.-] | K02372 | 2 | 23 | 0.0081 | 92.3 |
| Aspartyl-tRNA(Asn)/glutamyl-tRNA (Gln) amidotransferase subunit B [EC:6.3.5.6 6.3.5.7] | K02434 | 2 | 60 | 0.0957 | 39.2 |
| General secretion pathway protein E | K02454 | 2 | 19 | 0.0057 | 89 |
| Two-component system, LytT family, response regulator | K02477 | 2 | out of the network | 0.002 | 78.2 |
| Lipid A biosynthesis lauroyl acyltransferase [EC:2.3.1.-] | K02517 | 2 | 10 | 0.0102 | 80.6 |
| Octaprenyl diphosphate synthase [EC:2.5.1.-] | K02523 | 2 | 36 | 0.0068 | 90.1 |
| Nitrogenase molybdenum-iron protein alpha chain [EC:1.18.6.1] | K02586 | 2 | 9 | 0.0264 | 109.8 |
| Nitrogenase iron protein NifH [EC:1.18.6.1] | K02588 | 2 | 9 | 0.0137 | 67.4 |
| Nitrogenase molybdenum-iron protein beta chain [EC:1.18.6.1] | K02591 | 2 | 9 | 0.0216 | 111.8 |
| Type IV pilus assembly protein PilB | K02652 | 2 | 9 | 0.022 | 81 |
| Twitching motility protein PilT | K02669 | 2 | 23 | 0.0259 | 80.7 |
| Ribosomal protein L11 methyltransferase [EC:2.1.1.-] | K02687 | 2 | 10 | 0.0124 | 74.1 |
| Pyrimidine operon attenuation protein / uracil phosphoribosyltransferase [EC:2.4.2.9] | K02825 | 2 | 23 | 0.0033 | 87.6 |
| RNA polymerase sigma-54 factor | K03092 | 2 | 25 | 0.0198 | 61.8 |
| Signal peptidase II [EC:3.4.23.36] | K03101 | 2 | 15 | 0.007 | 65.2 |
| DNA topoisomerase III [EC:5.99.1.2] | K03169 | 2 | 29 | 0.0556 | 44.3 |
| Hydrophobic/amphiphilic exporter-1 (mainly G- bacteria), HAE1 family | K03296 | 2 | 23 | 0.0481 | 99 |
| Na+:H+ antiporter, NhaA family | K03313 | 2 | 61 | 0.0096 | 93 |
| Sulfate permease, SulP family | K03321 | 2 | 29 | 0.0408 | 44 |
| tRNA (guanine-N7-)-methyltransferase [EC:2.1.1.33] | K03439 | 2 | 15 | 0.0111 | 59.9 |
| Chromosome segregation protein | K03529 | 2 | 85 | 0.046 | 67.9 |
| Biopolymer transport protein ExbD | K03559 | 2 | 23 | 0.0109 | 102.1 |
| TldD protein | K03568 | 2 | 9 | 0.0115 | 98.5 |
| Rod shape-determining protein MreC | K03570 | 2 | 25 | 0.0076 | 74.8 |
| 7,8-dihydro-8-oxoguanine triphosphatase [EC:3.6.1.-] | K03574 | 2 | 9 | 0.0098 | 65.7 |
| Exodeoxyribonuclease V alpha subunit [EC:3.1.11.5] | K03581 | 2 | 19 | 0.0237 | 98.8 |
| ATP-binding protein involved in chromosome partitioning | K03593 | 2 | 15 | 0.019 | 61.1 |
| ATP-dependent DNA helicase RecQ [EC:3.6.4.12] | K03654 | 2 | 15 | 0.059 | 52.7 |
| Ribonuclease D [EC:3.1.13.5] | K03684 | 2 | 61 | 0.0078 | 104.4 |
| ATP-dependent DNA helicase DinG [EC:3.6.4.12] | K03722 | 2 | 9 | 0.0166 | 121.7 |
| Peptidyl-prolyl cis-trans isomerase D [EC:5.2.1.8] | K03770 | 2 | 36 | 0.0133 | 101.1 |
| Peptidyl-prolyl cis-trans isomerase SurA [EC:5.2.1.8] | K03771 | 2 | 29 | 0.0143 | 53.7 |
| 5prime-nucleotidase [EC:3.1.3.5] | K03787 | 2 | No Class | 0.0102 | 68.2 |
| MoxR-like ATPase [EC:3.6.3.-] | K03924 | 2 | 100 | 0.069 | 33.5 |
| Putative transcription regulator | K03976 | 2 | 19 | 0.005 | 94.3 |
| Virulence factor | K03980 | 2 | 9 | 0.0139 | 108 |
| Glucose inhibited division protein Gid | K04094 | 2 | 9 | 0.0139 | 78.9 |
| Prephenate dehydrogenase [EC:1.3.1.12] | K04517 | 2 | No Class | 0.0123 | 81.2 |
| Ferrous iron transport protein B | K04759 | 2 | No Class | 0.1463 | 29.1 |
| ATP-dependent RNA helicase DeaD [EC:3.6.4.13] | K05592 | 2 | 25 | 0.0694 | 45.9 |
| Dipeptidase E [EC:3.4.13.21] | K05995 | 2 | 9 | 0.004 | 106 |
| HPr kinase/phosphorylase [EC:2.7.11.- 2.7.4.-] | K06023 | 2 | 19 | 0.0069 | 87.8 |
| Segregation and condensation protein B | K06024 | 2 | 19 | 0.0049 | 89.5 |
| Undecaprenyl-diphosphatase [EC:3.6.1.27] | K06153 | 2 | 34 | 0.0176 | 58.7 |
| Bifunctional enzyme involved in thiolation and methylation of tRNA | K06168 | 2 | 25 | 0.0295 | 45.1 |
| Ribosomal large subunit pseudouridine synthase C [EC:5.4.99.12] | K06179 | 2 | 19 | 0.0085 | 95.4 |
| 7,8-dihydropterin-6-yl-methyl-4-(beta-D-ribofuranosyl)aminobenzene 5'-Phosphate synthase [EC:2.5.1.105] | K06897 | 2 | 36 | 0.0058 | 92.6 |
| Uncharacterized protein | K06959 | 2 | 23 | 0.0525 | 65.6 |
| Pyrimidine/purine-5'-nucleotide nucleosidase [EC:3.2.2.10 3.2.2.-] | K06966 | 2 | No Class | 0.0114 | 49.5 |
| PLP dependent protein | K06997 | 2 | 15 | 0.0115 | 63.6 |
| Uncharacterized protein | K07003 | 2 | 23 | 0.04 | 102.2 |
| Glycosyltransferase 2 family protein | K07027 | 2 | 93 | 0.0113 | 68.1 |
| Uncharacterized protein | K07037 | 2 | 36 | 0.0158 | 74.6 |
| Probable rRNA maturation factor | K07042 | 2 | 47 | 0.0057 | 95.9 |
| 3',5'-nucleoside bisphosphate phosphatase [EC:3.1.3.97] | K07053 | 2 | 19 | 0.0062 | 93.5 |
| UPF0755 protein | K07082 | 2 | 15 | 0.0114 | 66.3 |
| Flotillin | K07192 | 2 | 9 | 0.0132 | 92.3 |
| D-alanyl-D-alanine carboxypeptidase [EC:3.4.16.4] | K07260 | 2 | 9 | 0.0034 | 124.2 |
| Two-component system, OmpR family, response regulator VicR | K07668 | 2 | 23 | 0.0036 | 96.6 |
| Two-component system, NtrC family, nitrogen regulation response regulator GlnG | K07712 | 2 | 9 | 0.0081 | 121.2 |
| Soluble lytic murein transglycosylase [EC:3.2.1.-] | K08309 | 2 | 23 | 0.0096 | 116.2 |
| D-alanyl-D-alanine dipeptidase [EC:3.4.13.-] | K08641 | 2 | 94 | 0.0065 | 64.6 |
| Hypothetical protein | K09702 | 2 | 9 | 0.0025 | 102.5 |
| Lipoprotein-releasing system permease protein | K09808 | 2 | 36 | 0.0187 | 65.7 |
| Hypothetical protein | K09952 | 2 | out of the network | 0.0059 | 145.5 |
| DNA excision repair protein ERCC-3 [EC:3.6.4.12] | K10843 | 2 | 9 | 0.0176 | 108.1 |
| Aminotransferase [EC:2.6.1.-] | K10907 | 2 | 23 | 0.0095 | 92.4 |
| Cysteine desulfurase / selenocysteine lyase [EC:2.8.1.7 4.4.1.16] | K11717 | 2 | No Class | 0.0742 | 39.9 |
| ATP-dependent RNA helicase RhlE [EC:3.6.4.13] | K11927 | 2 | 9 | 0.0091 | 109.5 |
| Ribonuclease R [EC:3.1.-.-] | K12573 | 2 | 25 | 0.052 | 45.7 |
| Anthranilate synthase/phosphoribosyltransferase [EC:4.1.3.27 2.4.2.18] | K13497 | 2 | 9 | 0.0276 | 106.2 |
| Phosphate acetyltransferase [EC:2.3.1.8] | K13788 | 2 | 61 | 0.0121 | 103.2 |
| Ribosomal protein S12 methylthiotransferase [EC:2.-.-.-] | K14441 | 2 | 25 | 0.0204 | 59.8 |
| Algae Stramenopiles | *Aureococcus* | 3 | 63 | 0.0141 | 42.7 |
| Archaea Crenarchaeota | *Pyrodictium* | 3 | out of the network | 0.0003 | 49.8 |
| Archaea Crenarchaeota | *Thermoproteus* | 3 | out of the network | 0.0004 | 61.5 |
| Archaea Euryarchaeota | *Archaeoglobus* | 3 | 16 | 0.0007 | 63.3 |
| Archaea Euryarchaeota | *Haladaptatus* | 3 | out of the network | 0.0031 | 38.6 |
| Archaea Euryarchaeota | *Halalkalicoccus* | 3 | No Class | 0.0012 | 56 |
| Archaea Euryarchaeota | *Haloarcula* | 3 | 5 | 0.0054 | 36.4 |
| Archaea Euryarchaeota | *Halobacterium* | 3 | No Class | 0.004 | 41.1 |
| Archaea Euryarchaeota | *Halobaculum* | 3 | out of the network | 0.0007 | 48.2 |
| Archaea Euryarchaeota | *Halobiforma* | 3 | 11 | 0.002 | 51.3 |
| Archaea Euryarchaeota | *Haloferax* | 3 | 7 | 0.0084 | 35.3 |
| Archaea Euryarchaeota | *Halopenitus* | 3 | 8 | 0.0013 | 44.1 |
| Archaea Euryarchaeota | *Halopiger* | 3 | 5 | 0.0035 | 38.7 |
| Archaea Euryarchaeota | *Haloprofundus* | 3 | out of the network | 0.0007 | 62.8 |
| Archaea Euryarchaeota | *Halorubrum* | 3 | 5 | 0.011 | 32.3 |
| Archaea Euryarchaeota | *Haloterrigena* | 3 | No Class | 0.0055 | 33 |
| Archaea Euryarchaeota | *Methanocella* | 3 | 5 | 0.0031 | 41.4 |
| Archaea Euryarchaeota | *Methanocorpusculum* | 3 | No Class | 0.0036 | 35.7 |
| Archaea Euryarchaeota | *Methanoculleus* | 3 | 5 | 0.0112 | 32.2 |
| Archaea Euryarchaeota | *Methanofollis* | 3 | No Class | 0.0059 | 32.9 |
| Archaea Euryarchaeota | *Methanogenium* | 3 | 11 | 0.001 | 53 |
| Archaea Euryarchaeota | *Methanolacinia* | 3 | out of the network | 0.0012 | 52.5 |
| Archaea Euryarchaeota | *Methanolinea* | 3 | out of the network | 0.0008 | 46.9 |
| Archaea Euryarchaeota | *Methanomassiliicoccus* | 3 | 7 | 0.005 | 38.4 |
| Archaea Euryarchaeota | *Methanosaeta* | 3 | 5 | 0.0038 | 37 |
| Archaea Euryarchaeota | *Methanosphaerula* | 3 | No Class | 0.0009 | 45.8 |
| Archaea Euryarchaeota | *Natronobacterium* | 3 | out of the network | 0.0004 | 63 |
| Archaea Euryarchaeota | *Thermococcus* | 3 | 8 | 0.0099 | 31.7 |
| Archaea Halobacteria | *Natrialba* | 3 | 11 | 0.0056 | 34.8 |
| Archaea Halobacteria | *Natronomonas* | 3 | No Class | 0.0022 | 43.6 |
| Archaea Halobacteria | *Natronorubrum* | 3 | 8 | 0.002 | 47.8 |
| Archaea Thaumarchaeota | *Nitrososphaera* | 3 | out of the network | 0.0004 | 72.8 |
| Bacteria Acidobacteria | *Acidobacterium* | 3 | 11 | 0.0068 | 38.6 |
| Bacteria Acidobacteria | Candidatus *Koribacter* | 3 | 8 | 0.0077 | 32.1 |
| Bacteria Acidobacteria | *Chloracidobacterium* | 3 | 16 | 0.0052 | 44.2 |
| Bacteria Acidobacteria | *Granulicella* | 3 | 7 | 0.0161 | 25.1 |
| Bacteria Acidobacteria | *Terriglobus* | 3 | 3 | 0.0128 | 28.5 |
| Bacteria Actinobacteria | *Acidimicrobium* | 3 | 3 | 0.0033 | 40 |
| Bacteria Actinobacteria | *Acidipropionibacterium* | 3 | 5 | 0.0157 | 34.3 |
| Bacteria Actinobacteria | *Acidothermus* | 3 | 7 | 0.0037 | 37.7 |
| Bacteria Actinobacteria | *Actinoalloteichus* | 3 | 5 | 0.0085 | 38.6 |
| Bacteria Actinobacteria | *Actinomyces* | 3 | 5 | 0.0785 | 29.4 |
| Bacteria Actinobacteria | *Actinoplanes* | 3 | 5 | 0.0272 | 34.2 |
| Bacteria Actinobacteria | *Actinosynnema* | 3 | 11 | 0.0045 | 45.8 |
| Bacteria Actinobacteria | *Actinotignum* | 3 | 3 | 0.0042 | 38.7 |
| Bacteria Actinobacteria | *Adlercreutzia* | 3 | 5 | 0.0662 | 33 |
| Bacteria Actinobacteria | *Aeromicrobium* | 3 | 3 | 0.0098 | 36.7 |
| Bacteria Actinobacteria | *Agromyces* | 3 | 8 | 0.0056 | 33.6 |
| Bacteria Actinobacteria | *Alloactinosynnema* | 3 | 5 | 0.0036 | 40.2 |
| Bacteria Actinobacteria | *Amycolatopsis* | 3 | 5 | 0.0227 | 32.2 |
| Bacteria Actinobacteria | *Arcanobacterium* | 3 | out of the network | 0.0024 | 31.6 |
| Bacteria Actinobacteria | *Arsenicicoccus* | 3 | 5 | 0.0067 | 36.6 |
| Bacteria Actinobacteria | *Arthrobacter* | 3 | 7 | 0.0513 | 30.4 |
| Bacteria Actinobacteria | *Beutenbergia* | 3 | 18 | 0.0058 | 31.9 |
| Bacteria Actinobacteria | *Blastococcus* | 3 | 7 | 0.0035 | 36.2 |
| Bacteria Actinobacteria | *Brachybacterium* | 3 | 5 | 0.0148 | 33.3 |
| Bacteria Actinobacteria | *Catenulispora* | 3 | 8 | 0.0099 | 33.1 |
| Bacteria Actinobacteria | *Cellulomonas* | 3 | 8 | 0.0244 | 33.9 |
| Bacteria Actinobacteria | *Clavibacter* | 3 | 7 | 0.0201 | 39.1 |
| Bacteria Actinobacteria | *Conexibacter* | 3 | No Class | 0.0129 | 36.2 |
| Bacteria Actinobacteria | *Coriobacterium* | 3 | 7 | 0.0223 | 35.3 |
| Bacteria Actinobacteria | *Corynebacterium* | 3 | 7 | 0.1961 | 30.1 |
| Bacteria Actinobacteria | *Cryobacterium* | 3 | 11 | 0.0079 | 36.7 |
| Bacteria Actinobacteria | *Cryptobacterium* | 3 | No Class | 0.0048 | 42.6 |
| Bacteria Actinobacteria | *Cutibacterium* | 3 | 8 | 0.0096 | 27.4 |
| Bacteria Actinobacteria | *Denitrobacterium* | 3 | 7 | 0.0662 | 31.8 |
| Bacteria Actinobacteria | *Dermacoccus* | 3 | 5 | 0.0045 | 34.1 |
| Bacteria Actinobacteria | *Dietzia* | 3 | 8 | 0.0038 | 37 |
| Bacteria Actinobacteria | *Eggerthella* | 3 | 5 | 0.0982 | 36.7 |
| Bacteria Actinobacteria | *Frankia* | 3 | 5 | 0.0276 | 34 |
| Bacteria Actinobacteria | *Frondihabitans* | 3 | 8 | 0.0058 | 37.9 |
| Bacteria Actinobacteria | *Geodermatophilus* | 3 | 5 | 0.0043 | 36.5 |
| Bacteria Actinobacteria | *Gordonia* | 3 | 3 | 0.0214 | 40.3 |
| Bacteria Actinobacteria | *Gordonibacter* | 3 | No Class | 0.0846 | 40.2 |
| Bacteria Actinobacteria | *Hoyosella* | 3 | out of the network | 0.0017 | 36.2 |
| Bacteria Actinobacteria | *Ilumatobacter* | 3 | 63 | 0.0066 | 38.5 |
| Bacteria Actinobacteria | *Intrasporangium* | 3 | 5 | 0.0045 | 39.1 |
| Bacteria Actinobacteria | *Isoptericola* | 3 | 5 | 0.0104 | 35.4 |
| Bacteria Actinobacteria | *Janibacter* | 3 | out of the network | 0.006 | 50 |
| Bacteria Actinobacteria | *Kibdelosporangium* | 3 | 5 | 0.0046 | 37.5 |
| Bacteria Actinobacteria | *Kitasatospora* | 3 | 5 | 0.0049 | 42 |
| Bacteria Actinobacteria | *Klebsiella* | 3 | 3 | 0.0364 | 30.8 |
| Bacteria Actinobacteria | *Kocuria* | 3 | 5 | 0.0198 | 35.4 |
| Bacteria Actinobacteria | *Kribbella* | 3 | 5 | 0.0056 | 37 |
| Bacteria Actinobacteria | *Kutzneria* | 3 | 7 | 0.0068 | 30.1 |
| Bacteria Actinobacteria | *Kytococcus* | 3 | 7 | 0.0055 | 36.4 |
| Bacteria Actinobacteria | *Lawsonella* | 3 | 5 | 0.0033 | 34.9 |
| Bacteria Actinobacteria | *Leifsonia* | 3 | 8 | 0.0114 | 32.8 |
| Bacteria Actinobacteria | *Lentzea* | 3 | 5 | 0.006 | 30.8 |
| Bacteria Actinobacteria | *Luteipulveratus* | 3 | 3 | 0.0043 | 41.2 |
| Bacteria Actinobacteria | *Microbacterium* | 3 | 7 | 0.055 | 35.1 |
| Bacteria Actinobacteria | *Micrococcus* | 3 | 5 | 0.01 | 37.1 |
| Bacteria Actinobacteria | *Microlunatus* | 3 | 5 | 0.0047 | 39.9 |
| Bacteria Actinobacteria | *Micromonospora* | 3 | 5 | 0.0065 | 36.8 |
| Bacteria Actinobacteria | *Microterricola* | 3 | 8 | 0.0051 | 36.6 |
| Bacteria Actinobacteria | *Mobiluncus* | 3 | 5 | 0.0028 | 45.5 |
| Bacteria Actinobacteria | *Mycobacterium* | 3 | 7 | 0.1213 | 41.1 |
| Bacteria Actinobacteria | *Nakamurella* | 3 | 46 | 0.0063 | 35.2 |
| Bacteria Actinobacteria | *Neomicrococcus* | 3 | 46 | 0.0029 | 37 |
| Bacteria Actinobacteria | *Nocardia* | 3 | 5 | 0.0236 | 34.3 |
| Bacteria Actinobacteria | *Nocardioides* | 3 | 5 | 0.0177 | 32 |
| Bacteria Actinobacteria | *Parascardovia* | 3 | 18 | 0.0115 | 44.1 |
| Bacteria Actinobacteria | *Pimelobacter* | 3 | 5 | 0.0068 | 39 |
| Bacteria Actinobacteria | *Propionibacterium* | 3 | 8 | 0.0133 | 29.6 |
| Bacteria Actinobacteria | *Pseudarthrobacter* | 3 | No Class | 0.0148 | 34.6 |
| Bacteria Actinobacteria | *Pseudonocardia* | 3 | 8 | 0.0186 | 31 |
| Bacteria Actinobacteria | *Pseudopropionibacterium* | 3 | 5 | 0.0092 | 32 |
| Bacteria Actinobacteria | *Rhodococcus* | 3 | 7 | 0.0583 | 29.3 |
| Bacteria Actinobacteria | *Rothia* | 3 | out of the network | 0.0068 | 29.3 |
| Bacteria Actinobacteria | *Rubrobacter* | 3 | 7 | 0.0126 | 33.4 |
| Bacteria Actinobacteria | *Salinispora* | 3 | 5 | 0.0062 | 35.6 |
| Bacteria Actinobacteria | *Serinicoccus* | 3 | No Class | 0.0054 | 39.2 |
| Bacteria Actinobacteria | *Sinomonas* | 3 | 89 | 0.0058 | 41.9 |
| Bacteria Actinobacteria | *Slackia* | 3 | 5 | 0.1117 | 32 |
| Bacteria Actinobacteria | *Streptomyces* | 3 | 5 | 0.2728 | 31.8 |
| Bacteria Actinobacteria | *Streptosporangium* | 3 | 5 | 0.0084 | 39.3 |
| Bacteria Actinobacteria | *Thermobispora* | 3 | 8 | 0.0061 | 38.7 |
| Bacteria Actinobacteria | *Thermomonospora* | 3 | 5 | 0.0065 | 38.2 |
| Bacteria Actinobacteria | *Trueperella* | 3 | 3 | 0.0088 | 32.9 |
| Bacteria Actinobacteria | *Xylanimonas* | 3 | 8 | 0.0081 | 31.9 |
| Bacteria Aquificae | *Aquifex* | 3 | out of the network | 0.0004 | 67 |
| Bacteria Aquificae | *Thermovibrio* | 3 | out of the network | 0.0016 | 35.7 |
| Bacteria Armatimonadetes | *Fimbriimonas* | 3 | No Class | 0.006 | 40.7 |
| Bacteria Bacteroidetes | *Algoriphagus* | 3 | 83 | 0.0048 | 38.9 |
| Bacteria Bacteroidetes | *Alistipes* | 3 | 73 | 0.208 | 25.2 |
| Bacteria Bacteroidetes | *Cytophaga* | 3 | No Class | 0.0042 | 38.9 |
| Bacteria Bacteroidetes | *Draconibacterium* | 3 | 62 | 0.0113 | 22.9 |
| Bacteria Bacteroidetes | *Hymenobacter* | 3 | 73 | 0.1808 | 27.8 |
| Bacteria Bacteroidetes | *Muricauda* | 3 | out of the network | 0.0089 | 35.3 |
| Bacteria Bacteroidetes | *Niastella* | 3 | No Class | 0.008 | 32.3 |
| Bacteria Bacteroidetes | *Ornithobacterium* | 3 | out of the network | 0.0038 | 40.5 |
| Bacteria Bacteroidetes | *Rhodothermus* | 3 | 11 | 0.021 | 35.5 |
| Bacteria Bacteroidetes | *Robiginitalea* | 3 | out of the network | 0.0105 | 32.9 |
| Bacteria Bacteroidetes | *Rufibacter* | 3 | 69 | 0.0342 | 31 |
| Bacteria Bacteroidetes | *Runella* | 3 | 83 | 0.0086 | 33.3 |
| Bacteria Bacteroidetes | *Salinibacter* | 3 | 101 | 0.0109 | 33.5 |
| Bacteria Bacteroidetes | *Saprospira* | 3 | 11 | 0.0048 | 50.3 |
| Bacteria Bacteroidetes | *Spirosoma* | 3 | 73 | 0.0165 | 24.3 |
| Bacteria Bacteroidetes | *Weeksella* | 3 | out of the network | 0.0017 | 44 |
| Bacteria Chlorobi | *Chlorobaculum* | 3 | 8 | 0.0306 | 30 |
| Bacteria Chlorobi | *Pelodictyon* | 3 | No Class | 0.0119 | 33.6 |
| Bacteria Chloroflexi | *Caldilinea* | 3 | 18 | 0.008 | 31.1 |
| Bacteria Chloroflexi | *Dehalogenimonas* | 3 | No Class | 0.0113 | 30.1 |
| Bacteria Chloroflexi | *Sphaerobacter* | 3 | 11 | 0.0097 | 36.5 |
| Bacteria Chrysiogenetes | *Desulfurispirillum* | 3 | 3 | 0.0087 | 33.1 |
| Bacteria Chlorobi | *Chlorobium* | 3 | 3 | 0.0167 | 25.8 |
| Bacteria Chlorobi | *Chloroherpeton* | 3 | out of the network | 0.0024 | 36.7 |
| Bacteria Cyanobacteria | *Chroococcidiopsis* | 3 | No Class | 0.0065 | 54.4 |
| Bacteria Cyanobacteria | *Cyanobium* | 3 | 7 | 0.01 | 34.6 |
| Bacteria Cyanobacteria | *Geitlerinema* | 3 | 11 | 0.0046 | 43.5 |
| Bacteria Cyanobacteria | *Gloeobacter* | 3 | 7 | 0.0154 | 31.7 |
| Bacteria Cyanobacteria | *Pleurocapsa* | 3 | 11 | 0.001 | 59.8 |
| Bacteria Cyanobacteria | *Synechococcus* | 3 | 7 | 0.0496 | 24 |
| Bacteria Cyanobacteria | *Synechocystis* | 3 | 80 | 0.0014 | 32 |
| Bacteria Deinococcus-Thermus | *Deinococcus* | 3 | 5 | 0.0791 | 33 |
| Bacteria Deinococcus-Thermus | *Marinithermus* | 3 | 5 | 0.0057 | 39.9 |
| Bacteria Deinococcus-Thermus | *Meiothermus* | 3 | 11 | 0.0084 | 34.3 |
| Bacteria Deinococcus-Thermus | *Oceanithermus* | 3 | 5 | 0.0118 | 33.3 |
| Bacteria Deinococcus-Thermus | *Thermus* | 3 | 11 | 0.0283 | 42.7 |
| Bacteria Deinococcus-Thermus | *Truepera* | 3 | 3 | 0.0074 | 36.7 |
| Bacteria Desulfitobacterium | *Desulfarculus* | 3 | 11 | 0.0152 | 41.8 |
| Bacteria Firmicutes | *Acetobacterium* | 3 | 31 | 0.0054 | 35.4 |
| Bacteria Firmicutes | *Aerococcus* | 3 | 43 | 0.0121 | 32.8 |
| Bacteria Firmicutes | *Alicyclobacillus* | 3 | 7 | 0.016 | 32.2 |
| Bacteria Firmicutes | *Alkaliphilus* | 3 | No Class | 0.0095 | 38 |
| Bacteria Firmicutes | *Ammonifex* | 3 | 11 | 0.0052 | 44.4 |
| Bacteria Firmicutes | *Brevibacillus* | 3 | No Class | 0.005 | 33.9 |
| Bacteria Firmicutes | *Carboxydothermus* | 3 | out of the network | 0.0025 | 26.8 |
| Bacteria Firmicutes | *Desulfitobacterium* | 3 | 18 | 0.0184 | 28.8 |
| Bacteria Firmicutes | *Desulfosporosinus* | 3 | 18 | 0.0131 | 31.8 |
| Bacteria Firmicutes | *Desulfotomaculum* | 3 | 18 | 0.0243 | 31 |
| Bacteria Firmicutes | *Ethanoligenens* | 3 | 18 | 0.0682 | 35 |
| Bacteria Firmicutes | *Exiguobacterium* | 3 | 3 | 0.0098 | 33.2 |
| Bacteria Firmicutes | *Faecalibaculum* | 3 | 18 | 0.0237 | 34.7 |
| Bacteria Firmicutes | *Filifactor* | 3 | No Class | 0.0078 | 36.3 |
| Bacteria Firmicutes | *Geobacillus* | 3 | 3 | 0.0169 | 34.4 |
| Bacteria Firmicutes | *Geosporobacter* | 3 | No Class | 0.0102 | 40 |
| Bacteria Firmicutes | *Halobacillus* | 3 | out of the network | 0.0013 | 54.1 |
| Bacteria Firmicutes | *Halothermothrix* | 3 | out of the network | 0.002 | 36.7 |
| Bacteria Firmicutes | *Heliobacterium* | 3 | 18 | 0.0248 | 32.1 |
| Bacteria Firmicutes | *Kyrpidia* | 3 | 80 | 0.0044 | 36.8 |
| Bacteria Firmicutes | *Lachnoclostridium* | 3 | 18 | 0.844 | 33 |
| Bacteria Firmicutes | *Lentibacillus* | 3 | 52 | 0.0018 | 44.5 |
| Bacteria Firmicutes | *Mageeibacillus* | 3 | 18 | 0.0126 | 39.6 |
| Bacteria Firmicutes | *Mahella* | 3 | 52 | 0.0038 | 49.4 |
| Bacteria Firmicutes | *Moorella* | 3 | 7 | 0.0114 | 30.3 |
| Bacteria Firmicutes | *Ndongobacter* | 3 | 3 | 0.0079 | 31.4 |
| Bacteria Firmicutes | *Oscillibacter* | 3 | 18 | 0.31 | 35.1 |
| Bacteria Firmicutes | *Paenibacillus* | 3 | 18 | 0.2117 | 28.4 |
| Bacteria Firmicutes | *Peptoclostridium* | 3 | No Class | 0.0089 | 38 |
| Bacteria Firmicutes | *Planococcus* | 3 | No Class | 0.0145 | 33.4 |
| Bacteria Firmicutes | *Ruminiclostridium* | 3 | 5 | 0.1884 | 37.5 |
| Bacteria Firmicutes | *Symbiobacterium* | 3 | 5 | 0.0378 | 34 |
| Bacteria Firmicutes | *Syntrophothermus* | 3 | No Class | 0.0028 | 48.9 |
| Bacteria Firmicutes | *Thermacetogenium* | 3 | No Class | 0.007 | 44.1 |
| Bacteria Firmicutes | *Thermaerobacter* | 3 | 5 | 0.0211 | 38.4 |
| Bacteria Firmicutes | *Thermincola* | 3 | 7 | 0.0056 | 32.8 |
| Bacteria Firmicutes | *Thermobacillus* | 3 | 5 | 0.0276 | 31.5 |
| Bacteria Firmicutes | *Thermosediminibacter* | 3 | 5 | 0.0034 | 46.5 |
| Bacteria Firmicutes | *Tyzzerella* | 3 | 18 | 0.0132 | 35.1 |
| Bacteria Gemmatimonadetes | *Gemmatimonas* | 3 | 73 | 0.0118 | 31.8 |
| Bacteria Ignavibacteriae | *Melioribacter* | 3 | out of the network | 0.0019 | 31.8 |
| Bacteria Nitrospirae | *Leptospirillum* | 3 | out of the network | 0.0025 | 35 |
| Bacteria Nitrospirae | *Nitrospira* | 3 | 7 | 0.0137 | 31.3 |
| Bacteria Planctomycetes | *Phycisphaera* | 3 | 101 | 0.0138 | 35.8 |
| Bacteria α-Proteobacteria | *Acidiphilium* | 3 | 7 | 0.0074 | 31.9 |
| Bacteria α-Proteobacteria | *Agrobacterium* | 3 | 3 | 0.0348 | 35.2 |
| Bacteria α-Proteobacteria | *Altererythrobacter* | 3 | 8 | 0.0183 | 29.6 |
| Bacteria α-Proteobacteria | *Aminobacter* | 3 | 7 | 0.0067 | 34 |
| Bacteria α-Proteobacteria | *Asticcacaulis* | 3 | 3 | 0.0051 | 42.3 |
| Bacteria α-Proteobacteria | *Aureimonas* | 3 | 5 | 0.0079 | 41.6 |
| Bacteria α-Proteobacteria | *Azorhizobium* | 3 | 7 | 0.0094 | 33.6 |
| Bacteria α-Proteobacteria | *Azospirillum* | 3 | 8 | 0.0506 | 30.2 |
| Bacteria α-Proteobacteria | *Bosea* | 3 | 5 | 0.0206 | 29.1 |
| Bacteria α-Proteobacteria | *Bradyrhizobium* | 3 | 8 | 0.0552 | 30.4 |
| Bacteria α-Proteobacteria | *Brevundimonas* | 3 | 7 | 0.0223 | 31.2 |
| Bacteria α-Proteobacteria | *Brucella* | 3 | 16 | 0.0047 | 42.5 |
| Bacteria α-Proteobacteria | *Caulobacter* | 3 | 8 | 0.0246 | 29.6 |
| Bacteria α-Proteobacteria | *Celeribacter* | 3 | 8 | 0.0086 | 36.3 |
| Bacteria α-Proteobacteria | *Chelatococcus* | 3 | 3 | 0.0156 | 42.3 |
| Bacteria α-Proteobacteria | *Citromicrobium* | 3 | 5 | 0.0033 | 39.9 |
| Bacteria α-Proteobacteria | *Confluentimicrobium* | 3 | 8 | 0.0043 | 35.6 |
| Bacteria α-Proteobacteria | *Croceicoccus* | 3 | 7 | 0.0034 | 37.3 |
| Bacteria α-Proteobacteria | *Devosia* | 3 | 7 | 0.0158 | 29.6 |
| Bacteria α-Proteobacteria | *Dinoroseobacter* | 3 | 11 | 0.0039 | 47 |
| Bacteria α-Proteobacteria | *Donghicola* | 3 | out of the network | 0.0021 | 37.1 |
| Bacteria α-Proteobacteria | *Ensifer* | 3 | 18 | 0.0111 | 30.4 |
| Bacteria α-Proteobacteria | *Erythrobacter* | 3 | 5 | 0.0114 | 30.2 |
| Bacteria α-Proteobacteria | *Filomicrobium* | 3 | out of the network | 0.0021 | 39.5 |
| Bacteria α-Proteobacteria | *Gluconacetobacter* | 3 | No Class | 0.0046 | 41.7 |
| Bacteria α-Proteobacteria | *Gluconobacter* | 3 | 5 | 0.0072 | 27.9 |
| Bacteria α-Proteobacteria | *Granulibacter* | 3 | 5 | 0.0061 | 32.6 |
| Bacteria α-Proteobacteria | *Hoeflea* | 3 | 5 | 0.0062 | 36.2 |
| Bacteria α-Proteobacteria | *Hyphomicrobium* | 3 | 5 | 0.0162 | 30.8 |
| Bacteria α-Proteobacteria | *Hyphomonas* | 3 | 7 | 0.0061 | 31.7 |
| Bacteria α-Proteobacteria | *Jannaschia* | 3 | 5 | 0.0033 | 35.8 |
| Bacteria α-Proteobacteria | *Ketogulonicigenium* | 3 | 8 | 0.0029 | 40.3 |
| Bacteria α-Proteobacteria | *Komagataeibacter* | 3 | 5 | 0.0096 | 30.2 |
| Bacteria α-Proteobacteria | *Leisingera* | 3 | 3 | 0.0039 | 33.8 |
| Bacteria α-Proteobacteria | *Magnetococcus* | 3 | out of the network | 0.0024 | 41.1 |
| Bacteria α-Proteobacteria | *Magnetospirillum* | 3 | 11 | 0.024 | 38.8 |
| Bacteria α-Proteobacteria | *Maricaulis* | 3 | 73 | 0.0043 | 34.9 |
| Bacteria α-Proteobacteria | *Martelella* | 3 | 8 | 0.0148 | 33.6 |
| Bacteria α-Proteobacteria | *Mesorhizobium* | 3 | 3 | 0.0451 | 36.3 |
| Bacteria α-Proteobacteria | *Methylobacterium* | 3 | 5 | 0.0559 | 30.2 |
| Bacteria α-Proteobacteria | *Methyloceanibacter* | 3 | 3 | 0.0059 | 37.3 |
| Bacteria α-Proteobacteria | *Methylocella* | 3 | 7 | 0.0041 | 32.5 |
| Bacteria α-Proteobacteria | *Methylocystis* | 3 | 5 | 0.0035 | 44.8 |
| Bacteria α-Proteobacteria | *Micavibrio* | 3 | out of the network | 0.0041 | 35.9 |
| Bacteria α-Proteobacteria | *Neorhizobium* | 3 | 7 | 0.0092 | 33.3 |
| Bacteria α-Proteobacteria | *Neorickettsia* | 3 | out of the network | 0.001 | 43.7 |
| Bacteria α-Proteobacteria | *Nitratireductor* | 3 | out of the network | 0.0038 | 36.3 |
| Bacteria α-Proteobacteria | *Novosphingobium* | 3 | 5 | 0.0185 | 30.3 |
| Bacteria α-Proteobacteria | *Octadecabacter* | 3 | 8 | 0.004 | 38.9 |
| Bacteria α-Proteobacteria | *Pannonibacter* | 3 | 7 | 0.0074 | 34.5 |
| Bacteria α-Proteobacteria | *Paracoccus* | 3 | 7 | 0.012 | 41.3 |
| Bacteria α-Proteobacteria | *Pararhodospirillum* | 3 | out of the network | 0.0044 | 35.1 |
| Bacteria α-Proteobacteria | *Parvibaculum* | 3 | 101 | 0.0041 | 40.3 |
| Bacteria α-Proteobacteria | *Pelagibaca* | 3 | 18 | 0.0059 | 30.7 |
| Bacteria α-Proteobacteria | *Pelagibacterium* | 3 | 3 | 0.0047 | 38 |
| Bacteria α-Proteobacteria | *Phaeobacter* | 3 | 5 | 0.0095 | 33.4 |
| Bacteria α-Proteobacteria | *Polymorphum* | 3 | 8 | 0.0056 | 34.1 |
| Bacteria α-Proteobacteria | *Porphyrobacter* | 3 | 7 | 0.0059 | 38.3 |
| Bacteria α-Proteobacteria | *Rhizobium* | 3 | 3 | 0.0802 | 30.2 |
| Bacteria α-Proteobacteria | *Rhodobacter* | 3 | 3 | 0.0314 | 31.8 |
| Bacteria α-Proteobacteria | *Rhodomicrobium* | 3 | 8 | 0.0047 | 34.1 |
| Bacteria α-Proteobacteria | *Rhodoplanes* | 3 | out of the network | 0.0058 | 39.4 |
| Bacteria α-Proteobacteria | *Rhodopseudomonas* | 3 | 7 | 0.0255 | 30.1 |
| Bacteria α-Proteobacteria | *Rhodospirillum* | 3 | 7 | 0.0171 | 30.1 |
| Bacteria α-Proteobacteria | *Roseibacterium* | 3 | 5 | 0.0036 | 39.6 |
| Bacteria α-Proteobacteria | *Roseobacter* | 3 | 5 | 0.0041 | 30 |
| Bacteria α-Proteobacteria | *Roseomonas* | 3 | 5 | 0.0108 | 33.5 |
| Bacteria α-Proteobacteria | *Ruegeria* | 3 | 7 | 0.0111 | 30.5 |
| Bacteria α-Proteobacteria | *Shinella* | 3 | 8 | 0.009 | 33.9 |
| Bacteria α-Proteobacteria | *Sinorhizobium* | 3 | 7 | 0.0333 | 33.8 |
| Bacteria α-Proteobacteria | *Sphingobium* | 3 | 8 | 0.0345 | 31 |
| Bacteria α-Proteobacteria | *Sphingomonas* | 3 | 8 | 0.0518 | 30.1 |
| Bacteria α-Proteobacteria | *Sphingopyxis* | 3 | 3 | 0.0307 | 30.4 |
| Bacteria α-Proteobacteria | *Starkeya* | 3 | 8 | 0.0066 | 35.1 |
| Bacteria α-Proteobacteria | *Thiobacimonas* | 3 | 7 | 0.0082 | 38.1 |
| Bacteria α-Proteobacteria | *Tistrella* | 3 | 18 | 0.0087 | 28.2 |
| Bacteria α-Proteobacteria | *Xanthobacter* | 3 | 3 | 0.0093 | 37.2 |
| Bacteria α-Proteobacteria | *Yangia* | 3 | 5 | 0.0049 | 37.6 |
| Bacteria β-Proteobacteria | *Achromobacter* | 3 | 7 | 0.034 | 28.8 |
| Bacteria β-Proteobacteria | *Acidovorax* | 3 | 7 | 0.0294 | 29.1 |
| Bacteria β-Proteobacteria | *Alcaligenes* | 3 | out of the network | 0.0033 | 41 |
| Bacteria β-Proteobacteria | *Alicycliphilus* | 3 | 5 | 0.0081 | 36.4 |
| Bacteria β-Proteobacteria | *Aromatoleum* | 3 | 3 | 0.0063 | 41.6 |
| Bacteria β-Proteobacteria | *Azoarcus* | 3 | 3 | 0.0243 | 34.8 |
| Bacteria β-Proteobacteria | *Azospira* | 3 | 3 | 0.0126 | 31.6 |
| Bacteria β-Proteobacteria | *Bordetella* | 3 | 7 | 0.0647 | 29 |
| Bacteria β-Proteobacteria | *Burkholderia* | 3 | 7 | 0.1477 | 30.8 |
| Bacteria β-Proteobacteria | Candidatus *Accumulibacter* | 3 | 3 | 0.0066 | 40 |
| Bacteria β-Proteobacteria | *Castellaniella* | 3 | 3 | 0.011 | 39.3 |
| Bacteria β-Proteobacteria | *Chromobacterium* | 3 | 7 | 0.0164 | 32.8 |
| Bacteria β-Proteobacteria | *Collimonas* | 3 | 80 | 0.0148 | 27.8 |
| Bacteria β-Proteobacteria | *Comamonas* | 3 | 3 | 0.0161 | 27.2 |
| Bacteria β-Proteobacteria | *Cupriavidus* | 3 | 7 | 0.0428 | 28.1 |
| Bacteria β-Proteobacteria | *Dechloromonas* | 3 | 8 | 0.0066 | 28.1 |
| Bacteria β-Proteobacteria | *Delftia* | 3 | 3 | 0.016 | 30.1 |
| Bacteria β-Proteobacteria | *Gallionella* | 3 | out of the network | 0.0021 | 33.1 |
| Bacteria β-Proteobacteria | *Herbaspirillum* | 3 | 3 | 0.0173 | 33.3 |
| Bacteria β-Proteobacteria | *Hydrogenophaga* | 3 | 7 | 0.0169 | 31.3 |
| Bacteria β-Proteobacteria | *Janthinobacterium* | 3 | 3 | 0.0149 | 24.8 |
| Bacteria β-Proteobacteria | *Jeongeupia* | 3 | 7 | 0.0071 | 30.5 |
| Bacteria β-Proteobacteria | *Leptothrix* | 3 | 3 | 0.008 | 35.1 |
| Bacteria β-Proteobacteria | *Limnohabitans* | 3 | out of the network | 0.0049 | 35.6 |
| Bacteria β-Proteobacteria | *Massilia* | 3 | 7 | 0.0255 | 29.1 |
| Bacteria β-Proteobacteria | *Methylibium* | 3 | 8 | 0.0074 | 37.9 |
| Bacteria β-Proteobacteria | *Methyloversatilis* | 3 | 8 | 0.0047 | 37.9 |
| Bacteria β-Proteobacteria | *Mitsuaria* | 3 | 3 | 0.0088 | 32 |
| Bacteria β-Proteobacteria | *Neisseria* | 3 | 3 | 0.0171 | 30.7 |
| Bacteria β-Proteobacteria | *Ottowia* | 3 | 5 | 0.0068 | 35.8 |
| Bacteria β-Proteobacteria | *Pandoraea* | 3 | 3 | 0.0322 | 26.8 |
| Bacteria β-Proteobacteria | *Paraburkholderia* | 3 | 3 | 0.0321 | 30.6 |
| Bacteria β-Proteobacteria | *Paucibacter* | 3 | 7 | 0.006 | 31.3 |
| Bacteria β-Proteobacteria | *Polaromonas* | 3 | No Class | 0.0096 | 37.6 |
| Bacteria β-Proteobacteria | *Pseudogulbenkiania* | 3 | 7 | 0.007 | 30.5 |
| Bacteria β-Proteobacteria | *Ramlibacter* | 3 | 8 | 0.0147 | 33.9 |
| Bacteria β-Proteobacteria | *Rhodoferax* | 3 | 7 | 0.0197 | 27 |
| Bacteria β-Proteobacteria | *Roseateles* | 3 | 5 | 0.0084 | 35 |
| Bacteria β-Proteobacteria | *Rubrivivax* | 3 | 7 | 0.0117 | 31.9 |
| Bacteria β-Proteobacteria | *Snodgrassella* | 3 | out of the network | 0.0011 | 45.7 |
| Bacteria β-Proteobacteria | *Sulfuritalea* | 3 | 3 | 0.0059 | 35.7 |
| Bacteria β-Proteobacteria | *Thauera* | 3 | 3 | 0.0209 | 32 |
| Bacteria β-Proteobacteria | *Thiobacillus* | 3 | 89 | 0.0057 | 39.4 |
| Bacteria β-Proteobacteria | *Thiomonas* | 3 | 3 | 0.0084 | 31.7 |
| Bacteria β-Proteobacteria | *Variovorax* | 3 | 7 | 0.0267 | 29 |
| Bacteria β-Proteobacteria | *Verminephrobacter* | 3 | 5 | 0.005 | 33 |
| Bacteria δ-Proteobacteria | *Anaeromyxobacter* | 3 | 89 | 0.0502 | 33 |
| Bacteria δ-Proteobacteria | *Archangium* | 3 | 8 | 0.0153 | 32 |
| Bacteria δ-Proteobacteria | *Corallococcus* | 3 | 5 | 0.0122 | 38.8 |
| Bacteria δ-Proteobacteria | *Desulfatibacillum* | 3 | No Class | 0.0111 | 43.6 |
| Bacteria δ-Proteobacteria | *Desulfobacca* | 3 | 7 | 0.0037 | 33.6 |
| Bacteria δ-Proteobacteria | *Desulfobacterium* | 3 | 3 | 0.0039 | 31.3 |
| Bacteria δ-Proteobacteria | *Desulfobulbus* | 3 | 7 | 0.0137 | 28.4 |
| Bacteria δ-Proteobacteria | *Desulfocapsa* | 3 | out of the network | 0.0014 | 39.7 |
| Bacteria δ-Proteobacteria | *Desulfococcus* | 3 | 7 | 0.0224 | 27.7 |
| Bacteria δ-Proteobacteria | *Desulfomicrobium* | 3 | 5 | 0.0247 | 32.7 |
| Bacteria δ-Proteobacteria | *Desulfomonile* | 3 | 46 | 0.0025 | 36.7 |
| Bacteria δ-Proteobacteria | *Desulfovibrio* | 3 | 5 | 0.2671 | 31.3 |
| Bacteria δ-Proteobacteria | *Desulfuromonas* | 3 | 5 | 0.0285 | 28.8 |
| Bacteria δ-Proteobacteria | *Geoalkalibacter* | 3 | 3 | 0.0101 | 35.8 |
| Bacteria δ-Proteobacteria | *Geobacter* | 3 | 8 | 0.1246 | 27.1 |
| Bacteria δ-Proteobacteria | *Haliangium* | 3 | 101 | 0.0159 | 35.1 |
| Bacteria δ-Proteobacteria | *Myxococcus* | 3 | 5 | 0.0502 | 29.1 |
| Bacteria δ-Proteobacteria | *Pajaroellobacter* | 3 | out of the network | 0.0004 | 61.2 |
| Bacteria δ-Proteobacteria | *Pelobacter* | 3 | 5 | 0.0352 | 28.3 |
| Bacteria δ-Proteobacteria | *Sandaracinus* | 3 | 89 | 0.0149 | 38.7 |
| Bacteria δ-Proteobacteria | *Sorangium* | 3 | 8 | 0.0292 | 32 |
| Bacteria δ-Proteobacteria | *Stigmatella* | 3 | 5 | 0.0109 | 29.5 |
| Bacteria δ-Proteobacteria | *Syntrophobacter* | 3 | 5 | 0.0078 | 37.3 |
| Bacteria δ-Proteobacteria | *Syntrophus* | 3 | 5 | 0.0055 | 38.4 |
| Bacteria δ-Proteobacteria | *Vulgatibacter* | 3 | 11 | 0.0112 | 37.1 |
| Bacteria ε-Proteobacteria | *Sulfuricurvum* | 3 | out of the network | 0.0007 | 80.7 |
| Bacteria ε-Proteobacteria | *Wolinella* | 3 | out of the network | 0.0008 | 51.9 |
| Bacteria γ-Proteobacteria | *Azotobacter* | 3 | 8 | 0.0105 | 33.8 |
| Bacteria γ-Proteobacteria | *Acidihalobacter* | 3 | 7 | 0.0147 | 23.2 |
| Bacteria γ-Proteobacteria | *Allochromatium* | 3 | 7 | 0.0086 | 30.5 |
| Bacteria γ-Proteobacteria | *Blastomonas* | 3 | 3 | 0.004 | 43.6 |
| Bacteria γ-Proteobacteria | *Brenneria* | 3 | 11 | 0.0048 | 39.7 |
| Bacteria γ-Proteobacteria | *Chromohalobacter* | 3 | 3 | 0.0054 | 39 |
| Bacteria γ-Proteobacteria | *Citrobacter* | 3 | 18 | 0.0233 | 22.8 |
| Bacteria γ-Proteobacteria | *Dyella* | 3 | 3 | 0.0229 | 32.2 |
| Bacteria γ-Proteobacteria | *Ectothiorhodospira* | 3 | 8 | 0.0077 | 31.5 |
| Bacteria γ-Proteobacteria | *Frateuria* | 3 | 8 | 0.0044 | 38.6 |
| Bacteria γ-Proteobacteria | *Hahella* | 3 | 52 | 0.0029 | 41.2 |
| Bacteria γ-Proteobacteria | *Kosakonia* | 3 | 3 | 0.0083 | 28.8 |
| Bacteria γ-Proteobacteria | *Kozakia* | 3 | out of the network | 0.0025 | 37.3 |
| Bacteria γ-Proteobacteria | *Luteibacter* | 3 | 8 | 0.0055 | 27.6 |
| Bacteria γ-Proteobacteria | *Marichromatium* | 3 | 8 | 0.0119 | 36.7 |
| Bacteria γ-Proteobacteria | *Marinobacter* | 3 | 3 | 0.0293 | 26.6 |
| Bacteria γ-Proteobacteria | *Obesumbacterium* | 3 | out of the network | 0.0005 | 51.9 |
| Bacteria γ-Proteobacteria | *Oceanimonas* | 3 | 3 | 0.0096 | 31.2 |
| Bacteria γ-Proteobacteria | *Pectobacterium* | 3 | out of the network | 0.0088 | 28.8 |
| Bacteria γ-Proteobacteria | *Pseudohongiella* | 3 | out of the network | 0.0025 | 29.4 |
| Bacteria γ-Proteobacteria | *Rahnella* | 3 | 3 | 0.0046 | 33.1 |
| Bacteria γ-Proteobacteria | *Raoultella* | 3 | 3 | 0.0087 | 36.5 |
| Bacteria γ-Proteobacteria | *Rhodanobacter* | 3 | 7 | 0.0072 | 35.8 |
| Bacteria γ-Proteobacteria | *Simiduia* | 3 | out of the network | 0.0027 | 40.4 |
| Bacteria γ-Proteobacteria | *Spiribacter* | 3 | 3 | 0.0078 | 38.1 |
| Bacteria γ-Proteobacteria | *Stenotrophomonas* | 3 | 7 | 0.0399 | 28.7 |
| Bacteria γ-Proteobacteria | *Thioalkalimicrobium* | 3 | out of the network | 0.0015 | 48.1 |
| Bacteria γ-Proteobacteria | *Thioalkalivibrio* | 3 | 3 | 0.0376 | 32.2 |
| Bacteria γ-Proteobacteria | *Thiolapillus* | 3 | 46 | 0.005 | 31.8 |
| Bacteria γ-Proteobacteria | *Woeseia* | 3 | 7 | 0.0024 | 31.5 |
| Bacteria Proteobacteria | *Acidithiobacillus* | 3 | 5 | 0.0109 | 27.7 |
| Bacteria Spirochaetes | *Salinispira* | 3 | 7 | 0.0048 | 40.9 |
| Bacteria Spirochaetes | *Sphaerochaeta* | 3 | 7 | 0.02 | 28.4 |
| Bacteria Spirochaetes | *Spirochaeta* | 3 | 8 | 0.02 | 29.3 |
| Bacteria Synergistetes | *Acetomicrobium* | 3 | out of the network | 0.0015 | 42.1 |
| Bacteria Synergistetes | *Aminomonas* | 3 | 7 | 0.0137 | 31.6 |
| Bacteria Synergistetes | *Cloacibacillus* | 3 | 5 | 0.0237 | 32.9 |
| Bacteria Synergistetes | *Jonquetella* | 3 | 3 | 0.0123 | 33.3 |
| Bacteria Synergistetes | *Thermanaerovibrio* | 3 | 46 | 0.0105 | 43.8 |
| Bacteria Thermobaculum | *Thermobaculum* | 3 | out of the network | 0.0018 | 45.3 |
| Bacteria Thermotogae | *Mesotoga* | 3 | out of the network | 0.0007 | 43.8 |
| Bacteria Thermotogae | *Pseudothermotoga* | 3 | out of the network | 0.0021 | 41.6 |
| Bacteria Thermotogae | *Thermotoga* | 3 | 3 | 0.0058 | 26.8 |
| Bacteria Verrucomicrobia | *Akkermansia* | 3 | 11 | 0.0247 | 37.7 |
| Bacteria Verrucomicrobia | *Coraliomargarita* | 3 | No Class | 0.004 | 44 |
| Fungi Ascomycota | *Coniosporium* | 3 | out of the network | 0.0028 | 48.5 |
| Fungi Basidiomycota | *Malassezia* | 3 | out of the network | 0.0026 | 33.3 |
| Fungi Basidiomycota | *Schizophyllum* | 3 | 5 | 0.0027 | 34.6 |
| Plantae Magnolophyta | *Fuerstia* | 3 | 63 | 0.0035 | 42.2 |
| Diamine N-acetyltransferase [EC:2.3.1.57] | K00657 | 3 | No Class | 0.0107 | 61.8 |
| Excinuclease ABC subunit B | K03702 | 3 | 60 | 0.0949 | 31.9 |
| Hydrogenase large subunit [EC:1.12.99.6] | K06281 | 3 | out of the network | 0.001 | 76.8 |
| Maltose/maltodextrin transport system ATP-binding protein | K10112 | 3 | 18 | 0.104 | 34.3 |
| Bacteria Aquificae | *Hydrogenobacter* | 4 | out of the network | 0.0003 | 53 |
| Bacteria Bacteroidetes | Candidatus *Amoebophilus* | 4 | No Class | 0.0014 | 46.5 |
| Bacteria Cyanobacteria | *Dactylococcopsis* | 4 | 16 | 0.0006 | 71.5 |
| Bacteria Firmicutes | *Butyrivibrio* | 4 | No Class | 2.5156 | 45.7 |
| Bacteria Firmicutes | *Pseudobutyrivibrio* | 4 | out of the network | 0.4866 | 41.4 |
| Flagellar hook protein FlgE | K02390 | 4 | out of the network | 0.0017 | 62.8 |
| Flagellar motor switch protein FliN/FliY | K02417 | 4 | out of the network | 0.0016 | 76.2 |
| UDP-N-acetyl-D-galactosamine dehydrogenase [EC:1.1.1.-] | K02474 | 4 | out of the network | 0.003 | 64.2 |
| Two-component system, chemotaxis family, sensor kinase CheA [EC:2.7.13.3] | K03407 | 4 | out of the network | 0.0054 | 60.4 |
| Beta-glucosidase [EC:3.2.1.21] | K05350 | 4 | No Class | 0.0043 | 77.9 |
| Alpha-L-rhamnosidase [EC:3.2.1.40] | K05989 | 4 | No Class | 0.0051 | 82.3 |
| Putative multiple sugar transport system substrate-binding protein | K10546 | 4 | No Class | 0.0029 | 73.3 |
| Bacteria Actinobacteria | *Saccharomonospora* | 5 | No Class | 0.0384 | 148.9 |
| Bacteria Actinobacteria | *Thermobifida* | 5 | No Class | 0.0161 | 178.2 |
| Bacteria Firmicutes | *Bacillus* | 5 | out of the network | 0.1809 | 60.5 |
| Bacteria Spirochaetes | *Sediminispirochaeta* | 5 | out of the network | 0.0052 | 69.9 |
| Pyruvate dehydrogenase E1 component [EC:1.2.4.1] | K00163 | 5 | out of the network | 0.002 | 87.8 |
| 2-oxoglutarate dehydrogenase E1 component [EC:1.2.4.2] | K00164 | 5 | out of the network | 0.0021 | 99 |
| Glycine dehydrogenase [EC:1.4.4.2] | K00281 | 5 | out of the network | 0.0021 | 73.7 |
| Pyruvate dehydrogenase E2 component (dihydrolipoamide acetyltransferase) [EC:2.3.1.12] | K00627 | 5 | out of the network | 0.0013 | 129.2 |
| Isocitrate lyase [EC:4.1.3.1] | K01637 | 5 | out of the network | 0.0012 | 89.6 |
| Enoyl-CoA hydratase [EC:4.2.1.17] | K01692 | 5 | out of the network | 0.0028 | 104.6 |
| Aspartate ammonia-lyase [EC:4.3.1.1] | K01744 | 5 | out of the network | 0.001 | 89.3 |
| Simple sugar transport system permease protein | K02057 | 5 | out of the network | 0.0022 | 63.9 |
| Cytochrome c oxidase subunit I [EC:1.9.3.1] | K02274 | 5 | out of the network | 0.0021 | 91.3 |
| Catalase [EC:1.11.1.6] | K03781 | 5 | out of the network | 0.0022 | 121.9 |
| DEAD/DEAH box helicase domain-containing protein | K06877 | 5 | out of the network | 0.0013 | 112.8 |
| Putative drug exporter of the RND superfamily | K06994 | 5 | out of the network | 0.0021 | 106.8 |
| PhoH-like ATPase | K07175 | 5 | out of the network | 0.0014 | 74.1 |
| Algae Cryptophyta | *Guillardia* | 6 | No Class | 0.0249 | 33.9 |
| Archaea Crenarchaeota | *Acidianus* | 6 | out of the network | 0.0002 | 93.9 |
| Archaea Euryarchaeota | *Acidiplasma* | 6 | 4 | 0.0002 | 96.6 |
| Archaea Euryarchaeota | *Methanocaldococcus* | 6 | 4 | 0.005 | 50.8 |
| Archaea Euryarchaeota | *Methanococcus* | 6 | 4 | 0.0124 | 47.5 |
| Archaea Euryarchaeota | *Methanothermococcus* | 6 | 4 | 0.0032 | 60.6 |
| Archaea Euryarchaeota | *Picrophilus* | 6 | out of the network | 0.0002 | 75.1 |
| Archaea Thaumarchaeota | Candidatus *Nitrosotenuis* | 6 | 4 | 0.0002 | 113.9 |
| Bacteria Actinobacteria | *Tuber* | 6 | 4 | 0.0096 | 62.8 |
| Bacteria Cyanobacteria | *Anabaena* | 6 | 4 | 0.03 | 109.3 |
| Bacteria Cyanobacteria | *Calothrix* | 6 | 4 | 0.4645 | 199.9 |
| Bacteria Cyanobacteria | *Cylindrospermum* | 6 | 4 | 0.0024 | 44.4 |
| Bacteria Cyanobacteria | *Leptolyngbya* | 6 | 65 | 0.0104 | 37.6 |
| Bacteria Cyanobacteria | *Nodularia* | 6 | 4 | 0.0704 | 187.9 |
| Bacteria Cyanobacteria | *Nostoc* | 6 | 4 | 0.8054 | 187.7 |
| Bacteria Cyanobacteria | *Rivularia* | 6 | 4 | 0.0253 | 114 |
| Bacteria Cyanobacteria | *Trichormus* | 6 | 4 | 0.009 | 138.4 |
| Bacteria Deferribacteres | *Deferribacter* | 6 | 75 | 0.0022 | 58 |
| Bacteria β-Proteobacteria | Candidatus *Profftella* | 6 | 75 | 0.0011 | 66 |
| Bacteria γ-Proteobacteria | Candidatus *Carsonella* | 6 | 88 | 0.0017 | 64.6 |
| Bacteria γ-Proteobacteria | Candidatus *Mikella* | 6 | 75 | 0.0008 | 53.5 |
| Bacteria γ-Proteobacteria | *Wigglesworthia* | 6 | 88 | 0.0035 | 45.8 |
| Bacteria γ-Proteobacteria | *Xanthomonas* | 6 | 4 | 0.2242 | 116.1 |
| Bacteria Spirochaetes | *Brachyspira* | 6 | 75 | 0.0168 | 44.1 |
| Protist Euglenozoa Kinetoplastida | *Trypanosoma* | 6 | 4 | 0.0751 | 64.2 |
| Protist Parabasalia Trichomonadida | *Trichomonas* | 6 | 4 | 0.0286 | 72.7 |
| Fungi Ascomycota | *Arthrobotrys* | 6 | 4 | 0.0097 | 64.5 |
| Fungi Ascomycota | *Aspergillus* | 6 | 4 | 0.0385 | 50 |
| Fungi Ascomycota | *Babjeviella* | 6 | out of the network | 0.0014 | 45 |
| Fungi Ascomycota | *Beauveria* | 6 | 4 | 0.006 | 61.9 |
| Fungi Ascomycota | *Bipolaris* | 6 | 4 | 0.0066 | 46.7 |
| Fungi Ascomycota | *Blastomyces* | 6 | 4 | 0.0267 | 72.5 |
| Fungi Ascomycota | *Botrytis* | 6 | 4 | 0.0125 | 40.1 |
| Fungi Ascomycota | *Candida* | 6 | 4 | 0.0453 | 75.2 |
| Fungi Ascomycota | *Chaetomium* | 6 | 4 | 0.0096 | 54.7 |
| Fungi Ascomycota | *Cladophialophora* | 6 | 65 | 0.0097 | 27.7 |
| Fungi Ascomycota | *Clavispora* | 6 | 4 | 0.0106 | 65.3 |
| Fungi Ascomycota | *Coccidioides* | 6 | 4 | 0.0238 | 66.4 |
| Fungi Ascomycota | *Colletotrichum* | 6 | 4 | 0.0299 | 29.4 |
| Fungi Ascomycota | *Cordyceps* | 6 | 4 | 0.0079 | 80.3 |
| Fungi Ascomycota | *Dactylellina* | 6 | 4 | 0.008 | 93 |
| Fungi Ascomycota | *Debaryomyces* | 6 | 4 | 0.0047 | 77.9 |
| Fungi Ascomycota | *Endocarpon* | 6 | No Class | 0.0022 | 46.9 |
| Fungi Ascomycota | *Eremothecium* | 6 | 4 | 0.0037 | 51.1 |
| Fungi Ascomycota | *Eutypa* | 6 | 4 | 0.0258 | 101 |
| Fungi Ascomycota | *Gaeumannomyces* | 6 | 4 | 0.0062 | 45.5 |
| Fungi Ascomycota | *Grosmannia* | 6 | 4 | 0.0049 | 36.7 |
| Fungi Ascomycota | *Histoplasma* | 6 | 4 | 0.0152 | 62 |
| Fungi Ascomycota | *Isaria* | 6 | 4 | 0.054 | 210.2 |
| Fungi Ascomycota | *Kazachstania* | 6 | 4 | 0.0027 | 94.3 |
| Fungi Ascomycota | *Kluyveromyces* | 6 | 4 | 0.0016 | 60.2 |
| Fungi Ascomycota | *Komagataella* | 6 | 4 | 0.0009 | 77.4 |
| Fungi Ascomycota | *Leptosphaeria* | 6 | 4 | 0.0037 | 43.2 |
| Fungi Ascomycota | *Lodderomyces* | 6 | 4 | 0.0293 | 72.4 |
| Fungi Ascomycota | *Magnaporthe* | 6 | 4 | 0.0111 | 48.3 |
| Fungi Ascomycota | *Marssonina* | 6 | 4 | 0.0271 | 64.1 |
| Fungi Ascomycota | *Metarhizium* | 6 | 4 | 0.0196 | 29.6 |
| Fungi Ascomycota | *Metschnikowia* | 6 | No Class | 0.0022 | 49.3 |
| Fungi Ascomycota | *Meyerozyma* | 6 | 4 | 0.0011 | 75.7 |
| Fungi Ascomycota | *Microsporum* | 6 | 4 | 0.0033 | 56.2 |
| Fungi Ascomycota | *Millerozyma* | 6 | 4 | 0.0025 | 58.8 |
| Fungi Ascomycota | *Nakaseomyces* | 6 | 4 | 0.0025 | 61.7 |
| Fungi Ascomycota | *Nannizzia* | 6 | 4 | 0.0029 | 43.9 |
| Fungi Ascomycota | *Naumovozyma* | 6 | 4 | 0.0082 | 89 |
| Fungi Ascomycota | *Neofusicoccum* | 6 | 65 | 0.0038 | 40.2 |
| Fungi Ascomycota | *Neurospora* | 6 | 4 | 0.0228 | 55.6 |
| Fungi Ascomycota | *Paracoccidioides* | 6 | 4 | 0.0395 | 72.9 |
| Fungi Ascomycota | *Penicillium* | 6 | 4 | 0.0101 | 37.3 |
| Fungi Ascomycota | *Pestalotiopsis* | 6 | No Class | 0.0087 | 49.9 |
| Fungi Ascomycota | *Phaeoacremonium* | 6 | 4 | 0.0019 | 49.2 |
| Fungi Ascomycota | *Phialocephala* | 6 | No Class | 0.0038 | 42.7 |
| Fungi Ascomycota | *Pichia* | 6 | 4 | 0.0018 | 62.7 |
| Fungi Ascomycota | *Pneumocystis* | 6 | 4 | 0.0145 | 88.7 |
| Fungi Ascomycota | *Pochonia* | 6 | 4 | 0.0022 | 39.1 |
| Fungi Ascomycota | *Podospora* | 6 | 4 | 0.0049 | 53.6 |
| Fungi Ascomycota | *Pseudogymnoascus* | 6 | No Class | 0.0155 | 42.4 |
| Fungi Ascomycota | *Rasamsonia* | 6 | 4 | 0.004 | 50.5 |
| Fungi Ascomycota | *Rhinocladiella* | 6 | 4 | 0.0027 | 48.3 |
| Fungi Ascomycota | *Saccharomyces* | 6 | 4 | 0.0077 | 76 |
| Fungi Ascomycota | *Scedosporium* | 6 | 4 | 0.0169 | 56.8 |
| Fungi Ascomycota | *Scheffersomyces* | 6 | 4 | 0.0013 | 70.2 |
| Fungi Ascomycota | *Schizosaccharomyces* | 6 | 4 | 0.0121 | 72.7 |
| Fungi Ascomycota | *Sclerotinia* | 6 | 4 | 0.0068 | 49.2 |
| Fungi Ascomycota | *Setosphaeria* | 6 | 4 | 0.0047 | 51 |
| Fungi Ascomycota | *Spathaspora* | 6 | 4 | 0.004 | 80.9 |
| Fungi Ascomycota | *Sphaerulina* | 6 | 4 | 0.0052 | 62.9 |
| Fungi Ascomycota | *Sporothrix* | 6 | 4 | 0.0075 | 40.2 |
| Fungi Ascomycota | *Sugiyamaella* | 6 | 4 | 0.0018 | 69.9 |
| Fungi Ascomycota | *Talaromyces* | 6 | 4 | 0.0047 | 50 |
| Fungi Ascomycota | *Tetrapisispora* | 6 | 4 | 0.0162 | 77 |
| Fungi Ascomycota | *Thermothelomyces* | 6 | 4 | 0.0074 | 40.5 |
| Fungi Ascomycota | *Thielavia* | 6 | 4 | 0.0085 | 40.4 |
| Fungi Ascomycota | *Torulaspora* | 6 | 4 | 0.0007 | 84.9 |
| Fungi Ascomycota | *Trichoderma* | 6 | 4 | 0.0308 | 53.6 |
| Fungi Ascomycota | *Trichophyton* | 6 | 4 | 0.0172 | 60 |
| Fungi Ascomycota | *Uncinocarpus* | 6 | 4 | 0.0013 | 55 |
| Fungi Ascomycota | *Vanderwaltozyma* | 6 | 4 | 0.0058 | 72.9 |
| Fungi Ascomycota | *Verruconis* | 6 | 4 | 0.0037 | 48.2 |
| Fungi Ascomycota | *Wickerhamomyces* | 6 | 4 | 0.0205 | 94.9 |
| Fungi Ascomycota | *Xylona* | 6 | 4 | 0.0071 | 53.5 |
| Fungi Ascomycota | *Yamadazyma* | 6 | 4 | 0.0012 | 99.8 |
| Fungi Ascomycota | *Zygosaccharomyces* | 6 | 4 | 0.0033 | 74.1 |
| Fungi Basidiomycota | *Agaricus* | 6 | 4 | 0.002 | 56.5 |
| Fungi Basidiomycota | *Cryptococcus* | 6 | 4 | 0.0038 | 42.6 |
| Fungi Basidiomycota | *Fomitiporia* | 6 | 4 | 0.0013 | 61.1 |
| Fungi Basidiomycota | *Kwoniella* | 6 | 4 | 0.0105 | 75 |
| Fungi Basidiomycota | *Melampsora* | 6 | 4 | 0.0069 | 71.1 |
| Fungi Basidiomycota | *Mixia* | 6 | 4 | 0.0008 | 100.3 |
| Fungi Basidiomycota | *Moniliophthora* | 6 | 4 | 0.0017 | 48.1 |
| Fungi Basidiomycota | *Puccinia* | 6 | 4 | 0.0126 | 61.9 |
| Fungi Basidiomycota | *Tsuchiyaea* | 6 | 4 | 0.0007 | 47.2 |
| Fungi Basidiomycota | *Wallemia* | 6 | 4 | 0.0042 | 78.6 |
| Fungi Chytridiomycota | *Spizellomyces* | 6 | 4 | 0.0041 | 88.3 |
| Fungi Microsporida | *Mitosporidium* | 6 | 4 | 0.0031 | 114.8 |
| Fungi Microsporidia | *Encephalitozoon* | 6 | 4 | 0.0003 | 88.1 |
| Fungi Microsporidia | *Enterocytozoon* | 6 | 4 | 0.0044 | 62 |
| Fungi Microsporidia | *Nematocida* | 6 | 4 | 0.0005 | 103 |
| Fungi Microsporidia | *Nosema* | 6 | 4 | 0.0037 | 92.6 |
| Fungi Mucoromycota | *Phycomyces* | 6 | 4 | 0.0351 | 72.8 |
| Protist Alveolata Perkinsea | *Perkinsus* | 6 | 4 | 0.0096 | 77.1 |
| Protist Alveolata Ciliophora | *Ichthyophthirius* | 6 | 4 | 0.2841 | 103.4 |
| Protist Alveolata Ciliophora | *Tetrahymena* | 6 | 4 | 0.0605 | 111.9 |
| Protist Alveolata Apicomplexa | *Cryptosporidium* | 6 | 4 | 0.0187 | 90.1 |
| Protist Alveolata Apicomplexa | *Gregarina* | 6 | 4 | 0.0025 | 90 |
| Protist Alveolata Apicomplexa | *Hammondia* | 6 | 65 | 0.0076 | 36.2 |
| Protist Alveolata Apicomplexa | *Neospora* | 6 | 4 | 0.0047 | 45.2 |
| Protist Alveolata Apicomplexa | *Plasmodium* | 6 | 4 | 1.2965 | 77.6 |
| Protist Alveolata Apicomplexa | *Theileria* | 6 | 4 | 0.0135 | 96.9 |
| Protist Alveolata Apicomplexa | *Toxoplasma* | 6 | 4 | 0.0053 | 58.9 |
| Protist Amoebozoa | *Acanthamoeba* | 6 | 4 | 0.0116 | 37.3 |
| Protist Amoebozoa Archamoebae | *Entamoeba* | 6 | 4 | 0.081 | 92.6 |
| Protist Amoebozoa Mycetozoa | *Acytostelium* | 6 | 4 | 0.0063 | 63.3 |
| Protist Amoebozoa Mycetozoa | *Dictyostelium* | 6 | 4 | 1.6272 | 90.4 |
| Protist Apusozoa Apusomonadidae | *Thecamonas* | 6 | No Class | 0.0224 | 27.9 |
| Protist Bigyra | *Blastocystis* | 6 | 4 | 0.0072 | 61.1 |
| Protist Ciliophora | *Paramecium* | 6 | 4 | 0.0371 | 104 |
| Protist Heterokonta | *Aphanomyces* | 6 | 4 | 0.0039 | 37.2 |
| Protist Heterokonta | *Phaeodactylum* | 6 | 4 | 0.0016 | 62.4 |
| Protist Heterokonta | *Phytophthora* | 6 | 4 | 0.0147 | 28.5 |
| Protist Percolozoa | *Naegleria* | 6 | 4 | 0.0203 | 114 |
| Phosphoribosylformylglycinamidine cyclo-ligase [EC:6.3.3.1] | K01933 | 6 | 4 | 0.2617 | 104.2 |
| DNA helicase II / ATP-dependent DNA helicase PcrA [EC:3.6.4.12] | K03657 | 6 | No Class | 0.1234 | 60.5 |
| SEC-C motif domain protein | K09858 | 6 | 4 | 0.2511 | 219.3 |
| Bacteria Actinobacteria | *Bifidobacterium* | 7 | 6 | 1.6475 | 169.2 |
| 6-phosphogluconate dehydrogenase [EC:1.1.1.44] | K00033 | 7 | 6 | 0.0095 | 141.5 |
| Glucose-6-phosphate 1-dehydrogenase [EC:1.1.1.49] | K00036 | 7 | 6 | 0.0081 | 168.7 |
| Lactaldehyde reductase [EC:1.1.1.77] | K00048 | 7 | 6 | 0.0202 | 83.4 |
| Dihydroorotate oxidase [EC:1.3.3.1] | K00226 | 7 | 48 | 0.0223 | 54.5 |
| Dihydrofolate reductase [EC:1.5.1.3] | K00287 | 7 | 26 | 0.0058 | 98.9 |
| NAD(P) transhydrogenase subunit alpha [EC:1.6.1.2] | K00324 | 7 | 6 | 0.006 | 151.5 |
| NAD(P) transhydrogenase subunit beta [EC:1.6.1.2] | K00325 | 7 | 6 | 0.0121 | 106.8 |
| Dihydrolipoamide dehydrogenase [EC:1.8.1.4] | K00382 | 7 | out of the network | 0.0145 | 57.3 |
| Ferredoxin--NADP+ reductase [EC:1.18.1.2] | K00528 | 7 | No Class | 0.034 | 70.2 |
| Methylated-DNA-[protein]-cysteine S-methyltransferase [EC:2.1.1.63] | K00567 | 7 | No Class | 0.0073 | 97.3 |
| Aspartate carbamoyltransferase catalytic subunit [EC:2.1.3.2] | K00609 | 7 | No Class | 0.0392 | 27.5 |
| Aspartate carbamoyltransferase regulatory subunit | K00610 | 7 | No Class | 0.0036 | 73.1 |
| Transaldolase [EC:2.2.1.2] | K00616 | 7 | 6 | 0.0121 | 124.9 |
| Homoserine O-succinyltransferase [EC:2.3.1.46] | K00651 | 7 | No Class | 0.0156 | 75.1 |
| 2,3,4,5-tetrahydropyridine-2-carboxylate N-succinyltransferase [EC:2.3.1.117] | K00674 | 7 | 6 | 0.0061 | 128 |
| Sucrose phosphorylase [EC:2.4.1.7] | K00690 | 7 | 6 | 0.013 | 176.9 |
| 4-alpha-glucanotransferase [EC:2.4.1.25] | K00705 | 7 | 13 | 0.0856 | 48.9 |
| Uracil phosphoribosyltransferase [EC:2.4.2.9] | K00761 | 7 | No Class | 0.0181 | 53.6 |
| Nicotinate phosphoribosyltransferase [EC:2.4.2.11] | K00763 | 7 | 6 | 0.016 | 151 |
| UDP-N-acetylglucosamine 1-carboxyvinyltransferase [EC:2.5.1.7] | K00790 | 7 | 26 | 0.0293 | 67.6 |
| Undecaprenyl diphosphate synthase [EC:2.5.1.31] | K00806 | 7 | 28 | 0.0181 | 50.7 |
| Fructokinase [EC:2.7.1.4] | K00847 | 7 | 55 | 0.0195 | 64 |
| Homoserine kinase [EC:2.7.1.39] | K00872 | 7 | 6 | 0.0044 | 234 |
| Pyruvate kinase [EC:2.7.1.40] | K00873 | 7 | 6 | 0.0342 | 70.4 |
| Polyphosphate kinase [EC:2.7.4.1] | K00937 | 7 | 6 | 0.0258 | 145.9 |
| UDPglucose--hexose-1-phosphate uridylyltransferase [EC:2.7.7.12] | K00965 | 7 | 6 | 0.0083 | 189.9 |
| Glutamate-ammonia-ligase adenylyltransferase [EC:2.7.7.42] | K00982 | 7 | 6 | 0.0052 | 240.4 |
| 2-C-methyl-D-erythritol 4-phosphate cytidylyltransferase [EC:2.7.7.60] | K00991 | 7 | 55 | 0.013 | 53.9 |
| Protein phosphatase [EC:3.1.3.16] | K01090 | 7 | 6 | 0.007 | 210 |
| Beta-fructofuranosidase [EC:3.2.1.26] | K01193 | 7 | No Class | 0.013 | 125.2 |
| Aminopeptidase N [EC:3.4.11.2] | K01256 | 7 | No Class | 0.0188 | 122.4 |
| Ovochymase [EC:3.4.21.-] | K01362 | 7 | 6 | 0.0056 | 217.3 |
| Bleomycin hydrolase [EC:3.4.22.40] | K01372 | 7 | No Class | 0.0224 | 100.9 |
| L-asparaginase [EC:3.5.1.1] | K01424 | 7 | 26 | 0.007 | 123.5 |
| Succinyl-diaminopimelate desuccinylase [EC:3.5.1.18] | K01439 | 7 | 6 | 0.0065 | 147 |
| Choloylglycine hydrolase [EC:3.5.1.24] | K01442 | 7 | No Class | 0.0102 | 97.9 |
| dCTP deaminase [EC:3.5.4.13] | K01494 | 7 | 6 | 0.0047 | 140.1 |
| No such data | K01552 | 7 | 6 | 0.014 | 225 |
| 5-(carboxyamino)imidazole ribonucleotide synthase [EC:6.3.4.18] | K01589 | 7 | 6 | 0.0044 | 192.1 |
| Phosphoenolpyruvate carboxylase [EC:4.1.1.31] | K01595 | 7 | 6 | 0.0273 | 149.2 |
| Threonine aldolase [EC:4.1.2.5] | K01620 | 7 | 20 | 0.0176 | 67.3 |
| 3-deoxy-7-phosphoheptulonate synthase [EC:2.5.1.54] | K01626 | 7 | 6 | 0.013 | 151.4 |
| Fructose-6-phosphate phosphoketolase [EC:4.1.2.22] | K01632 | 7 | 6 | 0.015 | 166.9 |
| L-serine dehydratase [EC:4.3.1.17] | K01752 | 7 | 6 | 0.0039 | 137.5 |
| Threonine dehydratase [EC:4.3.1.19] | K01754 | 7 | 6 | 0.0107 | 152.2 |
| Phosphoglucomutase [EC:5.4.2.2] | K01835 | 7 | 6 | 0.0099 | 188.6 |
| UDP-galactopyranose mutase [EC:5.4.99.9] | K01854 | 7 | 6 | 0.0282 | 98.3 |
| Glutamate--cysteine ligase [EC:6.3.2.2] | K01919 | 7 | 6 | 0.0057 | 169.9 |
| Formate--tetrahydrofolate ligase [EC:6.3.4.3] | K01938 | 7 | 68 | 0.0633 | 41.8 |
| Propionyl-CoA carboxylase beta chain [EC:6.4.1.3] | K01966 | 7 | No Class | 0.0281 | 77.2 |
| Branched-chain amino acid transport system ATP-binding protein | K01995 | 7 | 6 | 0.008 | 125 |
| Branched-chain amino acid transport system ATP-binding protein | K01996 | 7 | 6 | 0.0095 | 162.2 |
| Branched-chain amino acid transport system permease protein | K01997 | 7 | 6 | 0.0079 | 130.8 |
| Branched-chain amino acid transport system permease protein | K01998 | 7 | 6 | 0.0083 | 129.3 |
| Branched-chain amino acid transport system substrate-binding protein | K01999 | 7 | 6 | 0.0153 | 202.3 |
| Putative ABC transport system permease protein | K02004 | 7 | 55 | 0.1346 | 51.6 |
| Cobalt/nickel transport system ATP-binding protein | K02006 | 7 | No Class | 0.0293 | 78.5 |
| Multiple sugar transport system permease protein | K02025 | 7 | 6 | 0.0275 | 62.2 |
| Multiple sugar transport system permease protein | K02026 | 7 | 6 | 0.0293 | 53.3 |
| Polar amino acid transport system ATP-binding protein [EC:3.6.3.21] | K02028 | 7 | No Class | 0.021 | 82.4 |
| Polar amino acid transport system permease protein | K02029 | 7 | 6 | 0.0099 | 195.7 |
| Peptide/nickel transport system permease protein | K02033 | 7 | 68 | 0.0257 | 62.3 |
| Peptide/nickel transport system permease protein | K02034 | 7 | 60 | 0.0311 | 49.3 |
| Phosphate transport system ATP-binding protein [EC:3.6.3.27] | K02036 | 7 | 6 | 0.0088 | 130.2 |
| Phosphate transport system permease protein | K02037 | 7 | 6 | 0.0075 | 133.4 |
| Phosphate transport system permease protein | K02038 | 7 | 6 | 0.0073 | 137.9 |
| Phosphate transport system substrate-binding protein | K02040 | 7 | 55 | 0.0166 | 82.9 |
| D-methionine transport system ATP-binding protein | K02071 | 7 | 26 | 0.0075 | 102.1 |
| D-methionine transport system permease protein | K02072 | 7 | 26 | 0.0074 | 113.9 |
| D-methionine transport system substrate-binding protein | K02073 | 7 | 26 | 0.0091 | 89.9 |
| F-type H+-transporting ATPase subunit gamma [EC:3.6.3.14] | K02115 | 7 | 13 | 0.0332 | 41 |
| Aspartyl-tRNA(Asn)/glutamyl-tRNA (Gln) amidotransferase subunit C [EC:6.3.5.6 6.3.5.7] | K02435 | 7 | 60 | 0.006 | 78.3 |
| Glycogen operon protein GlgX [EC:3.2.1.-] | K02438 | 7 | 6 | 0.0143 | 187.6 |
| Glycerol uptake facilitator protein | K02440 | 7 | 68 | 0.0085 | 92.3 |
| Two-component system, OmpR family, response regulator | K02483 | 7 | 6 | 0.0029 | 144 |
| Two-component system, OmpR family, sensor kinase [EC:2.7.13.3] | K02484 | 7 | 6 | 0.005 | 245.1 |
| 1,4-dihydroxy-2-naphthoate octaprenyltransferase [EC:2.5.1.74] | K02548 | 7 | No Class | 0.0059 | 85.1 |
| Ribosome-binding factor A | K02834 | 7 | 48 | 0.007 | 68.2 |
| DNA-directed RNA polymerase subunit omega [EC:2.7.7.6] | K03060 | 7 | 6 | 0.0022 | 108.3 |
| Single-strand DNA-binding protein | K03111 | 7 | 26 | 0.0104 | 71 |
| Adenylyltransferase [EC:2.7.7.-] | K03148 | 7 | out of the network | 0.0099 | 83.7 |
| Thiamine biosynthesis ThiG | K03149 | 7 | 96 | 0.0204 | 62.7 |
| DNA topoisomerase I [EC:5.99.1.2] | K03168 | 7 | 6 | 0.0445 | 77.5 |
| RNA methyltransferase, TrmH family, group 2 [EC:2.1.1.-] | K03216 | 7 | 6 | 0.0039 | 168.6 |
| RNA methyltransferase, TrmH family [EC:2.1.1.-] | K03218 | 7 | 39 | 0.0125 | 101.5 |
| Amino acid transporter, AAT family | K03293 | 7 | 6 | 0.0199 | 205.6 |
| Basic amino acid/polyamine antiporter, APA family | K03294 | 7 | 6 | 0.0083 | 178.4 |
| Ammonium transporter, Amt family | K03320 | 7 | No Class | 0.0119 | 136.6 |
| Phosphoglucosamine mutase [EC:5.4.2.10] | K03431 | 7 | 6 | 0.0198 | 94.8 |
| Ribonuclease HI [EC:3.1.26.4] | K03469 | 7 | 39 | 0.0054 | 118 |
| Chromosome partitioning protein | K03496 | 7 | No Class | 0.0277 | 43.5 |
| Type III pantothenate kinase [EC:2.7.1.33] | K03525 | 7 | 54 | 0.0073 | 116.6 |
| Ribonuclease P protein component [EC:3.1.26.5] | K03536 | 7 | 54 | 0.003 | 95.1 |
| KUP system potassium uptake protein | K03549 | 7 | 6 | 0.0128 | 164.3 |
| A/G-specific adenine glycosylase [EC:3.2.2.-] | K03575 | 7 | 54 | 0.0072 | 77 |
| ATP-dependent helicase HrpA [EC:3.6.4.13] | K03578 | 7 | 6 | 0.0127 | 242.5 |
| Ubiquinone biosynthesis protein | K03688 | 7 | No Class | 0.0157 | 111.2 |
| Putative hemolysin | K03699 | 7 | 68 | 0.0187 | 77.9 |
| ATP-dependent RNA helicase HelY [EC:3.6.4.-] | K03727 | 7 | 6 | 0.0029 | 173.4 |
| Thiamine biosynthesis lipoprotein | K03734 | 7 | No Class | 0.0098 | 159 |
| Ribosomal-protein-alanine N-acetyltransferase [EC:2.3.1.128] | K03789 | 7 | 26 | 0.0034 | 142.1 |
| Heat shock protein HtpX [EC:3.4.24.-] | K03799 | 7 | 26 | 0.0057 | 127.2 |
| Membrane-associated protein | K03975 | 7 | 6 | 0.0028 | 112.3 |
| Bifunctional UDP-N-acetylglucosamine pyrophosphorylase / Glucosamine-1-phosphate N-acetyltransferase [EC:2.7.7.23 2.3.1.157] | K04042 | 7 | No Class | 0.0151 | 48.6 |
| Acetaldehyde dehydrogenase / alcohol dehydrogenase [EC:1.2.1.10 1.1.1.1] | K04072 | 7 | 6 | 0.0318 | 112 |
| DNA repair protein RadA/Sms | K04485 | 7 | 55 | 0.02 | 61.1 |
| Tellurite resistance protein TerC | K05794 | 7 | No Class | 0.0048 | 88.9 |
| Putative thioredoxin | K05838 | 7 | 61 | 0.0085 | 126.4 |
| Sialate O-acetylesterase [EC:3.1.1.53] | K05970 | 7 | No Class | 0.0245 | 87.7 |
| ATP-binding cassette, subfamily B, bacterial | K06147 | 7 | 26 | 0.1173 | 82 |
| Recombination protein RecR | K06187 | 7 | 22 | 0.009 | 75.5 |
| CrcB protein | K06199 | 7 | No Class | 0.0128 | 175.1 |
| Septum formation protein | K06287 | 7 | 26 | 0.0082 | 105.9 |
| Putative MFS transporter, AGZA family, xanthine/uracil permease | K06901 | 7 | No Class | 0.062 | 41.8 |
| MFS transporter, UMF1 family | K06902 | 7 | 6 | 0.0086 | 167.2 |
| Uncharacterized protein | K07009 | 7 | 6 | 0.0036 | 196 |
| CRISPR-associated endonuclease/helicase Cas3 [EC:3.1.-.- 3.6.4.-] | K07012 | 7 | 6 | 0.0074 | 168.5 |
| Uncharacterized protein | K07040 | 7 | 39 | 0.0071 | 90.7 |
| 16S rRNA (cytidine1402-2'-O)-methyltransferase [EC:2.1.1.198] | K07056 | 7 | 48 | 0.0155 | 75.5 |
| Uncharacterized protein | K07088 | 7 | 6 | 0.0116 | 106.7 |
| S-ribosylhomocysteine lyase [EC:4.4.1.21] | K07173 | 7 | 6 | 0.0083 | 61 |
| tRNA (adenine-N1-)-methyltransferase catalytic subunit [EC:2.1.1.36] | K07442 | 7 | No Class | 0.0029 | 136.5 |
| Hypothetical protein | K07503 | 7 | No Class | 0.0052 | 93.2 |
| Serine/threonine transporter | K07862 | 7 | 68 | 0.009 | 140.3 |
| Phosphotransferase system, enzyme I, PtsI [EC:2.7.3.9] | K08483 | 7 | No Class | 0.0342 | 84.1 |
| Dipeptidase [EC:3.4.-.-] | K08659 | 7 | 6 | 0.0137 | 200 |
| Serine/threonine protein kinase, bacterial [EC:2.7.11.1] | K08884 | 7 | 6 | 0.0239 | 98.2 |
| Hypothetical protein | K09118 | 7 | 6 | 0.0085 | 220.2 |
| Hypothetical protein | K09157 | 7 | 6 | 0.0278 | 82.2 |
| DNA recombination protein RmuC | K09760 | 7 | 55 | 0.0153 | 91.5 |
| Glutamate transport system ATP-binding protein [EC:3.6.3.-] | K10008 | 7 | 6 | 0.0055 | 129.1 |
| Maltose/maltodextrin transport system permease protein | K10109 | 7 | 6 | 0.0058 | 156.7 |
| Multiple sugar transport system permease protein | K10118 | 7 | 6 | 0.007 | 164.6 |
| Multiple sugar transport system permease protein | K10119 | 7 | 6 | 0.0073 | 164.7 |
| Cellobiose transport system ATP-binding protein | K10243 | 7 | 6 | 0.0145 | 131.6 |
| Myosin-crossreactive antigen | K10254 | 7 | 6 | 0.011 | 146.9 |
| Hemolysin III | K11068 | 7 | 54 | 0.004 | 98.1 |
| Acetyl-/propionyl-CoA carboxylase, biotin carboxylase, biotin carboxyl carrier protein [EC:6.3.4.14] | K11263 | 7 | 6 | 0.0079 | 220.7 |
| Aspartate aminotransferase [EC:2.6.1.1] | K11358 | 7 | 6 | 0.0074 | 208.8 |
| Fatty acid synthase, bacteria type [EC:2.3.1.-] | K11533 | 7 | 6 | 0.0487 | 215.6 |
| Dihydrofolate synthase / folylpolyglutamate synthase [EC:6.3.2.12 6.3.2.17] | K11754 | 7 | 26 | 0.0163 | 83.1 |
| Ribonuclease J [EC:3.1.-.-] | K12574 | 7 | 6 | 0.0188 | 125.8 |
| Alanine-synthesizing transaminase [EC:2.6.1.66 2.6.1.2] | K14260 | 7 | 6 | 0.01 | 124.8 |
| Bacteria Bacteroidetes | *Prevotella* | 8 | 2 | 38.6433 | 24.4 |
| L-ribulose-5-phosphate 4-epimerase [EC:5.1.3.4] | K01786 | 8 | 2 | 0.0386 | 34.6 |
| HlyD family secretion protein | K02005 | 8 | 2 | 0.0955 | 42.2 |
| DNA polymerase IV [EC:2.7.7.7] | K02346 | 8 | 2 | 0.0271 | 38.8 |
| N utilization substance protein A | K02600 | 8 | 2 | 0.131 | 28.7 |
| GTP-binding protein Era | K03595 | 8 | 2 | 0.0441 | 35.4 |
| Uncharacterized protein | K06950 | 8 | 2 | 0.1575 | 22.3 |
| N-succinyl-L-ornithine transcarbamylase [EC:2.1.3.11] | K13043 | 8 | 2 | 0.032 | 41.5 |
| Animalia Molusca | *Murdochiella* | 9 | No Class | 0.0138 | 38.3 |
| Archaea Euryarchaeota | Candidatus *Methanomethylophilus* | 9 | 12 | 0.0491 | 152.5 |
| Archaea Euryarchaeota | Candidatus *Methanoperedens* | 9 | 33 | 0.0019 | 104.2 |
| Archaea Euryarchaeota | *Halolamina* | 9 | 3 | 0.0136 | 44.6 |
| Archaea Euryarchaeota | *Haloplanus* | 9 | 3 | 0.0017 | 58.8 |
| Archaea Euryarchaeota | *Halorhabdus* | 9 | 3 | 0.0036 | 51 |
| Archaea Euryarchaeota | *Halosimplex* | 9 | No Class | 0.0019 | 66.7 |
| Archaea Euryarchaeota | *Methanoregula* | 9 | 3 | 0.0028 | 44.8 |
| Archaea Halobacteria | *Natrinema* | 9 | 12 | 0.002 | 45.8 |
| Bacteria Actinobacteria | *Atopobium* | 9 | No Class | 0.0089 | 39.8 |
| Bacteria Actinobacteria | *Curtobacterium* | 9 | 3 | 0.0118 | 49.8 |
| Bacteria Actinobacteria | *Dermabacter* | 9 | 3 | 0.0042 | 53.4 |
| Bacteria Actinobacteria | *Libanicoccus* | 9 | No Class | 0.1052 | 46.1 |
| Bacteria Actinobacteria | *Modestobacter* | 9 | 3 | 0.006 | 55.7 |
| Bacteria Actinobacteria | *Rathayibacter* | 9 | 3 | 0.0064 | 45.8 |
| Bacteria Actinobacteria | *Sanguibacter* | 9 | 3 | 0.0074 | 52.7 |
| Bacteria Actinobacteria | *Tsukamurella* | 9 | 3 | 0.0047 | 60 |
| Bacteria Armatimonadetes | *Chthonomonas* | 9 | 3 | 0.0025 | 52.9 |
| Bacteria Calditrichaeota | *Caldithrix* | 9 | 98 | 0.0059 | 30.5 |
| Bacteria Clamidiae | *Parachlamydia* | 9 | out of the network | 0.0004 | 62.8 |
| Bacteria Cloroflexi | *Dehalococcoides* | 9 | 33 | 0.0316 | 92.5 |
| Bacteria Cyanobacteria | *Acaryochloris* | 9 | 3 | 0.0018 | 52.7 |
| Bacteria Firmicutes | *Acidaminococcus* | 9 | 12 | 0.5603 | 160.7 |
| Bacteria Firmicutes | *Allisonella* | 9 | 12 | 0.0165 | 117.7 |
| Bacteria Firmicutes | *Aneurinibacillus* | 9 | 12 | 0.0045 | 66 |
| Bacteria Firmicutes | *Blautia* | 9 | 32 | 0.2909 | 46.8 |
| Bacteria Firmicutes | *Dialister* | 9 | 33 | 0.0042 | 46.9 |
| Bacteria Firmicutes | *Dorea* | 9 | 32 | 0.0769 | 75 |
| Bacteria Firmicutes | *Erysipelatoclostridium* | 9 | 18 | 0.0072 | 43.2 |
| Bacteria Firmicutes | *Eubacterium* | 9 | 33 | 1.0026 | 58.9 |
| Bacteria Firmicutes | *Faecalitalea* | 9 | 32 | 0.0133 | 89.9 |
| Bacteria Firmicutes | *Jeotgalibacillus* | 9 | 33 | 0.0028 | 37.6 |
| Bacteria Firmicutes | *Jeotgalicoccus* | 9 | 12 | 0.0024 | 51.5 |
| Bacteria Firmicutes | *Lachnospira* | 9 | 32 | 0.055 | 45.9 |
| Bacteria Firmicutes | *Lactobacillus* | 9 | No Class | 0.3213 | 71.9 |
| Bacteria Firmicutes | *Megasphaera* | 9 | 12 | 0.2269 | 182 |
| Bacteria Firmicutes | *Mitsuokella* | 9 | 12 | 0.2472 | 132.5 |
| Bacteria Firmicutes | *Oribacterium* | 9 | No Class | 0.3424 | 35.1 |
| Bacteria Firmicutes | *Pelosinus* | 9 | 33 | 0.0152 | 36.3 |
| Bacteria Firmicutes | *Proteiniclasticum* | 9 | No Class | 0.0096 | 36.2 |
| Bacteria Firmicutes | *Roseburia* | 9 | 32 | 0.1356 | 104.9 |
| Bacteria Firmicutes | *Salimicrobium* | 9 | 33 | 0.0025 | 40 |
| Bacteria Firmicutes | *Salinicoccus* | 9 | 12 | 0.0039 | 58.4 |
| Bacteria Firmicutes | *Syntrophobotulus* | 9 | 3 | 0.0066 | 43.7 |
| Bacteria Firmicutes | *Thermodesulfobium* | 9 | No Class | 0.0019 | 59.5 |
| Bacteria Firmicutes | *Veillonella* | 9 | 33 | 0.0042 | 51.7 |
| Bacteria Firmicutes | *Acetitomaculum* | 9 | 32 | 0.0496 | 37.7 |
| Bacteria α-Proteobacteria | *Acetobacter* | 9 | 3 | 0.0084 | 32 |
| Bacteria α-Proteobacteria | *Beijerinckia* | 9 | 3 | 0.0024 | 42.5 |
| Bacteria α-Proteobacteria | Candidatus *Pelagibacter* | 9 | No Class | 0.0025 | 56.1 |
| Bacteria α-Proteobacteria | Candidatus *Phaeomarinobacter* | 9 | 3 | 0.003 | 49.2 |
| Bacteria α-Proteobacteria | *Chelativorans* | 9 | 3 | 0.0076 | 40.6 |
| Bacteria α-Proteobacteria | *Defluviimonas* | 9 | 3 | 0.0059 | 53.4 |
| Bacteria α-Proteobacteria | *Halocynthiibacter* | 9 | 3 | 0.0014 | 50.9 |
| Bacteria α-Proteobacteria | *Nitrobacter* | 9 | 3 | 0.0072 | 45.3 |
| Bacteria α-Proteobacteria | *Rhodovulum* | 9 | 3 | 0.0062 | 42.6 |
| Bacteria α-Proteobacteria | *Sulfitobacter* | 9 | 3 | 0.0048 | 36.9 |
| Bacteria α-Proteobacteria | *Thalassospira* | 9 | 12 | 0.0034 | 45.7 |
| Bacteria β-Proteobacteria | *Advenella* | 9 | 33 | 0.0049 | 38.5 |
| Bacteria β-Proteobacteria | Candidatus *Methylopumilus* | 9 | out of the network | 0.0014 | 59.7 |
| Bacteria β-Proteobacteria | *Nitrosospira* | 9 | 3 | 0.0065 | 89.8 |
| Bacteria β-Proteobacteria | *Pusillimonas* | 9 | out of the network | 0.0041 | 61.9 |
| Bacteria β-Proteobacteria | *Ralstonia* | 9 | 3 | 0.0416 | 39.8 |
| Bacteria β-Proteobacteria | *Sideroxydans* | 9 | 3 | 0.0067 | 61 |
| Bacteria ε-Proteobacteria | *Sulfurovum* | 9 | 12 | 0.005 | 29.2 |
| Bacteria γ-Proteobacteria | *Actinobacillus* | 9 | 3 | 0.0066 | 50.1 |
| Bacteria γ-Proteobacteria | *Aeromonas* | 9 | 3 | 0.0953 | 70.7 |
| Bacteria γ-Proteobacteria | *Alcanivorax* | 9 | 3 | 0.0151 | 48.3 |
| Bacteria γ-Proteobacteria | *Alkalilimnicola* | 9 | 3 | 0.009 | 47.8 |
| Bacteria γ-Proteobacteria | Candidatus *Ishikawaella* | 9 | 3 | 0.0029 | 145.4 |
| Bacteria γ-Proteobacteria | *Cellvibrio* | 9 | 3 | 0.0025 | 34.1 |
| Bacteria γ-Proteobacteria | *Colwellia* | 9 | 3 | 0.0042 | 53.1 |
| Bacteria γ-Proteobacteria | *Cronobacter* | 9 | 3 | 0.0265 | 59.7 |
| Bacteria γ-Proteobacteria | *Dickeya* | 9 | 3 | 0.0395 | 50 |
| Bacteria γ-Proteobacteria | *Dokdonella* | 9 | 3 | 0.0083 | 46.4 |
| Bacteria γ-Proteobacteria | *Edwardsiella* | 9 | 3 | 0.0223 | 49.3 |
| Bacteria γ-Proteobacteria | *Enterobacter* | 9 | 3 | 0.0495 | 31.3 |
| Bacteria γ-Proteobacteria | *Erwinia* | 9 | 3 | 0.0146 | 37.4 |
| Bacteria γ-Proteobacteria | *Ferrimonas* | 9 | 3 | 0.0068 | 43.9 |
| Bacteria γ-Proteobacteria | *Frischella* | 9 | 3 | 0.0054 | 145.4 |
| Bacteria γ-Proteobacteria | *Grimontia* | 9 | No Class | 0.0031 | 68.4 |
| Bacteria γ-Proteobacteria | *Halioglobus* | 9 | 3 | 0.0037 | 53.9 |
| Bacteria γ-Proteobacteria | *Halomonas* | 9 | 3 | 0.0288 | 40.5 |
| Bacteria γ-Proteobacteria | *Halorhodospira* | 9 | 3 | 0.0093 | 49.8 |
| Bacteria γ-Proteobacteria | *Halotalea* | 9 | 3 | 0.0067 | 55.8 |
| Bacteria γ-Proteobacteria | *Halothiobacillus* | 9 | 3 | 0.008 | 35 |
| Bacteria γ-Proteobacteria | *Histophilus* | 9 | 3 | 0.007 | 90.2 |
| Bacteria γ-Proteobacteria | *Immundisolibacter* | 9 | 3 | 0.0115 | 44.3 |
| Bacteria γ-Proteobacteria | *Lacimicrobium* | 9 | No Class | 0.0024 | 48.4 |
| Bacteria γ-Proteobacteria | *Leclercia* | 9 | 3 | 0.0036 | 60.5 |
| Bacteria γ-Proteobacteria | *Methylomonas* | 9 | 3 | 0.0136 | 52.3 |
| Bacteria γ-Proteobacteria | *Microbulbifer* | 9 | 3 | 0.0124 | 54.3 |
| Bacteria γ-Proteobacteria | *Moraxella* | 9 | 3 | 0.0097 | 79.7 |
| Bacteria γ-Proteobacteria | *Morganella* | 9 | No Class | 0.0049 | 50.2 |
| Bacteria γ-Proteobacteria | *Pantoea* | 9 | 3 | 0.0262 | 45.7 |
| Bacteria γ-Proteobacteria | *Pluralibacter* | 9 | 3 | 0.0108 | 44.1 |
| Bacteria γ-Proteobacteria | *Pseudomonas* | 9 | 3 | 0.3938 | 36.4 |
| Bacteria γ-Proteobacteria | *Pseudoxanthomonas* | 9 | 3 | 0.0196 | 40.9 |
| Bacteria γ-Proteobacteria | *Sedimenticola* | 9 | 3 | 0.0063 | 38.3 |
| Bacteria γ-Proteobacteria | *Serratia* | 9 | 3 | 0.0513 | 34 |
| Bacteria γ-Proteobacteria | *Shimwellia* | 9 | 3 | 0.0044 | 45 |
| Bacteria β-Proteobacteria | *Sodalis* | 9 | 3 | 0.0117 | 54.6 |
| Bacteria γ-Proteobacteria | *Thiocystis* | 9 | 3 | 0.0112 | 35.6 |
| Bacteria γ-Proteobacteria | *Thioflavicoccus* | 9 | 3 | 0.012 | 37.3 |
| Bacteria γ-Proteobacteria | *Thioploca* | 9 | 3 | 0.0017 | 119.8 |
| Bacteria γ-Proteobacteria | *Tolumonas* | 9 | 3 | 0.0103 | 105.5 |
| Bacteria γ-Proteobacteria | *Wenzhouxiangella* | 9 | 3 | 0.008 | 87.4 |
| Bacteria Proteobacteria | *Bdellovibrio* | 9 | 3 | 0.008 | 31.4 |
| Fungi Ascomycota | *Glarea* | 9 | out of the network | 0.0016 | 74.3 |
| Fungi Basidiomycota | *Kalmanozyma* | 9 | 3 | 0.0016 | 70.8 |
| Fungi Basidiomycota | *Tilletiaria* | 9 | 3 | 0.0015 | 65.1 |
| L-lactate dehydrogenase [EC:1.1.1.27] | K00016 | 9 | 6 | 0.0138 | 178.2 |
| Malate dehydrogenase (oxaloacetate-decarboxylating) [EC:1.1.1.38] | K00027 | 9 | 103 | 0.0105 | 107.6 |
| 3-hydroxybutyryl-CoA dehydrogenase [EC:1.1.1.157] | K00074 | 9 | 40 | 0.0196 | 61.9 |
| Glycerol-1-phosphate dehydrogenase [NAD(P)] [EC:1.1.1.261] | K00096 | 9 | 40 | 0.0048 | 105.4 |
| Glycolate oxidase [EC:1.1.3.15] | K00104 | 9 | 53 | 0.0032 | 198.1 |
| Glycerol-3-phosphate dehydrogenase [EC:1.1.5.3] | K00111 | 9 | 53 | 0.0043 | 210.3 |
| Succinate-semialdehyde dehydrogenase (NADP+) [EC:1.2.1.16] | K00135 | 9 | 53 | 0.003 | 152.8 |
| Butyryl-CoA dehydrogenase [EC:1.3.99.2] | K00248 | 9 | 78 | 0.0108 | 89.9 |
| Glutamate dehydrogenase [EC:1.4.1.2] | K00260 | 9 | 53 | 0.0037 | 170 |
| Glutamate synthase (ferredoxin) [EC:1.4.7.1] | K00284 | 9 | 87 | 0.0118 | 175.1 |
| Hydroxylamine reductase [EC:1.7.-.-] | K00378 | 9 | 12 | 0.0445 | 55.3 |
| DNA (cytosine-5-)-methyltransferase [EC:2.1.1.37] | K00558 | 9 | 12 | 0.0108 | 63.9 |
| Precorrin-6Y C5,15-methyltransferase / precorrin-8W decarboxylase [EC:2.1.1.132 1.-.-.-] | K00595 | 9 | No Class | 0.0085 | 96.5 |
| Methyltransferase-like protein 6 [EC:2.1.1.-] | K00599 | 9 | No Class | 0.0245 | 91 |
| Gamma-glutamyltranspeptidase / glutathione hydrolase [EC:2.3.2.2 3.4.19.13] | K00681 | 9 | 53 | 0.0025 | 154 |
| Ribonuclease Z [EC:3.1.26.11] | K00784 | 9 | No Class | 0.0062 | 93.6 |
| Spermidine synthase [EC:2.5.1.16] | K00797 | 9 | No Class | 0.0049 | 119.6 |
| Acetylornithine/N-succinyldiaminopimelate aminotransferase [EC:2.6.1.11 2.6.1.17] | K00821 | 9 | No Class | 0.0238 | 75.9 |
| Glycerol kinase [EC:2.7.1.30] | K00864 | 9 | 87 | 0.0063 | 112.4 |
| Shikimate kinase [EC:2.7.1.71] | K00891 | 9 | 86 | 0.0065 | 104.6 |
| Carbamate kinase [EC:2.7.2.2] | K00926 | 9 | 12 | 0.0072 | 121.9 |
| Pantetheine-phosphate adenylyltransferase [EC:2.7.7.3] | K00954 | 9 | 54 | 0.0039 | 93.8 |
| Glucose-1-phosphate adenylyltransferase [EC:2.7.7.27] | K00975 | 9 | No Class | 0.0353 | 91 |
| Deoxyribonuclease IV [EC:3.1.21.2] | K01151 | 9 | out of the network | 0.0066 | 80.7 |
| Type I restriction enzyme, R subunit [EC:3.1.21.3] | K01153 | 9 | 12 | 0.0764 | 82.2 |
| Type I restriction enzyme, S subunit [EC:3.1.21.3] | K01154 | 9 | 12 | 0.011 | 47.7 |
| 6-phospho-beta-glucosidase [EC:3.2.1.86] | K01223 | 9 | out of the network | 0.0029 | 89.1 |
| Tripeptide aminopeptidase [EC:3.4.11.4] | K01258 | 9 | No Class | 0.0209 | 58.3 |
| Putative membrane protein | K01421 | 9 | 6 | 0.0124 | 215.7 |
| Agmatinase [EC:3.5.3.11] | K01480 | 9 | 103 | 0.0036 | 162 |
| Adenosine deaminase [EC:3.5.4.4] | K01488 | 9 | 87 | 0.0035 | 194.2 |
| Cytidine deaminase [EC:3.5.4.5] | K01489 | 9 | 104 | 0.0021 | 123.9 |
| Cu2+-exporting ATPase [EC:3.6.3.4] | K01533 | 9 | No Class | 0.0239 | 79.9 |
| Cd2+/Zn2+-exporting ATPase [EC:3.6.3.3 3.6.3.5] | K01534 | 9 | No Class | 0.0196 | 70.4 |
| Oxaloacetate decarboxylase, alpha subunit [EC:4.1.1.3] | K01571 | 9 | 78 | 0.0165 | 68.6 |
| Indole-3-glycerol phosphate synthase [EC:4.1.1.48] | K01609 | 9 | No Class | 0.0089 | 63.4 |
| Glutaconyl-CoA decarboxylase [EC:4.1.1.70] | K01615 | 9 | 12 | 0.0075 | 158.5 |
| Anthranilate synthase component II [EC:4.1.3.27] | K01658 | 9 | 20 | 0.0049 | 104.4 |
| Fumarate hydratase subunit beta [EC:4.2.1.2] | K01678 | 9 | out of the network | 0.0052 | 78.3 |
| Fumarate hydratase, class II [EC:4.2.1.2] | K01679 | 9 | 3 | 0.0095 | 56.6 |
| Glucarate dehydratase [EC:4.2.1.40] | K01706 | 9 | 53 | 0.002 | 186.9 |
| Urocanate hydratase [EC:4.2.1.49] | K01712 | 9 | 12 | 0.0104 | 67.7 |
| 3-dehydroquinate synthase [EC:4.2.3.4] | K01735 | 9 | No Class | 0.0068 | 101.8 |
| Phosphopentomutase [EC:5.4.2.7] | K01839 | 9 | No Class | 0.0059 | 131 |
| Glycyl-tRNA synthetase alpha chain [EC:6.1.1.14] | K01878 | 9 | 12 | 0.0204 | 74.8 |
| Glycyl-tRNA synthetase beta chain [EC:6.1.1.14] | K01879 | 9 | 12 | 0.0043 | 128.2 |
| AT-rich DNA-binding protein | K01926 | 9 | out of the network | 0.002 | 104.9 |
| Acetyl-CoA carboxylase, biotin carboxylase subunit [EC:6.4.1.2 6.3.4.14] | K01961 | 9 | 3 | 0.0047 | 84.7 |
| Acetyl-CoA carboxylase carboxyl transferase subunit beta [EC:6.4.1.2] | K01963 | 9 | out of the network | 0.0022 | 85.5 |
| M1 RNA | K01978 | 9 | 32 | 0.0203 | 52.5 |
| ABC-2 type transport system permease protein | K01992 | 9 | No Class | 0.0043 | 193.5 |
| Iron(III) transport system ATP-binding protein [EC:3.6.3.30] | K02010 | 9 | 3 | 0.0098 | 64.4 |
| Iron(III) transport system permease protein | K02011 | 9 | 32 | 0.0119 | 70.8 |
| Iron(III) transport system substrate-binding protein | K02012 | 9 | 33 | 0.0102 | 75.4 |
| Iron complex transport system ATP-binding protein [EC:3.6.3.34] | K02013 | 9 | 54 | 0.0084 | 91.9 |
| Cobyrinic acid a,c-diamide synthase [EC:6.3.5.9 6.3.1.-] | K02224 | 9 | out of the network | 0.0117 | 56.4 |
| Adenosylcobinamide-phosphate synthase CobD [EC:6.3.1.10] | K02227 | 9 | 38 | 0.0031 | 134.5 |
| Adenosylcobinamide kinase / adenosylcobinamide-phosphate guanylyltransferase [EC:2.7.1.156 2.7.7.62] | K02231 | 9 | 38 | 0.0021 | 156.9 |
| Adenosylcobinamide-GDP ribazoletransferase [EC:2.7.8.26] | K02233 | 9 | out of the network | 0.0032 | 107.3 |
| Pilus assembly protein CpaF | K02283 | 9 | No Class | 0.0071 | 87.2 |
| DNA polymerase bacteriophage-type [EC:2.7.7.7] | K02334 | 9 | 12 | 0.0157 | 122.8 |
| Enoyl-[acyl carrier protein] reductase II [EC:1.3.1.-] | K02371 | 9 | out of the network | 0.0031 | 141.2 |
| Flagellar biosynthesis protein FlhA | K02400 | 9 | 3 | 0.0035 | 79.6 |
| Flagellin | K02406 | 9 | 78 | 0.0096 | 116.8 |
| LacI family transcriptional regulator | K02529 | 9 | 54 | 0.0271 | 72 |
| Carbohydrate diacid regulator | K02647 | 9 | 87 | 0.0052 | 100.1 |
| Dihydroorotate dehydrogenase electron transfer subunit | K02823 | 9 | No Class | 0.0074 | 74.3 |
| RNA polymerase primary sigma factor | K03086 | 9 | 3 | 0.1638 | 38.9 |
| RNA polymerase sporulation-specific sigma factor | K03091 | 9 | No Class | 0.0071 | 155.6 |
| Thiamine biosynthesis protein ThiI | K03151 | 9 | out of the network | 0.0045 | 112.6 |
| 4-methyl-5(b-hydroxyethyl)-thiazole monophosphate biosynthesis | K03152 | 9 | 104 | 0.0023 | 127.1 |
| Type IV secretion system protein VirD4 | K03205 | 9 | No Class | 0.0065 | 51 |
| Glycoside/pentoside/hexuronide:cation symporter, GPH family | K03292 | 9 | 38 | 0.011 | 125 |
| Methyl-accepting chemotaxis protein | K03406 | 9 | 78 | 0.0236 | 119.8 |
| Type I restriction enzyme M protein [EC:2.1.1.72] | K03427 | 9 | 3 | 0.0479 | 76.1 |
| S-adenosyl-methyltransferase [EC:2.1.1.-] | K03438 | 9 | 92 | 0.0164 | 73.1 |
| Trk system potassium uptake protein TrkH | K03498 | 9 | No Class | 0.0252 | 70.5 |
| BirA family transcriptional regulator, biotin operon repressor / biotin-[acetyl-CoA-carboxylase] ligase [EC:6.3.4.15] | K03524 | 9 | 10 | 0.0055 | 122.9 |
| ATP-dependent Clp protease ATP-binding subunit ClpX | K03544 | 9 | 12 | 0.0836 | 25.4 |
| Exonuclease SbcC | K03546 | 9 | No Class | 0.0061 | 90.1 |
| Regulatory protein | K03565 | 9 | 38 | 0.0025 | 127.5 |
| Septum site-determining protein MinD | K03609 | 9 | 103 | 0.0035 | 162.2 |
| Electron transport complex protein RnfG | K03612 | 9 | 38 | 0.002 | 131.4 |
| Molybdenum cofactor biosynthesis protein C | K03637 | 9 | 40 | 0.003 | 102.3 |
| CMP-binding protein | K03698 | 9 | 103 | 0.004 | 171.2 |
| Cold shock protein (beta-ribbon, CspA family) | K03704 | 9 | 32 | 0.0054 | 88.9 |
| Heat-inducible transcriptional repressor | K03705 | 9 | No Class | 0.0048 | 141.2 |
| DNA polymerase III subunit alpha, Gram-positive type [EC:2.7.7.7] | K03763 | 9 | No Class | 0.0241 | 96 |
| D-lactate dehydrogenase [EC:1.1.1.28] | K03778 | 9 | No Class | 0.0037 | 96.2 |
| Cell division protease FtsH [EC:3.4.24.-] | K03798 | 9 | No Class | 0.1709 | 24.9 |
| 3-deoxy-7-phosphoheptulonate synthase [EC:2.5.1.54] | K03856 | 9 | 12 | 0.0057 | 71.5 |
| ArsR family transcriptional regulator | K03892 | 9 | No Class | 0.0173 | 88.5 |
| Cysteine desulfurase [EC:2.8.1.7] | K04487 | 9 | 19 | 0.0122 | 104.7 |
| Threonine-phosphate decarboxylase [EC:4.1.1.81] | K04720 | 9 | 38 | 0.0034 | 160.4 |
| Conserved Hypothetical protein | K05810 | 9 | 104 | 0.0038 | 145.5 |
| Putative ABC transport system ATP-binding protein | K05833 | 9 | 12 | 0.004 | 214.7 |
| Segregation and condensation protein A | K05896 | 9 | No Class | 0.0025 | 153.6 |
| Cardiolipin synthase [EC:2.7.8.-] | K06131 | 9 | 38 | 0.0093 | 159.6 |
| ATP-binding cassette, subfamily C, bacterial | K06148 | 9 | No Class | 0.0061 | 184.1 |
| Carbon starvation protein | K06200 | 9 | 6 | 0.011 | 149.4 |
| Copper homeostasis protein | K06201 | 9 | 38 | 0.0029 | 150.3 |
| Magnesium transporter | K06213 | 9 | out of the network | 0.0038 | 80.3 |
| SpoIIIJ-associated protein | K06346 | 9 | No Class | 0.0045 | 118.3 |
| Stage V sporulation protein G | K06412 | 9 | out of the network | 0.0019 | 88.4 |
| UPF0042 nucleotide-binding protein | K06958 | 9 | 6 | 0.004 | 156.7 |
| Presequence protease [EC:3.4.24.-] | K06972 | 9 | 12 | 0.0094 | 180.6 |
| Uncharacterized protein | K07007 | 9 | No Class | 0.0122 | 110 |
| Uncharacterized protein | K07011 | 9 | 38 | 0.0076 | 93.3 |
| Putative hydrolases of HD superfamily | K07023 | 9 | No Class | 0.0048 | 170.8 |
| Sucrose-6-phosphatase  [EC:3.1.3.24] | K07024 | 9 | No Class | 0.0198 | 107.2 |
| Uncharacterized protein | K07030 | 9 | 12 | 0.0112 | 97.6 |
| Uncharacterized protein | K07043 | 9 | 104 | 0.0031 | 160.4 |
| Uncharacterized protein | K07052 | 9 | 38 | 0.0085 | 178.1 |
| Uncharacterized protein | K07137 | 9 | No Class | 0.0345 | 68.9 |
| Uncharacterized protein | K07138 | 9 | 53 | 0.0021 | 150.1 |
| Chromate transporter | K07240 | 9 | 86 | 0.0134 | 92.1 |
| D-alanyl-D-alanine carboxypeptidase (penicillin-binding protein 5/6) [EC:3.4.16.4] | K07258 | 9 | 38 | 0.0049 | 133.9 |
| Poly-gamma-glutamate synthesis protein (capsule biosynthesis protein) | K07282 | 9 | 6 | 0.005 | 177.4 |
| Inner membrane protein | K07301 | 9 | No Class | 0.0099 | 95.1 |
| Putative protein-disulfide isomerase | K07396 | 9 | 12 | 0.0173 | 122 |
| DNA mismatch repair protein MutS2 | K07456 | 9 | 86 | 0.0237 | 82.6 |
| Putative endonuclease | K07460 | 9 | 10 | 0.0019 | 107 |
| DNA-damage-inducible protein J | K07473 | 9 | 12 | 0.0063 | 152.1 |
| Putative transposase | K07496 | 9 | 30 | 0.006 | 103.6 |
| TdcF protein | K07567 | 9 | out of the network | 0.0027 | 82.8 |
| Metallo-beta-lactamase family protein | K07576 | 9 | No Class | 0.0043 | 213.6 |
| Antibiotic transport system ATP-binding protein | K09687 | 9 | No Class | 0.0202 | 115.7 |
| Hypothetical protein | K09762 | 9 | 6 | 0.0041 | 148 |
| Zinc transport system substrate-binding protein | K09815 | 9 | No Class | 0.0048 | 144.3 |
| Uncharacterized protein | K09861 | 9 | 32 | 0.0044 | 207.9 |
| Putative glutamine transport system ATP-binding protein [EC:3.6.3.-] | K10041 | 9 | 32 | 0.0016 | 112.6 |
| Lactose/L-arabinose transport system substrate-binding protein | K10188 | 9 | out of the network | 0.0033 | 150 |
| Spermidine/putrescine transport system permease protein | K11070 | 9 | out of the network | 0.0014 | 103.1 |
| Spermidine/putrescine transport system permease protein | K11071 | 9 | 12 | 0.0022 | 220.2 |
| Spermidine/putrescine transport system ATP-binding protein [EC:3.6.3.31] | K11072 | 9 | 32 | 0.0037 | 154.8 |
| Phosphoribosyl-ATP pyrophosphohydrolase / phosphoribosyl-AMP cyclohydrolase [EC:3.6.1.31 3.5.4.19] | K11755 | 9 | 35 | 0.0076 | 103.1 |
| Beta-galactosidase [EC:3.2.1.23] | K12308 | 9 | No Class | 0.017 | 106.8 |
| Aminobenzoyl-glutamate transport protein | K12942 | 9 | No Class | 0.0052 | 185.7 |
| Phosphatidylglycerol:prolipoprotein diacylglycerol transferase [EC:2.-.-.-] | K13292 | 9 | 19 | 0.0052 | 107.5 |
| Chorismate mutase / prephenate dehydratase [EC:5.4.99.5 4.2.1.51] | K14170 | 9 | 12 | 0.0035 | 164.3 |
| Bacteria Firmicutes | *Selenomonas* | 10 | 30 | 2.5841 | 80.6 |
| Peroxiredoxin Q/BCP [EC:1.11.1.15] | K03564 | 10 | out of the network | 0.0111 | 67.4 |
| Fur family transcriptional regulator, ferric uptake regulator | K03711 | 10 | out of the network | 0.0068 | 105.6 |
| Ethanolamine ammonia-lyase large subunit | K03735 | 10 | out of the network | 0.0016 | 99.6 |
| DNA adenine methylase [EC:2.1.1.72] | K06223 | 10 | out of the network | 0.0052 | 148 |
| Maltose/maltodextrin transport system substrate-binding protein | K10108 | 10 | 6 | 0.0099 | 178 |
| Maltose/maltodextrin transport system permease protein | K10110 | 10 | 6 | 0.0069 | 184.6 |

* Clustering equivalency in a network analysis computed with relative abundances in centered log ratio coordinates

# Table S1B. Composition of the different clusters identified in a network analysis using data transformed in log ratio coordinates in SPARCC (microbial genera and their genes) and applying a correlation threshold of 0.70.

| **Microbial taxon/gene** | **Genus/KEGG gene id** | **Cluster SPARCC** |
| --- | --- | --- |
| Hydroxymethylglutaryl-CoA reductase (NADPH) [EC:1.1.1.34] | K00021 | 1 |
| Formate dehydrogenase, alpha subunit [EC:1.2.1.2] | K00123 | 1 |
| Formate dehydrogenase, beta subunit [EC:1.2.1.2] | K00125 | 1 |
| Glyceraldehyde-3-phosphate dehydrogenase (NAD(P)) [EC:1.2.1.59] | K00150 | 1 |
| Pyruvate ferredoxin oxidoreductase, alpha subunit [EC:1.2.7.1] | K00169 | 1 |
| Pyruvate ferredoxin oxidoreductase, beta subunit [EC:1.2.7.1] | K00170 | 1 |
| Pyruvate ferredoxin oxidoreductase, delta subunit [EC:1.2.7.1] | K00171 | 1 |
| Pyruvate ferredoxin oxidoreductase, gamma subunit [EC:1.2.7.1] | K00172 | 1 |
| 2-oxoisovalerate ferredoxin oxidoreductase, alpha subunit [EC:1.2.7.7] | K00186 | 1 |
| 2-oxoisovalerate ferredoxin oxidoreductase, beta subunit [EC:1.2.7.7] | K00187 | 1 |
| 2-oxoisovalerate ferredoxin oxidoreductase, delta subunit [EC:1.2.7.7] | K00188 | 1 |
| Carbon-monoxide dehydrogenase iron sulfur subunit | K00196 | 1 |
| Formylmethanofuran dehydrogenase subunit A [EC:1.2.99.5] | K00200 | 1 |
| Formylmethanofuran dehydrogenase subunit B [EC:1.2.99.5] | K00201 | 1 |
| Formylmethanofuran dehydrogenase subunit C [EC:1.2.99.5] | K00202 | 1 |
| Formylmethanofuran dehydrogenase subunit D [EC:1.2.99.5] | K00203 | 1 |
| Formylmethanofuran dehydrogenase subunit H [EC:1.2.99.5] | K00204 | 1 |
| Formylmethanofuran dehydrogenase subunit F [EC:1.2.99.5] | K00205 | 1 |
| Fumarate reductase iron-sulfur protein [EC:1.3.99.1] | K00245 | 1 |
| Methylenetetrahydromethanopterin dehydrogenase [EC:1.5.99.9] | K00319 | 1 |
| Coenzyme F420-dependent N5,N10-methenyltetrahydromethanopterin reductase [EC:1.5.99.11] | K00320 | 1 |
| Methyl-coenzyme M reductase alpha subunit [EC:2.8.4.1] | K00399 | 1 |
| Methyl coenzyme M reductase system, component A2 | K00400 | 1 |
| Methyl-coenzyme M reductase beta subunit [EC:2.8.4.1] | K00401 | 1 |
| Methyl-coenzyme M reductase gamma subunit [EC:2.8.4.1] | K00402 | 1 |
| Coenzyme F420 hydrogenase alpha subunit [EC:1.12.98.1] | K00440 | 1 |
| Coenzyme F420 hydrogenase beta subunit [EC:1.12.98.1] | K00441 | 1 |
| Coenzyme F420 hydrogenase delta subunit | K00442 | 1 |
| Coenzyme F420 hydrogenase gamma subunit [EC:1.12.98.1] | K00443 | 1 |
| Tetrahydromethanopterin S-methyltransferase subunit A [EC:2.1.1.86] | K00577 | 1 |
| Tetrahydromethanopterin S-methyltransferase subunit C [EC:2.1.1.86] | K00579 | 1 |
| Tetrahydromethanopterin S-methyltransferase subunit D [EC:2.1.1.86] | K00580 | 1 |
| Tetrahydromethanopterin S-methyltransferase subunit E [EC:2.1.1.86] | K00581 | 1 |
| Tetrahydromethanopterin S-methyltransferase subunit G [EC:2.1.1.86] | K00583 | 1 |
| Tetrahydromethanopterin S-methyltransferase subunit H [EC:2.1.1.86] | K00584 | 1 |
| Chloramphenicol O-acetyltransferase [EC:2.3.1.28] | K00638 | 1 |
| Glycine C-acetyltransferase [EC:2.3.1.29] | K00639 | 1 |
| Formylmethanofuran--tetrahydromethanopterin N-formyltransferase [EC:2.3.1.101] | K00672 | 1 |
| Nucleoside-diphosphate kinase [EC:2.7.4.6] | K00940 | 1 |
| Pyruvate, water dikinase [EC:2.7.9.2] | K01007 | 1 |
| L-seryl-tRNA(Ser) seleniumtransferase [EC:2.9.1.1] | K01042 | 1 |
| Phosphoserine phosphatase [EC:3.1.3.3] | K01079 | 1 |
| Adenosylhomocysteinase [EC:3.3.1.1] | K01251 | 1 |
| Methenyltetrahydromethanopterin cyclohydrolase [EC:3.5.4.27] | K01499 | 1 |
| Nucleoside-triphosphatase [EC:3.6.1.15] | K01516 | 1 |
| Arsenite-transporting ATPase [EC:3.6.3.16] | K01551 | 1 |
| Fructose 1,6-bisphosphate aldolase/phosphatase [EC:4.1.2.13 3.1.3.11] | K01622 | 1 |
| Fructose-bisphosphate aldolase, class I [EC:4.1.2.13] | K01623 | 1 |
| N-acetylneuraminate synthase [EC:2.5.1.56] | K01654 | 1 |
| Carbonic anhydrase [EC:4.2.1.1] | K01673 | 1 |
| 6-pyruvoyl tetrahydrobiopterin synthase [EC:4.2.3.12] | K01737 | 1 |
| isopentenyl-diphosphate delta-isomerase [EC:5.3.3.2] | K01823 | 1 |
| Succinyl-CoA synthetase alpha subunit [EC:6.2.1.5] | K01902 | 1 |
| Succinyl-CoA synthetase beta subunit [EC:6.2.1.5] | K01903 | 1 |
| NAD+ synthase [EC:6.3.1.5] | K01916 | 1 |
| Pyruvate carboxylase subunit A [EC:6.4.1.1] | K01959 | 1 |
| SRP RNA | K01983 | 1 |
| Cobalt/nickel transport system permease protein | K02007 | 1 |
| Cobalt transport protein | K02009 | 1 |
| Molybdate transport system permease protein | K02018 | 1 |
| Molybdate transport system regulatory protein | K02019 | 1 |
| V-type H+-transporting ATPase subunit A [EC:3.6.3.14] | K02117 | 1 |
| V-type H+-transporting ATPase subunit B [EC:3.6.3.14] | K02118 | 1 |
| V-type H+-transporting ATPase subunit C [EC:3.6.3.14] | K02119 | 1 |
| V-type H+-transporting ATPase subunit D [EC:3.6.3.14] | K02120 | 1 |
| V-type H+-transporting ATPase subunit E [EC:3.6.3.14] | K02121 | 1 |
| V-type H+-transporting ATPase subunit F [EC:3.6.3.14] | K02122 | 1 |
| V-type H+-transporting ATPase subunit I [EC:3.6.3.14] | K02123 | 1 |
| V-type H+-transporting ATPase subunit K [EC:3.6.3.14] | K02124 | 1 |
| Ferritin [EC:1.16.3.1] | K02217 | 1 |
| Uroporphyrin-III C-methyltransferase [EC:2.1.1.107] | K02303 | 1 |
| DNA polymerase I [EC:2.7.7.7] | K02319 | 1 |
| DNA polymerase II large subunit [EC:2.7.7.7] | K02322 | 1 |
| DNA polymerase II small subunit [EC:2.7.7.7] | K02323 | 1 |
| FdhD protein | K02379 | 1 |
| UDP-N-acetyl-D-mannosaminuronic acid dehydrogenase [EC:1.1.1.-] | K02472 | 1 |
| Glutamyl-tRNA reductase [EC:1.2.1.70] | K02492 | 1 |
| Nitrogen fixation protein NifB | K02585 | 1 |
| DNA primase [EC:2.7.7.-] | K02683 | 1 |
| 3,4-dihydroxy 2-butanone 4-phosphate synthase [EC:4.1.99.12] | K02858 | 1 |
| Large subunit ribosomal protein L10e | K02866 | 1 |
| Large subunit ribosomal protein L12 | K02869 | 1 |
| Large subunit ribosomal protein L14e | K02875 | 1 |
| Large subunit ribosomal protein L15e | K02877 | 1 |
| Large subunit ribosomal protein L18e | K02883 | 1 |
| Large subunit ribosomal protein L19e | K02885 | 1 |
| Large subunit ribosomal protein L21e | K02889 | 1 |
| Large subunit ribosomal protein L24e | K02896 | 1 |
| Large subunit ribosomal protein L30e | K02908 | 1 |
| Large subunit ribosomal protein L31e | K02910 | 1 |
| Large subunit ribosomal protein L32e | K02912 | 1 |
| Large subunit ribosomal protein L34e | K02915 | 1 |
| Large subunit ribosomal protein L37Ae | K02921 | 1 |
| Large subunit ribosomal protein L37e | K02922 | 1 |
| Large subunit ribosomal protein L39e | K02924 | 1 |
| Large subunit ribosomal protein L40e | K02927 | 1 |
| Large subunit ribosomal protein L44e | K02929 | 1 |
| Large subunit ribosomal protein L4e | K02930 | 1 |
| Large subunit ribosomal protein L7Ae | K02936 | 1 |
| Large subunit ribosomal protein LX | K02944 | 1 |
| Small subunit ribosomal protein S17e | K02962 | 1 |
| Small subunit ribosomal protein S19e | K02966 | 1 |
| Small subunit ribosomal protein S24e | K02974 | 1 |
| Small subunit ribosomal protein S27e | K02978 | 1 |
| Small subunit ribosomal protein S28e | K02979 | 1 |
| Small subunit ribosomal protein S3Ae | K02984 | 1 |
| Small subunit ribosomal protein S4e | K02987 | 1 |
| Small subunit ribosomal protein S6e | K02991 | 1 |
| Small subunit ribosomal protein S8e | K02995 | 1 |
| DNA-directed RNA polymerase subunit Aprime [EC:2.7.7.6] | K03041 | 1 |
| DNA-directed RNA polymerase subunit Aprime [EC:2.7.7.6] | K03042 | 1 |
| DNA-directed RNA polymerase subunit Bprime [EC:2.7.7.6] | K03044 | 1 |
| DNA-directed RNA polymerase subunit Bprime [EC:2.7.7.6] | K03045 | 1 |
| DNA-directed RNA polymerase subunit D [EC:2.7.7.6] | K03047 | 1 |
| DNA-directed RNA polymerase subunit Eprime [EC:2.7.7.6] | K03049 | 1 |
| DNA-directed RNA polymerase subunit K [EC:2.7.7.6] | K03055 | 1 |
| DNA-directed RNA polymerase subunit N [EC:2.7.7.6] | K03058 | 1 |
| DNA-directed RNA polymerase subunit P [EC:2.7.7.6] | K03059 | 1 |
| Translation initiation factor eIF-1 | K03113 | 1 |
| Transcription initiation factor TFIID TATA-box-binding protein | K03120 | 1 |
| Transcription initiation factor TFIIB | K03124 | 1 |
| Transcription initiation factor TFIIE alpha subunit | K03136 | 1 |
| DNA topoisomerase VI subunit A [EC:5.99.1.3] | K03166 | 1 |
| DNA topoisomerase VI subunit B [EC:5.99.1.3] | K03167 | 1 |
| Elongation factor EF-1 alpha subunit [EC:3.6.5.3] | K03231 | 1 |
| Elongation factor EF-1 beta subunit | K03232 | 1 |
| Elongation factor EF-2 [EC:3.6.5.3] | K03234 | 1 |
| Translation initiation factor eIF-1A | K03236 | 1 |
| Translation initiation factor eIF-2 alpha subunit | K03237 | 1 |
| Translation initiation factor eIF-2 beta subunit | K03238 | 1 |
| Translation initiation factor eIF-2 gamma subunit | K03242 | 1 |
| Translation initiation factor eIF-5B | K03243 | 1 |
| Translation initiation factor eIF-5A | K03263 | 1 |
| Translation initiation factor eIF-6 | K03264 | 1 |
| Peptide chain release factor eRF subunit 1 | K03265 | 1 |
| Heterodisulfide reductase subunit A [EC:1.8.98.1] | K03388 | 1 |
| Heterodisulfide reductase subunit B [EC:1.8.98.1] | K03389 | 1 |
| Heterodisulfide reductase subunit C [EC:1.8.98.1] | K03390 | 1 |
| Proteasome regulatory subunit | K03420 | 1 |
| Methyl-coenzyme M reductase subunit C | K03421 | 1 |
| Methyl-coenzyme M reductase subunit D | K03422 | 1 |
| Proteasome alpha subunit [EC:3.4.25.1] | K03432 | 1 |
| Proteasome beta subunit [EC:3.4.25.1] | K03433 | 1 |
| Ribonuclease P protein subunit POP4 [EC:3.1.26.5] | K03538 | 1 |
| Ribonuclease P protein subunit RPR2 [EC:3.1.26.5] | K03540 | 1 |
| Holliday junction resolvase, archaea type | K03552 | 1 |
| Molybdopterin synthase catalytic subunit [EC:2.-.-.-] | K03635 | 1 |
| Exosome complex component RRP4 | K03679 | 1 |
| DtxR family transcriptional regulator, Mn-dependent transcriptional regulator | K03709 | 1 |
| Archaea-specific helicase [EC:3.6.1.-] | K03725 | 1 |
| Helicase [EC:3.6.4.-] | K03726 | 1 |
| Aldehyde:ferredoxin oxidoreductase [EC:1.2.7.5] | K03738 | 1 |
| Molybdopterin biosynthesis protein MoeA | K03750 | 1 |
| Molybdopterin-guanine dinucleotide biosynthesis protein A | K03752 | 1 |
| Molybdopterin-guanine dinucleotide biosynthesis protein B | K03753 | 1 |
| Peptidyl-prolyl cis-trans isomerase B (cyclophilin B) [EC:5.2.1.8] | K03768 | 1 |
| Sirohydrochlorin cobaltochelatase [EC:4.99.1.3] | K03795 | 1 |
| NADH dehydrogenase [EC:1.6.99.3] | K03885 | 1 |
| Lon-like ATP-dependent protease [EC:3.4.21.-] | K04076 | 1 |
| Chorismate mutase [EC:5.4.99.5] | K04093 | 1 |
| DNA repair protein RadA | K04483 | 1 |
| Lysyl-tRNA synthetase, class I [EC:6.1.1.6] | K04566 | 1 |
| Hydrogenase nickel incorporation protein HypB | K04652 | 1 |
| Hydrogenase expression/formation protein HypC | K04653 | 1 |
| Hydrogenase expression/formation protein HypD | K04654 | 1 |
| Hydrogenase expression/formation protein HypE | K04655 | 1 |
| Hydrogenase maturation protein HypF | K04656 | 1 |
| Peptidyl-tRNA hydrolase, PTH2 family [EC:3.1.1.29] | K04794 | 1 |
| Fibrillarin-like pre-rRNA processing protein | K04795 | 1 |
| Small nuclear ribonucleoprotein | K04796 | 1 |
| Prefoldin alpha subunit | K04797 | 1 |
| Prefoldin beta subunit | K04798 | 1 |
| Replication factor C small subunit | K04801 | 1 |
| Proliferating cell nuclear antigen | K04802 | 1 |
| (R)-2-hydroxyacid dehydrogenase [EC:1.1.1.272] | K05884 | 1 |
| Precorrin-6X reductase [EC:1.3.1.54] | K05895 | 1 |
| Precorrin-8X methylmutase [EC:5.4.1.2] | K06042 | 1 |
| ATP-binding cassette, sub-family E, member 1 | K06174 | 1 |
| tRNA pseudouridine synthase D [EC:5.4.99.12] | K06176 | 1 |
| 5-formaminoimidazole-4-carboxamide-1-(beta)-D-ribofuranosyl 5prime-monophosphate synthetase [EC:6.3.4.-] | K06863 | 1 |
| ATPase | K06865 | 1 |
| Programmed cell death protein 5 | K06875 | 1 |
| tRNA(Ile2)-agmatinylcytidine synthase [EC:6.3.4.22] | K06932 | 1 |
| 7,8-dihydro-6-hydroxymethylpterin dimethyltransferase [EC:2.1.1.-] | K06937 | 1 |
| Nucleolar GTP-binding protein | K06943 | 1 |
| Uncharacterized protein | K06944 | 1 |
| Ribosomal RNA assembly protein | K06961 | 1 |
| Beta-ribofuranosylaminobenzene 5'-phosphate synthase [EC:2.4.2.54] | K06984 | 1 |
| Uncharacterized protein | K06988 | 1 |
| Uncharacterized protein | K07013 | 1 |
| No such data | K07021 | 1 |
| Uncharacterized protein | K07041 | 1 |
| Uncharacterized protein | K07068 | 1 |
| (4-(4-[2-(gamma-L-glutamylamino)ethyl]phenoxymethyl)furan-2-yl)methanamine synthase [EC:2.5.1.131] | K07072 | 1 |
| Putative glycerol-1-phosphate prenyltransferase [EC:2.5.1.-] | K07094 | 1 |
| Uncharacterized protein | K07096 | 1 |
| Uncharacterized protein | K07108 | 1 |
| Uncharacterized protein | K07135 | 1 |
| 5-(aminomethyl)-3-furanmethanol phosphate kinase [EC:2.7.4.31] | K07144 | 1 |
| Dolichyl-diphosphooligosaccharide--protein glycosyltransferase [EC:2.4.1.119] | K07151 | 1 |
| Uncharacterized protein | K07158 | 1 |
| Uncharacterized protein | K07159 | 1 |
| Uncharacterized protein | K07161 | 1 |
| Hypothetical protein | K07254 | 1 |
| Hydrogenase expression/formation protein | K07388 | 1 |
| AAA family ATPase | K07392 | 1 |
| Putative methyltransferase | K07446 | 1 |
| Archaea-specific RecJ-like exonuclease | K07463 | 1 |
| Diphthamide synthase subunit DPH2 | K07561 | 1 |
| RNA-binding protein | K07569 | 1 |
| Putative nucleotide binding protein | K07572 | 1 |
| Exosome complex component CSL4 | K07573 | 1 |
| Putative RNA-binding protein containing KH domain | K07574 | 1 |
| Hypothetical protein | K07580 | 1 |
| Hypothetical protein | K07582 | 1 |
| Hypothetical protein | K07585 | 1 |
| ArsR family transcriptional regulator | K07721 | 1 |
| CopG family transcriptional regulator, nickel-responsive regulator | K07722 | 1 |
| Putative transcriptional regulator | K07728 | 1 |
| Putative transcriptional regulator | K07730 | 1 |
| Elongator complex protein 3 [EC:2.3.1.48] | K07739 | 1 |
| Hypothetical protein | K07744 | 1 |
| 6-phospho-3-hexuloisomerase [EC:5.3.1.27] | K08094 | 1 |
| GTP cyclohydrolase IIa [EC:3.5.4.29] | K08096 | 1 |
| Methylthioribose-1-phosphate isomerase [EC:5.3.1.23] | K08963 | 1 |
| Hypothetical protein | K09003 | 1 |
| Hypothetical protein | K09007 | 1 |
| Hypothetical protein | K09116 | 1 |
| Hypothetical protein | K09123 | 1 |
| Hypothetical protein | K09128 | 1 |
| Hypothetical protein | K09136 | 1 |
| Hypothetical protein | K09140 | 1 |
| Hypothetical protein | K09142 | 1 |
| Hypothetical protein | K09154 | 1 |
| Glutamyl-tRNA(Gln) amidotransferase subunit D [EC:6.3.5.7] | K09482 | 1 |
| Hypothetical protein | K09717 | 1 |
| Hypothetical protein | K09720 | 1 |
| Hypothetical protein | K09726 | 1 |
| Hypothetical protein | K09728 | 1 |
| Hypothetical protein | K09733 | 1 |
| Hypothetical protein | K09735 | 1 |
| Archaeal cell division control protein 6 | K10725 | 1 |
| Replicative DNA helicase Mcm [EC:3.6.4.-] | K10726 | 1 |
| Fanconi anemia group M protein [EC:3.6.4.13] | K10896 | 1 |
| Trans-homoaconitate synthase [EC:4.1.3.-] | K10977 | 1 |
| IMP cyclohydrolase [EC:3.5.4.10] | K11176 | 1 |
| Formylmethanofuran dehydrogenase subunit G [EC:1.2.99.5] | K11260 | 1 |
| Formylmethanofuran dehydrogenase subunit E [EC:1.2.99.5] | K11261 | 1 |
| Exosome complex component RRP41 | K11600 | 1 |
| Dehydroquinate synthase II [EC:1.4.1.-] | K11646 | 1 |
| FO synthase subunit 1 [EC:2.5.1.-] | K11780 | 1 |
| FO synthase subunit 2 [EC:2.5.1.-] | K11781 | 1 |
| Exosome complex component RRP42 | K12589 | 1 |
| Transitional endoplasmic reticulum ATPase | K13525 | 1 |
| Geranylgeranyl diphosphate synthase, type I [EC:2.5.1.1 2.5.1.10 2.5.1.29] | K13787 | 1 |
| Bifunctional enzyme Fae/Hps [EC:4.3.-.- 4.1.2.43] | K13812 | 1 |
| 5,10-methenyltetrahydromethanopterin hydrogenase [EC:1.12.98.2] | K13942 | 1 |
| Alcohol dehydrogenase, propanol-preferring [EC:1.1.1.1] | K13953 | 1 |
| Energy-converting hydrogenase A subunit C | K14094 | 1 |
| Energy-converting hydrogenase A subunit E | K14096 | 1 |
| Energy-converting hydrogenase A subunit G | K14098 | 1 |
| Energy-converting hydrogenase A subunit H | K14099 | 1 |
| Energy-converting hydrogenase A subunit J | K14101 | 1 |
| Energy-converting hydrogenase A subunit M | K14104 | 1 |
| Energy-converting hydrogenase A subunit N | K14105 | 1 |
| Energy-converting hydrogenase A subunit O | K14106 | 1 |
| Energy-converting hydrogenase A subunit P | K14107 | 1 |
| Energy-converting hydrogenase A subunit Q | K14108 | 1 |
| Energy-converting hydrogenase A subunit R | K14109 | 1 |
| Energy-converting hydrogenase B subunit A | K14110 | 1 |
| Energy-converting hydrogenase B subunit F | K14115 | 1 |
| Energy-converting hydrogenase B subunit K | K14120 | 1 |
| Energy-converting hydrogenase B subunit L | K14121 | 1 |
| Energy-converting hydrogenase B subunit M | K14122 | 1 |
| Energy-converting hydrogenase B subunit N | K14123 | 1 |
| F420-non-reducing hydrogenase subunit A [EC:1.12.99.-] | K14126 | 1 |
| F420-non-reducing hydrogenase iron-sulfur subunit D [EC:1.12.99.-] | K14127 | 1 |
| F420-non-reducing hydrogenase subunit G [EC:1.12.99.-] | K14128 | 1 |
| Archaea Euryarchaeota | *Methanobrevibacter* | 1 |
| Archaea Euryarchaeota | *Methanosphaera* | 1 |
| Archaea Euryarchaeota | *Methanothermus* | 1 |
| Bacteria Bacteroidetes | *Myroides* | 1 |
| Bacteria Firmicutes | *Sarcina* | 1 |
| Bacteria Firmicutes | *Syntrophomonas* | 1 |
| Bacteria Bacteroidetes | *Bacteroides* | 2 |
| Bacteria γ-Proteobacteria | Candidatus *Hamiltonella* | 2 |
| Bacteria Bacteroidetes | *Flavisolibacter* | 2 |
| malate dehydrogenase [EC:1.1.1.37] | K00024 | 2 |
| fructuronate reductase [EC:1.1.1.57] | K00040 | 2 |
| tagaturonate reductase [EC:1.1.1.58] | K00041 | 2 |
| Ketol-acid reductoisomerase [EC:1.1.1.86] | K00053 | 2 |
| D-3-phosphoglycerate dehydrogenase [EC:1.1.1.95] | K00058 | 2 |
| 3-oxoacyl-[acyl-carrier protein] reductase [EC:1.1.1.100] | K00059 | 2 |
| Aspartate-semialdehyde dehydrogenase [EC:1.2.1.11] | K00133 | 2 |
| glyceraldehyde 3-phosphate dehydrogenase [EC:1.2.1.12] | K00134 | 2 |
| 2-oxoglutarate ferredoxin oxidoreductase subunit alpha [EC:1.2.7.3] | K00174 | 2 |
| 2-oxoglutarate ferredoxin oxidoreductase subunit beta [EC:1.2.7.3] | K00175 | 2 |
| 2-oxoglutarate ferredoxin oxidoreductase subunit delta [EC:1.2.7.3] | K00176 | 2 |
| 2-oxoglutarate ferredoxin oxidoreductase subunit gamma [EC:1.2.7.3] | K00177 | 2 |
| succinate dehydrogenase flavoprotein subunit [EC:1.3.99.1] | K00239 | 2 |
| succinate dehydrogenase iron-sulfur protein [EC:1.3.99.1] | K00240 | 2 |
| succinate dehydrogenase cytochrome b-556 subunit | K00241 | 2 |
| glutamate dehydrogenase (NADP+) [EC:1.4.1.4] | K00262 | 2 |
| glutamate synthase (NADPH/NADH) large chain [EC:1.4.1.13 1.4.1.14] | K00265 | 2 |
| glutamate synthase (NADPH/NADH) small chain [EC:1.4.1.13 1.4.1.14] | K00266 | 2 |
| glycine dehydrogenase subunit 2 [EC:1.4.4.2] | K00283 | 2 |
| saccharopine dehydrogenase (NAD+, L-lysine forming) [EC:1.5.1.7] | K00290 | 2 |
| NADH dehydrogenase I subunit A [EC:1.6.5.3] | K00330 | 2 |
| NADH dehydrogenase I subunit I [EC:1.6.5.3] | K00338 | 2 |
| NADH dehydrogenase I subunit L [EC:1.6.5.3] | K00341 | 2 |
| NADH dehydrogenase I subunit M [EC:1.6.5.3] | K00342 | 2 |
| Na+-transporting NADH:ubiquinone oxidoreductase subunit A [EC:1.6.5.-] | K00346 | 2 |
| Na+-transporting NADH:ubiquinone oxidoreductase subunit B [EC:1.6.5.-] | K00347 | 2 |
| Na+-transporting NADH:ubiquinone oxidoreductase subunit C [EC:1.6.5.-] | K00348 | 2 |
| Na+-transporting NADH:ubiquinone oxidoreductase subunit D [EC:1.6.5.-] | K00349 | 2 |
| Na+-transporting NADH:ubiquinone oxidoreductase subunit E [EC:1.6.5.-] | K00350 | 2 |
| Na+-transporting NADH:ubiquinone oxidoreductase subunit F [EC:1.6.5.-] | K00351 | 2 |
| glycine hydroxymethyltransferase [EC:2.1.2.1] | K00600 | 2 |
| transketolase [EC:2.2.1.1] | K00615 | 2 |
| phosphate acetyltransferase [EC:2.3.1.8] | K00625 | 2 |
| fatty-acyl-CoA synthase [EC:6.2.1.-] | K00666 | 2 |
| UDP-N-acetylglucosamine acyltransferase [EC:2.3.1.129] | K00677 | 2 |
| starch phosphorylase [EC:2.4.1.1] | K00688 | 2 |
| aspartate aminotransferase [EC:2.6.1.1] | K00811 | 2 |
| branched-chain amino acid aminotransferase [EC:2.6.1.42] | K00826 | 2 |
| phosphoserine aminotransferase [EC:2.6.1.52] | K00831 | 2 |
| galactokinase [EC:2.7.1.6] | K00849 | 2 |
| xylulokinase [EC:2.7.1.17] | K00854 | 2 |
| 2-dehydro-3-deoxygluconokinase [EC:2.7.1.45] | K00874 | 2 |
| pyrophosphate--fructose-6-phosphate 1-phosphotransferase [EC:2.7.1.90] | K00895 | 2 |
| acetate kinase [EC:2.7.2.1] | K00925 | 2 |
| phosphoglycerate kinase [EC:2.7.2.3] | K00927 | 2 |
| polyribonucleotide nucleotidyltransferase [EC:2.7.7.8] | K00962 | 2 |
| pyruvate,orthophosphate dikinase [EC:2.7.9.1] | K01006 | 2 |
| glutaminase [EC:3.5.1.2] | K01425 | 2 |
| methylenetetrahydrofolate dehydrogenase (NADP+) / methenyltetrahydrofolate cyclohydrolase [EC:1.5.1.5 3.5.4.9] | K01491 | 2 |
| inorganic pyrophosphatase [EC:3.6.1.1] | K01507 | 2 |
| oxaloacetate decarboxylase, beta subunit [EC:4.1.1.3] | K01572 | 2 |
| phosphoenolpyruvate carboxykinase (ATP) [EC:4.1.1.49] | K01610 | 2 |
| fructose-bisphosphate aldolase, class II [EC:4.1.2.13] | K01624 | 2 |
| 2-dehydro-3-deoxyphosphogluconate aldolase / 4-hydroxy-2-oxoglutarate aldolase [EC:4.1.2.14 4.1.3.16] | K01625 | 2 |
| 2-isopropylmalate synthase [EC:2.3.3.13] | K01649 | 2 |
| fumarate hydratase, class I [EC:4.2.1.2] | K01676 | 2 |
| mannonate dehydratase [EC:4.2.1.8] | K01686 | 2 |
| dTDP-glucose 4,6-dehydratase [EC:4.2.1.46] | K01710 | 2 |
| cysteine synthase A [EC:2.5.1.47] | K01738 | 2 |
| L-ribulose-5-phosphate 4-epimerase [EC:5.1.3.4] | K01786 | 2 |
| triosephosphate isomerase (TIM) [EC:5.3.1.1] | K01803 | 2 |
| L-arabinose isomerase [EC:5.3.1.4] | K01804 | 2 |
| ribose 5-phosphate isomerase B [EC:5.3.1.6] | K01808 | 2 |
| glucuronate isomerase [EC:5.3.1.12] | K01812 | 2 |
| phosphoglycerate mutase [EC:5.4.2.1] | K01834 | 2 |
| phosphomannomutase [EC:5.4.2.8] | K01840 | 2 |
| methylmalonyl-CoA mutase [EC:5.4.99.2] | K01847 | 2 |
| tyrosyl-tRNA synthetase [EC:6.1.1.1] | K01866 | 2 |
| threonyl-tRNA synthetase [EC:6.1.1.3] | K01868 | 2 |
| leucyl-tRNA synthetase [EC:6.1.1.4] | K01869 | 2 |
| alanyl-tRNA synthetase [EC:6.1.1.7] | K01872 | 2 |
| valyl-tRNA synthetase [EC:6.1.1.9] | K01873 | 2 |
| methionyl-tRNA synthetase [EC:6.1.1.10] | K01874 | 2 |
| seryl-tRNA synthetase [EC:6.1.1.11] | K01875 | 2 |
| glycyl-tRNA synthetase [EC:6.1.1.14] | K01880 | 2 |
| prolyl-tRNA synthetase [EC:6.1.1.15] | K01881 | 2 |
| cysteinyl-tRNA synthetase [EC:6.1.1.16] | K01883 | 2 |
| arginyl-tRNA synthetase [EC:6.1.1.19] | K01887 | 2 |
| phenylalanyl-tRNA synthetase alpha chain [EC:6.1.1.20] | K01889 | 2 |
| asparaginyl-tRNA synthetase [EC:6.1.1.22] | K01893 | 2 |
| acetyl-CoA synthetase [EC:6.2.1.1] | K01895 | 2 |
| phosphoribosylaminoimidazole-succinocarboxamide synthase [EC:6.3.2.6] | K01923 | 2 |
| UDP-N-acetylmuramoylalanine--D-glutamate ligase [EC:6.3.2.9] | K01925 | 2 |
| argininosuccinate synthase [EC:6.3.4.5] | K01940 | 2 |
| phosphoribosylformylglycinamidine synthase [EC:6.3.5.3] | K01952 | 2 |
| asparagine synthase (glutamine-hydrolysing) [EC:6.3.5.4] | K01953 | 2 |
| 5S ribosomal RNA | K01985 | 2 |
| putative ABC transport system ATP-binding protein | K02003 | 2 |
| HlyD family secretion protein | K02005 | 2 |
| acyl carrier protein | K02078 | 2 |
| F-type H+-transporting ATPase subunit c [EC:3.6.3.14] | K02110 | 2 |
| F-type H+-transporting ATPase subunit alpha [EC:3.6.3.14] | K02111 | 2 |
| F-type H+-transporting ATPase subunit beta [EC:3.6.3.14] | K02112 | 2 |
| sirohydrochlorin cobaltochelatase [EC:4.99.1.3] | K02190 | 2 |
| DNA polymerase IV [EC:2.7.7.7] | K02346 | 2 |
| elongation factor EF-G [EC:3.6.5.3] | K02355 | 2 |
| elongation factor EF-P | K02356 | 2 |
| elongation factor EF-Ts | K02357 | 2 |
| elongation factor EF-Tu [EC:3.6.5.3] | K02358 | 2 |
| GDP-L-fucose synthase [EC:1.1.1.271] | K02377 | 2 |
| DNA gyrase subunit A [EC:5.99.1.3] | K02469 | 2 |
| DNA gyrase subunit B [EC:5.99.1.3] | K02470 | 2 |
| translation initiation factor IF-1 | K02518 | 2 |
| translation initiation factor IF-2 | K02519 | 2 |
| translation initiation factor IF-3 | K02520 | 2 |
| 3-deoxy-D-manno-octulosonic-acid transferase [EC:2.-.-.-] | K02527 | 2 |
| N utilization substance protein A | K02600 | 2 |
| peptide chain release factor RF-3 | K02837 | 2 |
| large subunit ribosomal protein L1 | K02863 | 2 |
| large subunit ribosomal protein L11 | K02867 | 2 |
| large subunit ribosomal protein L14 | K02874 | 2 |
| large subunit ribosomal protein L15 | K02876 | 2 |
| large subunit ribosomal protein L16 | K02878 | 2 |
| large subunit ribosomal protein L17 | K02879 | 2 |
| large subunit ribosomal protein L18 | K02881 | 2 |
| large subunit ribosomal protein L19 | K02884 | 2 |
| large subunit ribosomal protein L2 | K02886 | 2 |
| large subunit ribosomal protein L20 | K02887 | 2 |
| large subunit ribosomal protein L21 | K02888 | 2 |
| large subunit ribosomal protein L23 | K02892 | 2 |
| large subunit ribosomal protein L27 | K02899 | 2 |
| large subunit ribosomal protein L28 | K02902 | 2 |
| large subunit ribosomal protein L3 | K02906 | 2 |
| large subunit ribosomal protein L31 | K02909 | 2 |
| large subunit ribosomal protein L32 | K02911 | 2 |
| large subunit ribosomal protein L33 | K02913 | 2 |
| large subunit ribosomal protein L34 | K02914 | 2 |
| large subunit ribosomal protein L35 | K02916 | 2 |
| large subunit ribosomal protein L36 | K02919 | 2 |
| large subunit ribosomal protein L4 | K02926 | 2 |
| large subunit ribosomal protein L5 | K02931 | 2 |
| large subunit ribosomal protein L6 | K02933 | 2 |
| large subunit ribosomal protein L7/L12 | K02935 | 2 |
| large subunit ribosomal protein L9 | K02939 | 2 |
| small subunit ribosomal protein S1 | K02945 | 2 |
| small subunit ribosomal protein S10 | K02946 | 2 |
| small subunit ribosomal protein S11 | K02948 | 2 |
| small subunit ribosomal protein S12 | K02950 | 2 |
| small subunit ribosomal protein S13 | K02952 | 2 |
| small subunit ribosomal protein S14 | K02954 | 2 |
| small subunit ribosomal protein S16 | K02959 | 2 |
| small subunit ribosomal protein S17 | K02961 | 2 |
| small subunit ribosomal protein S18 | K02963 | 2 |
| small subunit ribosomal protein S19 | K02965 | 2 |
| small subunit ribosomal protein S2 | K02967 | 2 |
| small subunit ribosomal protein S20 | K02968 | 2 |
| small subunit ribosomal protein S21 | K02970 | 2 |
| small subunit ribosomal protein S3 | K02982 | 2 |
| small subunit ribosomal protein S4 | K02986 | 2 |
| small subunit ribosomal protein S5 | K02988 | 2 |
| small subunit ribosomal protein S6 | K02990 | 2 |
| small subunit ribosomal protein S7 | K02992 | 2 |
| small subunit ribosomal protein S8 | K02994 | 2 |
| DNA-directed RNA polymerase subunit alpha [EC:2.7.7.6] | K03040 | 2 |
| DNA-directed RNA polymerase subunit beta [EC:2.7.7.6] | K03043 | 2 |
| DNA-directed RNA polymerase subunit betaprime [EC:2.7.7.6] | K03046 | 2 |
| preprotein translocase subunit SecA | K03070 | 2 |
| preprotein translocase subunit SecY | K03076 | 2 |
| signal recognition particle subunit SRP54 | K03106 | 2 |
| UDP-2,3-diacylglucosamine hydrolase [EC:3.6.1.-] | K03269 | 2 |
| proton-dependent oligopeptide transporter, POT family | K03305 | 2 |
| solute:Na+ symporter, SSS family | K03307 | 2 |
| electron transfer flavoprotein beta subunit | K03521 | 2 |
| electron transfer flavoprotein alpha subunit | K03522 | 2 |
| DNA-binding protein HU-beta | K03530 | 2 |
| cell division protein FtsZ | K03531 | 2 |
| biopolymer transport protein ExbB | K03561 | 2 |
| rod shape-determining protein MreB and related proteins | K03569 | 2 |
| GTP-binding protein Era | K03595 | 2 |
| GTP-binding protein LepA | K03596 | 2 |
| electron transport complex protein RnfA | K03617 | 2 |
| ATP-dependent Clp protease ATP-binding subunit ClpB | K03695 | 2 |
| putative pyruvate-flavodoxin oxidoreductase [EC:1.2.7.-] | K03737 | 2 |
| periplasmic protein TonB | K03832 | 2 |
| molecular chaperone DnaK | K04043 | 2 |
| chaperonin GroEL | K04077 | 2 |
| molecular chaperone HtpG | K04079 | 2 |
| chorismate mutase [EC:5.4.99.5] | K04516 | 2 |
| lysyl-tRNA synthetase, class II [EC:6.1.1.6] | K04567 | 2 |
| nitrogen regulatory protein P-II 1 | K04751 | 2 |
| ribosomal large subunit pseudouridine synthase B [EC:5.4.99.12] | K06178 | 2 |
| uncharacterized protein | K06889 | 2 |
| ribosome-binding ATPase | K06942 | 2 |
| Uncharacterized protein | K06950 | 2 |
| uncharacterized protein | K07133 | 2 |
| uncharacterized protein | K07148 | 2 |
| uncharacterized protein | K07164 | 2 |
| alpha-amylase [EC:3.2.1.1] | K07405 | 2 |
| MFS transporter, ACS family, hexuronate transporter | K08191 | 2 |
| Fe-S cluster assembly ATP-binding protein | K09013 | 2 |
| Fe-S cluster assembly protein SufB | K09014 | 2 |
| uridylate kinase [EC:2.7.4.22] | K09903 | 2 |
| SecD/SecF fusion protein | K12257 | 2 |
| bifunctional aspartokinase / homoserine dehydrogenase 1 [EC:2.7.2.4 1.1.1.3] | K12524 | 2 |
| N-succinyl-L-ornithine transcarbamylase [EC:2.1.3.11] | K13043 | 2 |
| NADH dehydrogenase I subunit C/D [EC:1.6.5.3] | K13378 | 2 |
| HSP20 family protein | K13993 | 2 |
| tRNA Arg | K14219 | 2 |
| tRNA Ile | K14227 | 2 |
| tRNA Leu | K14228 | 2 |
| tRNA Met | K14230 | 2 |
| tRNA Ser | K14233 | 2 |
| tRNA Thr | K14234 | 2 |
| tRNA Trp | K14235 | 2 |
| Bacteria γ-Proteobacteria | *Marinomonas* | 2 |
| Bacteria Bacteroidetes | *Pedobacter* | 2 |
| Bacteria Bacteroidetes | *Petrimonas* | 2 |
| Bacteria Bacteroidetes | *Prevotella* | 2 |
| Bacteria Cyanobacteria | *Acaryochloris* | 3 |
| Bacteria α-Proteobacteria | *Acetobacter* | 3 |
| Bacteria Actinobacteria | *Acidimicrobium* | 3 |
| Bacteria γ-Proteobacteria | *Actinobacillus* | 3 |
| Bacteria Actinobacteria | *Actinotignum* | 3 |
| Bacteria Actinobacteria | *Aeromicrobium* | 3 |
| Bacteria γ-Proteobacteria | *Aeromonas* | 3 |
| Bacteria α-Proteobacteria | *Agrobacterium* | 3 |
| Bacteria γ-Proteobacteria | *Alcanivorax* | 3 |
| Bacteria γ-Proteobacteria | *Alkalilimnicola* | 3 |
| Bacteria β-Proteobacteria | *Aromatoleum* | 3 |
| Bacteria α-Proteobacteria | *Asticcacaulis* | 3 |
| Bacteria β-Proteobacteria | *Azoarcus* | 3 |
| Bacteria β-Proteobacteria | *Azospira* | 3 |
| Bacteria δ-Proteobacteria | *Bdellovibrio* | 3 |
| Bacteria α-Proteobacteria | *Beijerinckia* | 3 |
| Bacteria α-Proteobacteria | *Blastochloris* | 3 |
| Bacteria γ-Proteobacteria | *Blastomonas* | 3 |
| Bacteria α-Proteobacteria | Candidatus *Accumulibacter* | 3 |
| Bacteria α-Proteobacteria | Candidatus *Ishikawaella* | 3 |
| Bacteria α-Proteobacteria | Candidatus *Phaeomarinobacter* | 3 |
| Bacteria Proteobacteria | Candidatus *Puniceispirillum* | 3 |
| Bacteria β-Proteobacteria | *Castellaniella* | 3 |
| Bacteria γ-Proteobacteria | *Cellvibrio* | 3 |
| Bacteria α-Proteobacteria | *Chelativorans* | 3 |
| Bacteria α-Proteobacteria | *Chelatococcus* | 3 |
| Bacteria Chlorobi | *Chlorobium* | 3 |
| Bacteria γ-Proteobacteria | *Chromohalobacter* | 3 |
| Bacteria Armatimonadetes | *Chthonomonas* | 3 |
| Bacteria γ-Proteobacteria | *Colwellia* | 3 |
| Bacteria β-Proteobacteria | *Comamonas* | 3 |
| Bacteria γ-Proteobacteria | *Congregibacter* | 3 |
| Bacteria γ-Proteobacteria | *Cronobacter* | 3 |
| Bacteria Actinobacteria | *Curtobacterium* | 3 |
| Bacteria α-Proteobacteria | *Defluviimonas* | 3 |
| Bacteria β-Proteobacteria | *Delftia* | 3 |
| Bacteria Actinobacteria | *Dermabacter* | 3 |
| Bacteria δ-Proteobacteria | *Desulfobacterium* | 3 |
| Bacteria Chrysiogenetes | *Desulfurispirillum* | 3 |
| Bacteria γ-Proteobacteria | *Dickeya* | 3 |
| Bacteria γ-Proteobacteria | *Dokdonella* | 3 |
| Bacteria γ-Proteobacteria | *Dyella* | 3 |
| Bacteria γ-Proteobacteria | *Edwardsiella* | 3 |
| Bacteria γ-Proteobacteria | *Enterobacter* | 3 |
| Bacteria γ-Proteobacteria | *Erwinia* | 3 |
| Bacteria γ-Proteobacteria | *Escherichia* | 3 |
| Bacteria Firmicutes | *Exiguobacterium* | 3 |
| Bacteria γ-Proteobacteria | *Ferrimonas* | 3 |
| Bacteria γ-Proteobacteria | *Frischella* | 3 |
| Bacteria δ-Proteobacteria | *Geoalkalibacter* | 3 |
| Bacteria Firmicutes | *Geobacillus* | 3 |
| Bacteria Actinobacteria | *Gordonia* | 3 |
| Bacteria α-Proteobacteria | *Haematospirillum* | 3 |
| Archaea Euryarchaeota | *Halanaeroarchaeum* | 3 |
| Bacteria γ-Proteobacteria | *Halioglobus* | 3 |
| Bacteria α-Proteobacteria | *Halocynthiibacter* | 3 |
| Archaea Euryarchaeota | *Halogranum* | 3 |
| Archaea Euryarchaeota | *Halolamina* | 3 |
| Bacteria γ-Proteobacteria | *Halomonas* | 3 |
| Archaea Euryarchaeota | *Haloplanus* | 3 |
| Archaea Euryarchaeota | *Halorhabdus* | 3 |
| Bacteria γ-Proteobacteria | *Halorhodospira* | 3 |
| Bacteria γ-Proteobacteria | *Halotalea* | 3 |
| Bacteria γ-Proteobacteria | *Halothiobacillus* | 3 |
| Bacteria β-Proteobacteria | *Herbaspirillum* | 3 |
| Bacteria γ-Proteobacteria | *Histophilus* | 3 |
| Archaea Crenarchaeota | *Ignicoccus* | 3 |
| Bacteria γ-Proteobacteria | *Immundisolibacter* | 3 |
| Bacteria β-Proteobacteria | *Janthinobacterium* | 3 |
| Bacteria Synergistetes | *Jonquetella* | 3 |
| Fumarate hydratase, class II [EC:4.2.1.2] | K01679 | 3 |
| Acetyl-CoA carboxylase, biotin carboxylase subunit [EC:6.4.1.2 6.3.4.14] | K01961 | 3 |
| Iron(III) transport system ATP-binding protein [EC:3.6.3.30] | K02010 | 3 |
| Flagellar biosynthesis protein FlhA | K02400 | 3 |
| RNA polymerase primary sigma factor | K03086 | 3 |
| Type I restriction enzyme M protein [EC:2.1.1.72] | K03427 | 3 |
| Fungi Basidiomycota | *Kalmanozyma* | 3 |
| Bacteria Actinobacteria | Klebsiella | 3 |
| Bacteria γ-Proteobacteria | Kluyvera | 3 |
| Bacteria γ-Proteobacteria | Kosakonia | 3 |
| Bacteria β-Proteobacteria | *Laribacter* | 3 |
| Bacteria γ-Proteobacteria | *Leclercia* | 3 |
| Bacteria α-Proteobacteria | *Leisingera* | 3 |
| Bacteria β-Proteobacteria | *Leptothrix* | 3 |
| Bacteria Actinobacteria | *Luteipulveratus* | 3 |
| Bacteria γ-Proteobacteria | *Marinobacter* | 3 |
| Bacteria α-Proteobacteria | *Mesorhizobium* | 3 |
| Archaea Euryarchaeota | *Methanohalophilus* | 3 |
| Archaea Euryarchaeota | *Methanoregula* | 3 |
| Bacteria α-Proteobacteria | *Methyloceanibacter* | 3 |
| Bacteria γ-Proteobacteria | *Methylomonas* | 3 |
| Bacteria γ-Proteobacteria | *Microbulbifer* | 3 |
| Bacteria β-Proteobacteria | *Mitsuaria* | 3 |
| Bacteria Actinobacteria | *Modestobacter* | 3 |
| Bacteria γ-Proteobacteria | *Moraxella* | 3 |
| Bacteria Firmicutes | *Ndongobacter* | 3 |
| Bacteria β-Proteobacteria | *Neisseria* | 3 |
| Bacteria α-Proteobacteria | *Nitrobacter* | 3 |
| Bacteria γ-Proteobacteria | *Nitrosococcus* | 3 |
| Bacteria β-Proteobacteria | *Nitrosospira* | 3 |
| Bacteria γ-Proteobacteria | *Oceanimonas* | 3 |
| Bacteria β-Proteobacteria | *Pandoraea* | 3 |
| Bacteria γ-Proteobacteria | *Pantoea* | 3 |
| Bacteria β-Proteobacteria | *Paraburkholderia* | 3 |
| Bacteria α-Proteobacteria | *Pelagibacterium* | 3 |
| Bacteria γ-Proteobacteria | *Pluralibacter* | 3 |
| Bacteria γ-Proteobacteria | *Pseudomonas* | 3 |
| Bacteria γ-Proteobacteria | *Pseudoxanthomonas* | 3 |
| Bacteria γ-Proteobacteria | *Rahnella* | 3 |
| Bacteria β-Proteobacteria | *Ralstonia* | 3 |
| Bacteria γ-Proteobacteria | *Raoultella* | 3 |
| Bacteria Actinobacteria | *Rathayibacter* | 3 |
| Bacteria α-Proteobacteria | *Rhizobium* | 3 |
| Bacteria α-Proteobacteria | *Rhodobacter* | 3 |
| Bacteria α-Proteobacteria | *Rhodovulum* | 3 |
| Bacteria γ-Proteobacteria | *Salmonella* | 3 |
| Bacteria Actinobacteria | *Sanguibacter* | 3 |
| Bacteria Actinobacteria | *Scardovia* | 3 |
| Bacteria γ-Proteobacteria | *Sedimenticola* | 3 |
| Bacteria γ-Proteobacteria | *Serratia* | 3 |
| Bacteria γ-Proteobacteria | *Shimwellia* | 3 |
| Bacteria β-Proteobacteria | *Sideroxydans* | 3 |
| Bacteria β-Proteobacteria | *Sodalis* | 3 |
| Bacteria α-Proteobacteria | *Sphingopyxis* | 3 |
| Bacteria γ-Proteobacteria | *Spiribacter* | 3 |
| Bacteria α-Proteobacteria | *Sulfitobacter* | 3 |
| Bacteria β-Proteobacteria | *Sulfuritalea* | 3 |
| Bacteria Firmicutes | *Syntrophobotulus* | 3 |
| Bacteria Acidobacteria | *Terriglobus* | 3 |
| Bacteria β-Proteobacteria | *Thauera* | 3 |
| Archea Crenarchaeota | *Thermofilum* | 3 |
| Bacteria Thermotogae | *Thermotoga* | 3 |
| Bacteria γ-Proteobacteria | *Thioalkalivibrio* | 3 |
| Bacteria γ-Proteobacteria | *Thiocystis* | 3 |
| Bacteria γ-Proteobacteria | *Thioflavicoccus* | 3 |
| Bacteria β-Proteobacteria | *Thiomonas* | 3 |
| Bacteria γ-Proteobacteria | *Thioploca* | 3 |
| Fungi Basidiomycota | *Tilletiaria* | 3 |
| Bacteria γ-Proteobacteria | *Tolumonas* | 3 |
| Bacteria Deinococcus-Thermus | *Truepera* | 3 |
| Bacteria Actinobacteria | *Trueperella* | 3 |
| Bacteria Actinobacteria | *Tsukamurella* | 3 |
| Bacteria γ-Proteobacteria | *Wenzhouxiangella* | 3 |
| Bacteria α-Proteobacteria | *Xanthobacter* | 3 |
| Protist Amoebozoa | *Acanthamoeba* | 4 |
| Archaea Euryarchaeota | *Acidiplasma* | 4 |
| Protist Amoebozoa Mycetozoa | *Acytostelium* | 4 |
| Fungi Basidiomycota | *Agaricus* | 4 |
| Bacteria Cyanobacteria | *Anabaena* | 4 |
| Protist Heterokonta | *Aphanomyces* | 4 |
| Fungi Ascomycota | *Arthrobotrys* | 4 |
| Fungi Ascomycota | *Aspergillus* | 4 |
| Fungi Ascomycota | *Beauveria* | 4 |
| Fungi Ascomycota | *Bipolaris* | 4 |
| Protist Bigyra | *Blastocystis* | 4 |
| Fungi Ascomycota | *Blastomyces* | 4 |
| Fungi Ascomycota | *Botrytis* | 4 |
| Bacteria Cyanobacteria | *Calothrix* | 4 |
| Fungi Ascomycota | *Candida* | 4 |
| Archaea Thaumarchaeaota | Candidatus *Nitrosotenuis* | 4 |
| Fungi Ascomycota | *Chaetomium* | 4 |
| Fungi Ascomycota | *Clavispora* | 4 |
| Fungi Ascomycota | *Coccidioides* | 4 |
| Fungi Ascomycota | *Colletotrichum* | 4 |
| Fungi Ascomycota | *Cordyceps* | 4 |
| Bacteria γ-Proteobacteria | *Coxiella* | 4 |
| Fungi Basidiomycota | *Cryptococcus* | 4 |
| Protist Alveolata Apicomplexa | *Cryptosporidium* | 4 |
| Bacteria Cyanobacteria | *Cylindrospermum* | 4 |
| Fungi Ascomycota | *Dactylellina* | 4 |
| Fungi Ascomycota | *Debaryomyces* | 4 |
| Protist Amoebozoa Mycetozoa | *Dictyostelium* | 4 |
| Fungi Microsporidia | *Encephalitozoon* | 4 |
| Protist Amoebozoa Archamoebae | *Entamoeba* | 4 |
| Fungi Microsporidia | *Enterocytozoon* | 4 |
| Fungi Ascomycota | *Eremothecium* | 4 |
| Fungi Ascomycota | *Eutypa* | 4 |
| Fungi Basidiomycota | *Fomitiporia* | 4 |
| Fungi>Ascomycota | *Fusarium* | 4 |
| Fungi Ascomycota | *Gaeumannomyces* | 4 |
| Protist Alveolata Apicomplexa | *Gregarina* | 4 |
| Fungi Ascomycota | *Grosmannia* | 4 |
| Bacteria ε-Proteobacteria | *Helicobacter* | 4 |
| Fungi Ascomycota | *Histoplasma* | 4 |
| Protist Alveolata Ciliophora | *Ichthyophthirius* | 4 |
| Fungi Ascomycota | *Isaria* | 4 |
| Phosphoribosylformylglycinamidine cyclo-ligase [EC:6.3.3.1] | K01933 | 4 |
| SEC-C motif domain protein | K09858 | 4 |
| Fungi Ascomycota | *Kazachstania* | 4 |
| Fungi Ascomycota | *Kluyveromyces* | 4 |
| Fungi Ascomycota | *Komagataella* | 4 |
| Fungi Basidiomycota | *Kwoniella* | 4 |
| Fungi Ascomycota | *Leptosphaeria* | 4 |
| Fungi Ascomycota | *Lodderomyces* | 4 |
| Fungi Ascomycota | *Magnaporthe* | 4 |
| Fungi Ascomycota | *Marssonina* | 4 |
| Fungi Basidiomycota | *Melampsora* | 4 |
| Fungi Ascomycota | *Metarhizium* | 4 |
| Archaea Euryarchaeota | *Methanocaldococcus* | 4 |
| Archaea Euryarchaeota | *Methanococcus* | 4 |
| Archaea Euryarchaeota | *Methanosarcina* | 4 |
| Archaea Euryarchaeota | *Methanothermococcus* | 4 |
| Fungi Ascomycota | *Meyerozyma* | 4 |
| Fungi Ascomycota | *Microsporum* | 4 |
| Fungi Ascomycota | *Millerozyma* | 4 |
| Fungi Microsporida | *Mitosporidium* | 4 |
| Fungi Basidiomycota | *Mixia* | 4 |
| Fungi Basidiomycota | *Moniliophthora* | 4 |
| Protist Percolozoa | *Naegleria* | 4 |
| Fungi Ascomycota | *Nakaseomyces* | 4 |
| Fungi Ascomycota | *Nannizzia* | 4 |
| Fungi Ascomycota | *Naumovozyma* | 4 |
| Fungi Microsporidia | *Nematocida* | 4 |
| Protist Alveolata Apicomplexa | *Neospora* | 4 |
| Fungi Ascomycota | *Neurospora* | 4 |
| Bacteria Cyanobacteria | *Nodularia* | 4 |
| Fungi Microsporidia | *Nosema* | 4 |
| Bacteria Cyanobacteria | *Nostoc* | 4 |
| Fungi Ascomycota | *Paracoccidioides* | 4 |
| Protist Ciliophora | *Paramecium* | 4 |
| Fungi Ascomycota | *Penicillium* | 4 |
| Protist Alveolata Perkinsea | *Perkinsus* | 4 |
| Fungi Ascomycota | *Phaeoacremonium* | 4 |
| Protist Heterokonta | *Phaeodactylum* | 4 |
| Fungi Mucoromycota | *Phycomyces* | 4 |
| Protist Heterokonta | *Phytophthora* | 4 |
| Fungi Ascomycota | *Pichia* | 4 |
| Protist Alveolata Apicomplexa | *Plasmodium* | 4 |
| Fungi Ascomycota | *Pneumocystis* | 4 |
| Fungi Ascomycota | *Pochonia* | 4 |
| Fungi Ascomycota | *Podospora* | 4 |
| Fungi Basidiomycota | *Puccinia* | 4 |
| Fungi Ascomycota | *Rasamsonia* | 4 |
| Fungi Ascomycota | *Rhinocladiella* | 4 |
| Bacteria Cyanobacteria | *Rivularia* | 4 |
| Fungi Ascomycota | *Saccharomyces* | 4 |
| Protist Stramenopiles | *Saprolegnia* | 4 |
| Fungi Ascomycota | *Scedosporium* | 4 |
| Fungi Ascomycota | *Scheffersomyces* | 4 |
| Fungi Ascomycota | *Schizosaccharomyces* | 4 |
| Fungi Ascomycota | *Sclerotinia* | 4 |
| Fungi Ascomycota | *Setosphaeria* | 4 |
| Fungi Ascomycota | *Spathaspora* | 4 |
| Fungi Ascomycota | *Sphaerulina* | 4 |
| Fungi Chytridiomycota | *Spizellomyces* | 4 |
| Fungi Ascomycota | *Sporothrix* | 4 |
| Fungi Ascomycota | *Sugiyamaella* | 4 |
| Fungi Ascomycota | *Talaromyces* | 4 |
| Protist Alveolata Ciliophora | *Tetrahymena* | 4 |
| Fungi Ascomycota | *Tetrapisispora* | 4 |
| Protist Alveolata Apicomplexa | *Theileria* | 4 |
| Fungi Ascomycota | *Thermothelomyces* | 4 |
| Fungi Ascomycota | *Thielavia* | 4 |
| Fungi Ascomycota | *Torulaspora* | 4 |
| Protist Alveolata Apicomplexa | *Toxoplasma* | 4 |
| Fungi Ascomycota | *Trichoderma* | 4 |
| Protist Parabasalia Trichomonadida | *Trichomonas* | 4 |
| Fungi Ascomycota | *Trichophyton* | 4 |
| Bacteria Cyanobacteria | *Trichormus* | 4 |
| Protist Euglenozoa Kinetoplastida | *Trypanosoma* | 4 |
| Fungi Basidiomycota | *Tsuchiyaea* | 4 |
| Bacteria Actinobacteria | *Tuber* | 4 |
| Fungi Ascomycota | *Uncinocarpus* | 4 |
| Fungi Ascomycota | *Vanderwaltozyma* | 4 |
| Fungi Microsporidia | *Vavraia* | 4 |
| Fungi Ascomycota | *Verruconis* | 4 |
| Fungi Basidiomycota | *Wallemia* | 4 |
| Fungi Ascomycota | *Wickerhamomyces* | 4 |
| Bacteria γ-Proteobacteria | *Xanthomonas* | 4 |
| Fungi Ascomycota | *Xylona* | 4 |
| Fungi Ascomycota | *Yamadazyma* | 4 |
| Fungi Ascomycota | *Zygosaccharomyces* | 4 |
| Bacteria Actinobacteria | *Acidipropionibacterium* | 5 |
| Bacteria Proteobacteria | *Acidithiobacillus* | 5 |
| Bacteria Actinobacteria | *Actinoalloteichus* | 5 |
| Bacteria Actinobacteria | *Actinomyces* | 5 |
| Bacteria Actinobacteria | *Actinoplanes* | 5 |
| Bacteria Actinobacteria | *Adlercreutzia* | 5 |
| Bacteria β-Proteobacteria | *Alicycliphilus* | 5 |
| Bacteria Actinobacteria | *Alloactinosynnema* | 5 |
| Bacteria Actinobacteria | *Amycolatopsis* | 5 |
| Bacteria Actinobacteria | *Arsenicicoccus* | 5 |
| Bacteria α-Proteobacteria | *Aureimonas* | 5 |
| Bacteria α-Proteobacteria | *Bosea* | 5 |
| Bacteria Actinobacteria | *Brachybacterium* | 5 |
| Bacteria α-Proteobacteria | *Citromicrobium* | 5 |
| Bacteria Synergistetes | *Cloacibacillus* | 5 |
| Bacteria δ-Proteobacteria | *Corallococcus* | 5 |
| Bacteria Deinococcus-Thermus | *Deinococcus* | 5 |
| Bacteria Actinobacteria | *Dermacoccus* | 5 |
| Bacteria δ-Proteobacteria | *Desulfomicrobium* | 5 |
| Bacteria δ-Proteobacteria | *Desulfovibrio* | 5 |
| Bacteria δ-Proteobacteria | *Desulfuromonas* | 5 |
| Bacteria Actinobacteria | *Eggerthella* | 5 |
| Bacteria α-Proteobacteria | *Erythrobacter* | 5 |
| Bacteria Actinobacteria | *Frankia* | 5 |
| Bacteria Actinobacteria | *Geodermatophilus* | 5 |
| Bacteria α-Proteobacteria | *Gluconobacter* | 5 |
| Bacteria α-Proteobacteria | *Granulibacter* | 5 |
| Archaea Euryarchaeota | *Haloarcula* | 5 |
| Archaea Euryarchaeota | *Halopiger* | 5 |
| Archaea Euryarchaeota | *Halorubrum* | 5 |
| Bacteria α-Proteobacteria | *Hoeflea* | 5 |
| Bacteria α-Proteobacteria | *Hyphomicrobium* | 5 |
| Bacteria Actinobacteria | *Intrasporangium* | 5 |
| Bacteria Actinobacteria | *Isoptericola* | 5 |
| Bacteria α-Proteobacteria | *Jannaschia* | 5 |
| Bacteria Actinobacteria | *Kibdelosporangium* | 5 |
| Bacteria Actinobacteria | *Kitasatospora* | 5 |
| Bacteria Actinobacteria | *Kocuria* | 5 |
| Bacteria α-Proteobacteria | *Komagataeibacter* | 5 |
| Bacteria Actinobacteria | *Kribbella* | 5 |
| Bacteria Actinobacteria | *Lawsonella* | 5 |
| Bacteria Actinobacteria | *Lentzea* | 5 |
| Bacteria Deinococcus-Thermus | *Marinithermus* | 5 |
| Archaea Euryarchaeota | *Methanocella* | 5 |
| Archaea Euryarchaeota | *Methanoculleus* | 5 |
| Archaea Euryarchaeota | *Methanosaeta* | 5 |
| Bacteria α-Proteobacteria | *Methylobacterium* | 5 |
| Bacteria α-Proteobacteria | *Methylocystis* | 5 |
| Bacteria Actinobacteria | *Micrococcus* | 5 |
| Bacteria Actinobacteria | *Microlunatus* | 5 |
| Bacteria Actinobacteria | *Micromonospora* | 5 |
| Bacteria Actinobacteria | *Mobiluncus* | 5 |
| Bacteria δ-Proteobacteria | *Myxococcus* | 5 |
| Bacteria Actinobacteria | *Nocardia* | 5 |
| Bacteria Actinobacteria | *Nocardioides* | 5 |
| Bacteria α-Proteobacteria | *Novosphingobium* | 5 |
| Bacteria Deinococcus-Thermus | *Oceanithermus* | 5 |
| Bacteria β-Proteobacteria | *Ottowia* | 5 |
| Bacteria δ-Proteobacteria | *Pelobacter* | 5 |
| Bacteria α-Proteobacteria | *Phaeobacter* | 5 |
| Bacteria Actinobacteria | *Pimelobacter* | 5 |
| Bacteria Actinobacteria | *Pseudopropionibacterium* | 5 |
| Bacteria β-Proteobacteria | *Roseateles* | 5 |
| Bacteria α-Proteobacteria | *Roseibacterium* | 5 |
| Bacteria α-Proteobacteria | *Roseobacter* | 5 |
| Bacteria α-Proteobacteria | *Roseomonas* | 5 |
| Bacteria Firmicutes | *Ruminiclostridium* | 5 |
| Bacteria Actinobacteria | *Salinispora* | 5 |
| Fungi Basidiomycota | *Schizophyllum* | 5 |
| Bacteria Actinobacteria | *Slackia* | 5 |
| Bacteria Actinobacteria | *Stackebrandtia* | 5 |
| Bacteria δ-Proteobacteria | *Stigmatella* | 5 |
| Bacteria Actinobacteria | *Streptomyces* | 5 |
| Bacteria Actinobacteria | *Streptosporangium* | 5 |
| Bacteria Firmicutes | *Symbiobacterium* | 5 |
| Bacteria δ-Proteobacteria | *Syntrophobacter* | 5 |
| Bacteria δ-Proteobacteria | *Syntrophus* | 5 |
| Bacteria Firmicutes | *Thermaerobacter* | 5 |
| Bacteria Firmicutes | *Thermobacillus* | 5 |
| Bacteria Actinobacteria | *Thermomonospora* | 5 |
| Bacteria Firmicutes | *Thermosediminibacter* | 5 |
| Bacteria β-Proteobacteria | *Verminephrobacter* | 5 |
| Bacteria α-Proteobacteria | *Yangia* | 5 |
| Bacteria Actinobacteria | *Bifidobacterium* | 6 |
| L-lactate dehydrogenase [EC:1.1.1.27] | K00016 | 6 |
| 6-phosphogluconate dehydrogenase [EC:1.1.1.44] | K00033 | 6 |
| Glucose-6-phosphate 1-dehydrogenase [EC:1.1.1.49] | K00036 | 6 |
| Lactaldehyde reductase [EC:1.1.1.77] | K00048 | 6 |
| NAD(P) transhydrogenase subunit alpha [EC:1.6.1.2] | K00324 | 6 |
| NAD(P) transhydrogenase subunit beta [EC:1.6.1.2] | K00325 | 6 |
| Transaldolase [EC:2.2.1.2] | K00616 | 6 |
| 2,3,4,5-tetrahydropyridine-2-carboxylate N-succinyltransferase [EC:2.3.1.117] | K00674 | 6 |
| Sucrose phosphorylase [EC:2.4.1.7] | K00690 | 6 |
| Nicotinate phosphoribosyltransferase [EC:2.4.2.11] | K00763 | 6 |
| Homoserine kinase [EC:2.7.1.39] | K00872 | 6 |
| Pyruvate kinase [EC:2.7.1.40] | K00873 | 6 |
| Polyphosphate kinase [EC:2.7.4.1] | K00937 | 6 |
| UDPglucose--hexose-1-phosphate uridylyltransferase [EC:2.7.7.12] | K00965 | 6 |
| Glutamate-ammonia-ligase adenylyltransferase [EC:2.7.7.42] | K00982 | 6 |
| Protein phosphatase [EC:3.1.3.16] | K01090 | 6 |
| alpha-glucosidase [EC:3.2.1.20] | K01187 | 6 |
| Ovochymase [EC:3.4.21.-] | K01362 | 6 |
| Putative membrane protein | K01421 | 6 |
| Succinyl-diaminopimelate desuccinylase [EC:3.5.1.18] | K01439 | 6 |
| dCTP deaminase [EC:3.5.4.13] | K01494 | 6 |
| No such data | K01552 | 6 |
| 5-(carboxyamino)imidazole ribonucleotide synthase [EC:6.3.4.18] | K01589 | 6 |
| Phosphoenolpyruvate carboxylase [EC:4.1.1.31] | K01595 | 6 |
| 3-deoxy-7-phosphoheptulonate synthase [EC:2.5.1.54] | K01626 | 6 |
| Fructose-6-phosphate phosphoketolase [EC:4.1.2.22] | K01632 | 6 |
| L-serine dehydratase [EC:4.3.1.17] | K01752 | 6 |
| Threonine dehydratase [EC:4.3.1.19] | K01754 | 6 |
| Phosphoglucomutase [EC:5.4.2.2] | K01835 | 6 |
| UDP-galactopyranose mutase [EC:5.4.99.9] | K01854 | 6 |
| Glutamate--cysteine ligase [EC:6.3.2.2] | K01919 | 6 |
| Branched-chain amino acid transport system ATP-binding protein | K01995 | 6 |
| Branched-chain amino acid transport system ATP-binding protein | K01996 | 6 |
| Branched-chain amino acid transport system permease protein | K01997 | 6 |
| Branched-chain amino acid transport system permease protein | K01998 | 6 |
| Branched-chain amino acid transport system substrate-binding protein | K01999 | 6 |
| Multiple sugar transport system permease protein | K02025 | 6 |
| Multiple sugar transport system permease protein | K02026 | 6 |
| Polar amino acid transport system permease protein | K02029 | 6 |
| Phosphate transport system ATP-binding protein [EC:3.6.3.27] | K02036 | 6 |
| Phosphate transport system permease protein | K02037 | 6 |
| Phosphate transport system permease protein | K02038 | 6 |
| Glycogen operon protein GlgX [EC:3.2.1.-] | K02438 | 6 |
| Two-component system, OmpR family, response regulator | K02483 | 6 |
| Two-component system, OmpR family, sensor kinase [EC:2.7.13.3] | K02484 | 6 |
| DNA-directed RNA polymerase subunit omega [EC:2.7.7.6] | K03060 | 6 |
| DNA topoisomerase I [EC:5.99.1.2] | K03168 | 6 |
| RNA methyltransferase, TrmH family, group 2 [EC:2.1.1.-] | K03216 | 6 |
| Amino acid transporter, AAT family | K03293 | 6 |
| Basic amino acid/polyamine antiporter, APA family | K03294 | 6 |
| Phosphoglucosamine mutase [EC:5.4.2.10] | K03431 | 6 |
| KUP system potassium uptake protein | K03549 | 6 |
| ATP-dependent helicase HrpA [EC:3.6.4.13] | K03578 | 6 |
| ATP-dependent RNA helicase HelY [EC:3.6.4.-] | K03727 | 6 |
| Membrane-associated protein | K03975 | 6 |
| Acetaldehyde dehydrogenase / alcohol dehydrogenase [EC:1.2.1.10 1.1.1.1] | K04072 | 6 |
| Carbon starvation protein | K06200 | 6 |
| MFS transporter, UMF1 family | K06902 | 6 |
| UPF0042 nucleotide-binding protein | K06958 | 6 |
| Uncharacterized protein | K07009 | 6 |
| CRISPR-associated endonuclease/helicase Cas3 [EC:3.1.-.- 3.6.4.-] | K07012 | 6 |
| Uncharacterized protein | K07088 | 6 |
| S-ribosylhomocysteine lyase [EC:4.4.1.21] | K07173 | 6 |
| Poly-gamma-glutamate synthesis protein (capsule biosynthesis protein) | K07282 | 6 |
| Dipeptidase [EC:3.4.-.-] | K08659 | 6 |
| Serine/threonine protein kinase, bacterial [EC:2.7.11.1] | K08884 | 6 |
| Hypothetical protein | K09118 | 6 |
| Hypothetical protein | K09157 | 6 |
| Hypothetical protein | K09762 | 6 |
| Glutamate transport system ATP-binding protein [EC:3.6.3.-] | K10008 | 6 |
| Maltose/maltodextrin transport system substrate-binding protein | K10108 | 6 |
| Maltose/maltodextrin transport system permease protein | K10109 | 6 |
| Maltose/maltodextrin transport system permease protein | K10110 | 6 |
| Multiple sugar transport system permease protein | K10118 | 6 |
| Multiple sugar transport system permease protein | K10119 | 6 |
| Cellobiose transport system ATP-binding protein | K10243 | 6 |
| Myosin-crossreactive antigen | K10254 | 6 |
| Acetyl-/propionyl-CoA carboxylase, biotin carboxylase, biotin carboxyl carrier protein [EC:6.3.4.14] | K11263 | 6 |
| Aspartate aminotransferase [EC:2.6.1.1] | K11358 | 6 |
| Fatty acid synthase, bacteria type [EC:2.3.1.-] | K11533 | 6 |
| Ribonuclease J [EC:3.1.-.-] | K12574 | 6 |
| Alanine-synthesizing transaminase [EC:2.6.1.66 2.6.1.2] | K14260 | 6 |
| Bacteria β-Proteobacteria | *Achromobacter* | 7 |
| Bacteria γ-Proteobacteria | *Acidihalobacter* | 7 |
| Bacteria α-Proteobacteria | *Acidiphilium* | 7 |
| Bacteria Actinobacteria | *Acidothermus* | 7 |
| Bacteria β-Proteobacteria | *Acidovorax* | 7 |
| Bacteria Firmicutes | *Alicyclobacillus* | 7 |
| Bacteria γ-Proteobacteria | *Allochromatium* | 7 |
| Bacteria α-Proteobacteria | *Aminobacter* | 7 |
| Bacteria Synergistetes | *Aminomonas* | 7 |
| Bacteria Actinobacteria | *Arthrobacter* | 7 |
| Bacteria α-Proteobacteria | *Azorhizobium* | 7 |
| Bacteria Actinobacteria | *Blastococcus* | 7 |
| Bacteria β-Proteobacteria | *Bordetella* | 7 |
| Bacteria α-Proteobacteria | *Brevundimonas* | 7 |
| Bacteria β-Proteobacteria | *Burkholderia* | 7 |
| Bacteria β-Proteobacteria | *Chromobacterium* | 7 |
| Bacteria Actinobacteria | *Clavibacter* | 7 |
| Bacteria Actinobacteria | *Coriobacterium* | 7 |
| Bacteria Actinobacteria | *Corynebacterium* | 7 |
| Bacteria α-Proteobacteria | *Croceicoccus* | 7 |
| Bacteria β-Proteobacteria | *Cupriavidus* | 7 |
| Bacteria Cyanobacteria | *Cyanobium* | 7 |
| Bacteria Actinobacteria | *Denitrobacterium* | 7 |
| Bacteria δ-Proteobacteria | *Desulfobacca* | 7 |
| Bacteria δ-Proteobacteria | *Desulfobulbus* | 7 |
| Bacteria δ-Proteobacteria | *Desulfococcus* | 7 |
| Bacteria α-Proteobacteria | *Devosia* | 7 |
| Bacteria Cyanobacteria | *Gloeobacter* | 7 |
| Bacteria Acidobacteria | *Granulicella* | 7 |
| Archaea Euryarchaeota | *Haloferax* | 7 |
| Bacteria β-Proteobacteria | *Hydrogenophaga* | 7 |
| Bacteria α-Proteobacteria | *Hyphomonas* | 7 |
| Bacteria β-Proteobacteria | *Jeongeupia* | 7 |
| Bacteria Actinobacteria | *Kutzneria* | 7 |
| Bacteria Actinobacteria | *Kytococcus* | 7 |
| Bacteria β-Proteobacteria | *Massilia* | 7 |
| Archaea Euryarchaeota | *Methanomassiliicoccus* | 7 |
| Bacteria α-Proteobacteria | *Methylocella* | 7 |
| Bacteria Actinobacteria | *Microbacterium* | 7 |
| Bacteria Firmicutes | *Moorella* | 7 |
| Bacteria Actinobacteria | *Mycobacterium* | 7 |
| Bacteria α-Proteobacteria | *Neorhizobium* | 7 |
| Bacteria Nitrospirae | *Nitrospira* | 7 |
| Bacteria α-Proteobacteria | *Pannonibacter* | 7 |
| Bacteria α-Proteobacteria | *Paracoccus* | 7 |
| Bacteria β-Proteobacteria | *Paucibacter* | 7 |
| Bacteria α-Proteobacteria | *Porphyrobacter* | 7 |
| Bacteria β-Proteobacteria | *Pseudogulbenkiania* | 7 |
| Bacteria γ-Proteobacteria | *Rhodanobacter* | 7 |
| Bacteria Actinobacteria | *Rhodococcus* | 7 |
| Bacteria β-Proteobacteria | *Rhodoferax* | 7 |
| Bacteria α-Proteobacteria | *Rhodopseudomonas* | 7 |
| Bacteria α-Proteobacteria | *Rhodospirillum* | 7 |
| Bacteria β-Proteobacteria | *Rubrivivax* | 7 |
| Bacteria Actinobacteria | *Rubrobacter* | 7 |
| Bacteria α-Proteobacteria | *Ruegeria* | 7 |
| Bacteria Spirochaetes | *Salinispira* | 7 |
| Bacteria α-Proteobacteria | *Sinorhizobium* | 7 |
| Bacteria Spirochaetes | *Sphaerochaeta* | 7 |
| Bacteria γ-Proteobacteria | *Stenotrophomonas* | 7 |
| Bacteria Cyanobacteria | *Synechococcus* | 7 |
| Bacteria Firmicutes | *Thermincola* | 7 |
| Bacteria α-Proteobacteria | *Thiobacimonas* | 7 |
| Bacteria β-Proteobacteria | *Variovorax* | 7 |
| Bacteria γ-Proteobacteria | *Woeseia* | 7 |
| Bacteria Actinobacteria | *Agromyces* | 8 |
| Bacteria α-Proteobacteria | *Altererythrobacter* | 8 |
| Bacteria δ-Proteobacteria | *Archangium* | 8 |
| Bacteria α-Proteobacteria | *Azospirillum* | 8 |
| Bacteria γ-Proteobacteria | *Azotobacter* | 8 |
| Bacteria α-Proteobacteria | *Bradyrhizobium* | 8 |
| Bacteria Acidobacteria | *Candidatus Koribacter* | 8 |
| Bacteria Actinobacteria | *Catenulispora* | 8 |
| Bacteria α-Proteobacteria | *Caulobacter* | 8 |
| Bacteria α-Proteobacteria | *Celeribacter* | 8 |
| Bacteria Actinobacteria | *Cellulomonas* | 8 |
| Bacteria Chlorobi | *Chlorobaculum* | 8 |
| Bacteria α-Proteobacteria | *Confluentimicrobium* | 8 |
| Bacteria Actinobacteria | *Cutibacterium* | 8 |
| Bacteria β-Proteobacteria | *Dechloromonas* | 8 |
| Bacteria Actinobacteria | *Dietzia* | 8 |
| Bacteria γ-Proteobacteria | *Ectothiorhodospira* | 8 |
| Bacteria γ-Proteobacteria | *Frateuria* | 8 |
| Bacteria Actinobacteria | *Frondihabitans* | 8 |
| Bacteria δ-Proteobacteria | *Geobacter* | 8 |
| Archaea Euryarchaeota | *Halopenitus* | 8 |
| Bacteria α-Proteobacteria | *Ketogulonicigenium* | 8 |
| Bacteria α-Proteobacteria | *Labrenzia* | 8 |
| Bacteria Actinobacteria | *Leifsonia* | 8 |
| Bacteria γ-Proteobacteria | *Luteibacter* | 8 |
| Bacteria γ-Proteobacteria | *Marichromatium* | 8 |
| Bacteria α-Proteobacteria | *Martelella* | 8 |
| Bacteria β-Proteobacteria | *Methylibium* | 8 |
| Bacteria β-Proteobacteria | *Methyloversatilis* | 8 |
| Bacteria Actinobacteria | *Microterricola* | 8 |
| Archaea Halobacteria | *Natronorubrum* | 8 |
| Bacteria α-Proteobacteria | *Octadecabacter* | 8 |
| Bacteria α-Proteobacteria | *Polymorphum* | 8 |
| Bacteria Actinobacteria | *Propionibacterium* | 8 |
| Bacteria Actinobacteria | *Pseudonocardia* | 8 |
| Bacteria β-Proteobacteria | *Ramlibacter* | 8 |
| Bacteria α-Proteobacteria | *Rhodomicrobium* | 8 |
| Bacteria α-Proteobacteria | *Shinella* | 8 |
| Bacteria δ-Proteobacteria | *Sorangium* | 8 |
| Bacteria α-Proteobacteria | *Sphingobium* | 8 |
| Bacteria α-Proteobacteria | *Sphingomonas* | 8 |
| Bacteria Spirochaetes | *Spirochaeta* | 8 |
| Bacteria α-Proteobacteria | *Starkeya* | 8 |
| Bacteria Actinobacteria | *Thermobispora* | 8 |
| Archaea Euryarchaeota | *Thermococcus* | 8 |
| Bacteria Actinobacteria | *Xylanimonas* | 8 |
| Bacteria Fibrobacteres | *Fibrobacter* | 9 |
| Malate dehydrogenase (oxaloacetate-decarboxylating)(NADP+) [EC:1.1.1.40] | K00029 | 9 |
| Aldehyde dehydrogenase (NAD+) [EC:1.2.1.3] | K00128 | 9 |
| Acyl-ACP dehydrogenase [EC:1.3.99.-] | K00257 | 9 |
| NADH dehydrogenase I subunit B [EC:1.6.5.3] | K00331 | 9 |
| NADH dehydrogenase I subunit D [EC:1.6.5.3] | K00333 | 9 |
| NADH dehydrogenase I subunit F [EC:1.6.5.3] | K00335 | 9 |
| Homoserine O-acetyltransferase [EC:2.3.1.31] | K00641 | 9 |
| Endoglucanase [EC:3.2.1.4] | K01179 | 9 |
| Endo-1,4-beta-xylanase [EC:3.2.1.8] | K01181 | 9 |
| Leucyl aminopeptidase [EC:3.4.11.1] | K01255 | 9 |
| Phosphoenolpyruvate carboxykinase (GTP) [EC:4.1.1.32] | K01596 | 9 |
| Porphobilinogen synthase [EC:4.2.1.24] | K01698 | 9 |
| Glutamate-1-semialdehyde 2,1-aminomutase [EC:5.4.3.8] | K01845 | 9 |
| Peptide/nickel transport system ATP-binding protein | K02031 | 9 |
| Sulfonate/nitrate/taurine transport system ATP-binding protein | K02049 | 9 |
| Nitrogenase molybdenum-iron protein alpha chain [EC:1.18.6.1] | K02586 | 9 |
| Nitrogenase iron protein NifH [EC:1.18.6.1] | K02588 | 9 |
| Nitrogenase molybdenum-iron protein beta chain [EC:1.18.6.1] | K02591 | 9 |
| Type IV pilus assembly protein PilB | K02652 | 9 |
| TldD protein | K03568 | 9 |
| 7,8-dihydro-8-oxoguanine triphosphatase [EC:3.6.1.-] | K03574 | 9 |
| ATP-dependent DNA helicase DinG [EC:3.6.4.12] | K03722 | 9 |
| Virulence factor | K03980 | 9 |
| Glucose inhibited division protein Gid | K04094 | 9 |
| Dipeptidase E [EC:3.4.13.21] | K05995 | 9 |
| Flotillin | K07192 | 9 |
| D-alanyl-D-alanine carboxypeptidase [EC:3.4.16.4] | K07260 | 9 |
| Two-component system, NtrC family, nitrogen regulation response regulator GlnG | K07712 | 9 |
| Hypothetical protein | K09702 | 9 |
| DNA excision repair protein ERCC-3 [EC:3.6.4.12] | K10843 | 9 |
| ATP-dependent RNA helicase RhlE [EC:3.6.4.13] | K11927 | 9 |
| Anthranilate synthase/phosphoribosyltransferase [EC:4.1.3.27 2.4.2.18] | K13497 | 9 |
| Fungi Ascomycota | *Lachancea* | 9 |
| shikimate dehydrogenase [EC:1.1.1.25] | K00014 | 10 |
| riboflavin synthase alpha chain [EC:2.5.1.9] | K00793 | 10 |
| dephospho-CoA kinase [EC:2.7.1.24] | K00859 | 10 |
| 2-amino-4-hydroxy-6-hydroxymethyldihydropteridine diphosphokinase [EC:2.7.6.3] | K00950 | 10 |
| phosphatidate cytidylyltransferase [EC:2.7.7.41] | K00981 | 10 |
| phosphatidylserine synthase [EC:2.7.8.8] | K00998 | 10 |
| phosphoglycolate phosphatase [EC:3.1.3.18] | K01091 | 10 |
| N-acetylmuramoyl-L-alanine amidase [EC:3.5.1.28] | K01448 | 10 |
| 5-formyltetrahydrofolate cyclo-ligase [EC:6.3.3.2] | K01934 | 10 |
| iron complex transport system substrate-binding protein | K02016 | 10 |
| oxygen-independent coproporphyrinogen III oxidase [EC:1.3.99.22] | K02495 | 10 |
| Lipid A biosynthesis lauroyl acyltransferase [EC:2.3.1.-] | K02517 | 10 |
| Ribosomal protein L11 methyltransferase [EC:2.1.1.-] | K02687 | 10 |
| alanine or glycine:cation symporter, AGCS family | K03310 | 10 |
| glucose inhibited division protein A | K03495 | 10 |
| glucose inhibited division protein B [EC:2.1.-.-] | K03501 | 10 |
| BirA family transcriptional regulator, biotin operon repressor / biotin-[acetyl-CoA-carboxylase] ligase [EC:6.3.4.15] | K03524 | 10 |
| holliday junction DNA helicase RuvA | K03550 | 10 |
| thioredoxin 1 | K03671 | 10 |
| tRNA(Ile)-lysidine synthase [EC:6.3.4.-] | K04075 | 10 |
| integrase/recombinase XerD | K04763 | 10 |
| 4prime-phosphopantetheinyl transferase [EC:2.7.8.-] | K06133 | 10 |
| bifunctional oligoribonuclease and PAP phosphatase NrnA [EC:3.1.3.73.1.13.3] | K06881 | 10 |
| uncharacterized protein | K06921 | 10 |
| ribosome biogenesis GTPase [EC:3.6.1.-] | K06949 | 10 |
| ribosomal RNA large subunit methyltransferase I [EC:2.1.1.-] | K06969 | 10 |
| zinc transporter, ZIP family | K07238 | 10 |
| Putative endonuclease | K07460 | 10 |
| antibiotic transport system permease protein | K09686 | 10 |
| hypothetical protein | K09765 | 10 |
| cell division transport system permease protein | K09811 | 10 |
| carboxynorspermidine decarboxylase [EC:4.1.1.-] | K13747 | 10 |
| cystathione beta-lyase [EC:4.4.1.8] | K14155 | 10 |
| Bacteria Acidobacteria | *Acidobacterium* | 11 |
| Bacteria Actinobacteria | *Actinosynnema* | 11 |
| Bacteria Verrucomicrobia | *Akkermansia* | 11 |
| Bacteria Firmicutes | *Ammonifex* | 11 |
| Fungi Basidiomycota | *Anthracocystis* | 11 |
| Bacteria α-Proteobacteria | *Asaia* | 11 |
| Bacteria γ-Proteobacteria | *Brenneria* | 11 |
| Bacteria Firmicutes | Candidatus *Desulforudis* | 11 |
| Bacteria α-Proteobacteria | Candidatus *Symbiobacter* | 11 |
| Bacteria Actinobacteria | *Cryobacterium* | 11 |
| Bacteria Desulfitobacterium | *Desulfarculus* | 11 |
| Bacteria δ-Proteobacteria | *Desulfohalobium* | 11 |
| Bacteria δ-Proteobacteria | *Desulfurivibrio* | 11 |
| Bacteria α-Proteobacteria | *Dinoroseobacter* | 11 |
| Bacteria Cyanobacteria | *Geitlerinema* | 11 |
| Archaea Euryarchaeota | *Halobiforma* | 11 |
| Bacteria Firmicutes | *Limnochorda* | 11 |
| Bacteria α-Proteobacteria | *Magnetospirillum* | 11 |
| Bacteria α-Proteobacteria | *Marinovum* | 11 |
| Bacteria Deinococcus-Thermus | *Meiothermus* | 11 |
| Archaea Euryarchaeota | *Methanogenium* | 11 |
| Bacteria β-Proteobacteria | *Methylobacillus* | 11 |
| Archaea Halobacteria | *Natrialba* | 11 |
| Bacteria Cyanobacteria | *Pleurocapsa* | 11 |
| Bacteria Bacteroidetes | *Rhodothermus* | 11 |
| Bacteria Bacteroidetes | *Saprospira* | 11 |
| Bacteria Actinobacteria | *Segniliparus* | 11 |
| Bacteria Chloroflexi | *Sphaerobacter* | 11 |
| Bacteria Deinococcus-Thermus | *Thermus* | 11 |
| Bacteria Actinobacteria | *Verrucosispora* | 11 |
| Bacteria δ-Proteobacteria | *Vulgatibacter* | 11 |
| Bacteria Firmicutes | *Acidaminococcus* | 12 |
| Bacteria Firmicutes | *Allisonella* | 12 |
| Bacteria Firmicutes | *Aneurinibacillus* | 12 |
| Archaea Euryarchaeota | Candidatus *Methanomethylophilus* | 12 |
| Bacteria Firmicutes | *Jeotgalicoccus* | 12 |
| Hydroxylamine reductase [EC:1.7.-.-] | K00378 | 12 |
| DNA (cytosine-5-)-methyltransferase [EC:2.1.1.37] | K00558 | 12 |
| Carbamate kinase [EC:2.7.2.2] | K00926 | 12 |
| Type I restriction enzyme, R subunit [EC:3.1.21.3] | K01153 | 12 |
| Type I restriction enzyme, S subunit [EC:3.1.21.3] | K01154 | 12 |
| Glutaconyl-CoA decarboxylase [EC:4.1.1.70] | K01615 | 12 |
| Urocanate hydratase [EC:4.2.1.49] | K01712 | 12 |
| Glycyl-tRNA synthetase alpha chain [EC:6.1.1.14] | K01878 | 12 |
| Glycyl-tRNA synthetase beta chain [EC:6.1.1.14] | K01879 | 12 |
| DNA polymerase bacteriophage-type [EC:2.7.7.7] | K02334 | 12 |
| ATP-dependent Clp protease ATP-binding subunit ClpX | K03544 | 12 |
| 3-deoxy-7-phosphoheptulonate synthase [EC:2.5.1.54] | K03856 | 12 |
| Putative ABC transport system ATP-binding protein | K05833 | 12 |
| Presequence protease [EC:3.4.24.-] | K06972 | 12 |
| Uncharacterized protein | K07030 | 12 |
| Putative protein-disulfide isomerase | K07396 | 12 |
| DNA-damage-inducible protein J | K07473 | 12 |
| Spermidine/putrescine transport system permease protein | K11071 | 12 |
| Chorismate mutase / prephenate dehydratase [EC:5.4.99.5 4.2.1.51] | K14170 | 12 |
| Bacteria Firmicutes | *Megasphaera* | 12 |
| Bacteria Firmicutes | *Mitsuokella* | 12 |
| Archaea Halobacteria | *Natrinema* | 12 |
| Bacteria>Firmicutes | *Ruminococcus* | 12 |
| Bacteria Firmicutes | *Salinicoccus* | 12 |
| Bacteria ε-Proteobacteria | *Sulfurovum* | 12 |
| Bacteria α-Proteobacteria | *Thalassospira* | 12 |
| L-aspartate oxidase [EC:1.4.3.16] | K00278 | 13 |
| formate C-acetyltransferase [EC:2.3.1.54] | K00656 | 13 |
| 1,4-alpha-glucan branching enzyme [EC:2.4.1.18] | K00700 | 13 |
| 4-alpha-glucanotransferase [EC:2.4.1.25] | K00705 | 13 |
| pullulanase [EC:3.2.1.41] | K01200 | 13 |
| S-adenosylhomocysteine/5prime-methylthioadenosine nucleosidase [EC:3.2.2.9] | K01243 | 13 |
| Xaa-Pro aminopeptidase [EC:3.4.11.9] | K01262 | 13 |
| ATP-dependent Clp protease, protease subunit [EC:3.4.21.92] | K01358 | 13 |
| O-sialoglycoprotein endopeptidase [EC:3.4.24.57] | K01409 | 13 |
| GTP cyclohydrolase I [EC:3.5.4.16] | K01495 | 13 |
| UDP-glucose 4-epimerase [EC:5.1.3.2] | K01784 | 13 |
| glucose-6-phosphate isomerase [EC:5.3.1.9] | K01810 | 13 |
| tryptophanyl-tRNA synthetase [EC:6.1.1.2] | K01867 | 13 |
| histidyl-tRNA synthetase [EC:6.1.1.21] | K01892 | 13 |
| CTP synthase [EC:6.3.4.2] | K01937 | 13 |
| adenylosuccinate synthase [EC:6.3.4.4] | K01939 | 13 |
| F-type H+-transporting ATPase subunit a [EC:3.6.3.14] | K02108 | 13 |
| F-type H+-transporting ATPase subunit b [EC:3.6.3.14] | K02109 | 13 |
| F-type H+-transporting ATPase subunit gamma [EC:3.6.3.14] | K02115 | 13 |
| replicative DNA helicase [EC:3.6.4.12] | K02314 | 13 |
| DNA polymerase III subunit alpha [EC:2.7.7.7] | K02337 | 13 |
| peptide chain release factor RF-1 | K02835 | 13 |
| preprotein translocase subunit YidC | K03217 | 13 |
| holliday junction DNA helicase RuvB | K03551 | 13 |
| recombination protein RecA | K03553 | 13 |
| electron transport complex protein RnfE | K03613 | 13 |
| electron transport complex protein RnfC | K03615 | 13 |
| transcription termination factor Rho | K03628 | 13 |
| GTP-binding protein HflX | K03665 | 13 |
| GTP-binding protein | K06207 | 13 |
| pyridoxine biosynthesis protein [EC:4.-.-.-] | K06215 | 13 |
| Bacteria γ-Proteobacteria | *Agarivorans* | 14 |
| Bacteria γ-Proteobacteria | *Aggregatibacter* | 14 |
| Bacteria γ-Proteobacteria | *Alteromonas* | 14 |
| Bacteria Enterobacteria | Candidatus *Blochmannia* | 14 |
| Bacteria Enterobacteria | Candidatus *Hoaglandella* | 14 |
| Bacteria γ-Proteobacteria | *Cedecea* | 14 |
| Bacteria γ-Proteobacteria | *Dichelobacter* | 14 |
| Bacteria Sarcomatigophora | *Giardia* | 14 |
| Bacteria γ-Proteobacteria | *Gilliamella* | 14 |
| Bacteria γ-Proteobacteria | *Haemophilus* | 14 |
| Bacteria γ-Proteobacteria | *Idiomarina* | 14 |
| Bacteria δ-Proteobacteria | *Lawsonia* | 14 |
| Fungi Basidiomycota | *Mannheimia* | 14 |
| Bacteria ε-Proteobacteria | *Nitratiruptor* | 14 |
| Bacteria γ-Proteobacteria | *Oblitimonas* | 14 |
| Bacteria γ-Proteobacteria | *Paraglaciecola* | 14 |
| Bacteria Thermotogae | *Petrotoga* | 14 |
| Bacteria Enterobacteria | *Pragia* | 14 |
| Bacteria Enterobacteria | *Providencia* | 14 |
| Bacteria γ-Proteobacteria | *Shewanella* | 14 |
| Bacteria Enterobacteria | *Shigella* | 14 |
| Bacteria γ-Proteobacteria | *Succinivibrio* | 14 |
| Bacteria γ-Proteobacteria | *Sulfuricella* | 14 |
| Bacteria γ-Proteobacteria | *Thalassolituus* | 14 |
| Bacteria γ-Proteobacteria | *Vibrio* | 14 |
| Bacteria Enterobacteria | *Xenorhabdus* | 14 |
| Bacteria γ-Proteobacteria | *Yersinia* | 14 |
| UDP-N-acetylmuramate dehydrogenase [EC:1.1.1.158] | K00075 | 15 |
| Glutamate-5-semialdehyde dehydrogenase [EC:1.2.1.41] | K00147 | 15 |
| methylenetetrahydrofolate reductase (NADPH) [EC:1.5.1.20] | K00297 | 15 |
| thioredoxin reductase (NADPH) [EC:1.8.1.9] | K00384 | 15 |
| methionyl-tRNA formyltransferase [EC:2.1.2.9] | K00604 | 15 |
| Histidinol-phosphate aminotransferase [EC:2.6.1.9] | K00817 | 15 |
| phospho-N-acetylmuramoyl-pentapeptide-transferase [EC:2.7.8.13] | K01000 | 15 |
| dGTPase [EC:3.1.5.1] | K01129 | 15 |
| exodeoxyribonuclease III [EC:3.1.11.2] | K01142 | 15 |
| 1-deoxy-D-xylulose-5-phosphate synthase [EC:2.2.1.7] | K01662 | 15 |
| UDP-N-acetylmuramate--alanine ligase [EC:6.3.2.8] | K01924 | 15 |
| methyltransferase [EC:2.1.1.-] | K02493 | 15 |
| UDP-N-acetylglucosamine--N-acetylmuramyl-(pentapeptide) pyrophosphoryl-undecaprenol N-acetylglucosamine transferase [EC:2.4.1.227] | K02563 | 15 |
| Signal peptidase II [EC:3.4.23.36] | K03101 | 15 |
| RNA methyltransferase, TrmH family | K03437 | 15 |
| tRNA (guanine-N7-)-methyltransferase [EC:2.1.1.33] | K03439 | 15 |
| ribonuclease HII [EC:3.1.26.4] | K03470 | 15 |
| 4-hydroxy-3-methylbut-2-enyl diphosphate reductase [EC:1.17.1.2] | K03527 | 15 |
| cell division protein FtsQ | K03589 | 15 |
| ATP-binding protein involved in chromosome partitioning | K03593 | 15 |
| DNA replication and repair protein RecF | K03629 | 15 |
| ATP-dependent DNA helicase RecQ [EC:3.6.4.12] | K03654 | 15 |
| excinuclease ABC subunit C | K03703 | 15 |
| PLP dependent protein | K06997 | 15 |
| UPF0755 protein | K07082 | 15 |
| ribosomal RNA small subunit methyltransferase E [EC:2.1.1.-] | K09761 | 15 |
| Bacteria Chloroflexi | *Anaerolinea* | 16 |
| Archaea Euryarchaeota | *Archaeoglobus* | 16 |
| Bacteria α-Proteobacteria | *Brucella* | 16 |
| Bacteria Verrucomicrobia | Candidatus *Xiphinematobacter* | 16 |
| Bacteria Acidobacteria | *Chloracidobacterium* | 16 |
| Bacteria δ-Proteobacteria | *Chondromyces* | 16 |
| Bacteria Cyanobacteria | *Dactylococcopsis* | 16 |
| Fungi Basidiomycota | *Dichomitus* | 16 |
| Bacteria Bacteroidetes | *Emticicia* | 16 |
| Bacteria γ-Proteobacteria | *Endozoicomonas* | 16 |
| Archaea Euryarchaeota | *Halogeometricum* | 16 |
| Archaea Euryarchaeota | *Halovivax* | 16 |
| Bacteria Planctomycetes | *Isosphaera* | 16 |
| Bacteria Actinobacteria | *Jonesia* | 16 |
| Bacteria α-Proteobacteria | *Oligotropha* | 16 |
| Bacteria Verrucomicrobia | *Opitutus* | 16 |
| Bacteria Planctomycetes | *Paludisphaera* | 16 |
| Bacteria α-Proteobacteria | *Parvularcula* | 16 |
| Bacteria α-Proteobacteria | *Planktomarina* | 16 |
| Bacteria Planctomycetes | *Rhodopirellula* | 16 |
| Archaea Euryarchaeota | *Salinarchaeum* | 16 |
| Bacteria Planctomycetes | *Singulisphaera* | 16 |
| Bacteria Thermodesulfobacteria | *Thermodesulfatator* | 16 |
| Fungi Basidiomycota | *Trametes* | 16 |
| Bacteria Verrucomicrobia | *Verrucomicrobium* | 16 |
| 3-methyl-2-oxobutanoate hydroxymethyltransferase [EC:2.1.2.11] | K00606 | 17 |
| Nicotinate-nucleotide pyrophosphorylase (carboxylating) [EC:2.4.2.19] | K00767 | 17 |
| glucokinase [EC:2.7.1.2] | K00845 | 17 |
| guanylate kinase [EC:2.7.4.8] | K00942 | 17 |
| 3-deoxy-manno-octulosonate cytidylyltransferase (CMP-KDO synthetase) [EC:2.7.7.38] | K00979 | 17 |
| ATP-dependent Lon protease [EC:3.4.21.53] | K01338 | 17 |
| phosphatidylserine decarboxylase [EC:4.1.1.65] | K01613 | 17 |
| ABC-2 type transport system ATP-binding protein | K01990 | 17 |
| Hit-like protein involved in cell-cycle regulation | K02503 | 17 |
| UDP-3-O-[3-hydroxymyristoyl] glucosamine N-acyltransferase [EC:2.3.1.-] | K02536 | 17 |
| signal peptidase I [EC:3.4.21.89] | K03100 | 17 |
| preprotein translocase subunit YajC | K03210 | 17 |
| N utilization substance protein B | K03625 | 17 |
| carboxyl-terminal processing protease [EC:3.4.21.102] | K03797 | 17 |
| GTP-binding protein | K03978 | 17 |
| penicillin-binding protein 1A [EC:2.4.1.- 3.4.-.-] | K05366 | 17 |
| penicillin-binding protein 2 | K05515 | 17 |
| putative transport protein | K07085 | 17 |
| NA | K07098 | 17 |
| S-adenosylmethionine:tRNA ribosyltransferase-isomerase [EC:5.-.-.-] | K07568 | 17 |
| ribonuclease G [EC:3.1.26.-] | K08301 | 17 |
| hypothetical protein | K09748 | 17 |
| Bacteria Actinobacteria | *Beutenbergia* | 18 |
| Bacteria Chloroflexi | *Caldilinea* | 18 |
| Bacteria γ-Proteobacteria | *Citrobacter* | 18 |
| Bacteria Firmicutes | *Desulfitobacterium* | 18 |
| Bacteria Firmicutes | *Desulfosporosinus* | 18 |
| Bacteria Firmicutes | *Desulfotomaculum* | 18 |
| Bacteria α-Proteobacteria | *Ensifer* | 18 |
| Bacteria Firmicutes | *Erysipelatoclostridium* | 18 |
| Bacteria Firmicutes | *Ethanoligenens* | 18 |
| Bacteria Firmicutes | *Faecalibaculum* | 18 |
| Bacteria Firmicutes | *Heliobacterium* | 18 |
| Maltose/maltodextrin transport system ATP-binding protein | K10112 | 18 |
| Bacteria Firmicutes | *Lachnoclostridium* | 18 |
| Bacteria Firmicutes | *Mageeibacillus* | 18 |
| Archea>Euryarchaeota | *Methanococcoides* | 18 |
| Bacteria Firmicutes | *Oscillibacter* | 18 |
| Bacteria Firmicutes | *Paenibacillus* | 18 |
| Bacteria Actinobacteria | *Parascardovia* | 18 |
| Bacteria α-Proteobacteria | *Pelagibaca* | 18 |
| Bacteria γ-Proteobacteria | *Steroidobacter* | 18 |
| Bacteria α-Proteobacteria | *Tistrella* | 18 |
| Bacteria Firmicutes | *Tyzzerella* | 18 |
| Adenylylsulfate reductase, subunit A [EC:1.8.99.2] | K00394 | 19 |
| UDP-N-acetylglucosamine pyrophosphorylase [EC:2.7.7.23] | K00972 | 19 |
| Biotin synthetase [EC:2.8.1.6] | K01012 | 19 |
| Histidine ammonia-lyase [EC:4.3.1.3] | K01745 | 19 |
| General secretion pathway protein E | K02454 | 19 |
| ribosomal RNA small subunit methyltransferase B [EC:2.1.1.-] | K03500 | 19 |
| Exodeoxyribonuclease V alpha subunit [EC:3.1.11.5] | K03581 | 19 |
| glycerol-3-phosphate acyltransferase PlsX [EC:2.3.1.15] | K03621 | 19 |
| 3-dehydroquinate dehydratase I [EC:4.2.1.10] | K03785 | 19 |
| Putative transcription regulator | K03976 | 19 |
| molecular chaperone Hsp33 | K04083 | 19 |
| Cysteine desulfurase [EC:2.8.1.7] | K04487 | 19 |
| ferrous iron transport protein A | K04758 | 19 |
| HPr kinase/phosphorylase [EC:2.7.11.- 2.7.4.-] | K06023 | 19 |
| Segregation and condensation protein B | K06024 | 19 |
| Ribosomal large subunit pseudouridine synthase C [EC:5.4.99.12] | K06179 | 19 |
| 3',5'-nucleoside bisphosphate phosphatase [EC:3.1.3.97] | K07053 | 19 |
| oligoendopeptidase F [EC:3.4.24.-] | K08602 | 19 |
| zinc transport system ATP-binding protein [EC:3.6.3.-] | K09817 | 19 |
| phosphocarrier protein | K11189 | 19 |
| Phosphatidylglycerol:prolipoprotein diacylglycerol transferase [EC:2.-.-.-] | K13292 | 19 |
| aminoacylhistidine dipeptidase [EC:3.4.13.3] | K01270 | 20 |
| deoxyribose-phosphate aldolase [EC:4.1.2.4] | K01619 | 20 |
| Threonine aldolase [EC:4.1.2.5] | K01620 | 20 |
| 2-dehydro-3-deoxyphosphooctonate aldolase (KDO 8-P synthase) [EC:2.5.1.55] | K01627 | 20 |
| Anthranilate synthase component II [EC:4.1.3.27] | K01658 | 20 |
| aldose 1-epimerase [EC:5.1.3.3] | K01785 | 20 |
| aspartate--ammonia ligase [EC:6.3.1.1] | K01914 | 20 |
| pantoate--beta-alanine ligase [EC:6.3.2.1] | K01918 | 20 |
| glycosyltransferase 2 family protein | K02014 | 20 |
| transcriptional regulator of arginine metabolism | K03402 | 20 |
| cell division protein FtsW | K03588 | 20 |
| putative colanic acid biosysnthesis UDP-glucose lipid carrier transferase | K03606 | 20 |
| electron transport complex protein RnfD | K03614 | 20 |
| transcription elongation factor GreA | K03624 | 20 |
| integrase/recombinase XerC | K03733 | 20 |
| pyruvate formate lyase activating enzyme [EC:1.97.1.4] | K04069 | 20 |
| phosphate starvation-inducible protein PhoH and related proteins | K06217 | 20 |
| putative holliday junction resolvase [EC:3.1.-.-] | K07447 | 20 |
| two-component system, OmpR family, phosphate regulon sensor histidine kinase PhoR [EC:2.7.13.3] | K07636 | 20 |
| ATP-binding cassette, subfamily B, bacterial MsbA [EC:3.6.3.-] | K11085 | 20 |
| indolepyruvate ferredoxin oxidoreductase, alpha subunit [EC:1.2.7.8] | K00179 | 21 |
| indolepyruvate ferredoxin oxidoreductase, beta subunit [EC:1.2.7.8] | K00180 | 21 |
| Dihydrodipicolinate reductase [EC:1.3.1.26] | K00215 | 21 |
| NADH dehydrogenase I subunit H [EC:1.6.5.3] | K00337 | 21 |
| NADH dehydrogenase I subunit J [EC:1.6.5.3] | K00339 | 21 |
| NADH dehydrogenase I subunit K [EC:1.6.5.3] | K00340 | 21 |
| glutathione peroxidase [EC:1.11.1.9] | K00432 | 21 |
| formyltetrahydrofolate deformylase [EC:3.5.1.10] | K01433 | 21 |
| arginine decarboxylase [EC:4.1.1.19] | K01585 | 21 |
| phenylacetate-CoA ligase [EC:6.2.1.30] | K01912 | 21 |
| pyruvate carboxylase subunit B [EC:6.4.1.1] | K01960 | 21 |
| DNA polymerase III subunit epsilon [EC:2.7.7.7] | K02342 | 21 |
| topoisomerase IV subunit B [EC:5.99.1.-] | K02622 | 21 |
| diaminopimelate dehydrogenase [EC:1.4.1.16] | K03340 | 21 |
| prephenate dehydratase [EC:4.2.1.51] | K04518 | 21 |
| tryptophan synthase beta chain [EC:4.2.1.20] | K06001 | 21 |
| NA | K07089 | 21 |
| hypothetical protein | K09125 | 21 |
| 3-isopropylmalate dehydrogenase [EC:1.1.1.85] | K00052 | 22 |
| pyrroline-5-carboxylate reductase [EC:1.5.1.2] | K00286 | 22 |
| 1-acyl-sn-glycerol-3-phosphate acyltransferase [EC:2.3.1.51] | K00655 | 22 |
| myo-inositol-1(or 4)-monophosphatase [EC:3.1.3.25] | K01092 | 22 |
| protein-tyrosine phosphatase [EC:3.1.3.48] | K01104 | 22 |
| chromosomal replication initiator protein | K02313 | 22 |
| DNA polymerase III subunit beta [EC:2.7.7.7] | K02338 | 22 |
| DNA repair protein RecO (recombination protein O) | K03584 | 22 |
| exodeoxyribonuclease VII large subunit [EC:3.1.11.6] | K03601 | 22 |
| DNA repair protein RecN (Recombination protein N) | K03631 | 22 |
| SsrA-binding protein | K03664 | 22 |
| xanthine phosphoribosyltransferase [EC:2.4.2.22] | K03816 | 22 |
| DNA processing protein | K04096 | 22 |
| Recombination protein RecR | K06187 | 22 |
| magnesium chelatase family protein | K07391 | 22 |
| glutamine amidotransferase [EC:2.6.-.-] | K08681 | 22 |
| hypothetical protein | K08998 | 22 |
| riboflavin kinase / FMN adenylyltransferase [EC:2.7.1.26 2.7.7.2] | K11753 | 22 |
| NADH dehydrogenase I subunit G [EC:1.6.5.3] | K00336 | 23 |
| Adenosylmethionine-8-amino-7-oxononanoate aminotransferase [EC:2.6.1.62] | K00833 | 23 |
| Sulfate adenylyltransferase subunit 1 [EC:2.7.7.4] | K00956 | 23 |
| Sulfate adenylyltransferase subunit 2 [EC:2.7.7.4] | K00957 | 23 |
| 4-hydroxy 2-oxovalerate aldolase [EC:4.1.3.39] | K01666 | 23 |
| Sulfate transport system substrate-binding protein | K02048 | 23 |
| Phosphoserine / homoserine phosphotransferase [EC:3.1.3.3 2.7.1.39] | K02203 | 23 |
| Precorrin-2 dehydrogenase / sirohydrochlorin ferrochelatase [EC:1.3.1.76 4.99.1.4] | K02304 | 23 |
| 3R-hydroxymyristoyl ACP dehydrase [EC:4.2.1.-] | K02372 | 23 |
| Twitching motility protein PilT | K02669 | 23 |
| Pyrimidine operon attenuation protein / uracil phosphoribosyltransferase [EC:2.4.2.9] | K02825 | 23 |
| Hydrophobic/amphiphilic exporter-1 (mainly G- bacteria), HAE1 family | K03296 | 23 |
| Biopolymer transport protein ExbD | K03559 | 23 |
| Uncharacterized protein | K06959 | 23 |
| Uncharacterized protein | K07003 | 23 |
| Two-component system, OmpR family, response regulator VicR | K07668 | 23 |
| Soluble lytic murein transglycosylase [EC:3.2.1.-] | K08309 | 23 |
| Aminotransferase [EC:2.6.1.-] | K10907 | 23 |
| Bacteria Firmicutes | *Anaerococcus* | 24 |
| Bacteria ε-Proteobacteria | *Arcobacter* | 24 |
| Bacteria Spirochaetes | *Borreliella* | 24 |
| Bacteria ε-Proteobacteria | *Campylobacter* | 24 |
| Bacteria Firmicutes | *Carnobacterium* | 24 |
| Bacteria Dictyoglomi | *Dictyoglomus* | 24 |
| Bacteria Firmicutes | *Erysipelothrix* | 24 |
| Bacteria Fusobacteria | *Fusobacterium* | 24 |
| Bacteria β-Proteobacteria | *Kinetoplastibacterium* | 24 |
| Bacteria Firmicutes | *Lactococcus* | 24 |
| Bacteria Fusobacteria | *Leptotrichia* | 24 |
| Bacteria Firmicutes | *Macrococcus* | 24 |
| Bacteria Thermotogae | *Marinitoga* | 24 |
| Bacteria Fusobacteria | *Sebaldella* | 24 |
| Bacteria ε-Proteobacteria | *Sulfurimonas* | 24 |
| Bacteria Firmicutes | *Terrisporobacter* | 24 |
| Bacteria Aquificales | *Thermocrinis* | 24 |
| hypothetical protein | K00243 | 25 |
| lipid-A-disaccharide synthase [EC:2.4.1.182] | K00748 | 25 |
| 2-C-methyl-D-erythritol 2,4-cyclodiphosphate synthase [EC:4.6.1.12] | K01770 | 25 |
| RNA polymerase sigma-54 factor | K03092 | 25 |
| Rod shape-determining protein MreC | K03570 | 25 |
| DNA mismatch repair protein MutL | K03572 | 25 |
| tRNA modification GTPase | K03650 | 25 |
| ATP-dependent RNA helicase DeaD [EC:3.6.4.13] | K05592 | 25 |
| rod shape determining protein RodA | K05837 | 25 |
| Bifunctional enzyme involved in thiolation and methylation of tRNA | K06168 | 25 |
| membrane protein | K07058 | 25 |
| phosphoribosylglycinamide formyltransferase 1 [EC:2.1.2.2] | K11175 | 25 |
| N-carbamoylputrescine amidase [EC:3.5.1.53] | K12251 | 25 |
| Ribonuclease R [EC:3.1.-.-] | K12573 | 25 |
| phosphopantothenoylcysteine decarboxylase / phosphopantothenate--cysteine ligase [EC:4.1.1.36 6.3.2.5] | K13038 | 25 |
| geranylgeranyl diphosphate synthase, type II [EC:2.5.1.1 2.5.1.10 2.5.1.29] | K13789 | 25 |
| Ribosomal protein S12 methylthiotransferase [EC:2.-.-.-] | K14441 | 25 |
| Dihydrofolate reductase [EC:1.5.1.3] | K00287 | 26 |
| hypothetical protein | K00783 | 26 |
| UDP-N-acetylglucosamine 1-carboxyvinyltransferase [EC:2.5.1.7] | K00790 | 26 |
| glucosamine--fructose-6-phosphate aminotransferase (isomerizing) [EC:2.6.1.16] | K00820 | 26 |
| 4-diphosphocytidyl-2-C-methyl-D-erythritol kinase [EC:2.7.1.148] | K00919 | 26 |
| ribose-phosphate pyrophosphokinase [EC:2.7.6.1] | K00948 | 26 |
| L-asparaginase [EC:3.5.1.1] | K01424 | 26 |
| D-methionine transport system ATP-binding protein | K02071 | 26 |
| D-methionine transport system permease protein | K02072 | 26 |
| D-methionine transport system substrate-binding protein | K02073 | 26 |
| Single-strand DNA-binding protein | K03111 | 26 |
| excinuclease ABC subunit A | K03701 | 26 |
| Ribosomal-protein-alanine N-acetyltransferase [EC:2.3.1.128] | K03789 | 26 |
| Heat shock protein HtpX [EC:3.4.24.-] | K03799 | 26 |
| ATP-binding cassette, subfamily B, bacterial | K06147 | 26 |
| Septum formation protein | K06287 | 26 |
| Dihydrofolate synthase / folylpolyglutamate synthase [EC:6.3.2.12 6.3.2.17] | K11754 | 26 |
| glycerate dehydrogenase [EC:1.1.1.29] | K00018 | 27 |
| protoporphyrinogen oxidase [EC:1.3.3.4] | K00231 | 27 |
| glycerate kinase [EC:2.7.1.31] | K00865 | 27 |
| alpha-mannosidase [EC:3.2.1.24] | K01191 | 27 |
| muramoyltetrapeptide carboxypeptidase [EC:3.4.17.13] | K01297 | 27 |
| uroporphyrinogen decarboxylase [EC:4.1.1.37] | K01599 | 27 |
| HlyD family secretion protein | K02022 | 27 |
| isochorismate synthase [EC:5.4.4.2] | K02361 | 27 |
| DnaJ like chaperone protein | K05801 | 27 |
| arabinan endo-1,5-alpha-L-arabinosidase [EC:3.2.1.99] | K06113 | 27 |
| ribosomal large subunit pseudouridine synthase A [EC:5.4.99.12] | K06177 | 27 |
| maltose 6'-phosphate phosphatase [EC:3.1.3.90] | K06896 | 27 |
| uncharacterized protein | K07000 | 27 |
| lipopolysaccharide cholinephosphotransferase [EC:2.7.8.-] | K07271 | 27 |
| hypothetical protein | K09704 | 27 |
| hexosaminidase [EC:3.2.1.52] | K12373 | 27 |
| histidinol dehydrogenase [EC:1.1.1.23] | K00013 | 28 |
| hypoxanthine phosphoribosyltransferase [EC:2.4.2.8] | K00760 | 28 |
| Undecaprenyl diphosphate synthase [EC:2.5.1.31] | K00806 | 28 |
| dUTP pyrophosphatase [EC:3.6.1.23] | K01520 | 28 |
| Dihydroxy-acid dehydratase [EC:4.2.1.9] | K01687 | 28 |
| D-alanine-D-alanine ligase [EC:6.3.2.4] | K01921 | 28 |
| UDP-N-acetylmuramoylalanyl-D-glutamate--2,6-diaminopimelate ligase [EC:6.3.2.13] | K01928 | 28 |
| ribosome recycling factor | K02838 | 28 |
| large subunit ribosomal protein L25 | K02897 | 28 |
| preprotein translocase subunit SecE | K03073 | 28 |
| fused signal recognition particle receptor | K03110 | 28 |
| DNA segregation ATPase FtsK/SpoIIIE, S-DNA-T family | K03466 | 28 |
| (E)-4-hydroxy-3-methylbut-2-enyl-diphosphate synthase [EC:1.17.7.1] | K03526 | 28 |
| exodeoxyribonuclease VII small subunit [EC:3.1.11.6] | K03602 | 28 |
| GTP-binding protein | K03979 | 28 |
| NAD-dependent deacetylase [EC:3.5.1.-] | K12410 | 28 |
| hypothetical protein | K00782 | 29 |
| thiamine-monophosphate kinase [EC:2.7.4.16] | K00946 | 29 |
| mannose-1-phosphate guanylyltransferase [EC:2.7.7.22] | K00971 | 29 |
| cysteine desulfuration protein SufE | K02426 | 29 |
| nucleoside-triphosphate pyrophosphatase [EC:3.6.1.19] | K02428 | 29 |
| DNA topoisomerase III [EC:5.99.1.2] | K03169 | 29 |
| ubiquinone/menaquinone biosynthesis methyltransferase [EC:2.1.1.-] | K03183 | 29 |
| Sulfate permease, SulP family | K03321 | 29 |
| cell division protein FtsA | K03590 | 29 |
| Peptidyl-prolyl cis-trans isomerase SurA [EC:5.2.1.8] | K03771 | 29 |
| putative lipoprotein | K05807 | 29 |
| long-chain fatty acid transport protein | K06076 | 29 |
| lipopolysaccharide export system ATP-binding protein [EC:3.6.3.-] | K06861 | 29 |
| 7-cyano-7-deazaguanine reductase [EC:1.7.1.13] | K09457 | 29 |
| hypothetical protein | K09922 | 29 |
| Bacteria Bacteroidetes | *Algibacter* | 30 |
| Bacteria Bacteroidetes | *Arachidicoccus* | 30 |
| Bacteria Bacteroidetes | *Chryseobacterium* | 30 |
| 16S ribosomal RNA | K01977 | 30 |
| 23S ribosomal RNA | K01980 | 30 |
| Putative transposase | K07496 | 30 |
| tRNA Ala | K14218 | 30 |
| tRNA Asn | K14220 | 30 |
| tRNA Tyr | K14236 | 30 |
| Bacteria β-Proteobacteria | *Methylophilus* | 30 |
| Bacteria Bacteroidetes | *Odoribacter* | 30 |
| Bacteria Firmicutes | *Selenomonas* | 30 |
| Bacteria γ-Proteobacteria | *Tatlockia* | 30 |
| Bacteria Firmicutes | *Acetobacterium* | 31 |
| Bacteria Firmicutes | *Anoxybacillus* | 31 |
| Bacteria Firmicutes | *Caldicellulosiruptor* | 31 |
| Bacteria Firmicutes | Candidatus *Arthromitus* | 31 |
| Bacteria Firmicutes | *Cellulosilyticum* | 31 |
| Bacteria Firmicutes | *Ezakiella* | 31 |
| Bacteria Firmicutes | *Leuconostoc* | 31 |
| Bacteria Fusobacteria | *Sneathia* | 31 |
| Bacteria Firmicutes | *Tepidanaerobacter* | 31 |
| Bacteria Firmicutes | *Terribacillus* | 31 |
| Bacteria Firmicutes | *Thermoanaerobacter* | 31 |
| Bacteria Firmicutes | *Thermoanaerobacterium* | 31 |
| Bacteria Firmicutes | *Virgibacillus* | 31 |
| Bacteria Firmicutes | *Acetitomaculum* | 32 |
| Bacteria Firmicutes | *Blautia* | 32 |
| Bacteria Firmicutes | *Dorea* | 32 |
| Bacteria Firmicutes | *Faecalitalea* | 32 |
| M1 RNA | K01978 | 32 |
| Iron(III) transport system permease protein | K02011 | 32 |
| Cold shock protein (beta-ribbon, CspA family) | K03704 | 32 |
| Uncharacterized protein | K09861 | 32 |
| Putative glutamine transport system ATP-binding protein [EC:3.6.3.-] | K10041 | 32 |
| Spermidine/putrescine transport system ATP-binding protein [EC:3.6.3.31] | K11072 | 32 |
| Bacteria Firmicutes | *Lachnospira* | 32 |
| Bacteria Firmicutes | *Roseburia* | 32 |
| Bacteria β-Proteobacteria | *Advenella* | 33 |
| Archaea Euryarchaeota | Candidatus *Methanoperedens* | 33 |
| Bacteria Cloroflexi | *Dehalococcoides* | 33 |
| Bacteria Firmicutes | *Dialister* | 33 |
| Bacteria Firmicutes | *Eubacterium* | 33 |
| Bacteria Firmicutes | *Jeotgalibacillus* | 33 |
| xylulose-5-phosphate/fructose-6-phosphate phosphoketolase [EC:4.1.2.9 4.1.2.22] | K01621 | 33 |
| Iron(III) transport system substrate-binding protein | K02012 | 33 |
| RNA polymerase sigma-B factor | K03090 | 33 |
| Bacteria Firmicutes | *Pelosinus* | 33 |
| Bacteria Firmicutes | *Salimicrobium* | 33 |
| Bacteria Firmicutes | *Veillonella* | 33 |
| homoserine dehydrogenase [EC:1.1.1.3] | K00003 | 34 |
| 5-methyltetrahydropteroyltriglutamate--homocysteine methyltransferase [EC:2.1.1.14] | K00549 | 34 |
| Glutamate N-acetyltransferase / amino-acid N-acetyltransferase [EC:2.3.1.35 2.3.1.1] | K00620 | 34 |
| CDP-diacylglycerol--glycerol-3-phosphate 3-phosphatidyltransferase [EC:2.7.8.5] | K00995 | 34 |
| Phosphoribosyl-ATP pyrophosphohydrolase [EC:3.6.1.31] | K01523 | 34 |
| 3-isopropylmalate/(R)-2-methylmalate dehydratase large subunit [EC:4.2.1.33 4.2.1.35] | K01703 | 34 |
| 3-isopropylmalate/(R)-2-methylmalate dehydratase small subunit [EC:4.2.1.33 4.2.1.35] | K01704 | 34 |
| ribose 5-phosphate isomerase A [EC:5.3.1.6] | K01807 | 34 |
| carbamoyl-phosphate synthase small subunit [EC:6.3.5.5] | K01956 | 34 |
| Peptide/nickel transport system substrate-binding protein | K02035 | 34 |
| Sulfonate/nitrate/taurine transport system permease protein | K02050 | 34 |
| Undecaprenyl-diphosphatase [EC:3.6.1.27] | K06153 | 34 |
| uridine phosphorylase [EC:2.4.2.3] | K00757 | 35 |
| uridine kinase [EC:2.7.1.48] | K00876 | 35 |
| sec-independent protein translocase protein TatC | K03118 | 35 |
| Na+:H+ antiporter, NhaC family | K03315 | 35 |
| membrane fusion protein | K03585 | 35 |
| competence/damage-inducible protein CinA | K03742 | 35 |
| 3-dehydroquinate dehydratase II [EC:4.2.1.10] | K03786 | 35 |
| LacI family transcriptional regulator, repressor for deo operon, udp, cdd, tsx, nupC, and nupG | K05499 | 35 |
| arabinose-5-phosphate isomerase [EC:5.3.1.13] | K06041 | 35 |
| L-fuconolactonase [EC:3.1.1.-] | K07046 | 35 |
| Phosphoribosyl-ATP pyrophosphohydrolase / phosphoribosyl-AMP cyclohydrolase [EC:3.6.1.31 3.5.4.19] | K11755 | 35 |
| NADH dehydrogenase I subunit N [EC:1.6.5.3] | K00343 | 36 |
| Dolichol-phosphate mannosyltransferase [EC:2.4.1.83] | K00721 | 36 |
| HlyD family secretion protein | K01993 | 36 |
| Octaprenyl diphosphate synthase [EC:2.5.1.-] | K02523 | 36 |
| Peptidyl-prolyl cis-trans isomerase D [EC:5.2.1.8] | K03770 | 36 |
| 7,8-dihydropterin-6-yl-methyl-4-(beta-D-ribofuranosyl)aminobenzene 5'-Phosphate synthase [EC:2.5.1.105] | K06897 | 36 |
| Uncharacterized protein | K07037 | 36 |
| sigma-B regulation protein RsbU (phosphoserine phosphatase) | K07315 | 36 |
| membrane-bound lytic murein transglycosylase D [EC:3.2.1.-] | K08307 | 36 |
| hypothetical protein | K09797 | 36 |
| Lipoprotein-releasing system permease protein | K09808 | 36 |
| Bacteria Enterobacteria | *Arsenophonus* | 37 |
| Bacteria Bacteroidetes | *Blattabacterium* | 37 |
| Bacteria Spirochaetes | *Borrelia* | 37 |
| Bacteria Cyanobacteria | Candidatus *Atelocyanobacterium* | 37 |
| Bacteria Firmicutes | *Halanaerobium* | 37 |
| Bacteria Firmicutes | *Halobacteroides* | 37 |
| Bacteria Bacteroidetes | *Lacinutrix* | 37 |
| Bacteria Tenericutes | *Mycoplasma* | 37 |
| Bacteria Cyanobacteria | *Prochlorococcus* | 37 |
| Archaea Crenarchaeota | *Sulfolobus* | 37 |
| Bacteria Tenericutes | *Ureaplasma* | 37 |
| Adenosylcobinamide-phosphate synthase CobD [EC:6.3.1.10] | K02227 | 38 |
| Adenosylcobinamide kinase / adenosylcobinamide-phosphate guanylyltransferase [EC:2.7.1.156 2.7.7.62] | K02231 | 38 |
| Glycoside/pentoside/hexuronide:cation symporter, GPH family | K03292 | 38 |
| Regulatory protein | K03565 | 38 |
| Electron transport complex protein RnfG | K03612 | 38 |
| Threonine-phosphate decarboxylase [EC:4.1.1.81] | K04720 | 38 |
| Cardiolipin synthase [EC:2.7.8.-] | K06131 | 38 |
| Copper homeostasis protein | K06201 | 38 |
| Uncharacterized protein | K07011 | 38 |
| Uncharacterized protein | K07052 | 38 |
| D-alanyl-D-alanine carboxypeptidase (penicillin-binding protein 5/6) [EC:3.4.16.4] | K07258 | 38 |
| Isocitrate dehydrogenase [EC:1.1.1.42] | K00031 | 39 |
| Ornithine carbamoyltransferase [EC:2.1.3.3] | K00611 | 39 |
| citrate synthase [EC:2.3.3.1] | K01647 | 39 |
| aconitate hydratase 1 [EC:4.2.1.3] | K01681 | 39 |
| imidazoleglycerol-phosphate dehydratase [EC:4.2.1.19] | K01693 | 39 |
| RNA methyltransferase, TrmH family [EC:2.1.1.-] | K03218 | 39 |
| TatD DNase family protein [EC:3.1.21.-] | K03424 | 39 |
| Ribonuclease HI [EC:3.1.26.4] | K03469 | 39 |
| Uncharacterized protein | K07040 | 39 |
| NA | K07090 | 39 |
| sodium/proline symporter | K11928 | 39 |
| 3-hydroxybutyryl-CoA dehydrogenase [EC:1.1.1.157] | K00074 | 40 |
| Glycerol-1-phosphate dehydrogenase [NAD(P)] [EC:1.1.1.261] | K00096 | 40 |
| Acetyl-CoA C-acetyltransferase [EC:2.3.1.9] | K00626 | 40 |
| Fumarate hydratase subunit alpha [EC:4.2.1.2] | K01677 | 40 |
| Molybdate transport system substrate-binding protein | K02020 | 40 |
| cobalamin biosynthesis protein CbiG | K02189 | 40 |
| Molybdenum cofactor biosynthesis protein C | K03637 | 40 |
| Molybdenum cofactor biosynthesis protein | K03639 | 40 |
| Precorrin-4 C11-methyltransferase [EC:2.1.1.133] | K05936 | 40 |
| NA | K07033 | 40 |
| cytochrome bd-I oxidase subunit I [EC:1.10.3.-] | K00425 | 41 |
| 2prime,3prime-cyclic-nucleotide 2prime-phosphodiesterase [EC:3.1.4.16] | K01119 | 41 |
| beta-glucuronidase [EC:3.2.1.31] | K01195 | 41 |
| peptidyl-dipeptidase Dcp [EC:3.4.15.5] | K01284 | 41 |
| naphthoate synthase [EC:4.1.3.36] | K01661 | 41 |
| glucosamine-6-phosphate deaminase [EC:3.5.99.6] | K02564 | 41 |
| curved DNA-binding protein | K05516 | 41 |
| Uncharacterized protein | K06915 | 41 |
| enterochelin esterase and related enzymes | K07214 | 41 |
| anaerobic C4-dicarboxylate transporter DcuB | K07792 | 41 |
| tRNA (guanine-N1-)-methyltransferase [EC:2.1.1.31] | K00554 | 42 |
| queuine tRNA-ribosyltransferase [EC:2.4.2.29] | K00773 | 42 |
| glutamate 5-kinase [EC:2.7.2.11] | K00931 | 42 |
| peptidyl-tRNA hydrolase, PTH1 family [EC:3.1.1.29] | K01056 | 42 |
| Crossover junction endodeoxyribonuclease RuvC [EC:3.1.22.4] | K01159 | 42 |
| ribulose-phosphate 3-epimerase [EC:5.1.3.1] | K01783 | 42 |
| DNA primase [EC:2.7.7.-] | K02316 | 42 |
| DNA polymerase III subunit deltaprime [EC:2.7.7.7] | K02341 | 42 |
| DNA polymerase III subunit gamma/tau [EC:2.7.7.7] | K02343 | 42 |
| anaerobic ribonucleoside-triphosphate reductase activating protein [EC:1.97.1.4] | K04068 | 42 |
| Bacteria Firmicutes | *Acetoanaerobium* | 43 |
| Bacteria Firmicutes | *Aerococcus* | 43 |
| Bacteria Firmicutes | *Caldanaerobacter* | 43 |
| Bacteria Firmicutes | *Clostridioides* | 43 |
| Bacteria Firmicutes | *Clostridium* | 43 |
| Bacteria>Firmicutes | *Enterococcus* | 43 |
| Bacteria Actinobacteria | *Gardnerella* | 43 |
| Bacteria β-Proteobacteria | *Nitrosomonas* | 43 |
| Bacteria Firmicutes | *Peptoniphilus* | 43 |
| Bacteria Firmicutes | *Peptostreptococcus* | 43 |
| Bacteria γ-Proteobacteria | *Acinetobacter* | 44 |
| Bacteria γ-Proteobacteria | *Pasteurella* | 44 |
| Bacteria γ-Proteobacteria | *Photobacterium* | 44 |
| Bacteria γ-Proteobacteria | *Photorhabdus* | 44 |
| Bacteria γ-Proteobacteria | *Proteus* | 44 |
| Bacteria γ-Proteobacteria | *Pseudoalteromonas* | 44 |
| Bacteria γ-Proteobacteria | *Psychrobacter* | 44 |
| Bacteria γ-Proteobacteria | *Ruminobacter* | 44 |
| Bacteria β-Proteobacteria | *Taylorella* | 44 |
| 4-hydroxythreonine-4-phosphate dehydrogenase [EC:1.1.1.262] | K00097 | 45 |
| cytochrome bd-I oxidase subunit II [EC:1.10.3.-] | K00426 | 45 |
| alpha-L-fucosidase [EC:3.2.1.51] | K01206 | 45 |
| dipeptidyl-peptidase III [EC:3.4.14.4] | K01277 | 45 |
| acetylornithine deacetylase [EC:3.5.1.16] | K01438 | 45 |
| Lrp/AsnC family transcriptional regulator, regulator for asnA, asnC and gidA | K03718 | 45 |
| PhnP protein | K06167 | 45 |
| zinc protease [EC:3.4.99.-] | K07263 | 45 |
| PadR family transcriptional regulator, regulatory protein PadR | K10947 | 45 |
| Bacteria Firmicutes | *Acetohalobium* | 46 |
| Bacteria δ-Proteobacteria | *Desulfomonile* | 46 |
| Bacteria Firmicutes | *Flavonifractor* | 46 |
| Bacteria Firmicutes | *Intestinimonas* | 46 |
| Ribonucleoside-triphosphate reductase [EC:1.17.4.2] | K00527 | 46 |
| Bacteria Actinobacteria | *Nakamurella* | 46 |
| Bacteria Actinobacteria | *Neomicrococcus* | 46 |
| Bacteria Synergistetes | *Thermanaerovibrio* | 46 |
| Bacteria γ-Proteobacteria | *Thiolapillus* | 46 |
| 2-dehydropantoate 2-reductase [EC:1.1.1.169] | K00077 | 47 |
| acetylglutamate/acetylaminoadipate kinase [EC:2.7.2.8 2.7.2.-] | K00930 | 47 |
| repressor LexA [EC:3.4.21.88] | K01356 | 47 |
| Cystathionine beta-lyase [EC:4.4.1.8] | K01760 | 47 |
| bile acid:Na+ symporter, BASS family | K03453 | 47 |
| nucleobase:cation symporter-2, NCS2 family | K03458 | 47 |
| Probable rRNA maturation factor | K07042 | 47 |
| transcriptional repressor NrdR | K07738 | 47 |
| hypothetical protein | K09117 | 47 |
| Dihydroorotate oxidase [EC:1.3.3.1] | K00226 | 48 |
| tRNA dimethylallyltransferase [EC:2.5.1.75] | K00791 | 48 |
| nicotinate-nucleotide adenylyltransferase [EC:2.7.7.18] | K00969 | 48 |
| peptide deformylase [EC:3.5.1.88] | K01462 | 48 |
| Ribosome-binding factor A | K02834 | 48 |
| 16S rRNA processing protein RimM | K02860 | 48 |
| UPF0079 ATP-binding protein | K06925 | 48 |
| 16S rRNA (cytidine1402-2'-O)-methyltransferase [EC:2.1.1.198] | K07056 | 48 |
| thymidylate synthase [EC:2.1.1.45] | K00560 | 49 |
| phosphoribosylaminoimidazolecarboxamide formyltransferase / IMP cyclohydrolase [EC:2.1.2.3 3.5.4.10] | K00602 | 49 |
| methionyl aminopeptidase [EC:3.4.11.18] | K01265 | 49 |
| F-type H+-transporting ATPase subunit delta [EC:3.6.3.14] | K02113 | 49 |
| multidrug resistance protein A | K03543 | 49 |
| putative sigma-54 modulation protein | K05808 | 49 |
| putative endopeptidase [EC:3.4.24.-] | K07386 | 49 |
| Fe-S cluster assembly protein SufD | K09015 | 49 |
| [acyl-carrier-protein] S-malonyltransferase [EC:2.3.1.39] | K00645 | 50 |
| 3-oxoacyl-[acyl-carrier-protein] synthase III [EC:2.3.1.180] | K00648 | 50 |
| aspartate aminotransferase [EC:2.6.1.1] | K00812 | 50 |
| imidazoleglycerol-phosphate dehydratase / histidinol-phosphatase [EC:4.2.1.19 3.1.3.15] | K01089 | 50 |
| alpha-N-arabinofuranosidase [EC:3.2.1.55] | K01209 | 50 |
| FKBP-type peptidyl-prolyl cis-trans isomerase FklB [EC:5.2.1.8] | K03773 | 50 |
| outer membrane protein | K06142 | 50 |
| regulator of sigma E protease [EC:3.4.24.-] | K11749 | 50 |
| glycerol-3-phosphate dehydrogenase (NAD(P)+) [EC:1.1.1.94] | K00057 | 51 |
| glutamate racemase [EC:5.1.1.3] | K01776 | 51 |
| glutamyl-tRNA synthetase [EC:6.1.1.17] | K01885 | 51 |
| peptide chain release factor RF-2 | K02836 | 51 |
| metal ion transporter, MIT family | K03284 | 51 |
| trigger factor | K03545 | 51 |
| GTP-binding protein | K03977 | 51 |
| ribosomal large subunit pseudouridine synthase D [EC:5.4.99.12] | K06180 | 51 |
| Archaea Euryarchaeota | Candidatus *Halobonum* | 52 |
| Bacteria Firmicutes | *Dehalobacter* | 52 |
| Bacteria γ-Proteobacteria | *Hahella* | 52 |
| Bacteria Actinobacteria | *Kineococcus* | 52 |
| Bacteria Firmicutes | *Lentibacillus* | 52 |
| Bacteria Firmicutes | *Mahella* | 52 |
| Bacteria Firmicutes | *Parageobacillus* | 52 |
| Bacteria α-Proteobacteria | *Phenylobacterium* | 52 |
| Glycolate oxidase [EC:1.1.3.15] | K00104 | 53 |
| Glycerol-3-phosphate dehydrogenase [EC:1.1.5.3] | K00111 | 53 |
| Succinate-semialdehyde dehydrogenase (NADP+) [EC:1.2.1.16] | K00135 | 53 |
| Glutamate dehydrogenase [EC:1.4.1.2] | K00260 | 53 |
| Gamma-glutamyltranspeptidase / glutathione hydrolase [EC:2.3.2.2 3.4.19.13] | K00681 | 53 |
| Glucarate dehydratase [EC:4.2.1.40] | K01706 | 53 |
| Uncharacterized protein | K07138 | 53 |
| Pantetheine-phosphate adenylyltransferase [EC:2.7.7.3] | K00954 | 54 |
| Iron complex transport system ATP-binding protein [EC:3.6.3.34] | K02013 | 54 |
| LacI family transcriptional regulator | K02529 | 54 |
| Type III pantothenate kinase [EC:2.7.1.33] | K03525 | 54 |
| Ribonuclease P protein component [EC:3.1.26.5] | K03536 | 54 |
| A/G-specific adenine glycosylase [EC:3.2.2.-] | K03575 | 54 |
| Hemolysin III | K11068 | 54 |
| Fructokinase [EC:2.7.1.4] | K00847 | 55 |
| 2-C-methyl-D-erythritol 4-phosphate cytidylyltransferase [EC:2.7.7.60] | K00991 | 55 |
| Putative ABC transport system permease protein | K02004 | 55 |
| Phosphate transport system substrate-binding protein | K02040 | 55 |
| ATP-dependent Clp protease ATP-binding subunit ClpC | K03696 | 55 |
| DNA repair protein RadA/Sms | K04485 | 55 |
| DNA recombination protein RmuC | K09760 | 55 |
| Bacteria Proteobacteria | Candidatus *Babela* | 56 |
| Bacteria α-Proteobacteria | *Ehrlichia* | 56 |
| Bacteria Tenericutes | *Mesoplasma* | 56 |
| Bacteria Bacteroidetes | *Polaribacter* | 56 |
| Bacteria Tenericutes | *Spiroplasma* | 56 |
| Bacteria Thermodesulfobacteria | *Thermodesulfobacterium* | 56 |
| Bacteria Thermotogae | *Thermosipho* | 56 |
| 6-phosphofructokinase [EC:2.7.1.11] | K00850 | 57 |
| UDP-N-acetylmuramoylalanyl-D-glutamyl-2,6-diaminopimelate--D-alanyl-D-alanine ligase [EC:6.3.2.10] | K01929 | 57 |
| DNA ligase (NAD+) [EC:6.5.1.2] | K01972 | 57 |
| DNA polymerase III subunit delta [EC:2.7.7.7] | K02340 | 57 |
| glutamine amidotransferase [EC:2.4.2.-] | K02501 | 57 |
| cell division protein FtsI (penicillin-binding protein 3) [EC:2.4.1.129] | K03587 | 57 |
| ribonuclease III [EC:3.1.26.3] | K03685 | 57 |
| rhamnulokinase [EC:2.7.1.5] | K00848 | 58 |
| pectinesterase [EC:3.1.1.11] | K01051 | 58 |
| levanase [EC:3.2.1.65] | K01212 | 58 |
| mannose-6-phosphate isomerase [EC:5.3.1.8] | K01809 | 58 |
| 2-succinyl-5-enolpyruvyl-6-hydroxy-3-cyclohexene-1-carboxylate synthase [EC:2.2.1.9] | K02551 | 58 |
| L-rhamnose mutarotase [EC:5.1.3.-] | K03534 | 58 |
| type II pantothenate kinase [EC:2.7.1.33] | K09680 | 58 |
| arginine deiminase [EC:3.5.3.6] | K01478 | 59 |
| DNA integrity scanning protein | K07067 | 59 |
| lipopolysaccharide transport system ATP-binding protein | K09691 | 59 |
| putrescine carbamoyltransferase [EC:2.1.3.6] | K13252 | 59 |
| Bacteria Actinobacteria | *Olsenella* | 59 |
| Bacteria>Firmicutes | *Sharpea* | 59 |
| Peptide/nickel transport system permease protein | K02034 | 60 |
| aspartyl-tRNA(Asn)/glutamyl-tRNA (Gln) amidotransferase subunit A [EC:6.3.5.6 6.3.5.7] | K02433 | 60 |
| Aspartyl-tRNA(Asn)/glutamyl-tRNA (Gln) amidotransferase subunit B [EC:6.3.5.6 6.3.5.7] | K02434 | 60 |
| Aspartyl-tRNA(Asn)/glutamyl-tRNA (Gln) amidotransferase subunit C [EC:6.3.5.6 6.3.5.7] | K02435 | 60 |
| Excinuclease ABC subunit B | K03702 | 60 |
| two-component system, OmpR family, response regulator RegX3 | K07776 | 60 |
| Ribonuclease PH [EC:2.7.7.56] | K00989 | 61 |
| peptidylprolyl isomerase [EC:5.2.1.8] | K01802 | 61 |
| Na+:H+ antiporter, NhaA family | K03313 | 61 |
| Ribonuclease D [EC:3.1.13.5] | K03684 | 61 |
| Putative thioredoxin | K05838 | 61 |
| Phosphate acetyltransferase [EC:2.3.1.8] | K13788 | 61 |
| Bacteria Bacteroidetes | *Capnocytophaga* | 62 |
| Bacteria Bacteroidetes | *Draconibacterium* | 62 |
| Bacteria Bacteroidetes | *Flammeovirga* | 62 |
| Bacteria Bacteroidetes | *Mucinivorans* | 62 |
| Bacteria Bacteroidetes | *Parabacteroides* | 62 |
| Bacteria Bacteroidetes | *Tannerella* | 62 |
| Algae Stramenopiles | *Aureococcus* | 63 |
| Bacteria Acidobacteria | Candidatus *Solibacter* | 63 |
| Plantae Magnolophyta | *Fuerstia* | 63 |
| Bacteria Actinobacteria | *Ilumatobacter* | 63 |
| Bacteria Kiritimatiellaeota | *Kiritimatiella* | 63 |
| Bacteria Planctomycetes | *Planctomyces* | 63 |
| Bacteria Tenericutes | Candidatus *Izimaplasma* | 64 |
| Bacteria Firmicutes | *Gottschalkia* | 64 |
| Bacteria Firmicutes | *Listeria* | 64 |
| Bacteria Firmicutes | *Parvimonas* | 64 |
| Bacteria Firmicutes | *Rummeliibacillus* | 64 |
| Bacteria Firmicutes | *Turicibacter* | 64 |
| Fungi Ascomycota | *Cladophialophora* | 65 |
| Protist Alveolata Apicomplexa | *Hammondia* | 65 |
| Euglenozoa> Kinetoplastida | *Leishmania* | 65 |
| Bacteria Cyanobacteria | *Leptolyngbya* | 65 |
| Fungi Ascomycota | *Neofusicoccum* | 65 |
| Tetrahydromethanopterin S-methyltransferase subunit B [EC:2.1.1.86] | K00578 | 66 |
| Glycerol-3-phosphate cytidylyltransferase [EC:2.7.7.39] | K00980 | 66 |
| Ribosomal RNA large subunit methyltransferase E [EC:2.1.1.-] | K02427 | 66 |
| Small subunit ribosomal protein S27Ae | K02977 | 66 |
| DNA-directed RNA polymerase subunit L [EC:2.7.7.6] | K03056 | 66 |
| Bacteria Firmicutes | *Amphibacillus* | 67 |
| Bacteria Thermotogae | *Defluviitoga* | 67 |
| Bacteria Firmicutes | *Gemella* | 67 |
| Bacteria Firmicutes | *Melissococcus* | 67 |
| Bacteria Firmicutes | *Staphylococcus* | 67 |
| Formate--tetrahydrofolate ligase [EC:6.3.4.3] | K01938 | 68 |
| Peptide/nickel transport system permease protein | K02033 | 68 |
| Glycerol uptake facilitator protein | K02440 | 68 |
| Putative hemolysin | K03699 | 68 |
| Serine/threonine transporter | K07862 | 68 |
| Bacteria Bacteroidetes | *Dyadobacter* | 69 |
| Bacteria Bacteroidetes | *Haliscomenobacter* | 69 |
| Epsilonproteobacteria | *Nitratifractor* | 69 |
| Bacteria Bacteroidetes | *Pontibacter* | 69 |
| Bacteria Bacteroidetes | *Rufibacter* | 69 |
| glycine dehydrogenase subunit 1 [EC:1.4.4.2] | K00282 | 70 |
| thymidine kinase [EC:2.7.1.21] | K00857 | 70 |
| tetraacyldisaccharide 4prime-kinase [EC:2.7.1.130] | K00912 | 70 |
| NA | K06885 | 70 |
| MEMO1 family protein | K06990 | 70 |
| NAD+ synthase (glutamine-hydrolysing) [EC:6.3.5.1] | K01950 | 71 |
| MraZ protein | K03925 | 71 |
| diacylglycerol kinase (ATP) [EC:2.7.1.107] | K07029 | 71 |
| putative ATPase | K07478 | 71 |
| D-tyrosyl-tRNA(Tyr) deacylase [EC:3.1.-.-] | K07560 | 71 |
| NAD+ kinase [EC:2.7.1.23] | K00858 | 72 |
| pyridoxine kinase [EC:2.7.1.35] | K00868 | 72 |
| dihydrodipicolinate synthase [EC:4.2.1.52] | K01714 | 72 |
| cyclase [EC:4.1.3.-] | K02500 | 72 |
| tRNA pseudouridine synthase A [EC:5.4.99.12] | K06173 | 72 |
| Bacteria Bacteroidetes | *Alistipes* | 73 |
| Bacteria Gemmatimonadetes | *Gemmatimonas* | 73 |
| Bacteria Bacteroidetes | *Hymenobacter* | 73 |
| Bacteria α-Proteobacteria | *Maricaulis* | 73 |
| Bacteria Bacteroidetes | *Spirosoma* | 73 |
| GDPmannose 4,6-dehydratase [EC:4.2.1.47] | K01711 | 74 |
| uroporphyrinogen-III synthase [EC:4.2.1.75] | K01719 | 74 |
| putative family 31 glucosidase | K01811 | 74 |
| aquaporin Z | K06188 | 74 |
| MFS transporter, PAT family, beta-lactamase induction signal transducer AmpG | K08218 | 74 |
| Bacteria Spirochaetes | *Brachyspira* | 75 |
| Bacteria Proteobacteria | Candidatus *Mikella* | 75 |
| Bacteria Proteobacteria | Candidatus *Profftella* | 75 |
| Bacteria Deferribacteres | *Deferribacter* | 75 |
| large subunit ribosomal protein L13 | K02871 | 76 |
| large subunit ribosomal protein L22 | K02890 | 76 |
| large subunit ribosomal protein L24 | K02895 | 76 |
| Small subunit ribosomal protein S9 | K02996 | 76 |
| Bacteria Cyanobacteria | *Arthrospira* | 77 |
| Bacteria Cyanobacteria | *Crinalium* | 77 |
| Bacteria Bacteroidetes | *Flavobacterium* | 77 |
| Bacteria Bacteroidetes | *Paludibacter* | 77 |
| Butyryl-CoA dehydrogenase [EC:1.3.99.2] | K00248 | 78 |
| Oxaloacetate decarboxylase, alpha subunit [EC:4.1.1.3] | K01571 | 78 |
| Flagellin | K02406 | 78 |
| Methyl-accepting chemotaxis protein | K03406 | 78 |
| MFS transporter, FHS family, L-fucose permease | K02429 | 79 |
| monovalent cation:H+ antiporter-2, CPA2 family | K03455 | 79 |
| integration host factor subunit alpha | K04764 | 79 |
| LAO/AO transport system kinase [EC:2.7.-.-] | K07588 | 79 |
| Bacteria β-Proteobacteria | *Collimonas* | 80 |
| Archaea Euryarchaeota | *Halomicrobium* | 80 |
| Bacteria Firmicutes | *Kyrpidia* | 80 |
| Bacteria Cyanobacteria | *Synechocystis* | 80 |
| Bacteria Tenericutes | *Acholeplasma* | 81 |
| Bacteria Firmicutes | *Tetragenococcus* | 81 |
| Bacteria Firmicutes | *Vagococcus* | 81 |
| Bacteria Firmicutes | *Weissella* | 81 |
| nicotinate-nucleotide--dimethylbenzimidazole phosphoribosyltransferase [EC:2.4.2.21] | K00768 | 82 |
| diacylglycerol kinase (ATP) [EC:2.7.1.107] | K02015 | 82 |
| fructose-1,6-bisphosphatase III [EC:3.1.3.11] | K04041 | 82 |
| uncharacterized protein | K07095 | 82 |
| Bacteria Bacteroidetes | *Algoriphagus* | 83 |
| Bacteria Bacteroidetes | *Owenweeksia* | 83 |
| Bacteria Bacteroidetes | *Runella* | 83 |
| Bacteria Bacteroidetes | *Solitalea* | 83 |
| beta-glucosidase [EC:3.2.1.21] | K01188 | 84 |
| rhamnulose-1-phosphate aldolase [EC:4.1.2.19] | K01629 | 84 |
| PTS system, galactitol-specific IIC component | K02775 | 84 |
| MFS transporter, SP family, inositol transporter | K06610 | 84 |
| 1-phosphofructokinase [EC:2.7.1.56] | K00882 | 85 |
| Peptide/nickel transport system ATP-binding protein | K02032 | 85 |
| Chromosome segregation protein | K03529 | 85 |
| agmatine deiminase [EC:3.5.3.12] | K10536 | 85 |
| Shikimate kinase [EC:2.7.1.71] | K00891 | 86 |
| purine-nucleoside phosphorylase [EC:2.4.2.1] | K03783 | 86 |
| Chromate transporter | K07240 | 86 |
| DNA mismatch repair protein MutS2 | K07456 | 86 |
| Glutamate synthase (ferredoxin) [EC:1.4.7.1] | K00284 | 87 |
| Glycerol kinase [EC:2.7.1.30] | K00864 | 87 |
| Adenosine deaminase [EC:3.5.4.4] | K01488 | 87 |
| Carbohydrate diacid regulator | K02647 | 87 |
| Bacteria γ-Proteobacteria | *Buchnera* | 88 |
| Bacteria Proteobacteria | Candidatus *Carsonella* | 88 |
| Bacteria γ-Proteobacteria | Candidatus *Tachikawaea* | 88 |
| Bacteria γ-Proteobacteria | *Wigglesworthia* | 88 |
| Bacteria δ-Proteobacteria | *Anaeromyxobacter* | 89 |
| Bacteria δ-Proteobacteria | *Sandaracinus* | 89 |
| Bacteria Actinobacteria | *Sinomonas* | 89 |
| Bacteria β-Proteobacteria | *Thiobacillus* | 89 |
| isoleucyl-tRNA synthetase [EC:6.1.1.5] | K01870 | 90 |
| Aspartyl-tRNA synthetase [EC:6.1.1.12] | K01876 | 90 |
| GMP synthase (glutamine-hydrolysing) [EC:6.3.5.2] | K01951 | 90 |
| Carbamoyl-phosphate synthase large subunit [EC:6.3.5.5] | K01955 | 90 |
| superoxide dismutase, Fe-Mn family [EC:1.15.1.1] | K04564 | 91 |
| protease IV [EC:3.4.21.-] | K04773 | 91 |
| NA | K07001 | 91 |
| D-alanyl-D-alanine carboxypeptidase / D-alanyl-D-alanine-endopeptidase (penicillin-binding protein 4) [EC:3.4.16.4 3.4.99.-] | K07259 | 91 |
| dihydroorotase [EC:3.5.2.3] | K01465 | 92 |
| S-adenosyl-methyltransferase [EC:2.1.1.-] | K03438 | 92 |
| hypothetical protein | K09710 | 92 |
| diaminohydroxyphosphoribosylaminopyrimidine deaminase / 5-amino-6-(5-phosphoribosylamino)uracil reductase [EC:3.5.4.26 1.1.1.193] | K11752 | 92 |
| dTDP-4-dehydrorhamnose reductase [EC:1.1.1.133] | K00067 | 93 |
| Putative ABC transport system ATP-binding protein | K02065 | 93 |
| Glycosyltransferase 2 family protein | K07027 | 93 |
| outer membrane channel protein TolC | K12340 | 93 |
| Alcohol dehydrogenase [EC:1.1.1.1] | K00001 | 94 |
| Beta-N-acetylhexosaminidase [EC:3.2.1.52] | K01207 | 94 |
| Competence protein ComEC | K02238 | 94 |
| D-alanyl-D-alanine dipeptidase [EC:3.4.13.-] | K08641 | 94 |
| Archaea Crenarchaeota | *Caldisphaera* | 95 |
| Bacteria Bacteroidetes | *Marivirga* | 95 |
| Bacteria Bacteroidetes | *Psychroflexus* | 95 |
| Bacteria Bacteroidetes | *Zunongwangia* | 95 |
| thiamine-phosphate pyrophosphorylase [EC:2.5.1.3] | K00788 | 96 |
| hydroxymethylpyrimidine/phosphomethylpyrimidine kinase [EC:2.7.1.49 2.7.4.7] | K00941 | 96 |
| Thiamine biosynthesis ThiG | K03149 | 96 |
| thiamine biosynthesis ThiH | K03150 | 96 |
| N-acetyl-gamma-glutamyl-phosphate/N-acetyl-gamma-aminoadipyl-phosphate reductase [EC:1.2.1.38 1.2.1.-] | K00145 | 97 |
| Acetolactate synthase I/II/III large subunit [EC:2.2.1.6] | K01652 | 97 |
| NAD+ diphosphatase [EC:3.6.1.22] | K03426 | 97 |
| putative protease [EC:3.4.-.-] | K08303 | 97 |
| Bacteria Calditrichaeota | Caldithrix | 98 |
| Bacteria Bacteroidetes | *Maribacter* | 98 |
| Bacteria Bacteroidetes | *Porphyromonas* | 98 |
| Bacteria Bacteroidetes | *Zobellia* | 98 |
| acetylornithine aminotransferase [EC:2.6.1.11] | K00818 | 99 |
| dipeptidyl-peptidase 4 [EC:3.4.14.5] | K01278 | 99 |
| prephenate dehydratase [EC:4.2.1.51] | K01713 | 99 |
| NA | K06890 | 99 |
| amidophosphoribosyltransferase [EC:2.4.2.14] | K00764 | 100 |
| O-acetylhomoserine (thiol)-lyase [EC:2.5.1.49] | K01740 | 100 |
| phenylalanyl-tRNA synthetase beta chain [EC:6.1.1.20] | K01890 | 100 |
| MoxR-like ATPase [EC:3.6.3.-] | K03924 | 100 |
| Bacteria δ-Proteobacteria | *Haliangium* | 101 |
| Bacteria α-Proteobacteria | *Parvibaculum* | 101 |
| Bacteria Planctomycetes | *Phycisphaera* | 101 |
| Bacteria Bacteroidetes | *Salinibacter* | 101 |
| tRNA Gln | K14223 | 102 |
| tRNA Glu | K14224 | 102 |
| tRNA Gly | K14225 | 102 |
| tRNA Pro | K14232 | 102 |
| Malate dehydrogenase (oxaloacetate-decarboxylating) [EC:1.1.1.38] | K00027 | 103 |
| Agmatinase [EC:3.5.3.11] | K01480 | 103 |
| Septum site-determining protein MinD | K03609 | 103 |
| CMP-binding protein | K03698 | 103 |
| Cytidine deaminase [EC:3.5.4.5] | K01489 | 104 |
| 4-methyl-5(b-hydroxyethyl)-thiazole monophosphate biosynthesis | K03152 | 104 |
| Conserved Hypothetical protein | K05810 | 104 |
| Uncharacterized protein | K07043 | 104 |
| Bacteria γ-Proteobacteria | *Aliivibrio* | No Class |
| Bacteria Firmicutes | *Alkaliphilus* | No Class |
| Bacteria Actinobacteria | *Atopobium* | No Class |
| Fungi Basidiomycota | *Auricularia* | No Class |
| Bacteria Bacteroidetes | *Barnesiella* | No Class |
| Bacteria α-Proteobacteria | *Bartonella* | No Class |
| Fungi Chytridiomycota | *Batrachochytrium* | No Class |
| Bacteria γ-Proteobacteria | *Bibersteinia* | No Class |
| Bacteria Firmicutes | *Brevibacillus* | No Class |
| Bacteria Actinobacteria | *Brevibacterium* | No Class |
| Bacteria Firmicutes | *Butyrivibrio* | No Class |
| Bacteria Bacteroidetes | Candidatus *Amoebophilus* | No Class |
| Bacteria Bacteroidetes | Candidatus *Azobacteroides* | No Class |
| Bacteria Bacteroidetes | Candidatus *Cardinium* | No Class |
| Bacteria Proteobacteria | Candidatus *Desulfofervidus* | No Class |
| Bacteria Proteobacteria | Candidatus *Liberibacter* | No Class |
| Archaea Euryarchaeota | Candidatus *Methanoplasma* | No Class |
| Bacteria Proteobacteria | Candidatus *Pelagibacter* | No Class |
| Bacteria Tenericutes | Candidatus *Phytoplasma* | No Class |
| Bacteria Bacteroidetes | Candidatus *Sulcia* | No Class |
| Fungi Ascomycota | *Capronia* | No Class |
| Bacteria Cyanobacteria | *Chamaesiphon* | No Class |
| Bacteria Bacteroidetes | *Chitinophaga* | No Class |
| Bacteria Cyanobacteria | *Chroococcidiopsis* | No Class |
| Bacteria Actinobacteria | *Conexibacter* | No Class |
| Fungi Basidiomycota | *Coniophora* | No Class |
| Bacteria Firmicutes | *Coprothermobacter* | No Class |
| Bacteria Verrucomicrobia | *Coraliomargarita* | No Class |
| Bacteria Bacteroidetes | *Croceibacter* | No Class |
| Bacteria Actinobacteria | *Cryptobacterium* | No Class |
| Fungi Basidiomycota | *Cutaneotrichosporon* | No Class |
| Bacteria Cyanobacteria | *Cyanothece* | No Class |
| Bacteria Bacteroidetes | *Cyclobacterium* | No Class |
| Bacteria γ-Proteobacteria | *Cycloclasticus* | No Class |
| Bacteria Bacteroidetes | *Cytophaga* | No Class |
| Bacteria Chloroflexi | *Dehalogenimonas* | No Class |
| Bacteria Deferribacteres | *Denitrovibrio* | No Class |
| Bacteria δ-Proteobacteria | *Desulfatibacillum* | No Class |
| Bacteria Bacteroidetes | *Echinicola* | No Class |
| Chromista Haptophyta | *Emiliania* | No Class |
| Fungi Ascomycota | *Endocarpon* | No Class |
| Bacteria Elusimicrobia | *Endomicrobium* | No Class |
| Fungi Ascomycota | *Exophiala* | No Class |
| Bacteria Bacteroidetes | *Fermentimonas* | No Class |
| Bacteria Bacteroidetes | *Fibrella* | No Class |
| Bacteria Firmicutes | *Fictibacillus* | No Class |
| Bacteria Firmicutes | *Filifactor* | No Class |
| Bacteria Armatimonadetes | *Fimbriimonas* | No Class |
| Bacteria Firmicutes | *Finegoldia* | No Class |
| Fungi Ascomycota | *Fonsecaea* | No Class |
| Bacteria Bacteroidetes | *Formosa* | No Class |
| Bacteria γ-Proteobacteria | *Francisella* | No Class |
| Bacteria Cyanobacteria | *Geminocystis* | No Class |
| Bacteria Firmicutes | *Geosporobacter* | No Class |
| Bacteria α-Proteobacteria | *Gluconacetobacter* | No Class |
| Bacteria Actinobacteria | *Glutamicibacter* | No Class |
| Bacteria Actinobacteria | *Gordonibacter* | No Class |
| Bacteria γ-Proteobacteria | *Grimontia* | No Class |
| Algae Cryptophyta | *Guillardia* | No Class |
| Bacteria γ-Proteobacteria | *Hafnia* | No Class |
| Archaea Euryarchaeota | *Halalkalicoccus* | No Class |
| Bacteria Proteobacteria | *Halobacteriovorax* | No Class |
| Archaea Euryarchaeota | *Halobacterium* | No Class |
| Archaea Euryarchaeota | *Halococcus* | No Class |
| Archaea Euryarchaeota | *Halosimplex* | No Class |
| Archaea Euryarchaeota | *Haloterrigena* | No Class |
| Bacteria Fusobacteria | *Ilyobacter* | No Class |
| Gluconate 5-dehydrogenase [EC:1.1.1.69] | K00046 | No Class |
| 1-deoxy-D-xylulose-5-phosphate reductoisomerase [EC:1.1.1.267] | K00099 | No Class |
| alanine dehydrogenase [EC:1.4.1.1] | K00259 | No Class |
| ribonucleoside-diphosphate reductase alpha chain [EC:1.17.4.1] | K00525 | No Class |
| ribonucleoside-diphosphate reductase beta chain [EC:1.17.4.1] | K00526 | No Class |
| Ferredoxin--NADP+ reductase [EC:1.18.1.2] | K00528 | No Class |
| 5-methyltetrahydrofolate--homocysteine methyltransferase [EC:2.1.1.13] | K00548 | No Class |
| tRNA-specific 2-thiouridylase [EC:2.8.1.-] | K00566 | No Class |
| Methylated-DNA-[protein]-cysteine S-methyltransferase [EC:2.1.1.63] | K00567 | No Class |
| Precorrin-6Y C5,15-methyltransferase / precorrin-8W decarboxylase [EC:2.1.1.132 1.-.-.-] | K00595 | No Class |
| Methyltransferase-like protein 6 [EC:2.1.1.-] | K00599 | No Class |
| aminomethyltransferase [EC:2.1.2.10] | K00605 | No Class |
| Aspartate carbamoyltransferase catalytic subunit [EC:2.1.3.2] | K00609 | No Class |
| Aspartate carbamoyltransferase regulatory subunit | K00610 | No Class |
| serine O-acetyltransferase [EC:2.3.1.30] | K00640 | No Class |
| Homoserine O-succinyltransferase [EC:2.3.1.46] | K00651 | No Class |
| Diamine N-acetyltransferase [EC:2.3.1.57] | K00657 | No Class |
| starch synthase [EC:2.4.1.21] | K00703 | No Class |
| adenine phosphoribosyltransferase [EC:2.4.2.7] | K00759 | No Class |
| Uracil phosphoribosyltransferase [EC:2.4.2.9] | K00761 | No Class |
| Orotate phosphoribosyltransferase [EC:2.4.2.10] | K00762 | No Class |
| ATP phosphoribosyltransferase [EC:2.4.2.17] | K00765 | No Class |
| Ribonuclease Z [EC:3.1.26.11] | K00784 | No Class |
| S-adenosylmethionine synthetase [EC:2.5.1.6] | K00789 | No Class |
| Riboflavin synthase beta chain [EC:2.5.1.-] | K00794 | No Class |
| Spermidine synthase [EC:2.5.1.16] | K00797 | No Class |
| 3-phosphoshikimate 1-carboxyvinyltransferase [EC:2.5.1.19] | K00800 | No Class |
| Acetylornithine/N-succinyldiaminopimelate aminotransferase [EC:2.6.1.11 2.6.1.17] | K00821 | No Class |
| ribokinase [EC:2.7.1.15] | K00852 | No Class |
| aspartate kinase [EC:2.7.2.4] | K00928 | No Class |
| adenylate kinase [EC:2.7.4.3] | K00939 | No Class |
| dTMP kinase [EC:2.7.4.9] | K00943 | No Class |
| Cytidylate kinase [EC:2.7.4.14] | K00945 | No Class |
| GTP pyrophosphokinase [EC:2.7.6.5] | K00951 | No Class |
| UTP--glucose-1-phosphate uridylyltransferase [EC:2.7.7.9] | K00963 | No Class |
| Glucose-1-phosphate adenylyltransferase [EC:2.7.7.27] | K00975 | No Class |
| streptomycin 3prime-adenylyltransferase [EC:2.7.7.47] | K00984 | No Class |
| type III restriction enzyme [EC:3.1.21.5] | K01156 | No Class |
| Beta-galactosidase [EC:3.2.1.23] | K01190 | No Class |
| Beta-fructofuranosidase [EC:3.2.1.26] | K01193 | No Class |
| Arabinogalactan endo-1,4-beta-galactosidase [EC:3.2.1.89] | K01224 | No Class |
| alpha-glucuronidase [EC:3.2.1.139] | K01235 | No Class |
| Aminopeptidase N [EC:3.4.11.2] | K01256 | No Class |
| Tripeptide aminopeptidase [EC:3.4.11.4] | K01258 | No Class |
| aminopeptidase [EC:3.4.11.-] | K01269 | No Class |
| Bleomycin hydrolase [EC:3.4.22.40] | K01372 | No Class |
| Choloylglycine hydrolase [EC:3.5.1.24] | K01442 | No Class |
| dCMP deaminase [EC:3.5.4.12] | K01493 | No Class |
| Phosphoribosyl-AMP cyclohydrolase [EC:3.5.4.19] | K01496 | No Class |
| Cu2+-exporting ATPase [EC:3.6.3.4] | K01533 | No Class |
| Cd2+/Zn2+-exporting ATPase [EC:3.6.3.3 3.6.3.5] | K01534 | No Class |
| aspartate 1-decarboxylase [EC:4.1.1.11] | K01579 | No Class |
| diaminopimelate decarboxylase [EC:4.1.1.20] | K01586 | No Class |
| orotidine-5prime-phosphate decarboxylase [EC:4.1.1.23] | K01591 | No Class |
| Indole-3-glycerol phosphate synthase [EC:4.1.1.48] | K01609 | No Class |
| acetolactate synthase I/III small subunit [EC:2.2.1.6] | K01653 | No Class |
| Anthranilate synthase component I [EC:4.1.3.27] | K01657 | No Class |
| tryptophan synthase alpha chain [EC:4.2.1.20] | K01695 | No Class |
| 3-dehydroquinate synthase [EC:4.2.3.4] | K01735 | No Class |
| chorismate synthase [EC:4.2.3.5] | K01736 | No Class |
| hydroxymethylbilane synthase [EC:2.5.1.61] | K01749 | No Class |
| alanine racemase [EC:5.1.1.1] | K01775 | No Class |
| xylose isomerase [EC:5.3.1.5] | K01805 | No Class |
| phosphoribosylformimino-5-aminoimidazole carboxamide ribotide isomerase [EC:5.3.1.16] | K01814 | No Class |
| Phosphopentomutase [EC:5.4.2.7] | K01839 | No Class |
| glutaminyl-tRNA synthetase [EC:6.1.1.18] | K01886 | No Class |
| long-chain acyl-CoA synthetase [EC:6.2.1.3] | K01897 | No Class |
| glutamine synthetase [EC:6.3.1.2] | K01915 | No Class |
| Phosphoribosylamine--glycine ligase [EC:6.3.4.13] | K01945 | No Class |
| Propionyl-CoA carboxylase beta chain [EC:6.4.1.3] | K01966 | No Class |
| ABC-2 type transport system permease protein | K01992 | No Class |
| Cobalt/nickel transport system ATP-binding protein | K02006 | No Class |
| ribosome-binding ATPase | K02008 | No Class |
| multiple sugar transport system substrate-binding protein | K02027 | No Class |
| Polar amino acid transport system ATP-binding protein [EC:3.6.3.21] | K02028 | No Class |
| polar amino acid transport system substrate-binding protein | K02030 | No Class |
| sulfonate/nitrate/taurine transport system substrate-binding protein | K02051 | No Class |
| Putative ABC transport system permease protein | K02066 | No Class |
| putative ABC transport system permease protein | K02069 | No Class |
| Adenosylcobyric acid synthase [EC:6.3.5.10] | K02232 | No Class |
| Pilus assembly protein CpaF | K02283 | No Class |
| DNA polymerase I [EC:2.7.7.7] | K02335 | No Class |
| ElaA protein | K02348 | No Class |
| glycine cleavage system H protein | K02437 | No Class |
| 16S rRNA (adenine1518-N6/adenine1519-N6)-dimethyltransferase [EC:2.1.1.182] | K02528 | No Class |
| 1,4-dihydroxy-2-naphthoate octaprenyltransferase [EC:2.5.1.74] | K02548 | No Class |
| O-succinylbenzoate synthase [EC:4.2.1.113] | K02549 | No Class |
| topoisomerase IV subunit A [EC:5.99.1.-] | K02621 | No Class |
| PTS system, mannose-specific IIC component | K02795 | No Class |
| PTS system, mannose-specific IID component | K02796 | No Class |
| Dihydroorotate dehydrogenase electron transfer subunit | K02823 | No Class |
| large subunit ribosomal protein L10 | K02864 | No Class |
| Large subunit ribosomal protein L29 | K02904 | No Class |
| large subunit ribosomal protein L30 | K02907 | No Class |
| Small subunit ribosomal protein S15 | K02956 | No Class |
| Preprotein translocase subunit SecD | K03072 | No Class |
| Preprotein translocase subunit SecF | K03074 | No Class |
| preprotein translocase subunit SecG | K03075 | No Class |
| RNA polymerase sigma-70 factor, ECF subfamily | K03088 | No Class |
| RNA polymerase sporulation-specific sigma factor | K03091 | No Class |
| sulfur carrier protein | K03154 | No Class |
| tRNA pseudouridine synthase B [EC:5.4.99.12] | K03177 | No Class |
| Type IV secretion system protein VirD4 | K03205 | No Class |
| chloride channel protein, CIC family | K03281 | No Class |
| large conductance mechanosensitive channel | K03282 | No Class |
| MFS transporter, NHS family, nucleoside permease | K03289 | No Class |
| lactate transporter, LctP family | K03303 | No Class |
| neurotransmitter:Na+ symporter, NSS family | K03308 | No Class |
| Ammonium transporter, Amt family | K03320 | No Class |
| Glutamyl-tRNA(Gln) amidotransferase subunit E [EC:6.3.5.7] | K03330 | No Class |
| Chromosome partitioning protein | K03496 | No Class |
| Trk system potassium uptake protein TrkH | K03498 | No Class |
| DNA polymerase V | K03502 | No Class |
| quinolinate synthase [EC:2.5.1.72] | K03517 | No Class |
| Exonuclease SbcC | K03546 | No Class |
| DNA mismatch repair protein MutS | K03555 | No Class |
| ATP-dependent DNA helicase RecG [EC:3.6.4.12] | K03655 | No Class |
| DNA helicase II / ATP-dependent DNA helicase PcrA [EC:3.6.4.12] | K03657 | No Class |
| molecular chaperone DnaJ | K03686 | No Class |
| Ubiquinone biosynthesis protein | K03688 | No Class |
| ATP-dependent Clp protease ATP-binding subunit ClpA | K03694 | No Class |
| Heat-inducible transcriptional repressor | K03705 | No Class |
| transcription-repair coupling factor (superfamily II helicase) [EC:3.6.4.-] | K03723 | No Class |
| ATP-dependent helicase Lhr and Lhr-like helicase [EC:3.6.4.-] | K03724 | No Class |
| Thiamine biosynthesis lipoprotein | K03734 | No Class |
| LemA protein | K03744 | No Class |
| arginine:ornithine antiporter | K03758 | No Class |
| DNA polymerase III subunit alpha, Gram-positive type [EC:2.7.7.7] | K03763 | No Class |
| FKBP-type peptidyl-prolyl cis-trans isomerase SlyD [EC:5.2.1.8] | K03775 | No Class |
| D-lactate dehydrogenase [EC:1.1.1.28] | K03778 | No Class |
| 5prime-nucleotidase [EC:3.1.3.5] | K03787 | No Class |
| Cell division protease FtsH [EC:3.4.24.-] | K03798 | No Class |
| ArsR family transcriptional regulator | K03892 | No Class |
| carboxylesterase type B [EC:3.1.1.1] | K03927 | No Class |
| Bifunctional UDP-N-acetylglucosamine pyrophosphorylase / Glucosamine-1-phosphate N-acetyltransferase [EC:2.7.7.23 2.3.1.157] | K04042 | No Class |
| primosomal protein Nprime (replication factor Y) (superfamily II helicase) [EC:3.6.4.-] | K04066 | No Class |
| chaperonin GroES | K04078 | No Class |
| methanol---5-hydroxybenzimidazolylcobamide Co-methyltransferase [EC:2.1.1.90] | K04480 | No Class |
| Nitrogen fixation protein NifU and related proteins | K04488 | No Class |
| Prephenate dehydrogenase [EC:1.3.1.12] | K04517 | No Class |
| Ferrous iron transport protein B | K04759 | No Class |
| beta-glucosidase [EC:3.2.1.21] | K05349 | No Class |
| Beta-glucosidase [EC:3.2.1.21] | K05350 | No Class |
| Tellurite resistance protein TerC | K05794 | No Class |
| Segregation and condensation protein A | K05896 | No Class |
| Precorrin-3B C17-methyltransferase [EC:2.1.1.131] | K05934 | No Class |
| Sialate O-acetylesterase [EC:3.1.1.53] | K05970 | No Class |
| Alpha-L-rhamnosidase [EC:3.2.1.40] | K05989 | No Class |
| ATP-binding cassette, subfamily C, bacterial | K06148 | No Class |
| ATP-binding cassette, sub-family F, member 3 | K06158 | No Class |
| CrcB protein | K06199 | No Class |
| SpoIIIJ-associated protein | K06346 | No Class |
| Putative MFS transporter, AGZA family, xanthine/uracil permease | K06901 | No Class |
| Queuosine biosynthesis protein QueC | K06920 | No Class |
| Pyrimidine/purine-5'-nucleotide nucleosidase [EC:3.2.2.10 3.2.2.-] | K06966 | No Class |
| Uncharacterized protein | K07007 | No Class |
| Putative hydrolases of HD superfamily | K07023 | No Class |
| Sucrose-6-phosphatase  [EC:3.1.3.24] | K07024 | No Class |
| putative hydrolase of the HAD superfamily | K07025 | No Class |
| Uncharacterized protein | K07045 | No Class |
| NA | K07054 | No Class |
| NA | K07079 | No Class |
| N-acetylmuramic acid 6-phosphate etherase [EC:4.2.-.-] | K07106 | No Class |
| acyl-CoA thioester hydrolase [EC:3.1.2.-] | K07107 | No Class |
| uncharacterized protein | K07114 | No Class |
| Uncharacterized protein | K07137 | No Class |
| NA | K07139 | No Class |
| outer membrane protein | K07277 | No Class |
| Inner membrane protein | K07301 | No Class |
| adenine-specific DNA-methyltransferase [EC:2.1.1.72] | K07316 | No Class |
| alpha-galactosidase [EC:3.2.1.22] | K07407 | No Class |
| tRNA (adenine-N1-)-methyltransferase catalytic subunit [EC:2.1.1.36] | K07442 | No Class |
| putative N6-adenine-specific DNA methylase [EC:2.1.1.-] | K07444 | No Class |
| single-stranded-DNA-specific exonuclease [EC:3.1.-.-] | K07462 | No Class |
| Putative transposase | K07491 | No Class |
| Hypothetical protein | K07503 | No Class |
| putative translation factor | K07566 | No Class |
| Metallo-beta-lactamase family protein | K07576 | No Class |
| anaerobic C4-dicarboxylate transporter DcuA | K07791 | No Class |
| MFS transporter, SP family, xylose:H+ symportor | K08138 | No Class |
| MFS transporter, DHA3 family, macrolide efflux protein | K08217 | No Class |
| phosphoribosylglycinamide formyltransferase 2 [EC:2.1.2.2] | K08289 | No Class |
| ribonuclease E [EC:3.1.26.12] | K08300 | No Class |
| putative serine protease PepD [EC:3.4.21.-] | K08372 | No Class |
| Phosphotransferase system, enzyme I, PtsI [EC:2.7.3.9] | K08483 | No Class |
| D-citramalate synthase [EC:2.3.1.182] | K09011 | No Class |
| Hypothetical protein | K09181 | No Class |
| 3-oxoacyl-[acyl-carrier-protein] synthase II [EC:2.3.1.179] | K09458 | No Class |
| Antibiotic transport system ATP-binding protein | K09687 | No Class |
| lipoprotein-releasing system ATP-binding protein [EC:3.6.3.-] | K09810 | No Class |
| cell division transport system ATP-binding protein | K09812 | No Class |
| Zinc transport system substrate-binding protein | K09815 | No Class |
| hypothetical protein | K09955 | No Class |
| LL-diaminopimelate aminotransferase [EC:2.6.1.83] | K10206 | No Class |
| Putative multiple sugar transport system substrate-binding protein | K10546 | No Class |
| DNA ligase 1 [EC:6.5.1.1] | K10747 | No Class |
| endonuclease III [EC:4.2.99.18] | K10773 | No Class |
| spermidine/putrescine transport system substrate-binding protein | K11069 | No Class |
| Cysteine desulfurase / selenocysteine lyase [EC:2.8.1.7 4.4.1.16] | K11717 | No Class |
| LysR family transcriptional regulator | K11921 | No Class |
| Beta-galactosidase [EC:3.2.1.23] | K12308 | No Class |
| Aminobenzoyl-glutamate transport protein | K12942 | No Class |
| uroporphyrinogen III methyltransferase / synthase [EC:2.1.1.107 4.2.1.75] | K13542 | No Class |
| methanol corrinoid protein | K14081 | No Class |
| solute carrier family 13 (sodium-dependent dicarboxylate transporter), member 2/3/5 | K14445 | No Class |
| Bacteria γ-Proteobacteria | *Kangiella* | No Class |
| Bacteria β-Proteobacteria | *Kingella* | No Class |
| Bacteria Firmicutes | *Kurthia* | No Class |
| Bacteria γ-Proteobacteria | *Lacimicrobium* | No Class |
| Bacteria Firmicutes | *Lactobacillus* | No Class |
| Bacteria Bacteroidetes | *Leadbetterella* | No Class |
| Bacteria γ-Proteobacteria | *Lelliottia* | No Class |
| Excavata>Euglenozoa | *Leptomonas* | No Class |
| Bacteria Actinobacteria | *Libanicoccus* | No Class |
| Bacteria γ-Proteobacteria | *Lysobacter* | No Class |
| Bacteria α-Proteobacteria | *Magnetospira* | No Class |
| Bacteria Firmicutes | *Marinilactibacillus* | No Class |
| Bacteria Firmicutes | *Megamonas* | No Class |
| Archaea Euryarchaeota | *Methanobacterium* | No Class |
| Archaea Euryarchaeota | *Methanocorpusculum* | No Class |
| Archaea Euryarchaeota | *Methanofollis* | No Class |
| Archaea Euryarchaeota | *Methanolobus* | No Class |
| Archaea Euryarchaeota | *Methanosphaerula* | No Class |
| Archaea Euryarchaeota | *Methanothermobacter* | No Class |
| Archaea Euryarchaeota | *Methanotorris* | No Class |
| Bacteria Verrucomicrobia | *Methylacidiphilum* | No Class |
| Bacteria γ-Proteobacteria | *Methylococcus* | No Class |
| Bacteria γ-Proteobacteria | *Methylophaga* | No Class |
| Bacteria β-Proteobacteria | *Methylotenera* | No Class |
| Fungi Ascomycota | *Metschnikowia* | No Class |
| Bacteria>Cyanobacteria | *Microcoleus* | No Class |
| Bacteria Cyanobacteria | *Microcystis* | No Class |
| Bacteria Cyanobacteria | *Moorea* | No Class |
| Bacteria γ-Proteobacteria | *Morganella* | No Class |
| Bacteria Bacteroidetes | *Mucilaginibacter* | No Class |
| Animalia Molusca | *Murdochiella* | No Class |
| Bacteria Halobacteria | *Natronolimnobius* | No Class |
| Archaea Halobacteria | *Natronomonas* | No Class |
| Bacteria Firmicutes | *Negativicoccus* | No Class |
| Bacteria Bacteroidetes | *Niabella* | No Class |
| Bacteria Bacteroidetes | *Niastella* | No Class |
| Bacteria Bacteroidetes | *Nonlabens* | No Class |
| Bacteria Firmicutes | *Oceanobacillus* | No Class |
| Bacteria α-Proteobacteria | *Ochrobactrum* | No Class |
| Bacteria Firmicutes | *Oribacterium* | No Class |
| Bacteria Cyanobacteria | *Oscillatoria* | No Class |
| Bacteria Chlorobi | *Pelodictyon* | No Class |
| Bacteria Firmicutes | *Peptoclostridium* | No Class |
| Bacteria Bacteroidetes | *Persicobacter* | No Class |
| Fungi Ascomycota | *Pestalotiopsis* | No Class |
| Fungi Ascomycota | *Phialocephala* | No Class |
| Bacteria Planctomycetes | *Pirellula* | No Class |
| Bacteria Planctomycetes | *Planctopirus* | No Class |
| Bacteria Firmicutes | *Planococcus* | No Class |
| Bacteria β-Proteobacteria | *Polaromonas* | No Class |
| Bacteria Chlorobi | *Prosthecochloris* | No Class |
| Bacteria Firmicutes | *Proteiniclasticum* | No Class |
| Bacteria Bacteroidetes | *Proteiniphilum* | No Class |
| Bacteria Actinobacteria | *Pseudarthrobacter* | No Class |
| Fungi Ascomycota | *Pseudogymnoascus* | No Class |
| Fungi Ascomycota | *Purpureocillium* | No Class |
| Fungi Basidiomycota | *Rhodotorula* | No Class |
| Bacteria Chloroflexi | *Roseiflexus* | No Class |
| Bacteria Planctomycetes | *Rubinisphaera* | No Class |
| Bacteria Actinobacteria | *Saccharomonospora* | No Class |
| Bacteria γ-Proteobacteria | *Saccharophagus* | No Class |
| Bacteria Bacteroidetes | *Salegentibacter* | No Class |
| Bacteria Bacteroidetes | *Sediminicola* | No Class |
| Bacteria Actinobacteria | *Serinicoccus* | No Class |
| Fungi>Ascomycota | *Sordaria* | No Class |
| Bacteria Bacteroidetes | *Sphingobacterium* | No Class |
| Bacteria γ-Proteobacteria | *Spongiibacter* | No Class |
| Bacteria Fusobacteria | *Streptobacillus* | No Class |
| Bacteria Firmicutes | *Syntrophothermus* | No Class |
| Bacteria α-Proteobacteria | *Tateyamaria* | No Class |
| Protist Apusozoa Apusomonadidae | *Thecamonas* | No Class |
| Bacteria Firmicutes | *Thermacetogenium* | No Class |
| Bacteria Actinobacteria | *Thermobifida* | No Class |
| Bacteria Firmicutes | *Thermodesulfobium* | No Class |
| Archea Euryarchaeota | *Thermogymnomonas* | No Class |
| Bacteria γ-Proteobacteria | *Thiomicrospira* | No Class |
| Fungi Basidiomycota | *Tremella* | No Class |

**Table S2A. Enrichment analysis of methanogenesis cluster in Low methane emissions animals (LME) animals compared to High methane emissions animals (HME).**

| **Current Class** | **Class/cluster ID** | **Class Set** | **Observed** | **Expected** | **OverRep (Obs/Exp)** | **Fisher's P** | **Adj. Fisher's P** | **Members** |
| --- | --- | --- | --- | --- | --- | --- | --- | --- |
| Cluster 1 in LME | 3 | Cluster High | 29/675 | 22.77/675 | 1.27 | 1.37E-01 | 3.15E+00 | 88 |
| Cluster 1 in LME | 5 | Cluster High | 1/675 | 1.03/675 | 0.97 | 1.00E+00 | 2.30E+01 | 4 |
| Cluster 1 in LME | 4 | Cluster High | 3/675 | 2.07/675 | 1.45 | 4.34E-01 | 9.98E+00 | 8 |
| Cluster 1 in LME | other | Cluster High | 1/675 | 13.71/675 | 0.07 | 3.72E-06 | **8.56E-05** | 53 |
| Cluster 1 in LME | other | Cluster High | 1/675 | 3.1/675 | 0.32 | 3.18E-01 | 7.31E+00 | 12 |
| Cluster 1 in LME | other | Cluster High | 3/675 | 2.33/675 | 1.29 | 7.03E-01 | 1.62E+01 | 9 |
| Cluster 1 in LME | other | Cluster High | 2/675 | 3.1/675 | 0.64 | 7.42E-01 | 1.71E+01 | 12 |
| Cluster 1 in LME | other | Cluster High | 7/675 | 2.85/675 | 2.46 | 9.11E-03 | 2.10E-01 | 11 |
| Cluster 1 in LME | other | Cluster High | 7/675 | 2.33/675 | 3.01 | 1.64E-03 | **3.78E-02** | 9 |
| Cluster 1 in LME | other | Cluster High | 1/675 | 2.07/675 | 0.48 | 6.89E-01 | 1.58E+01 | 8 |
| Cluster 1 in LME | other | Cluster High | 1/675 | 1.81/675 | 0.55 | 6.85E-01 | 1.58E+01 | 7 |
| Cluster 1 in LME | other | Cluster High | 1/675 | 1.55/675 | 0.64 | 1.00E+00 | 2.30E+01 | 6 |
| Cluster 1 in LME | other | Cluster High | 1/675 | 1.55/675 | 0.64 | 1.00E+00 | 2.30E+01 | 6 |
| Cluster 1 in LME | other | Cluster High | 2/675 | 1.29/675 | 1.55 | 6.09E-01 | 1.40E+01 | 5 |
| Cluster 1 in LME | other | Cluster High | 2/675 | 1.03/675 | 1.93 | 2.76E-01 | 6.36E+00 | 4 |
| Cluster 1 in LME | other | Cluster High | 1/675 | 1.03/675 | 0.97 | 1.00E+00 | 2.30E+01 | 4 |
| Cluster 1 in LME | 6 | Cluster High | 3/675 | 37.51/675 | 0.08 | 1.56E-15 | **3.59E-14** | 145 |
| Cluster 1 in LME | 2 | Cluster High | 10/675 | 83.83/675 | 0.12 | 2.34E-31 | **5.38E-30** | 324 |
| Cluster 1 in LME | other | Cluster High | 1/675 | 54.59/675 | 0.02 | 2.71E-27 | **6.23E-26** | 211 |
| Cluster 1 in LME | 1 | Cluster High | 328/675 | 89.78/675 | 3.65 | 2.01E-196 | **4.63E-195** | 347 |
| Cluster 1 in LME | other | Cluster High | 12/675 | 20.18/675 | 0.59 | 3.50E-02 | 8.04E-01 | 78 |
| Cluster 1 in LME | other | Cluster High | 1/675 | 66.75/675 | 0.01 | 3.29E-34 | **7.57E-33** | 258 |
| Cluster 1 in LME | 9 | Cluster High | 247/675 | 129.88/675 | 1.9 | 1.01E-36 | **2.31E-35** | 502 |
| Cluster 1 in LME | Archaea | Domain | 43/675 | 21.22/675 | 2.03 | 2.29E-07 | **1.37E-06** | 82 |
| Cluster 1 in LME | Bacteria | Domain | 271/675 | 218.88/675 | 1.24 | 8.48E-07 | **5.09E-06** | 846 |
| Cluster 1 in LME | Fungi | Domain | 19/675 | 34.67/675 | 0.55 | 1.11E-03 | **6.67E-03** | 134 |
| Cluster 1 in LME | Genes | Domain | 339/675 | 391.96/675 | 0.86 | 1.91E-06 | **1.15E-05** | 1515 |
| Cluster 1 in LME | Other | Domain | 1/675 | 2.07/675 | 0.48 | 6.89E-01 | 4.13E+00 | 8 |
| Cluster 1 in LME | Protist | Domain | 2/675 | 6.21/675 | 0.32 | 5.88E-02 | 3.53E-01 | 24 |

Bold: values with a corrected *P*-value < 0.05.

**Table S2B. Enrichment analysis of methanogenesis cluster in High methane emissions animals (HME) compared to Low methane emissions animals (LME).**

| **Current Class** | **Class/cluster ID** | **Class Set** | **Observed** | **Expected** | **OverRep (Obs/Exp)** | **Fisher's P** | **Adj. Fisher's P** | **Members** |
| --- | --- | --- | --- | --- | --- | --- | --- | --- |
| Cluster 1 in HME | 3 | Cluster Low | 3/351 | 30.22/351 | 0.1 | 2.80E-11 | **3.08E-10** | 224 |
| Cluster 1 in HME | 7 | Cluster Low | 4/351 | 31.57/351 | 0.13 | 7.38E-11 | **8.12E-10** | 234 |
| Cluster 1 in HME | other | Cluster Low | 1/351 | 11.6/351 | 0.09 | 7.72E-05 | **8.49E-04** | 86 |
| Cluster 1 in HME | other | Cluster Low | 1/351 | 5.53/351 | 0.18 | 3.54E-02 | 3.90E-01 | 41 |
| Cluster 1 in HME | other | Cluster Low | 1/351 | 2.97/351 | 0.34 | 3.47E-01 | 3.82E+00 | 22 |
| Cluster 1 in HME | other | Cluster Low | 1/351 | 2.83/351 | 0.35 | 3.45E-01 | 3.80E+00 | 21 |
| Cluster 1 in HME | other | Cluster Low | 1/351 | 0.67/351 | 1.48 | 5.16E-01 | 5.67E+00 | 5 |
| Cluster 1 in HME | other | Cluster Low | 1/351 | 0.67/351 | 1.48 | 5.16E-01 | 5.67E+00 | 5 |
| Cluster 1 in HME | 2 | Cluster Low | 5/351 | 21.04/351 | 0.24 | 1.62E-05 | **1.78E-04** | 156 |
| Cluster 1 in HME | 1 | Cluster Low | 329/351 | 89.57/351 | 3.67 | 1.58E-196 | **1.73E-195** | 664 |
| Cluster 1 in HME | other | Cluster Low | 3/351 | 7.96/351 | 0.38 | 5.43E-02 | 5.97E-01 | 59 |
| Cluster 1 in HME | Archaea | Domain | 6/351 | 11.2/351 | 0.54 | 1.02E-01 | 4.08E-01 | 83 |
| Cluster 1 in HME | Bacteria | Domain | 14/351 | 114.39/351 | 0.12 | 8.11E-45 | **3.24E-44** | 848 |
| Cluster 1 in HME | Genes | Domain | 328/351 | 203.02/351 | 1.62 | 1.60E-57 | **6.42E-57** | 1505 |
| Cluster 1 in HME | Fungi | Domain | 3/351 | 17.94/351 | 0.17 | 1.05E-05 | **4.18E-05** | 133 |

Bold: values with a corrected *P*-value < 0.05.

# Table S3A. Common microbial communities and genes and classified in the methanogenesis cluster in networks with HME or LME animals.

| Microbial taxon/gene | Genus/KEGG gene id |
| --- | --- |
| Archaea Euryarchaeota | Candidatus *Methanoplasma* |
| Archaea Euryarchaeota | *Methanobrevibacter* |
| Archaea Euryarchaeota | *Methanomicrobium* |
| Archaea Euryarchaeota | *Methanosarcina* |
| Archaea Euryarchaeota | *Methanosphaera* |
| Archaea Euryarchaeota | *Methanothermus* |
| Bacteria Bacteroidetes | Candidatus *Azobacteroides* |
| Archaea Euryarchaeota | Candidatus *Methanoplasma* |
| Bacteria Firmicutes | *Flavonifractor* |
| Bacteria Firmicutes | *Intestinimonas* |
| Bacteria Firmicutes | *Sarcina* |
| Bacteria Plantomycetes | *Planctopirus* |
| Bacteria δ-Proteobacteria | *Desulfobacter* |
| Bacteria α-Proteobacteria | *Marinovum* |
| Bacteria β-Proteobacteria | *Methylobacillus* |
| Bacteria α-Proteobacteria | *PlanKtomarina* |
| Bacteria Thermodesulfobacteria | *Thermodesulfatator* |
| Fungi Basidiomycota | *Coprinopsis* |
| Fungi Basidiomycota | *Tremella* |
| Hydroxymethylglutaryl-CoA reductase (NADPH) [EC:1.1.1.34] | K00021 |
| Ketol-acid reductoisomerase [EC:1.1.1.86] | K00053 |
| Formate dehydrogenase, alpha subunit [EC:1.2.1.2] | K00123 |
| Formate dehydrogenase, beta subunit [EC:1.2.1.2] | K00125 |
| Aspartate-semialdehyde dehydrogenase [EC:1.2.1.11] | K00133 |
| Glyceraldehyde-3-phosphate dehydrogenase (NAD(P)) [EC:1.2.1.59] | K00150 |
| Pyruvate ferredoxin oxidoreductase, alpha subunit [EC:1.2.7.1] | K00169 |
| Pyruvate ferredoxin oxidoreductase, beta subunit [EC:1.2.7.1] | K00170 |
| Pyruvate ferredoxin oxidoreductase, delta subunit [EC:1.2.7.1] | K00171 |
| Pyruvate ferredoxin oxidoreductase, gamma subunit [EC:1.2.7.1] | K00172 |
| 2-oxoisovalerate ferredoxin oxidoreductase, alpha subunit [EC:1.2.7.7] | K00186 |
| 2-oxoisovalerate ferredoxin oxidoreductase, beta subunit [EC:1.2.7.7] | K00187 |
| 2-oxoisovalerate ferredoxin oxidoreductase, delta subunit [EC:1.2.7.7] | K00188 |
| Carbon-monoxide dehydrogenase iron sulfur subunit | K00196 |
| Formylmethanofuran dehydrogenase subunit A [EC:1.2.99.5] | K00200 |
| Formylmethanofuran dehydrogenase subunit B [EC:1.2.99.5] | K00201 |
| Formylmethanofuran dehydrogenase subunit C [EC:1.2.99.5] | K00202 |
| Formylmethanofuran dehydrogenase subunit D [EC:1.2.99.5] | K00203 |
| Formylmethanofuran dehydrogenase subunit H [EC:1.2.99.5] | K00204 |
| Formylmethanofuran dehydrogenase subunit F [EC:1.2.99.5] | K00205 |
| Dihydrodipicolinate reductase [EC:1.3.1.26] | K00215 |
| Fumarate reductase iron-sulfur protein [EC:1.3.99.1] | K00245 |
| Methylenetetrahydromethanopterin dehydrogenase [EC:1.5.99.9] | K00319 |
| Coenzyme F420-dependent N5,N10-methenyltetrahydromethanopterin reductase [EC:1.5.99.11] | K00320 |
| Methyl-coenzyme M reductase alpha subunit [EC:2.8.4.1] | K00399 |
| Methyl coenzyme M reductase system, component A2 | K00400 |
| Methyl-coenzyme M reductase beta subunit [EC:2.8.4.1] | K00401 |
| Methyl-coenzyme M reductase gamma subunit [EC:2.8.4.1] | K00402 |
| Coenzyme F420 hydrogenase alpha subunit [EC:1.12.98.1] | K00440 |
| Coenzyme F420 hydrogenase beta subunit [EC:1.12.98.1] | K00441 |
| Coenzyme F420 hydrogenase delta subunit | K00442 |
| Coenzyme F420 hydrogenase gamma subunit [EC:1.12.98.1] | K00443 |
| Ribonucleoside-triphosphate reductase [EC:1.17.4.2] | K00527 |
| Tetrahydromethanopterin S-methyltransferase subunit A [EC:2.1.1.86] | K00577 |
| Tetrahydromethanopterin S-methyltransferase subunit B [EC:2.1.1.86] | K00578 |
| Tetrahydromethanopterin S-methyltransferase subunit C [EC:2.1.1.86] | K00579 |
| Tetrahydromethanopterin S-methyltransferase subunit D [EC:2.1.1.86] | K00580 |
| Tetrahydromethanopterin S-methyltransferase subunit E [EC:2.1.1.86] | K00581 |
| Tetrahydromethanopterin S-methyltransferase subunit G [EC:2.1.1.86] | K00583 |
| Tetrahydromethanopterin S-methyltransferase subunit H [EC:2.1.1.86] | K00584 |
| Formylmethanofuran--tetrahydromethanopterin N-formyltransferase [EC:2.3.1.101] | K00672 |
| Orotate phosphoribosyltransferase [EC:2.4.2.10] | K00762 |
| Riboflavin synthase beta chain [EC:2.5.1.-] | K00794 |
| Deoxyhypusine synthase [EC:2.5.1.46] | K00809 |
| Nucleoside-diphosphate kinase [EC:2.7.4.6] | K00940 |
| UTP--glucose-1-phosphate uridylyltransferase [EC:2.7.7.9] | K00963 |
| Glycerol-3-phosphate cytidylyltransferase [EC:2.7.7.39] | K00980 |
| Pyruvate, water dikinase [EC:2.7.9.2] | K01007 |
| Phosphoserine phosphatase [EC:3.1.3.3] | K01079 |
| Adenosylhomocysteinase [EC:3.3.1.1] | K01251 |
| Phosphoribosyl-AMP cyclohydrolase [EC:3.5.4.19] | K01496 |
| Methenyltetrahydromethanopterin cyclohydrolase [EC:3.5.4.27] | K01499 |
| Nucleoside-triphosphatase [EC:3.6.1.15] | K01516 |
| Arsenite-transporting ATPase [EC:3.6.3.16] | K01551 |
| Fructose 1,6-bisphosphate aldolase/phosphatase [EC:4.1.2.13 3.1.3.11] | K01622 |
| Fructose-bisphosphate aldolase, class I [EC:4.1.2.13] | K01623 |
| N-acetylneuraminate synthase [EC:2.5.1.56] | K01654 |
| Carbonic anhydrase [EC:4.2.1.1] | K01673 |
| Fumarate hydratase subunit alpha [EC:4.2.1.2] | K01677 |
| Threonine synthase [EC:4.2.3.1] | K01733 |
| 6-pyruvoyl tetrahydrobiopterin synthase [EC:4.2.3.12] | K01737 |
| Diaminopimelate epimerase [EC:5.1.1.7] | K01778 |
| Myo-inositol-1-phosphate synthase [EC:5.5.1.4] | K01858 |
| Aspartyl-tRNA synthetase [EC:6.1.1.12] | K01876 |
| Succinyl-CoA synthetase alpha subunit [EC:6.2.1.5] | K01902 |
| Succinyl-CoA synthetase beta subunit [EC:6.2.1.5] | K01903 |
| NAD+ synthase [EC:6.3.1.5] | K01916 |
| Phosphoribosylamine--glycine ligase [EC:6.3.4.13] | K01945 |
| Pyruvate carboxylase subunit A [EC:6.4.1.1] | K01959 |
| SRP RNA | K01983 |
| Cobalt/nickel transport system permease protein | K02007 |
| Cobalt transport protein | K02009 |
| Molybdate transport system permease protein | K02018 |
| Molybdate transport system regulatory protein | K02019 |
| Molybdate transport system substrate-binding protein | K02020 |
| Peptide/nickel transport system ATP-binding protein | K02031 |
| V-type H+-transporting ATPase subunit A [EC:3.6.3.14] | K02117 |
| V-type H+-transporting ATPase subunit B [EC:3.6.3.14] | K02118 |
| V-type H+-transporting ATPase subunit C [EC:3.6.3.14] | K02119 |
| V-type H+-transporting ATPase subunit D [EC:3.6.3.14] | K02120 |
| V-type H+-transporting ATPase subunit E [EC:3.6.3.14] | K02121 |
| V-type H+-transporting ATPase subunit F [EC:3.6.3.14] | K02122 |
| V-type H+-transporting ATPase subunit I [EC:3.6.3.14] | K02123 |
| V-type H+-transporting ATPase subunit K [EC:3.6.3.14] | K02124 |
| Ferritin [EC:1.16.3.1] | K02217 |
| Adenosylcobyric acid synthase [EC:6.3.5.10] | K02232 |
| Uroporphyrin-III C-methyltransferase [EC:2.1.1.107] | K02303 |
| DNA polymerase I [EC:2.7.7.7] | K02319 |
| DNA polymerase II large subunit [EC:2.7.7.7] | K02322 |
| DNA polymerase II small subunit [EC:2.7.7.7] | K02323 |
| FdhD protein | K02379 |
| Ribosomal RNA large subunit methyltransferase E [EC:2.1.1.-] | K02427 |
| UDP-N-acetyl-D-mannosaminuronic acid dehydrogenase [EC:1.1.1.-] | K02472 |
| Nitrogen fixation protein NifB | K02585 |
| 3,4-dihydroxy 2-butanone 4-phosphate synthase [EC:4.1.99.12] | K02858 |
| Large subunit ribosomal protein L10e | K02866 |
| Large subunit ribosomal protein L12 | K02869 |
| Large subunit ribosomal protein L14e | K02875 |
| Large subunit ribosomal protein L15e | K02877 |
| Large subunit ribosomal protein L18e | K02883 |
| Large subunit ribosomal protein L19e | K02885 |
| Large subunit ribosomal protein L21e | K02889 |
| Large subunit ribosomal protein L24e | K02896 |
| Large subunit ribosomal protein L29 | K02904 |
| Large subunit ribosomal protein L30e | K02908 |
| Large subunit ribosomal protein L31e | K02910 |
| Large subunit ribosomal protein L32e | K02912 |
| Large subunit ribosomal protein L34e | K02915 |
| Large subunit ribosomal protein L37Ae | K02921 |
| Large subunit ribosomal protein L37e | K02922 |
| Large subunit ribosomal protein L39e | K02924 |
| Large subunit ribosomal protein L40e | K02927 |
| Large subunit ribosomal protein L44e | K02929 |
| Large subunit ribosomal protein L4e | K02930 |
| Large subunit ribosomal protein L7Ae | K02936 |
| Large subunit ribosomal protein LX | K02944 |
| Small subunit ribosomal protein S17e | K02962 |
| Small subunit ribosomal protein S19e | K02966 |
| Small subunit ribosomal protein S24e | K02974 |
| Small subunit ribosomal protein S27Ae | K02977 |
| Small subunit ribosomal protein S27e | K02978 |
| Small subunit ribosomal protein S28e | K02979 |
| Small subunit ribosomal protein S3Ae | K02984 |
| Small subunit ribosomal protein S4e | K02987 |
| Small subunit ribosomal protein S6e | K02991 |
| Small subunit ribosomal protein S8e | K02995 |
| Small subunit ribosomal protein S9 | K02996 |
| DNA-directed RNA polymerase subunit Aprime [EC:2.7.7.6] | K03041 |
| DNA-directed RNA polymerase subunit Aprime [EC:2.7.7.6] | K03042 |
| DNA-directed RNA polymerase subunit Bprime [EC:2.7.7.6] | K03044 |
| DNA-directed RNA polymerase subunit Bprime [EC:2.7.7.6] | K03045 |
| DNA-directed RNA polymerase subunit D [EC:2.7.7.6] | K03047 |
| DNA-directed RNA polymerase subunit Eprime [EC:2.7.7.6] | K03049 |
| DNA-directed RNA polymerase subunit K [EC:2.7.7.6] | K03055 |
| DNA-directed RNA polymerase subunit L [EC:2.7.7.6] | K03056 |
| DNA-directed RNA polymerase subunit N [EC:2.7.7.6] | K03058 |
| DNA-directed RNA polymerase subunit P [EC:2.7.7.6] | K03059 |
| Preprotein translocase subunit SecD | K03072 |
| Preprotein translocase subunit SecF | K03074 |
| Translation initiation factor eIF-1 | K03113 |
| Transcription initiation factor TFIID TATA-box-binding protein | K03120 |
| Transcription initiation factor TFIIB | K03124 |
| Transcription initiation factor TFIIE alpha subunit | K03136 |
| Thiamine biosynthesis protein ThiC | K03147 |
| DNA topoisomerase VI subunit A [EC:5.99.1.3] | K03166 |
| DNA topoisomerase VI subunit B [EC:5.99.1.3] | K03167 |
| Elongation factor EF-1 alpha subunit [EC:3.6.5.3] | K03231 |
| Elongation factor EF-1 beta subunit | K03232 |
| Elongation factor EF-2 [EC:3.6.5.3] | K03234 |
| Translation initiation factor eIF-1A | K03236 |
| Translation initiation factor eIF-2 alpha subunit | K03237 |
| Translation initiation factor eIF-2 beta subunit | K03238 |
| Translation initiation factor eIF-2 gamma subunit | K03242 |
| Translation initiation factor eIF-5B | K03243 |
| Translation initiation factor eIF-5A | K03263 |
| Translation initiation factor eIF-6 | K03264 |
| Peptide chain release factor eRF subunit 1 | K03265 |
| Glutamyl-tRNA(Gln) amidotransferase subunit E [EC:6.3.5.7] | K03330 |
| Heterodisulfide reductase subunit A [EC:1.8.98.1] | K03388 |
| Heterodisulfide reductase subunit B [EC:1.8.98.1] | K03389 |
| Heterodisulfide reductase subunit C [EC:1.8.98.1] | K03390 |
| Proteasome regulatory subunit | K03420 |
| Methyl-coenzyme M reductase subunit C | K03421 |
| Methyl-coenzyme M reductase subunit D | K03422 |
| Proteasome alpha subunit [EC:3.4.25.1] | K03432 |
| Proteasome beta subunit [EC:3.4.25.1] | K03433 |
| Ribonuclease P protein subunit POP4 [EC:3.1.26.5] | K03538 |
| Ribonuclease P protein subunit RPR2 [EC:3.1.26.5] | K03540 |
| Holliday junction resolvase, archaea type | K03552 |
| Molybdopterin synthase catalytic subunit [EC:2.-.-.-] | K03635 |
| Molybdenum cofactor biosynthesis protein | K03639 |
| Exosome complex component RRP4 | K03679 |
| DtxR family transcriptional regulator, Mn-dependent transcriptional regulator | K03709 |
| Archaea-specific helicase [EC:3.6.1.-] | K03725 |
| Helicase [EC:3.6.4.-] | K03726 |
| Aldehyde:ferredoxin oxidoreductase [EC:1.2.7.5] | K03738 |
| Molybdopterin biosynthesis protein MoeA | K03750 |
| Molybdopterin-guanine dinucleotide biosynthesis protein A | K03752 |
| Molybdopterin-guanine dinucleotide biosynthesis protein B | K03753 |
| Peptidyl-prolyl cis-trans isomerase B (cyclophilin B) [EC:5.2.1.8] | K03768 |
| FKBP-type peptidyl-prolyl cis-trans isomerase SlyD [EC:5.2.1.8] | K03775 |
| Sirohydrochlorin cobaltochelatase [EC:4.99.1.3] | K03795 |
| NADH dehydrogenase [EC:1.6.99.3] | K03885 |
| Lon-like ATP-dependent protease [EC:3.4.21.-] | K04076 |
| Chorismate mutase [EC:5.4.99.5] | K04093 |
| DNA repair protein RadA | K04483 |
| Lysyl-tRNA synthetase, class I [EC:6.1.1.6] | K04566 |
| Hydrogenase nickel incorporation protein HypB | K04652 |
| Hydrogenase expression/formation protein HypC | K04653 |
| Hydrogenase expression/formation protein HypD | K04654 |
| Hydrogenase expression/formation protein HypE | K04655 |
| Hydrogenase maturation protein HypF | K04656 |
| Peptidyl-tRNA hydrolase, PTH2 family [EC:3.1.1.29] | K04794 |
| Fibrillarin-like pre-rRNA processing protein | K04795 |
| Small nuclear ribonucleoprotein | K04796 |
| Prefoldin alpha subunit | K04797 |
| Prefoldin beta subunit | K04798 |
| Replication factor C small subunit | K04801 |
| Proliferating cell nuclear antigen | K04802 |
| (R)-2-hydroxyacid dehydrogenase [EC:1.1.1.272] | K05884 |
| Precorrin-6X reductase [EC:1.3.1.54] | K05895 |
| Precorrin-4 C11-methyltransferase [EC:2.1.1.133] | K05936 |
| Precorrin-8X methylmutase [EC:5.4.1.2] | K06042 |
| ATP-binding cassette, sub-family E, member 1 | K06174 |
| tRNA pseudouridine synthase D [EC:5.4.99.12] | K06176 |
| Hydrogenase large subunit [EC:1.12.99.6] | K06281 |
| 5-formaminoimidazole-4-carboxamide-1-(beta)-D-ribofuranosyl 5prime-monophosphate synthetase [EC:6.3.4.-] | K06863 |
| ATPase | K06865 |
| Programmed cell death protein 5 | K06875 |
| Nicotinic acid adenine dinucleotide carboxylase/hydrolase | K06898 |
| Queuosine biosynthesis protein QueC | K06920 |
| tRNA(Ile2)-agmatinylcytidine synthase [EC:6.3.4.22] | K06932 |
| 7,8-dihydro-6-hydroxymethylpterin dimethyltransferase [EC:2.1.1.-] | K06937 |
| Nucleolar GTP-binding protein | K06943 |
| Uncharacterized protein | K06944 |
| Ribosomal RNA assembly protein | K06961 |
| Beta-ribofuranosylaminobenzene 5'-phosphate synthase [EC:2.4.2.54] | K06984 |
| Uncharacterized protein | K06988 |
| Uncharacterized protein | K07013 |
| No such data | K07021 |
| Uncharacterized protein | K07041 |
| Uncharacterized protein | K07045 |
| Uncharacterized protein | K07068 |
| (4-(4-[2-(gamma-L-glutamylamino)ethyl]phenoxymethyl)furan-2-yl)methanamine synthase [EC:2.5.1.131] | K07072 |
| Putative glycerol-1-phosphate prenyltransferase [EC:2.5.1.-] | K07094 |
| Uncharacterized protein | K07096 |
| Uncharacterized protein | K07108 |
| Uncharacterized protein | K07135 |
| 5-(aminomethyl)-3-furanmethanol phosphate kinase [EC:2.7.4.31] | K07144 |
| Dolichyl-diphosphooligosaccharide--protein glycosyltransferase [EC:2.4.1.119] | K07151 |
| Uncharacterized protein | K07159 |
| Uncharacterized protein | K07161 |
| Hypothetical protein | K07254 |
| Anaerobic dimethyl sulfoxide reductase subunit A [EC:1.8.99.-] | K07306 |
| Hydrogenase expression/formation protein | K07388 |
| AAA family ATPase | K07392 |
| Putative methyltransferase | K07446 |
| Archaea-specific RecJ-like exonuclease | K07463 |
| Diphthamide synthase subunit DPH2 | K07561 |
| RNA-binding protein | K07569 |
| Putative nucleotide binding protein | K07572 |
| Exosome complex component CSL4 | K07573 |
| Putative RNA-binding protein containing KH domain | K07574 |
| Hypothetical protein | K07580 |
| Hypothetical protein | K07582 |
| Hypothetical protein | K07585 |
| ArsR family transcriptional regulator | K07721 |
| CopG family transcriptional regulator, nickel-responsive regulator | K07722 |
| Putative transcriptional regulator | K07728 |
| Putative transcriptional regulator | K07730 |
| Elongator complex protein 3 [EC:2.3.1.48] | K07739 |
| Hypothetical protein | K07744 |
| 6-phospho-3-hexuloisomerase [EC:5.3.1.27] | K08094 |
| GTP cyclohydrolase IIa [EC:3.5.4.29] | K08096 |
| Methylthioribose-1-phosphate isomerase [EC:5.3.1.23] | K08963 |
| Hypothetical protein | K09003 |
| Hypothetical protein | K09007 |
| Hypothetical protein | K09116 |
| Hypothetical protein | K09123 |
| Hypothetical protein | K09128 |
| Hypothetical protein | K09136 |
| Hypothetical protein | K09140 |
| Hypothetical protein | K09142 |
| Hypothetical protein | K09154 |
| Hypothetical protein | K09181 |
| Glutamyl-tRNA(Gln) amidotransferase subunit D [EC:6.3.5.7] | K09482 |
| Hypothetical protein | K09717 |
| Hypothetical protein | K09720 |
| Hypothetical protein | K09726 |
| Hypothetical protein | K09728 |
| Hypothetical protein | K09733 |
| Hypothetical protein | K09735 |
| LL-diaminopimelate aminotransferase [EC:2.6.1.83] | K10206 |
| Archaeal cell division control protein 6 | K10725 |
| Replicative DNA helicase Mcm [EC:3.6.4.-] | K10726 |
| Fanconi anemia group M protein [EC:3.6.4.13] | K10896 |
| Trans-homoaconitate synthase [EC:4.1.3.-] | K10977 |
| IMP cyclohydrolase [EC:3.5.4.10] | K11176 |
| Formylmethanofuran dehydrogenase subunit G [EC:1.2.99.5] | K11260 |
| Formylmethanofuran dehydrogenase subunit E [EC:1.2.99.5] | K11261 |
| Exosome complex component RRP41 | K11600 |
| Dehydroquinate synthase II [EC:1.4.1.-] | K11646 |
| FO synthase subunit 1 [EC:2.5.1.-] | K11780 |
| FO synthase subunit 2 [EC:2.5.1.-] | K11781 |
| Exosome complex component RRP42 | K12589 |
| Transitional endoplasmic reticulum ATPase | K13525 |
| Geranylgeranyl diphosphate synthase, type I [EC:2.5.1.1 2.5.1.10 2.5.1.29] | K13787 |
| Bifunctional enzyme Fae/Hps [EC:4.3.-.- 4.1.2.43] | K13812 |
| 5,10-methenyltetrahydromethanopterin hydrogenase [EC:1.12.98.2] | K13942 |
| Alcohol dehydrogenase, propanol-preferring [EC:1.1.1.1] | K13953 |
| Energy-converting hydrogenase A subunit C | K14094 |
| Energy-converting hydrogenase A subunit E | K14096 |
| Energy-converting hydrogenase A subunit G | K14098 |
| Energy-converting hydrogenase A subunit H | K14099 |
| Energy-converting hydrogenase A subunit J | K14101 |
| Energy-converting hydrogenase A subunit M | K14104 |
| Energy-converting hydrogenase A subunit N | K14105 |
| Energy-converting hydrogenase A subunit O | K14106 |
| Energy-converting hydrogenase A subunit P | K14107 |
| Energy-converting hydrogenase A subunit Q | K14108 |
| Energy-converting hydrogenase A subunit R | K14109 |
| Energy-converting hydrogenase B subunit A | K14110 |
| Energy-converting hydrogenase B subunit F | K14115 |
| Energy-converting hydrogenase B subunit K | K14120 |
| Energy-converting hydrogenase B subunit L | K14121 |
| Energy-converting hydrogenase B subunit M | K14122 |
| Energy-converting hydrogenase B subunit N | K14123 |
| Energy-converting hydrogenase B subunit O | K14124 |
| F420-non-reducing hydrogenase subunit A [EC:1.12.99.-] | K14126 |
| F420-non-reducing hydrogenase iron-sulfur subunit D [EC:1.12.99.-] | K14127 |
| F420-non-reducing hydrogenase subunit G [EC:1.12.99.-] | K14128 |

# Table S3B. Microbial communities and genes classified in the methanogenesis cluster exclusively in HME animals.

| Microbial taxon/gene | Genus/KEGG gene id |
| --- | --- |
| Bacteria Bacteroidetes | *Pseudopedobacter* |
| Bacteria Bacteroidetes | *Seonamhaeicola* |
| Bacteria Firmicutes | *Kyrpidia* |
| Bacteria Firmicutes | *Lachnobacterium* |
| Fungi Ascomycota | *Metschnikowia* |
| Branched-chain amino acid aminotransferase [EC:2.6.1.42] | K00826 |
| Ribokinase [EC:2.7.1.15] | K00852 |
| Phosphoribosyl-ATP pyrophosphohydrolase [EC:3.6.1.31] | K01523 |
| Diaminopimelate decarboxylase [EC:4.1.1.20] | K01586 |
| Hydroxymethylbilane synthase [EC:2.5.1.61] | K01749 |
| UDP-N-acetylglucosamine 2-epimerase [EC:5.1.3.14] | K01791 |
| L-rhamnose isomerase [EC:5.3.1.14] | K01813 |
| Cobalt/nickel transport system permease protein | K02008 |
| Polar amino acid transport system substrate-binding protein | K02030 |
| Cobalamin biosynthesis protein CbiG | K02189 |
| Glutamyl-tRNA reductase [EC:1.2.1.70] | K02492 |
| Uracil permease | K02824 |
| Large subunit ribosomal protein L30 | K02907 |
| tRNA pseudouridine synthase B [EC:5.4.99.12] | K03177 |
| Nitrogen fixation protein NifU and related proteins | K04488 |
| Precorrin-3B C17-methyltransferase [EC:2.1.1.131] | K05934 |
| Endonuclease III [EC:4.2.99.18] | K10773 |

# Table S3C. Microbial communities and genes classified in the methanogenesis cluster exclusively in LME animals.

| Microbial taxon/gene | Genus/KEGG gene id |
| --- | --- |
| Archaea Crenarchaeota | *Pyrodictium* |
| Archaea Crenarchaeota | *Thermoproteus* |
| Archaea Euryarchaeota | *Methanosalsum* |
| Archaea Euryarchaeota | *Archaeoglobus* |
| Archaea Euryarchaeota | Candidatus *Halobonum* |
| Archaea Euryarchaeota | *Haladaptatus* |
| Archaea Euryarchaeota | *Halalkalicoccus* |
| Archaea Euryarchaeota | *Haloarcula* |
| Archaea Euryarchaeota | *Halobellus* |
| Archaea Euryarchaeota | *Halobiforma* |
| Archaea Euryarchaeota | *Halogeometricum* |
| Archaea Euryarchaeota | *Halopenitus* |
| Archaea Euryarchaeota | *Halopiger* |
| Archaea Euryarchaeota | *Halorubrum* |
| Archaea Euryarchaeota | *Halostagnicola* |
| Archaea Euryarchaeota | *Haloterrigena* |
| Archaea Euryarchaeota | *Halovivax* |
| Archaea Euryarchaeota | *Methanobacterium* |
| Archaea Euryarchaeota | *Methanocella* |
| Archaea Euryarchaeota | *Methanoculleus* |
| Archaea Euryarchaeota | *Methanogenium* |
| Archaea Euryarcheaota | *Methanohalobium* |
| Archaea Euryarchaeota | *Methanolacinia* |
| Archaea Euryarchaeota | *Methanolinea* |
| Archaea Euryarcheaota | *Methanomethylovorans* |
| Archaea Euryarchaeota | *Methanosaeta* |
| Archaea Euryarchaeota | *Methanosphaerula* |
| Archaea Euryarchaeota | *Methanospirillum* |
| Archaea Euryarchaeota | *Methanotorris* |
| Archaea Euryarchaeota | *Methermicoccus* |
| Archaea Euryarchaeota | *Natronobacterium* |
| Archaea Euryarchaeota | *Salinarchaeum* |
| Archaea Euryarchaeota | *Thermogymnomonas* |
| Archaea Halobacteria | *Natrialba* |
| Archaea Halobacteria | *Natronomonas* |
| Archaea Thaumarchaeaota | Candidatus *Nitrosoarchaeum* |
| Archaea Thaumarchaeota | *Nitrosopumilus* |
| Bacteria Candidatus Cloacimonetes | Candidatus *Cloacimonas* |
| Bacteria Acidobacteria | *Acidobacterium* |
| Bacteria Acidobacteria | Candidatus *Solibacter* |
| Bacteria Acidobacteria | *Chloracidobacterium* |
| Bacteria Actinobacteria | *Acidipropionibacterium* |
| Bacteria Actinobacteria | *Actinoalloteichus* |
| Bacteria Actinobacteria | *Actinomyces* |
| Bacteria Actinobacteria | *Actinoplanes* |
| Bacteria Actinobacteria | *Actinosynnema* |
| Bacteria Actinobacteria | *Alloactinosynnema* |
| Bacteria Actinobacteria | *Amycolatopsis* |
| Bacteria Actinobacteria | *Arsenicicoccus* |
| Bacteria Actinobacteria | *Brachybacterium* |
| Bacteria Actinobacteria | *Cryobacterium* |
| Bacteria Actinobacteria | *Cryptobacterium* |
| Bacteria Actinobacteria | *Dermacoccus* |
| Bacteria Actinobacteria | *Devriesea* |
| Bacteria Actinobacteria | *Eggerthella* |
| Bacteria Actinobacteria | *Frankia* |
| Bacteria Actinobacteria | *Geodermatophilus* |
| Bacteria Actinobacteria | *Gordonibacter* |
| Bacteria Actinobacteria | *Ilumatobacter* |
| Bacteria Actinobacteria | *Jonesia* |
| Bacteria Actinobacteria | *Kibdelosporangium* |
| Bacteria Actinobacteria | *Kineococcus* |
| Bacteria Actinobacteria | *Kitasatospora* |
| Bacteria Actinobacteria | *Kocuria* |
| Bacteria Actinobacteria | *Kribbella* |
| Bacteria Actinobacteria | *Lentzea* |
| Bacteria Actinobacteria | *Micrococcus* |
| Bacteria Actinobacteria | *Microlunatus* |
| Bacteria Actinobacteria | *Micromonospora* |
| Bacteria Actinobacteria | *Mobiluncus* |
| Bacteria Actinobacteria | *Nakamurella* |
| Bacteria Actinobacteria | *Neomicrococcus* |
| Bacteria Actinobacteria | *Nocardia* |
| Bacteria Actinobacteria | *Nocardioides* |
| Bacteria Actinobacteria | *Nocardiopsis* |
| Bacteria Actinobacteria | *Pseudarthrobacter* |
| Bacteria Actinobacteria | *Pseudopropionibacterium* |
| Bacteria Actinobacteria | *Rhodoluna* |
| Bacteria Actinobacteria | *Rothia* |
| Bacteria Actinobacteria | *Saccharothrix* |
| Bacteria Actinobacteria | *Salinispora* |
| Bacteria Actinobacteria | *Segniliparus* |
| Bacteria Actinobacteria | *Slackia* |
| Bacteria Actinobacteria | *Streptomyces* |
| Bacteria Actinobacteria | *Streptosporangium* |
| Bacteria Actinobacteria | *Thermobispora* |
| Bacteria Actinobacteria | *Thermomonospora* |
| Bacteria Actinobacteria | *Tropheryma* |
| Bacteria Actinobacteria | *Verrucosispora* |
| Bacteria Aquificae | *Hydrogenobacter* |
| Bacteria Aquificae | *Hydrogenobaculum* |
| Bacteria Aquificae | *Persephonella* |
| Bacteria Aquificae | *Thermovibrio* |
| Bacteria Armatimonadetes | *Fimbriimonas* |
| Bacteria Bacteriodetes | *Lutibacter* |
| Bacteria Bacteroidetes | *Aequorivita* |
| Bacteria Bacteroidetes | *Algoriphagus* |
| Bacteria Bacteroidetes | *Croceibacter* |
| Bacteria Bacteroidetes | *Draconibacterium* |
| Bacteria Bacteroidetes | *Emticicia* |
| Bacteria Bacteroidetes | *Fibrella* |
| Bacteria Bacteroidetes | *Formosa* |
| Bacteria Bacteroidetes | *Hymenobacter* |
| Bacteria Bacteroidetes | *Lacinutrix* |
| Bacteria Bacteroidetes | *Muricauda* |
| Bacteria Bacteroidetes | *Niabella* |
| Bacteria Bacteroidetes | *Niastella* |
| Bacteria Bacteroidetes | *Ornithobacterium* |
| Bacteria Bacteroidetes | *Owenweeksia* |
| Bacteria Bacteroidetes | *Polaribacter* |
| Bacteria Bacteroidetes | *Psychroflexus* |
| Bacteria Bacteroidetes | *Rhodothermus* |
| Bacteria Bacteroidetes | *Runella* |
| Bacteria Bacteroidetes | *Salinibacter* |
| Bacteria Bacteroidetes | *Saprospira* |
| Bacteria Bacteroidetes | *Wenyingzhuangia* |
| Bacteria Bacteroidetes | *Winogradskyella* |
| Bacteria Bacteroidetes | *Zunongwangia* |
| Bacteria Chlamydiae | Candidatus *Protochlamydia* |
| Bacteria Chlorobi | *Pelodictyon* |
| Bacteria Chlorobi | *Prosthecochloris* |
| Bacteria Chloroflexi | *Sphaerobacter* |
| Bacteria Cloroflexi | *Anaerolinea* |
| Bacteria Cloroflexi | *Roseiflexus* |
| Bacteria Cyanobacteria | Candidatus *Atelocyanobacterium* |
| Bacteria Cyanobacteria | *Chamaesiphon* |
| Bacteria Cyanobacteria | *Chroococcidiopsis* |
| Bacteria Cyanobacteria | *Cyanothece* |
| Bacteria Cyanobacteria | *Dactylococcopsis* |
| Bacteria Cyanobacteria | *Fischerella* |
| Bacteria Cyanobacteria | *Geitlerinema* |
| Bacteria Cyanobacteria | *Halothece* |
| Bacteria Cyanobacteria | *Microcystis* |
| Bacteria Cyanobacteria | *Moorea* |
| Bacteria Cyanobacteria | *Planktothrix* |
| Bacteria Cyanobacteria | *Pleurocapsa* |
| Bacteria Cyanobacteria | *Prochlorococcus* |
| Bacteria Cyanobacteria | *Stanieria* |
| Bacteria Cyanobacteria | *Synechocystis* |
| Bacteria Deferribacteres | *Denitrovibrio* |
| Bacteria Deinococcus-Thermus | *Deinococcus* |
| Bacteria Deinococcus-Thermus | *Marinithermus* |
| Bacteria Deinococcus-Thermus | *Meiothermus* |
| Bacteria Deinococcus-Thermus | *Oceanithermus* |
| Bacteria Deinococcus-Thermus | *Thermus* |
| Bacteria Desulfitobacterium | *Desulfarculus* |
| Bacteria Elusimicrobia | *Endomicrobium* |
| Bacteria Firmicutes | *Acetohalobium* |
| Bacteria Firmicutes | *Aerococcus* |
| Bacteria Firmicutes | *Alkaliphilus* |
| Bacteria Firmicutes | *Ammonifex* |
| Bacteria Firmicutes | *Anoxybacillus* |
| Bacteria Firmicutes | *Brevibacillus* |
| Bacteria Firmicutes | *Butyrivibrio* |
| Bacteria Firmicutes | Candidatus *Desulforudis* |
| Bacteria Firmicutes | *Carboxydothermus* |
| Bacteria Firmicutes | *Coprothermobacter* |
| Bacteria Firmicutes | *Dehalobacter* |
| Bacteria Firmicutes | *Desulfotomaculum* |
| Bacteria Firmicutes | *Erysipelothrix* |
| Bacteria Firmicutes | *Ethanoligenens* |
| Bacteria Firmicutes | *Ezakiella* |
| Bacteria Firmicutes | *Filifactor* |
| Bacteria Firmicutes | *Gemella* |
| Bacteria Firmicutes | *Geosporobacter* |
| Bacteria Firmicutes | *Halobacillus* |
| Bacteria Firmicutes | *Lentibacillus* |
| Bacteria Firmicutes | *Limnochorda* |
| Bacteria Firmicutes | *Listeria* |
| Bacteria Firmicutes | *Lysinibacillus* |
| Bacteria Firmicutes | *Mageeibacillus* |
| Bacteria Firmicutes | *Mahella* |
| Bacteria Firmicutes | *Marinilactibacillus* |
| Bacteria Firmicutes | *Natranaerobius* |
| Bacteria Firmicutes | *Parageobacillus* |
| Bacteria Firmicutes | *Parvimonas* |
| Bacteria Firmicutes | *Peptoclostridium* |
| Bacteria Firmicutes | *Planococcus* |
| Bacteria Firmicutes | *Pseudobutyrivibrio* |
| Bacteria Firmicutes | *Ruminiclostridium* |
| Bacteria Firmicutes | *Solibacillus* |
| Bacteria Firmicutes | *Sporosarcina* |
| Bacteria Firmicutes | *Symbiobacterium* |
| Bacteria Firmicutes | *Syntrophomonas* |
| Bacteria Firmicutes | *Syntrophothermus* |
| Bacteria Firmicutes | *Tetragenococcus* |
| Bacteria Firmicutes | *Thermacetogenium* |
| Bacteria Firmicutes | *Thermaerobacter* |
| Bacteria Firmicutes | *Thermincola* |
| Bacteria Firmicutes | *Thermobacillus* |
| Bacteria Firmicutes | *Thermosediminibacter* |
| Bacteria Firmicutes | *Turicibacter* |
| Bacteria Firmicutes | *Vagococcus* |
| Bacteria Gemmatimonadetes | *Gemmatimonas* |
| Bacteria Ignavibacteriae | *Ignavibacterium* |
| Bacteria Ignavibacteriae | *Melioribacter* |
| Bacteria Kiritimatiellaeota | *Kiritimatiella* |
| Bacteria Planctomycetes | *Isosphaera* |
| Bacteria Planctomycetes | *Phycisphaera* |
| Bacteria Planctomycetes | *Rhodopirellula* |
| Bacteria Planctomycetes | *Rubinisphaera* |
| Bacteria Planctomycetes | *Singulisphaera* |
| Bacteria Plantomycetes | *Paludisphaera* |
| Bacteria Plantomycetes | *Pirellula* |
| Bacteria Plantomycetes | *Planctomyces* |
| Bacteria α-Proteobacteria | *Asaia* |
| Bacteria α-Proteobacteria | *Aureimonas* |
| Bacteria α-Proteobacteria | *Bosea* |
| Bacteria α-Proteobacteria | *Brucella* |
| Bacteria α-Proteobacteria | *Dinoroseobacter* |
| Bacteria α-Proteobacteria | *Donghicola* |
| Bacteria α-Proteobacteria | *Erythrobacter* |
| Bacteria α-Proteobacteria | *Filomicrobium* |
| Bacteria α-Proteobacteria | *Gluconacetobacter* |
| Bacteria α-Proteobacteria | *Gluconobacter* |
| Bacteria α-Proteobacteria | *Granulibacter* |
| Bacteria α-Proteobacteria | *Hoeflea* |
| Bacteria α-Proteobacteria | *Hyphomicrobium* |
| Bacteria α-Proteobacteria | *Jannaschia* |
| Bacteria α-Proteobacteria | *Komagataeibacter* |
| Bacteria α-Proteobacteria | *Magnetococcus* |
| Bacteria α-Proteobacteria | *Magnetospira* |
| Bacteria α-Proteobacteria | *Magnetospirillum* |
| Bacteria α-Proteobacteria | *Maricaulis* |
| Bacteria α-Proteobacteria | *Methylocystis* |
| Bacteria α-Proteobacteria | *Micavibrio* |
| Bacteria α-Proteobacteria | *Novosphingobium* |
| Bacteria α-Proteobacteria | *Oligotropha* |
| Bacteria α-Proteobacteria | *Parvibaculum* |
| Bacteria α-Proteobacteria | *Parvularcula* |
| Bacteria α-Proteobacteria | *Phaeobacter* |
| Bacteria α-Proteobacteria | *Phenylobacterium* |
| Bacteria α-Proteobacteria | *Rhodoplanes* |
| Bacteria α-Proteobacteria | *Roseibacterium* |
| Bacteria α-Proteobacteria | *Roseobacter* |
| Bacteria α-Proteobacteria | *Roseomonas* |
| Bacteria α-Proteobacteria | *Yangia* |
| Bacteria β-Proteobacteria | *Alicycliphilus* |
| Bacteria β-Proteobacteria | Candidatus *Symbiobacter* |
| Bacteria β-Proteobacteria | *Limnohabitans* |
| Bacteria β-Proteobacteria | *Ottowia* |
| Bacteria β-Proteobacteria | *Polaromonas* |
| Bacteria β-Proteobacteria | *Roseateles* |
| Bacteria β-Proteobacteria | *Snodgrassella* |
| Bacteria β-Proteobacteria | *Verminephrobacter* |
| Bacteria δ-Proteobacteria | *Anaeromyxobacter* |
| Bacteria δ-Proteobacteria | Candidatus *Babela* |
| Bacteria δ-Proteobacteria | Candidatus *Desulfofervidus* |
| Bacteria δ-Proteobacteria | *Chondromyces* |
| Bacteria δ-Proteobacteria | *Corallococcus* |
| Bacteria δ-Proteobacteria | *Desulfatibacillum* |
| Bacteria δ-Proteobacteria | *Desulfocapsa* |
| Bacteria δ-Proteobacteria | *Desulfohalobium* |
| Bacteria δ-Proteobacteria | *Desulfomicrobium* |
| Bacteria δ-Proteobacteria | *Desulfomonile* |
| Bacteria δ-Proteobacteria | *Desulfovibrio* |
| Bacteria δ-Proteobacteria | *Desulfurivibrio* |
| Bacteria δ-Proteobacteria | *Haliangium* |
| Bacteria δ-Proteobacteria | *Myxococcus* |
| Bacteria δ-Proteobacteria | *Pajaroellobacter* |
| Bacteria δ-Proteobacteria | *Pelobacter* |
| Bacteria δ-Proteobacteria | *Syntrophobacter* |
| Bacteria δ-Proteobacteria | *Syntrophus* |
| Bacteria δ-Proteobacteria | *Vulgatibacter* |
| Bacteria ε-Proteobacteria | *Campylobacter* |
| Bacteria ε-Proteobacteria | *Sulfurospirillum* |
| Bacteria ε-Proteobacteria | *Wolinella* |
| Bacteria γ-Proteobacteria | *Allofrancisella* |
| Bacteria γ-Proteobacteria | *Brenneria* |
| Bacteria γ-Proteobacteria | *Endozoicomonas* |
| Bacteria γ-Proteobacteria | *Fluoribacter* |
| Bacteria γ-Proteobacteria | *Hahella* |
| Bacteria γ-Proteobacteria | *Legionella* |
| Bacteria γ-Proteobacteria | *Luteibacter* |
| Bacteria γ-Proteobacteria | *Obesumbacterium* |
| Bacteria γ-Proteobacteria | *Pectobacterium* |
| Bacteria γ-Proteobacteria | *Saccharophagus* |
| Bacteria γ-Proteobacteria | *Simiduia* |
| Bacteria γ-Proteobacteria | *Thiolapillus* |
| Bacteria Proteobacteria | *Acidithiobacillus* |
| Bacteria Spirochaetes | *Leptospira* |
| Bacteria Spirochaetes | *Turneriella* |
| Bacteria Synergistetes | *Acetomicrobium* |
| Bacteria Synergistetes | *Cloacibacillus* |
| Bacteria Synergistetes | *Thermanaerovibrio* |
| Bacteria Tenericutes | *Acholeplasma* |
| Bacteria Tenericutes | Candidatus *Izimaplasma* |
| Bacteria Tenericutes | *Mesoplasma* |
| Bacteria Tenericutes | *Spiroplasma* |
| Bacteria Tenericutes | *Ureaplasma* |
| Bacteria Thermobaculum | *Thermobaculum* |
| Bacteria Thermotogae | *Fervidobacterium* |
| Bacteria Thermotogae | *Pseudothermotoga* |
| Bacteria Thermotogae | *Thermosipho* |
| Bacteria Verrucomicrobia | *Akkermansia* |
| Bacteria Verrucomicrobia | *Methylacidiphilum* |
| Bacteria Verrucomicrobia | *Opitutus* |
| Bacteria Verrucomicrobia | *Verrucomicrobium* |
| Fungi Ascomycota | *Capronia* |
| Fungi Ascomycota | *Lachancea* |
| Fungi Ascomycota | *Leptosphaeria* |
| Fungi Ascomycota | *Ogataea* |
| Fungi Ascomycota | *Paraphaeosphaeria* |
| Fungi Ascomycota | *Pochonia* |
| Fungi Ascomycota | *Verticillium* |
| Fungi Basidiomycota | *Anthracocystis* |
| Fungi Basidiomycota | *Auricularia* |
| Fungi Basidiomycota | *Coniophora* |
| Fungi Basidiomycota | *Dichomitus* |
| Fungi Basidiomycota | *Punctularia* |
| Fungi Basidiomycota | *Rhodotorula* |
| Fungi Basidiomycota | *Schizophyllum* |
| Fungi Basidiomycota | *Trametes* |
| Fungi Basidiomycota | *Tsuchiyaea* |
| Fungi Basidiomycota | *Ustilago* |
| Bacteria Planctomycetes | *Fuerstia* |
| Algae Stramenopiles | *Aureococcus* |
| Protist Stramenopiles | *Saprolegnia* |
| Gluconate 5-dehydrogenase [EC:1.1.1.69] | K00046 |
| Dihydroflavonol-4-reductase [EC:1.1.1.219] | K00091 |
| Acetyl-CoA C-acetyltransferase [EC:2.3.1.9] | K00626 |
| Chloramphenicol O-acetyltransferase [EC:2.3.1.28] | K00638 |
| Glycine C-acetyltransferase [EC:2.3.1.29] | K00639 |
| Diamine N-acetyltransferase [EC:2.3.1.57] | K00657 |
| Maltose O-acetyltransferase [EC:2.3.1.79] | K00661 |
| 3-phosphoshikimate 1-carboxyvinyltransferase [EC:2.5.1.19] | K00800 |
| Anthranilate synthase component I [EC:4.1.3.27] | K01657 |
| Isopentenyl-diphosphate delta-isomerase [EC:5.3.3.2] | K01823 |
| GMP synthase (glutamine-hydrolysing) [EC:6.3.5.2] | K01951 |
| Carbamoyl-phosphate synthase large subunit [EC:6.3.5.5] | K01955 |
| Sulfonate/nitrate/taurine transport system permease protein | K02050 |
| Flagellar hook protein FlgE | K02390 |
| Flagellar motor switch protein FliN/FliY | K02417 |
| Aspartyl-tRNA(Asn)/glutamyl-tRNA (Gln) amidotransferase subunit B [EC:6.3.5.6 6.3.5.7] | K02434 |
| UDP-N-acetyl-D-galactosamine dehydrogenase [EC:1.1.1.-] | K02474 |
| Transcriptional antiterminator NusG | K02601 |
| Small subunit ribosomal protein S15 | K02956 |
| Two-component system, chemotaxis family, sensor kinase CheA [EC:2.7.13.3] | K03407 |
| Beta-glucosidase [EC:3.2.1.21] | K05350 |
| Alpha-L-rhamnosidase [EC:3.2.1.40] | K05989 |
| Uncharacterized protein | K07158 |
| Basic membrane protein A and related proteins | K07335 |
| Hypothetical protein | K09702 |
| Glutamate/aspartate transport system ATP-binding protein [EC:3.6.3.-] | K10004 |
| Putative multiple sugar transport system substrate-binding protein | K10546 |
| DNA ligase 1 [EC:6.5.1.1] | K10747 |
| tRNA Asp | K14221 |
